# Sequential Therapy with Saratin, Bevacizumab and Ilomastat to Prolong Bleb Function following Glaucoma Filtration Surgery in a Rabbit Model

**DOI:** 10.1371/journal.pone.0138054

**Published:** 2015-09-22

**Authors:** Gina M. Martorana, Jamie L. Schaefer, Monica A. Levine, Zachary L. Lukowski, Jeff Min, Craig A. Meyers, Gregory S. Schultz, Mark B. Sherwood

**Affiliations:** 1 Department of Ophthalmology, COM, University of Florida, 1600 SW Archer Rd., Gainesville, FL, 32610, United States of America; 2 Department of Ob/Gyn and Institute of Wound Healing, COM, University of Florida, 1600 SW Archer Rd., Gainesville, FL, 32610, United States of America; University of Oklahoma Health Sciences Center, UNITED STATES

## Abstract

To determine if sequential treatment with Bevacizumab (Avastin), a monoclonal, VEGF antibody that blocks angiogenesis; Saratin, a 12 kD polypeptide with anti-inflammatory and anti-thrombotic properties; and Ilomastat, a matrix metalloproteinase (MMP) inhibitor, prolongs bleb life following glaucoma filtration surgery (GFS) in a rabbit model. Thirty-two New Zealand White rabbits (eight rabbits per group) underwent GFS in the left eye. Group 1 received a perioperative injection of both Saratin and Bevacizumab, and later, subconjuctival injections of Ilomastat on days 8 and 15. Group 2 received only Saratin perioperatively, and also received Ilomastat injections on days 8 and 15. Group 3, the negative control, received a single perioperative injection of Balanced Saline Solution (BSS) along with post-operative BSS injections on days 8 and 15. Group 4, the positive control, received topical treatment with Mitomycin-C (MMC) at the time of surgery with no further treatment. Blebs were evaluated by an observer masked to treatment every third day. Histology was obtained on two eyes in each group on post-op day twelve as well as all eyes following bleb failure. Eyes in group 1 had a mean bleb survival time of 29 ± 2.7 days, whereas those in group 2 that received the experimental treatment without Bevacizumab had a mean survival time of 25.5 ± 2.7 days. An ANOVA test showed that the Saratin/Ilomastat/Bevacizumab group demonstrated a significant prolongation of bleb survival compared to the BSS control—mean survival time of 19.7 ±2.7 days—(p = 0.0252) and was not significantly different from the MMC positive control group (p = 0.4238)—mean survival time of 32.5 ± 3.3. From tissue histology at day 12, the four different groups showed marked differences in the cellularity and capsule fibrosis. The MMC eyes showed minimal cellularity, were avascular and had minimal fibrous tissue. BSS group showed high cellularity, moderate to high fibrosis, and thicker and more defined capsules than either of the treatment groups and the positive control. Both the Saratin/Ilomastat/Bevacizumab and Saratin/Ilomastat only eyes showed moderate cellularity with minimal fibrosis, with less cellularity and fibrosis present in the triple treatment group. Sequential therapy with multiple agents, including Bevacizumab, prolonged bleb function following GFS in the rabbit model and were significantly better than the negative BSS control. The experimental group did not show the same surface tissue histological thinning and side effects associated with MMC treatment.

## Introduction

The central objective of glaucoma therapy is to reduce intraocular pressure (IOP) to levels considered safe for the optic nerve in order to preserve visual function. When maximally tolerated medical management and laser surgery are insufficient to control the progression of visual deterioration, glaucoma filtration surgery (GFS) is required. GFS shunts aqueous humor from the anterior chamber to the sub-Tenon’s space and has demonstrated large and sustained decreases in IOP [1,2,3]. Post-operative wound healing will largely dictate the outcome of a glaucoma surgery by determining the amount of resistance encountered in the bleb [3]. Failure is generally due to excessive sub-conjunctival and episcleral fibrosis at the site of filtration as a result of fibroblast migration and accumulation, collagen deposition, and angiogenesis. Localized administration of antimetabolites 5-fluorouracil (5-FU) and Mitomycin-C (MMC) are currently used in conjunction with topical steroids to help reduce or prevent scarring and failure of the filtering bleb; however, these agents lack specificity and increase the risk of adverse events [4].

The concept of more targeted anti-scarring therapies that minimize external tissue toxicity have been pursued by multiple investigators [5,6]. CAT 152, an anti-TGF beta agent, showed initial promise, but the phase three study was halted early because it did not demonstrate efficacy. Another therapy, Ilomastat, has been shown to prolong bleb survival by inhibiting the matrix metalloproteinases (MMPs) involved in regulating the degradation of the extracelluar matrix (ECM) and wound contracture [5,7]. It successfully improved surgical outcome when used on its own in an animal model; yet, it was shown to be more effective when used in conjunction with other targeted therapies [8]. Wounds in different tissues are characterized by having elevated levels of pro-inflammatory cytokines and matrix metalloproteinases (MMPs) in both extracellular fluid and the tissue that sustained the trauma [9]. Stimulatory proteins are utilized in the early response to injury which in turn stimulates the production of MMPs in fibroblasts. MMPs degrade damaged components of the extracellular matrix which ultimately allows for cellular adherence and are responsible for remodeling of the new matrix [10]. The levels and duration of expression of these factors must be tightly controlled to prevent excessive scarring or keloid formation. MMPs are activated in the remodeling phase of the wound healing process. With the addition of Ilomastat post-operatively, the potential for excessive effects of MMPs in the late stages of wound healing is reduced, leading to a longer bleb life and decreased scarring [7,11].

In previous studies [12], a single agent, Saratin, a protein known for its ability to control platelet aggregation, was given to rabbit models via sub-conjunctival injection post-operatively. These studies demonstrated that a single injection of Saratin, given post-operatively leads to significantly prolonged bleb survival time over the Balanced Saline Solution (BSS) group. Though there was a statistically significant difference from the BSS group, the survival time was less than the MMC positive control group. When more Saratin was injected post-operatively, if anything, there was a decrease in bleb survival [12] and external tissue toxicity was noted with high doses.

One of the more recent compounds under investigation for improving bleb survival is Bevacizumab (Avastin), a monoclonal antibody that targets vascular endothelial growth factor (VEGF) to block angiogenesis [13,14]. Initially, Avastin was used in the treatment of cancer but in more recent studies it has been used in the management of neovascular glaucoma because of its ability to suppress iridocorneal angle neovascularization and peripheral anterior synechae (PAS) [15]. Further studies in non neovascular glaucoma patients showed improved “bleb appearance” (vascularity, etc) when another anti-VEGF, Ranibizumab, was administered [14].

This current study evaluates the efficacy of a combination treatment utilizing intraoperative subconjuctival injections of Bevacizumab and Saratin in combination with post-operative injections of Ilomastat to prolong bleb survival following glaucoma filtration surgery (GFS) in a previously established rabbit model. The two study endpoints were to compare the overall duration of bleb survival of this treatment regimen compared to a Balanced Saline Solution (BSS) negative control and a MMC positive control, and to examine bleb tissue histology for evidence of tissue changes which may be associated with an increased likelihood of late adverse event related to bleb function.

## Materials and Methods

### Study design

Thirty-two New Zealand White (NZW) rabbits, weighing between 2 kg and 4 kg, were randomized into four different treatment groups. All animal experiments performed were approved by the University of Florida’s Institutional Animal Care and Use Committee (study number: #201104864) and adhered to the ARVO Statement for the Use of Animals in Ophthalmic and Vision Research.

The left eyes of thirty-two NZW rabbits underwent glaucoma filtration surgery with one surgeon (MBS) performing all of the procedures; the right eyes were not operated on. At the end of the procedure, the rabbits in group 1 (n = 8) were given a perioperative injection of 0.1 mL Saratin at 5 mg/ml and 1.25 mg of Bevacizumab in 0.05 mL under the superior conjunctiva, adjacent to the bleb, with a 32-gauge needle. In addition, these eyes received 0.1 mL injections of Ilomastat at 100μM on postoperative days (PODs) 8 and 15. Rabbits in group 2 (n = 8) received a 0.1 mL injection of only Saratin at 5 mg/mL at the end of the procedure, and also received the same postoperative injections of Ilomastat as group 1 rabbits on PODs 8 and 15. The rabbits in group 3 (n = 8) were given a 0.1 ml Balanced Saline Solution (BSS) injection under the superior conjunctiva at the end of surgery. Group 3 also received postoperative injection of 0.1 ml of BSS on days 8 and 15 to maintain consistency with the experimental groups. Finally, the rabbits in the positive control group 4 (n = 8) received a single topical treatment of 0.4 mg/mL of Mitomycin-C applied for three minutes with a Weck sponge (Alcon Surgical, Fort Worth, TX) and were not given any post-operative injections.

### Glaucoma filtering operation

A combination intramuscular injection of Ketamine (50mg/kg) (“Ketaject”, Phoenix Pharmaceuticals, Inc., St. Joseph, Mo) and Xylazine (10mg/kg) (“Xyla-ject”, Phoenix Pharmaceuticals, Inc., St. Joseph, Mo) was used to anesthetize the rabbits. Prior to surgery, a topical anesthetic was also administered, 0.1% Proparacaine eye drop (Bausch & Lomb, Tampa, FL). The technique utilized for the glaucoma filtration surgeries was similar to the procedures described in our previous publications [12,16]. In brief, the eyelids were retracted with an eyelid speculum. The eye was rotated inferiorly by placing a partial-thickness, corneal traction suture in the superior cornea. A standard 5 mm measured fornix-based conjunctival flap was created at the limbus of the eye in the superior lateral quadrant. Blunt dissection was used to help separate the conjunctiva from Tenon’s capsule. At this point for the group 4 rabbits, a 0.4 mg/ml Mitomycin-C soaked 5 mm x 5 mm partial-thickness Weck cell was placed locally between the conjunctiva/Tenon’s capsule and the sclera for 3 minutes. The sponge was removed after the three minutes and the area was washed with 30 ml of saline.

Next, for rabbits in all groups, a clear corneal paracentesis tract was fashioned using a # 75 Beaver™ blade (Becton Dickinson & Co., Franklin Lakes, NJ) in the superonasal quadrant. To maintain the anterior chamber, a viscoelastic material (Healon® 10mg/ml, Pharmacia & Upjohn) was injected through the paracentesis tract into the chamber. About 1 mm posterior to the limbus, a 25-gauge needle was then used to create a beveled, full-thickness tract through the sclera into the anterior chamber. This was followed by insertion of a 22-gauge, IV cannula (Insyte® Becton Dickinson Vascular Access, Sandy, UT) along this scleral tract into the anterior chamber. The needle of the cannula was pulled out. The remaining cannula was positioned beyond the pupillary margin in order to prevent iris occlusion of the tube foramen. The scleral end of the cannula was trimmed so not to protrude more than approximately 1 mm from the point of insertion. The tube was then anchored to the sclera with a 10–0 nylon suture (Ethicon Inc., Somerville, NJ).

Closure of the conjunctiva/Tenon’s capsule was completed at the limbus, using a running suture of 8–0 absorbent suture material (Vicryl®, Ethicon Inc., Somerville, NJ) in a watertight fashion. Saline solution was injected into the anterior chamber at the end of the procedure via the paracentesis tract to elevate the bleb. A Seidel test with fluorescein was then performed to ensure that there was no leakage of the bleb. The rabbits in the Saratin/ Bevacizumab /Ilomastat, Saratin/Ilomastat, and BSS treatment groups (Groups 1, 2, and 3) were given their assigned sub-conjunctival injections. 0.1 mL Saratin/ Bevacizumab (5mg/mL / 1.25mg/0.05mL), 0.1mL Saratin (5mg/mL), or 0.1 mL BSS respectively, was injected with a 32-gauge needle, adjacent to the blebs, immediately following the surgery in a masked fashion. The surgical encounter was completed with the application of a topical Neomycin and Dexamethasone ointment. By Institutional Animal Care and Use Committee (IACUC) request, rabbits in all groups were given oral meloxicam for three days after surgery as an analgesic and as an anti-inflammatory agent.

### Post-operative injections and clinical evaluations

Following the operation, an examiner masked to the treatment groups inspected the eyes of the rabbits every three days. The presence of bleb elevation as well as any complications that may have resulted from the surgeries or various treatments was evaluated. Examination included the assessing the eye conjunctival injection, bleb leakage, edema, mal-positioning of the tube, shallowing of the anterior chamber, hyphema or sub-conjunctival hemorrhage, and lens or corneal opacification. The appearance of a flat bleb on two consecutive examinations was declared as bleb failure, of which the first of the two dates was recorded as the endpoint.

After the clinical examination, rabbits in the Saratin/ Bevacizumab /Ilomastat, Saratin/Ilomastat and BSS treatment groups (Groups 1, 2, and 3) were also given injections of 0.1 mL Ilomastat at 100μM or 0.1 ml BSS respectively on post-operative days 8 and 15 by a second, unmasked observer into the area of the bleb. Isoflurane (VetOne, Boise, ID) was used to anesthetize the rabbits during their post-operative examinations. For the rabbits requiring post-operative injections on day 8 and 15, a topical 0.1% proparacaine anesthetic eye drop was also administered, and a speculum was used to retract the eyelids. Non-toothed Bishop-Harmon forceps were used to tent the conjunctiva and, using a 32-gauge needle on a 1 ml syringe, the 0.1 ml injections were given adjacent to the bleb.

### Histology

The eyes of two rabbits from each treatment group were obtained on post-operative day 12 for the purpose of comparing the bleb tissue histology at the same post-operative time point for each and while all blebs still remained elevated. For all other rabbits, the eyes were obtained for histology after declaration of bleb failure.

The eyes were harvested by enucleating the entire globe. No gelatin was injected into the eyes. Following the harvesting of the eyes, the tissues were fixed in a 10% Neutral Buffered Formalin solution for 24 hours. The whole globes were placed in tissue cassettes and then processed using a Sakura Tissue-Tek VIP 5 tissue processor (Torrance, CA) through graded ethanol and xylene solutions. Infiltration of the tissues with paraffin (Richard-Allan Scientific, Kalamazoo, MI) was then followed by embedding the tissues (whole globes) on a Tissue Tek III embedding center. The globes were sectioned in the sagittal plane serially, and each section had an average thickness of 5 micrometers. The region of the eye where the bleb was located was identified during sectioning by the position of the end of the cannula. The resulting sections were then stained using standard Harris hematoxylin and eosin or Masson’s Trichrome once they were de-paraffinized (to deionized water as to allow for staining to take hold). Light microscopy was used to examine the slides in a semi-quantitative analysis. Tissues in the area of the bleb, close to the tube, were examined for collagen deposition (Trichrome), cellular infiltration and tissue thickness surrounding the capsule. A trained histologist, masked to the treatment group, examined five representative slides from each eye and graded them on a semi-quantitative zero to three plus scale. Sections were photographed using an Olympus Vanox microscope with an attached Canon EOS T1i digital camera.

### Statistical analysis

Analysis of Variance (ANOVA) testing was used to determine any significant difference in bleb survival duration among the Saratin/Ilomastat/ Bevacizumab, Saratin/Ilommastat, MMC, and BSS treatment groups. Two post-hoc tests—Tukey’s Honestly Significantly Different (HSD) test and Fisher’s Least Significant Difference (LSD) test—were then used to further compare pairs of relevant groups.

## Results

### Bleb survival

**Fig 1:**
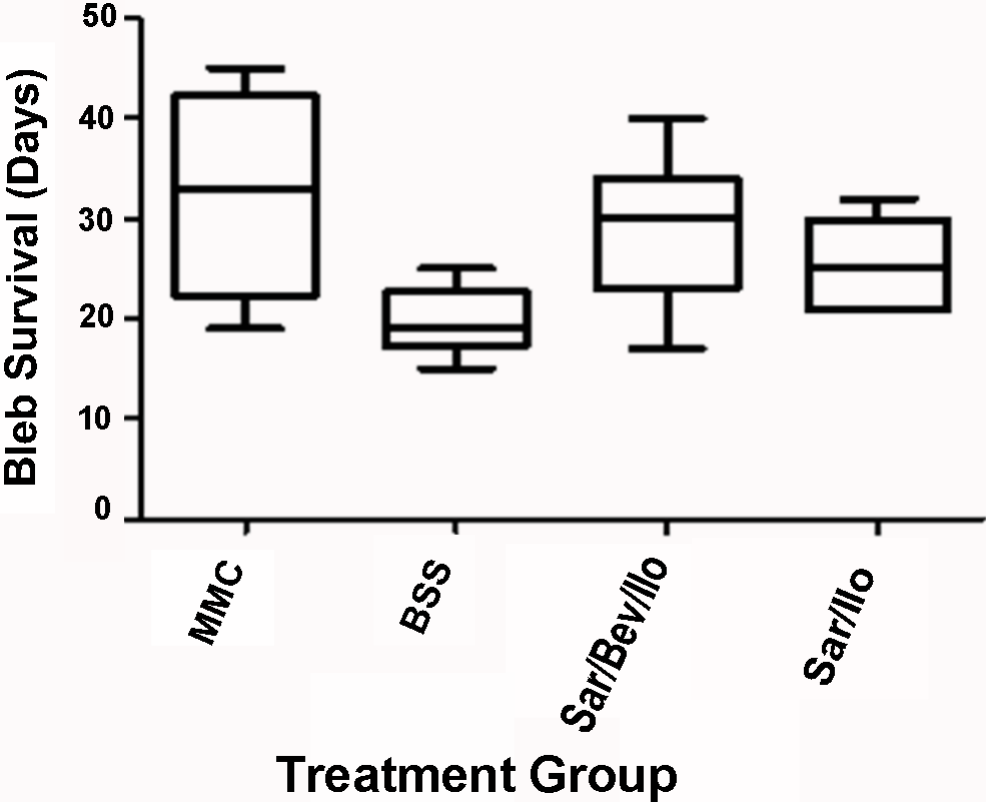
Box and Whisker bleb survival plot of eyes treated with MMC, BSS, Saratin/Bevacizumab/Ilomastat, or Saratin/Ilomastat. For each rabbit, bleb failure was declared after the bleb appeared flat in two consecutive masked clinical examinations. The first of the two dates was recorded as the endpoint.

**Fig 2:**
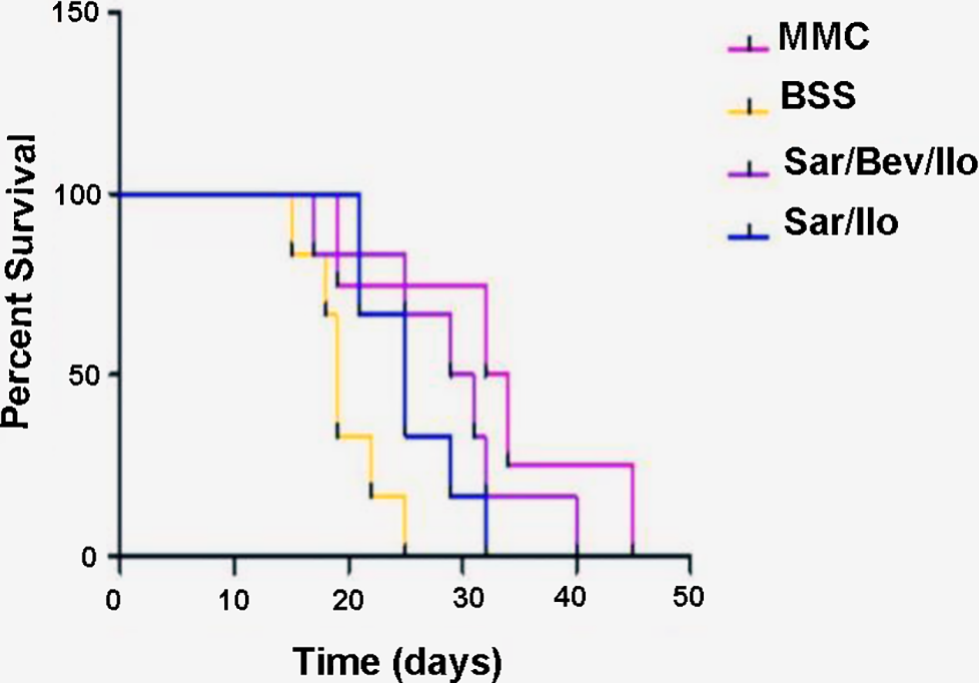
Kaplan-Meier bleb survival plot of eyes treated with MMC, BSS, Saratin/Bevacizumab/ Ilomastat, or Saratin/Ilomastat. For each rabbit, bleb failure was declared after the bleb appeared flat in two consecutive masked clinical examinations. The first of the two dates was recorded as the endpoint.

As can be seen in Figs 1 and 2, eyes in group 1 that received the triple experimental treatment with Bevacizumab (Avastin), Saratin, and Ilomastat had a mean survival time of 29 ± 2.7 days, whereas those in group 2 had a mean bleb survival time of 25.5 ± 2.7 days. The group 1 eyes receiving Saratin, Bevacizumab, and Ilomastat had a significant improvement in bleb duration over the negative control, BSS (group 3) eyes, which averaged 19.7 ± 2.7 days of survival (p = 0.0252). Compared to the MMC positive control, the group 1 eyes that received all three agents showed no significant difference (p = 0.4238) in length of bleb survival. One way ANOVA showed that group 2 eyes which received Saratin and Ilomastat alone were not statistically different compared to the BSS group (p = 0.1446). Groups 1 and 2 were also compared to each other and were found not to be statistically different (p>0.05). Clinical observation and post-operative histology found minimal, if any, complications, signs of toxicity, or fibrosis at the surgical site in the two experimental groups (Groups 1 and 2).

### Clinical evaluation

During post-operative clinical examinations, some bleb avascularity was noted among the MMC rabbits. Saratin/Bevacizumab /Ilomastat eyes showed normal indicators of tissue change after surgery, with moderate injection two days after surgery. Saratin/Ilomastat eyes showed similar results, but the conjunctival injection lasted for a longer period of time (3 or 4 days).

### Histology

**Fig 3:**
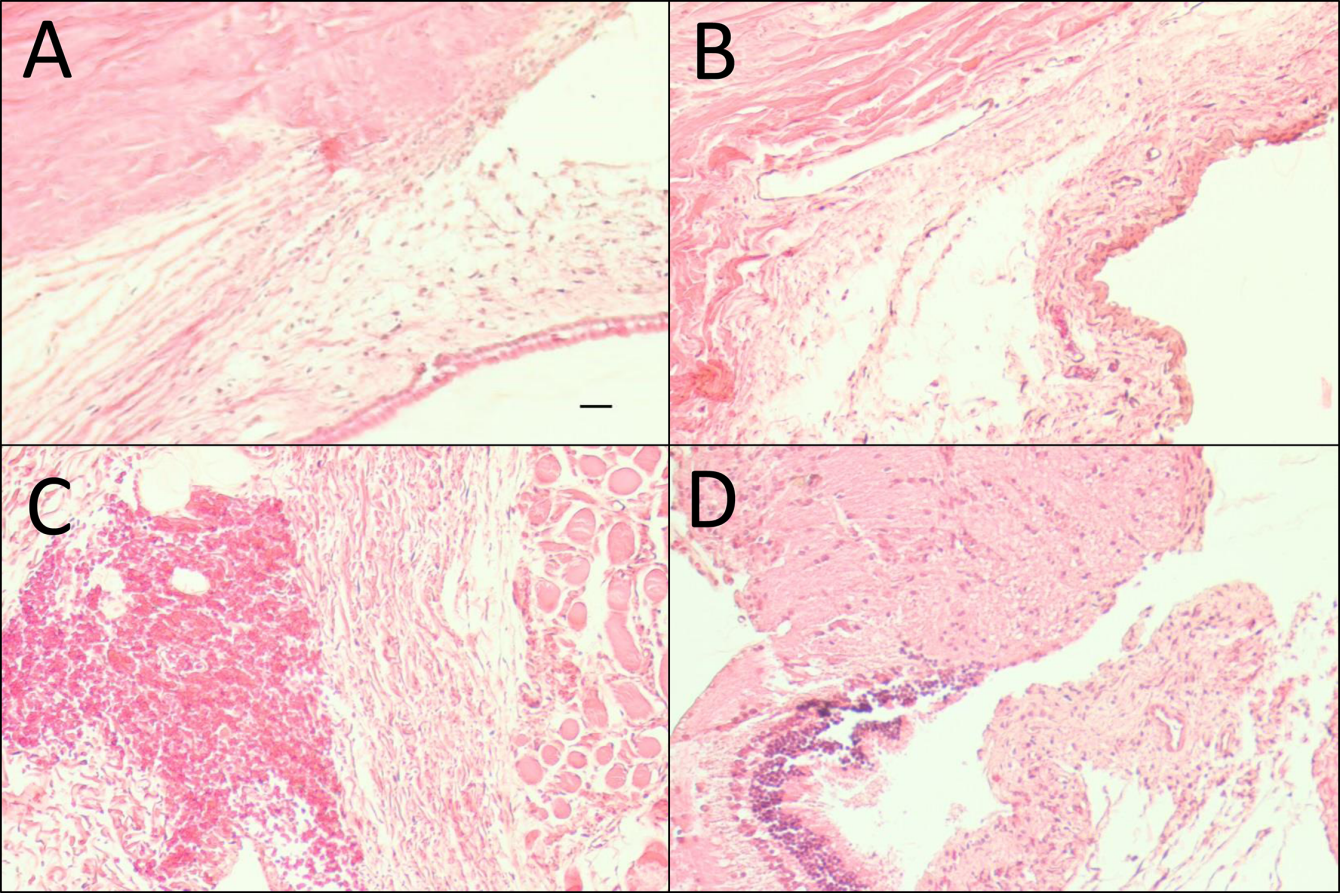
Harris Hematoxylin and Eosin stained representative sections near implants site taken on Post-Operative Day 12 (magnification 10x, black scale bars = 100 μm). (A) Saratin/Ilomostat; (B) Saratin/Bevacizumab/Ilomostat; (C) BSS; (D) MMC (all representative, come from areas of roughly the same location in relation to tube placement).

**Fig 4:**
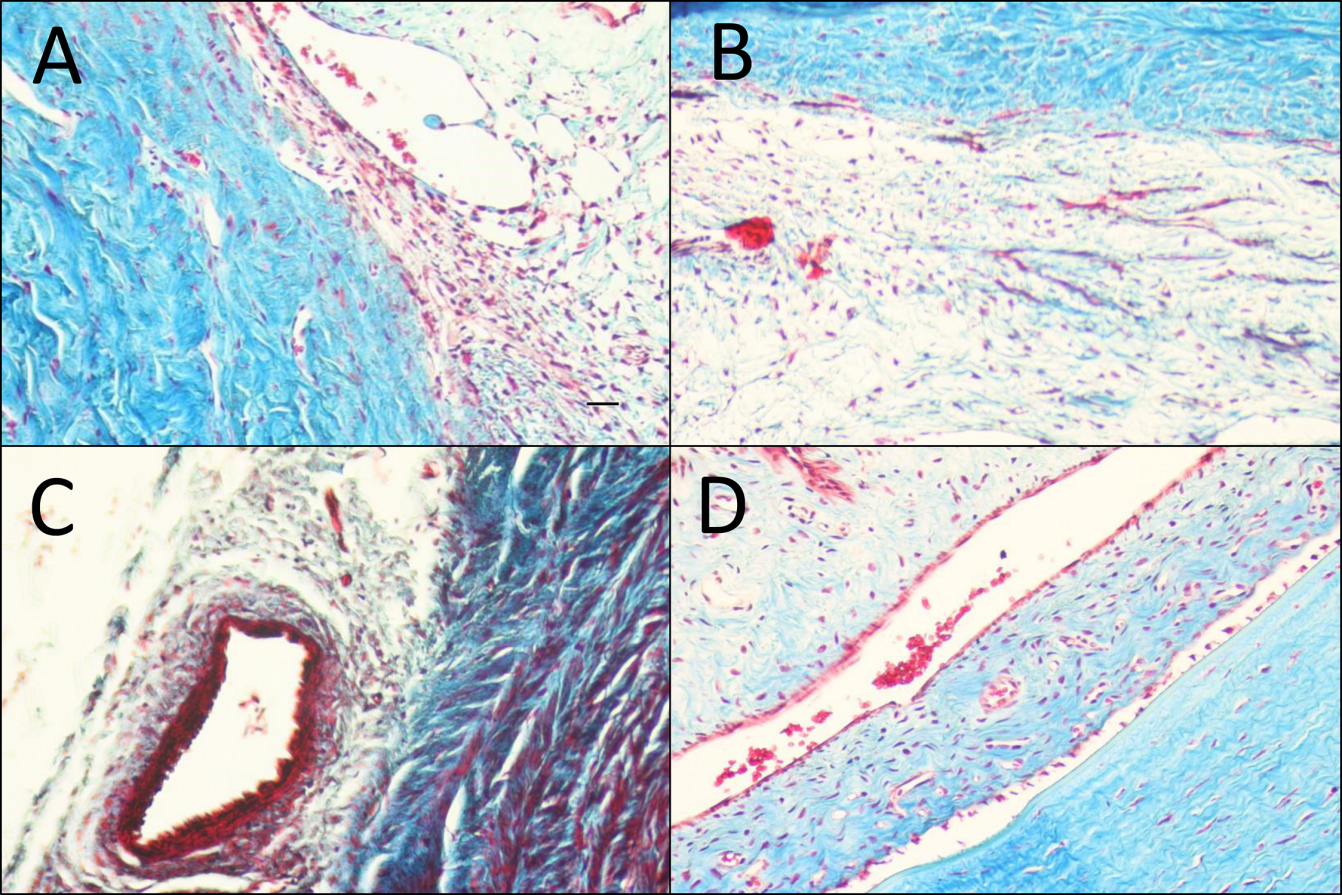
Masson’s Trichrome stained representative sections near implants site taken from POD 12, Masson’s Trichrome (magnification 10x, black scale bars = 100 μm). (A) Saratin/Ilomostat; (B) Saratin/Bevacizumab/Ilomostat; (C) BSS; (D) MMC (all representative, come from areas of roughly the same location in relation to tube placement)

Histological examination of bleb tissue samples and qualitative analysis (Table 1) were performed on POD 12 showed differences in implant site morphology among the treatment groups. Group 1 and 2 eyes showed normal vascularity and little to no capsule formation, with markedly thicker conjunctival tissues than the MMC (group 4) eyes. No distinct capsule around the end of the cannula was seen in rabbits in the MMC group. By comparison, the rabbits that received BSS injections consistently formed a fibrotic capsule around the implant. The Harris hematoxylin and eosin (H&E) stained images (Fig 3) demonstrate cellularity and the Masson’s Trichrome stained images (Fig 4) demonstrate fibrosis of post-op day 12. As can be seen in Table 1, masked semi-quantitative histological analysis showed reduced fibrosis and cellularity in the multi-treatment eyes compared with BSS control, but more than the Mitomycin-C positive control. Further examination of the 12-day samples showed that eyes treated with MMC had thinned, relatively avascular conjunctivas compared with the other groups. In contrast, Saratin/Bevacizumab/Ilomastat, Saratin/Ilomastat, and BSS-treated eyes displayed normal conjunctival morphology with an even distribution of goblet cells. Around the cannula site infiltration of vascular tissue and levels of fibrosis were minimal (group 1) to moderate (group 2) to high (group 3) respectively.

**Table 1:** Histologic Changes Around End of Implant on Post-Operative Day 12.

Treatment Group	Fibrosis (Masson’s Trichrome)	Cellularity (H&E)
**S/B/I**	**+**	**++**
**S/I**	**++**	**++**
**BSS**	**+++**	**+++**
**MMC**	**+/-**	**-**

S/B/I = Saratin/Bevacizumab/Ilomastat; S/I = Saratin/Ilomastat; BSS = Balanced Saline Solution; MMC = Mitomycin-C;– = absent; +/- = weakly present; +, ++, +++ = present in increasing amounts, as graded by the masked observer.

## Discussion

Historically, MMC has been shown to reduce fibrosis and prolong bleb function in glaucoma patients [17]. Although it has been shown to increase the lifespan of blebs in both rabbits and humans [4,18] there is a risk associated with this non-specific treatment. Many studies have shown that MMC causes decreased vascularization of the Tenon’s capsule, and a substantial reduction of cellular activity at the wound site [19]. MMC is highly toxic in ophthalmic applications, and in human studies has been shown to cause increased incidence of bleb leak, blebitis, endophthalmitis and hypotony maculopathy [4]. Due to these side effects there has been a strong desire to find alternative, more targeted therapies for reducing bleb scarring. There are multiple pathways for the inflammatory response and scarring processes to take in the healing cascade. To block the early inflammatory phase, the protein Saratin which affects platelet adherence and inflammatory cell cascade, together with Avastin which blocks the action of VEGF and reduces tissue vascularity were given perioperatively. A broad spectrum metalloproteinase inhibitor, Ilomastat, was given later at 1 and 2 weeks post-operatively to reduce the contraction phase of wound healing. Targeting a single pathway may be only partially effective, because there are so many alternate routes and systems. Instead, by targeting several pathways at once, it may be possible to reduce the risk of increased use of alternate pathways leading to wound healing. It may also allow use of a reduced dose for each single agent to achieve a safer level of concentration. To date, single agents have not been shown to be as effective in maintaining bleb survival as Mitomycin-C [12,20].

The multi-treatment approach has been used for years in the treatment of cancer [21]. In many diseases, combinations of drugs have been shown to have a more widespread effect on cell function. Drugs can target different phases in the cell cycle or in a cascade sequence and, therefore lower and less cytotoxic doses of each drug may be effective [22].

Wound healing shows variations and nuances in different parts of the body, but the main processes are similar across all tissues including the eye. It is well documented that cutaneous wound healing consists of multiple overlapping phases starting with blood coagulation following incision [23] followed by an inflammatory phase, in which lymphatic vessels play a role in the immune response and the clearing of cellular by-products. In the final phase of wound healing, tissue maturation, and remodeling, including the development and deposition of collagen fibers from the fibroblast cells, the wound area is fully closed while blood vessels mature to support the cells [23]. By changing the rate and/or extent of collagen fiber deposition or capsule thickness, it would be possible to further elongate the life of the bleb after GFS, thus reducing the need of revisions.

Our study showed a significant improvement in bleb survival with multiple therapies compared to the BSS control and a statistically similar duration of bleb elevation to the MMC positive control. Less thinning of tissues and avascularity was noted compared to MMC, which could translate to less late-stage side effects. The reduced cellular infiltration and fibrosis compared to the BSS was associated with improved bleb duration.

Specificity and safety are paramount in the search for prolonging bleb survival and improving the overall surgical outcomes in GFS. The process of bleb failure is complex and involves a number of different mediators, such as inflammatory mediators, growth factors and structural proteins. The search for alternatives to nonspecific antimetabolites should continue to focus more on how to modify several of these pathways at once in order to discourage re-routing of the healing cascade.

## Conclusions

This study has demonstrated that the sequential use of multiple agents to target different modulators of wound healing prolongs bleb survival in this rabbit model of GFS. Histologically, there is less conjunctival thinning compared with MMC and a reduction in the scarring process that causes for bleb failure compared with BSS. Future studies to determine the most effective combination of agents, the most successful concentrations, and the optimization of the timing and method of their delivery may improve efficacy further while maintaining the histological integrity of the tissues.

## Supporting Information

S1 Dataset Summary of Individual Data. Sheet 1: Summary of Individual Eye Pressure Data; Sheet 2: Summary of Individual Length of Bleb Survival.
(XLSX)

S1 Photoset Photos of BSS only treatment. (ZIP)

S2 Photoset Photos of Mitomycin-C treatment, Part 1. (ZIP)

S3 Photoset Photos of Mitomycin-C treatment, Part 2. (ZIP)

S4 Photoset Photos of Saratin and Ilomastat treatment, Part 1. (ZIP)

S5 Photoset Photos of Saratin and Ilomastat treatment, Part 2. (ZIP)

S6 Photoset Photos of Saratin, Ilomastat, and Avastin treatment, Part 1. (ZIP)

S7 Photoset Photos of Saratin, Ilomastat, and Avastin treatment, Part 2. (ZIP)
