# Supplementary figures and images for: Sequential Therapy with Saratin, Bevacizumab and Ilomastat to Prolong Bleb Function following Glaucoma Filtration Surgery in a Rabbit Model (part 1 of 2)
Source: PLoS One. 2015 Sep 22;10(9):e0138054. doi: 10.1371/journal.pone.0138054 (PMC4578880; doi:10.1371/journal.pone.0138054)

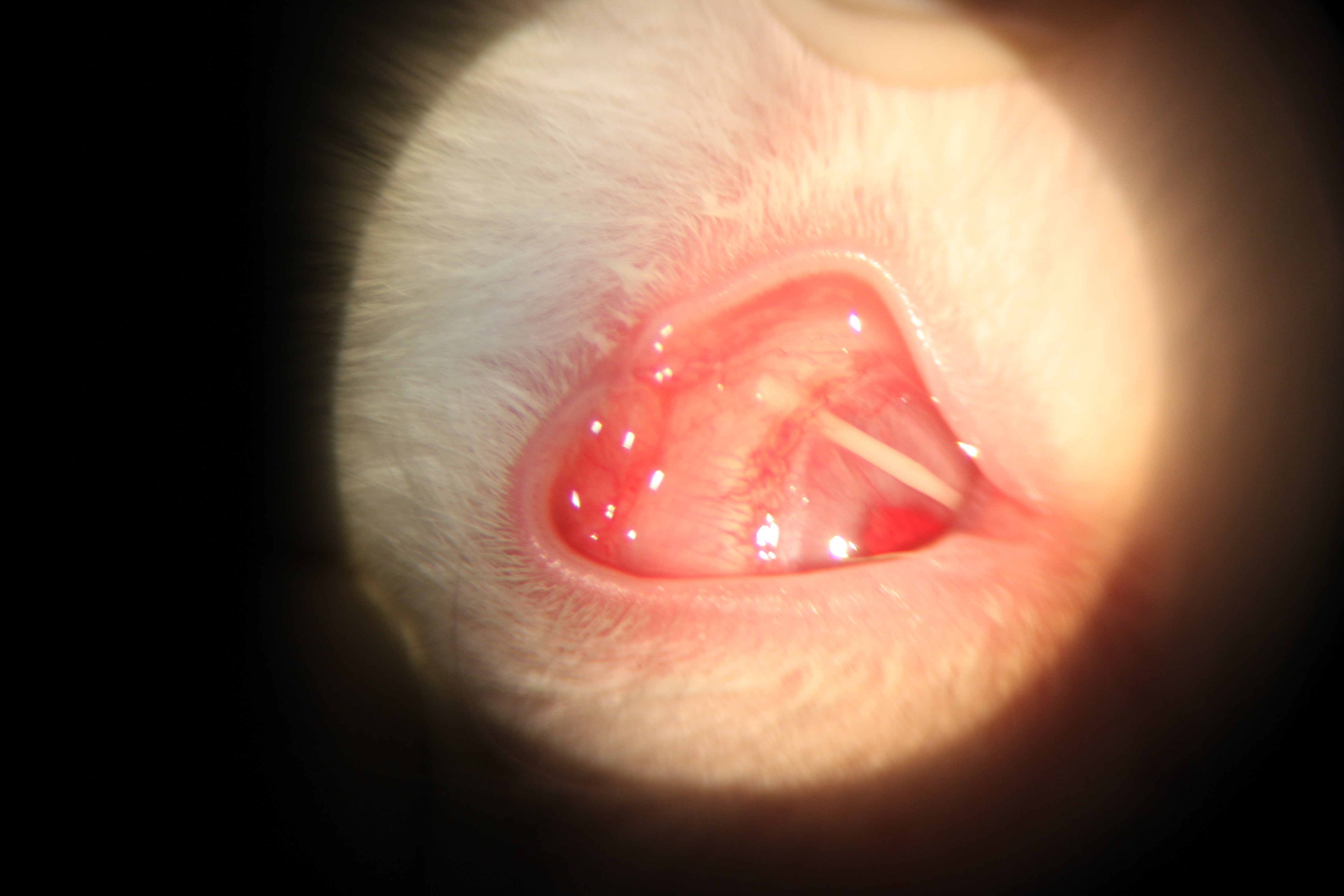

Supplement: S1 Photoset — (ZIP) [file pone.0138054.s002.zip › Multi Tx for Paper - BSS pics/IMG_1463.JPG]

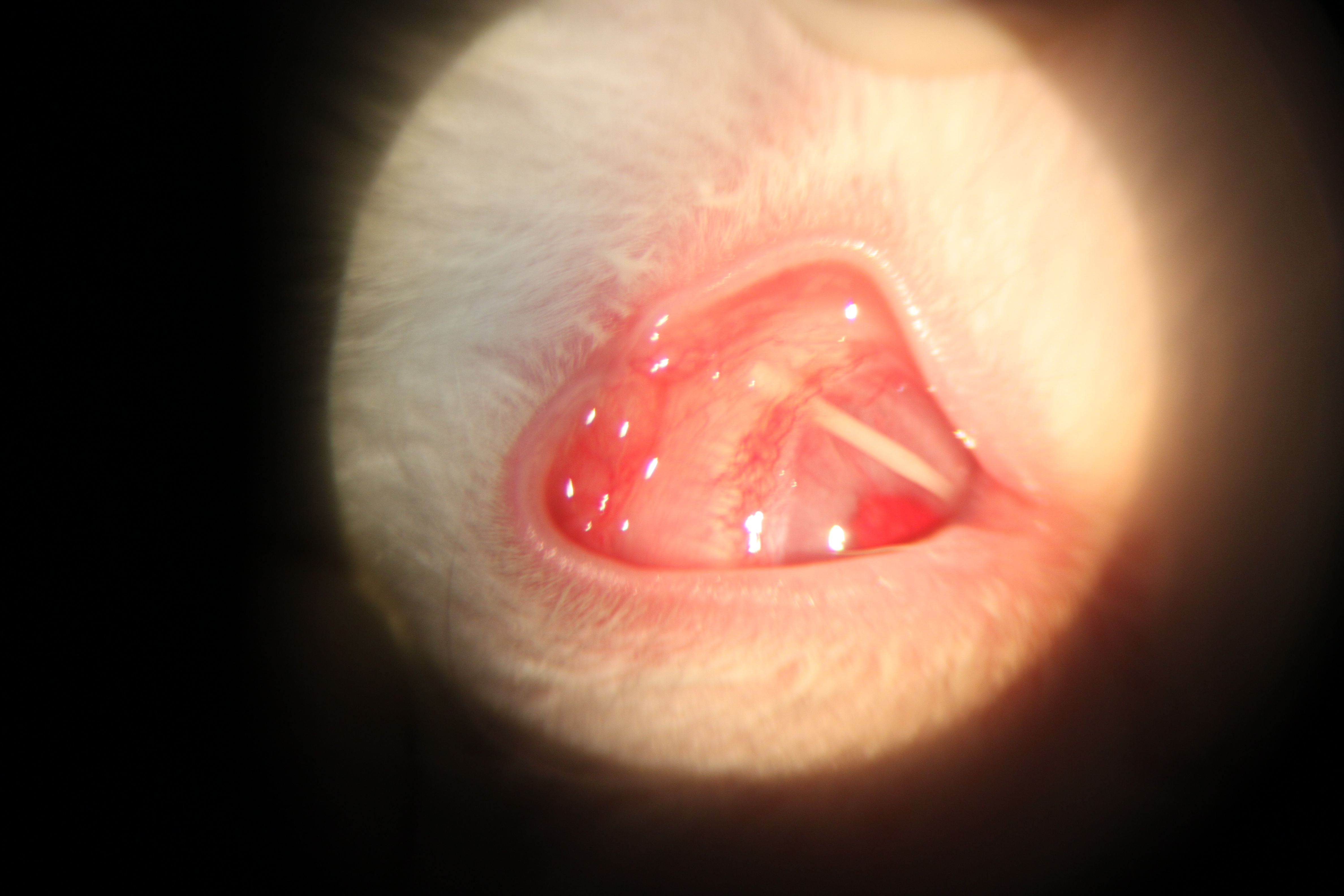

Supplement: S1 Photoset — (ZIP) [file pone.0138054.s002.zip › Multi Tx for Paper - BSS pics/IMG_1466 - Copy.JPG]

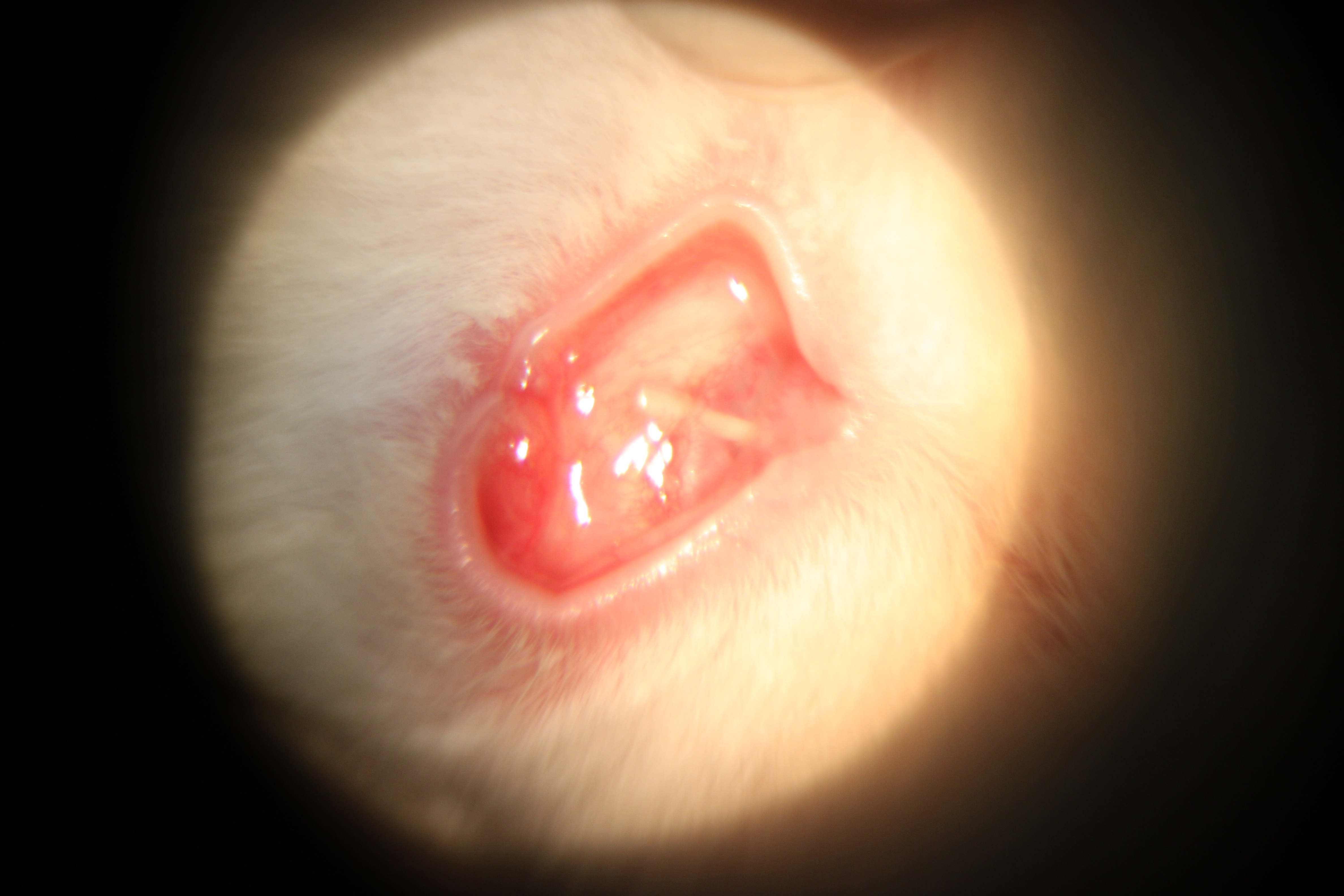

Supplement: S1 Photoset — (ZIP) [file pone.0138054.s002.zip › Multi Tx for Paper - BSS pics/IMG_2121.JPG]

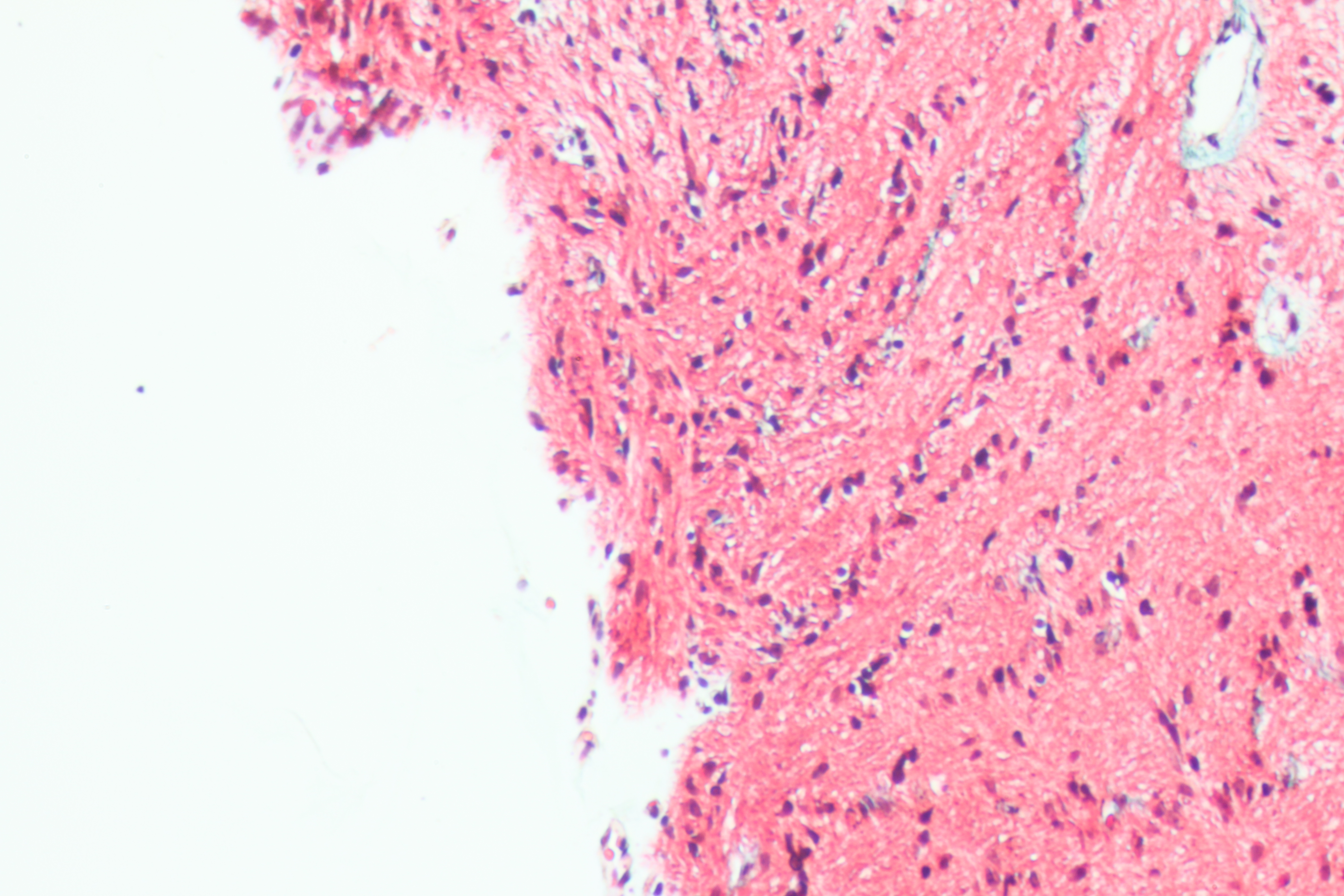

Supplement: S1 Photoset — (ZIP) [file pone.0138054.s002.zip › Multi Tx for Paper - BSS pics/IMG_6100.JPG]

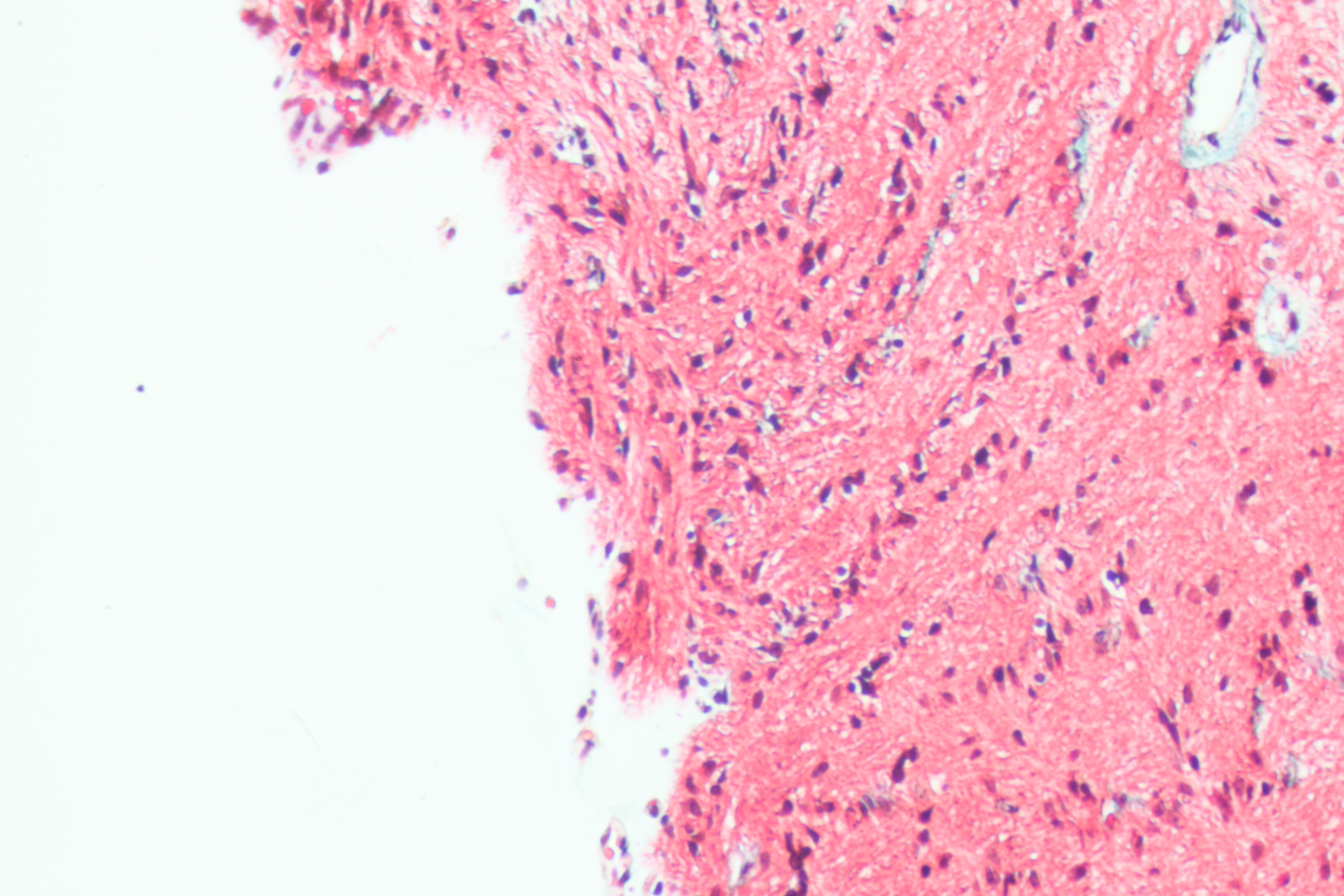

Supplement: S1 Photoset — (ZIP) [file pone.0138054.s002.zip › Multi Tx for Paper - BSS pics/IMG_6102.JPG]

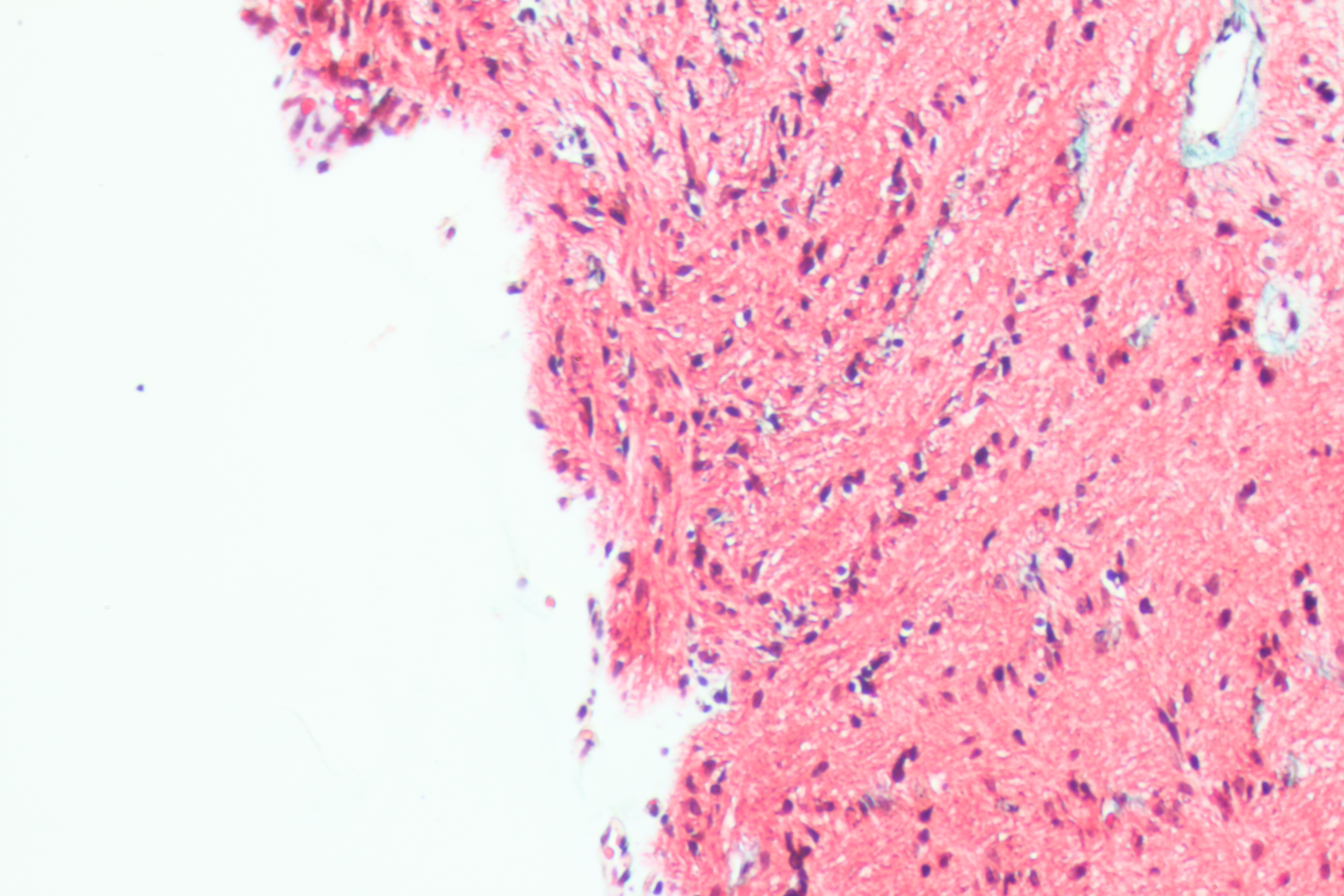

Supplement: S1 Photoset — (ZIP) [file pone.0138054.s002.zip › Multi Tx for Paper - BSS pics/IMG_6103.JPG]

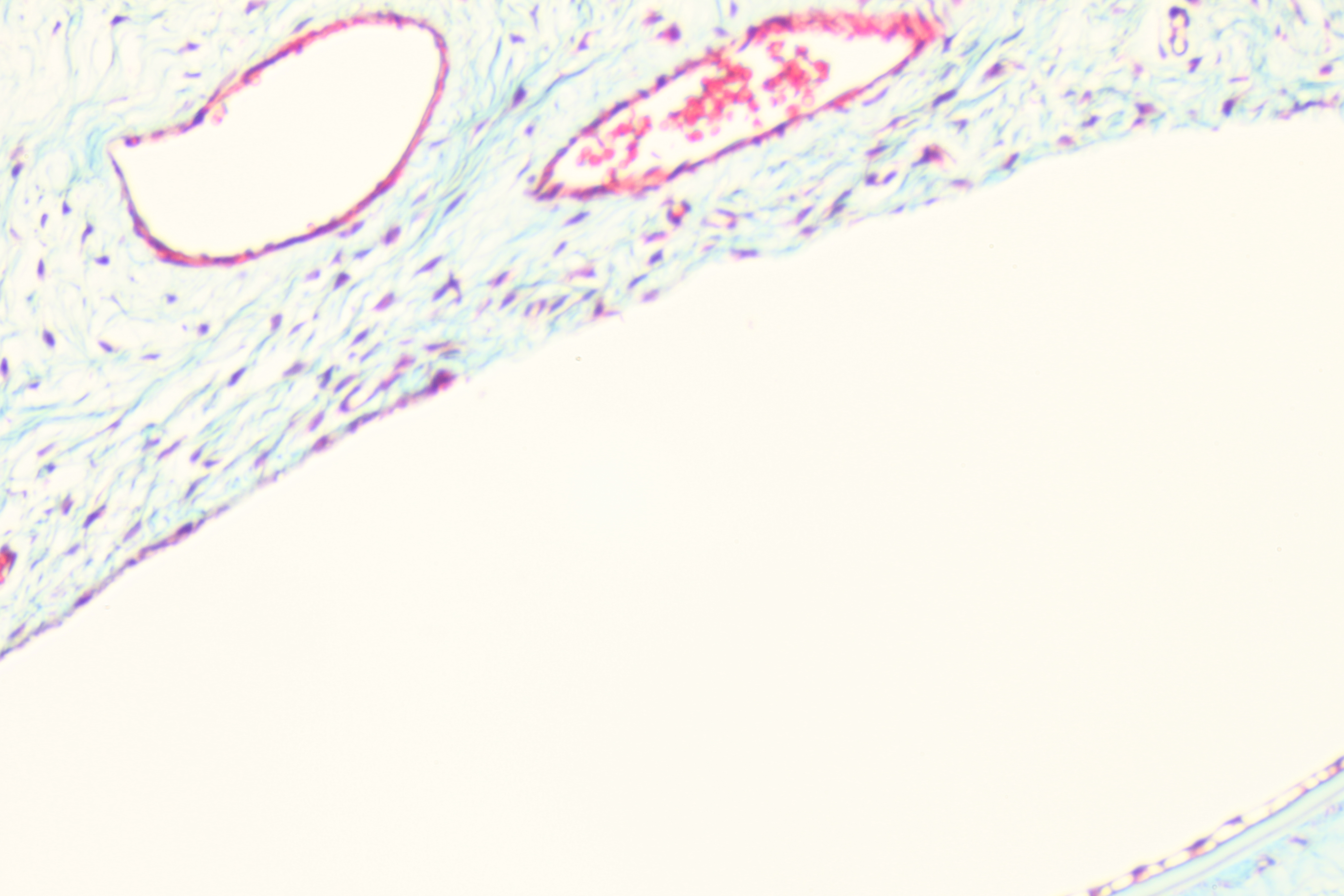

Supplement: S1 Photoset — (ZIP) [file pone.0138054.s002.zip › Multi Tx for Paper - BSS pics/IMG_6116.JPG]

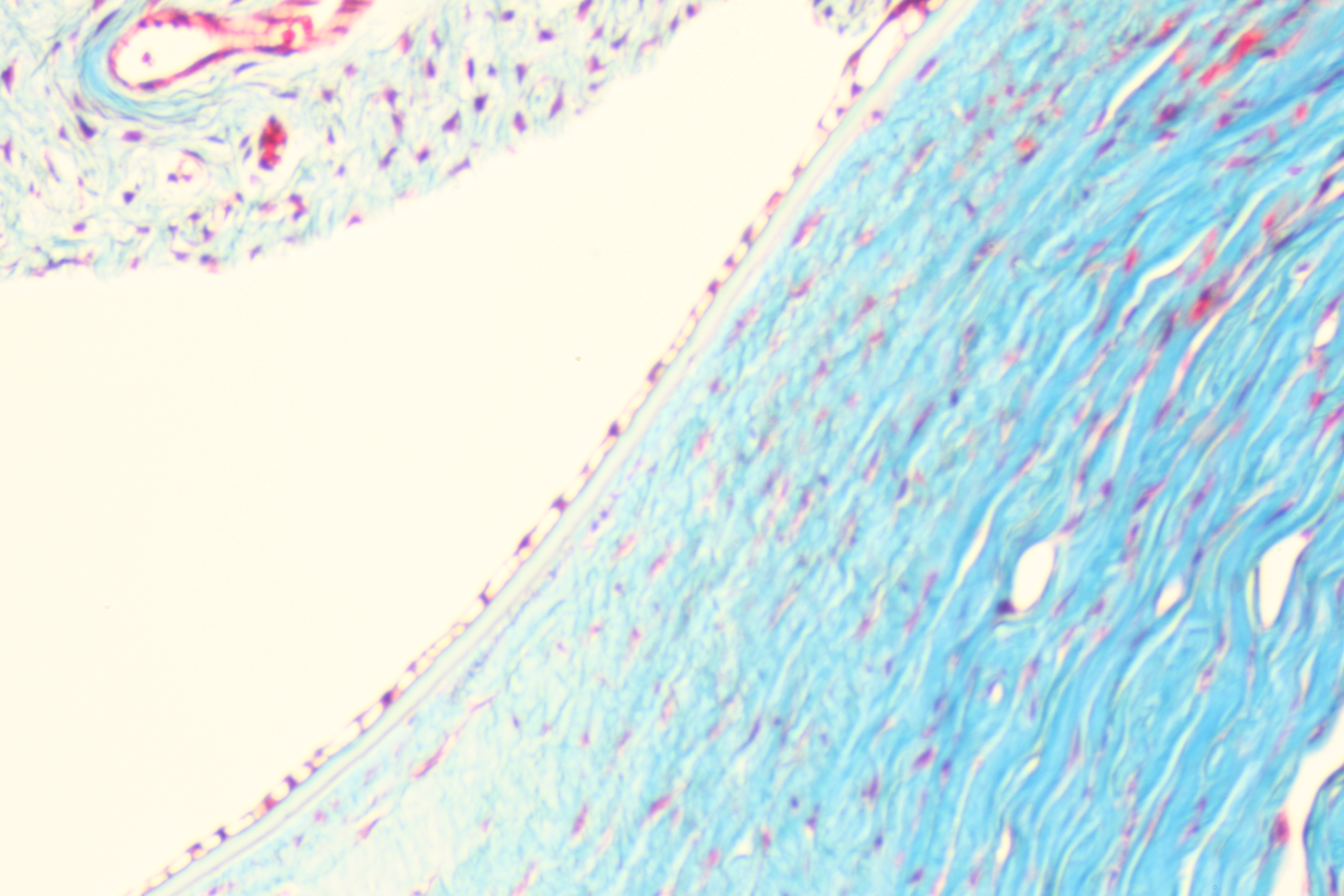

Supplement: S1 Photoset — (ZIP) [file pone.0138054.s002.zip › Multi Tx for Paper - BSS pics/IMG_6118.JPG]

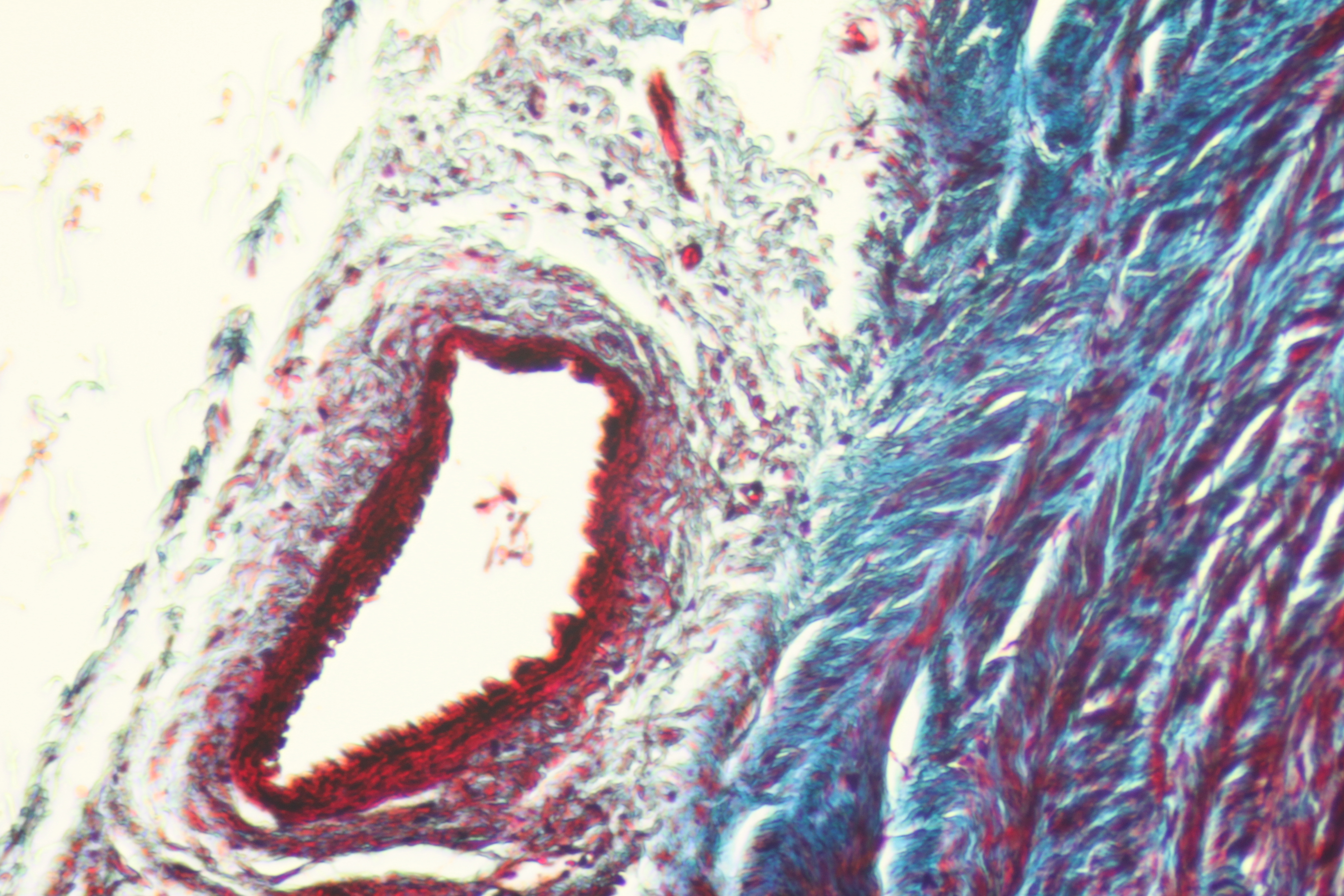

Supplement: S1 Photoset — (ZIP) [file pone.0138054.s002.zip › Multi Tx for Paper - BSS pics/IMG_6207.JPG]

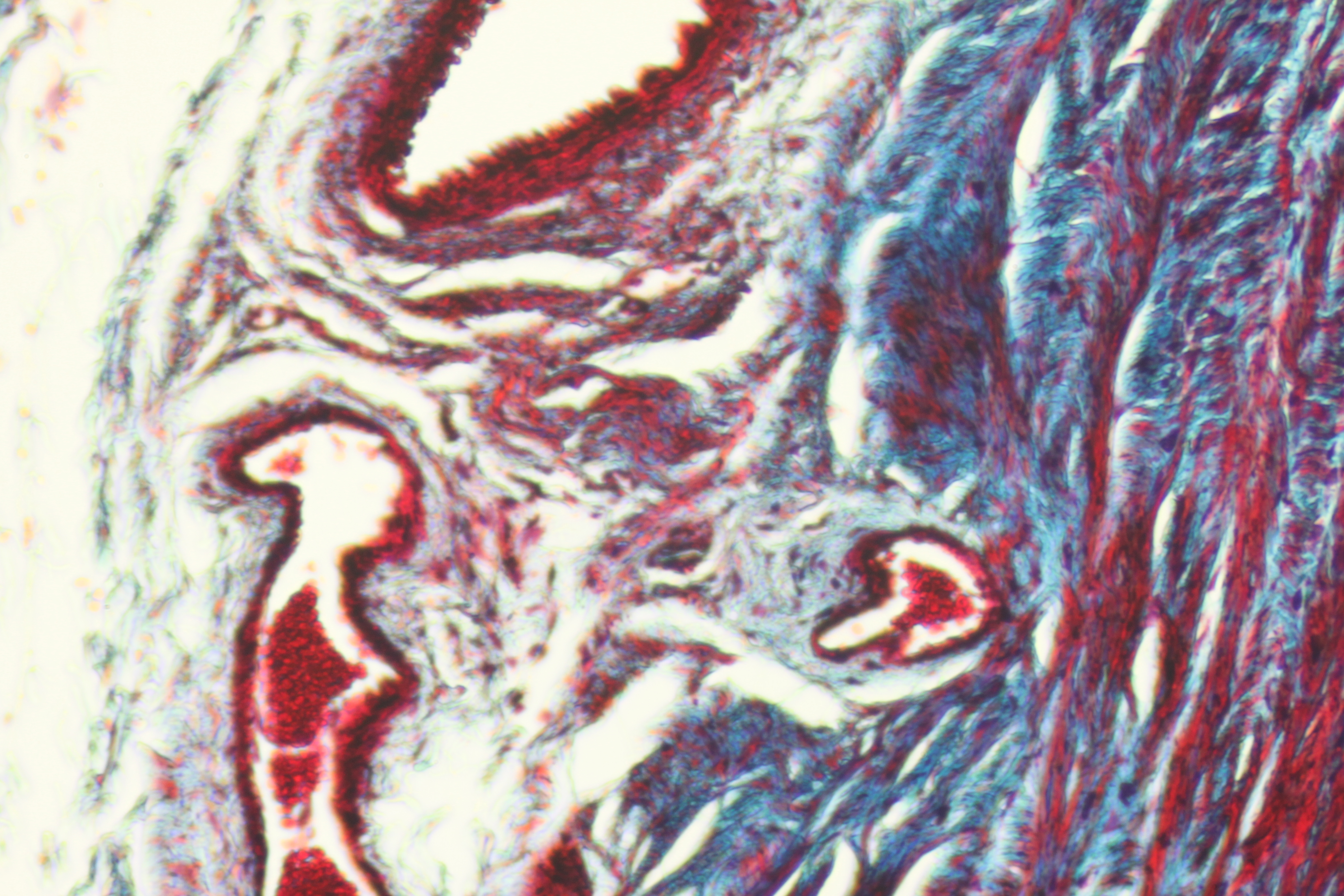

Supplement: S1 Photoset — (ZIP) [file pone.0138054.s002.zip › Multi Tx for Paper - BSS pics/IMG_6208.JPG]

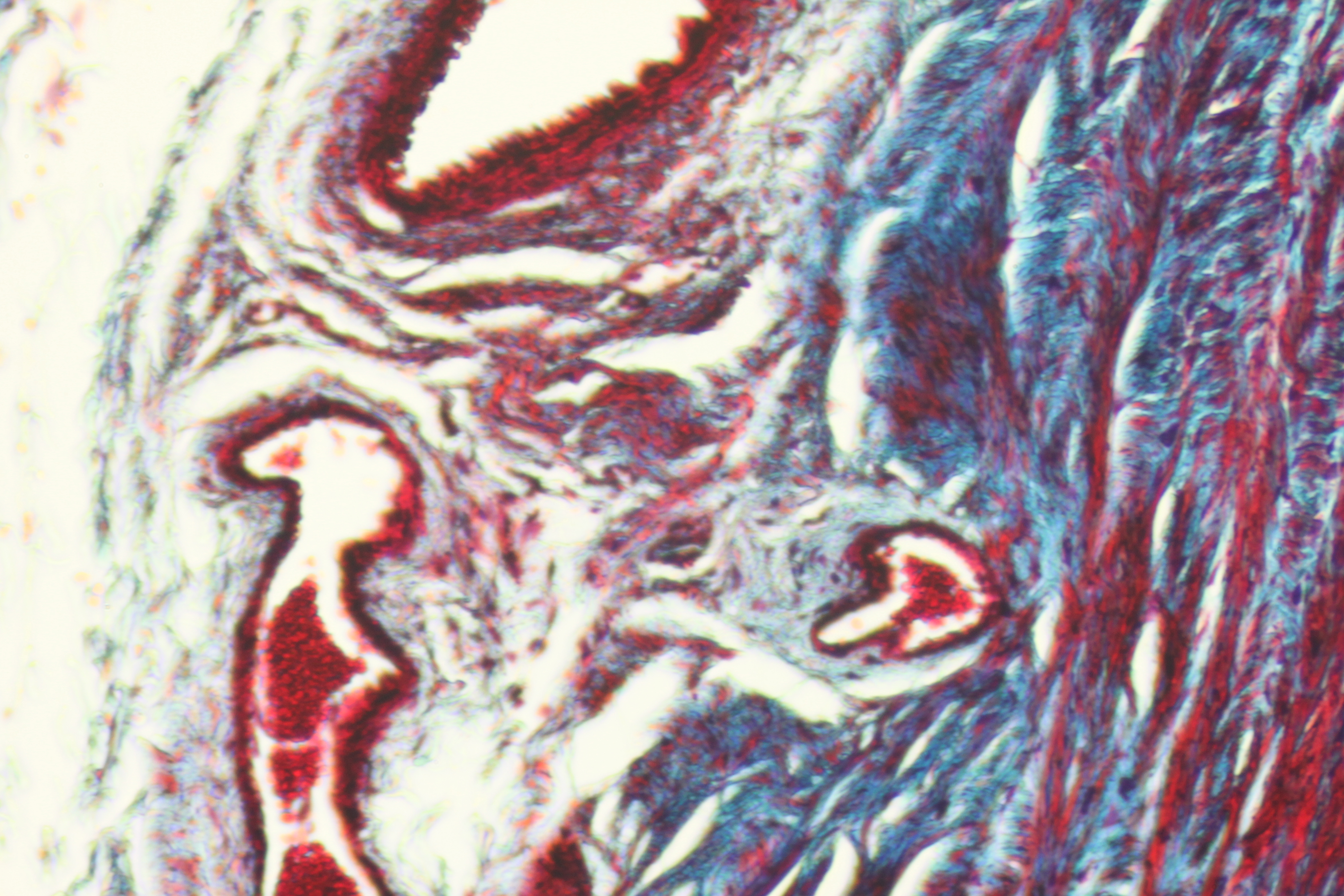

Supplement: S1 Photoset — (ZIP) [file pone.0138054.s002.zip › Multi Tx for Paper - BSS pics/IMG_6209.JPG]

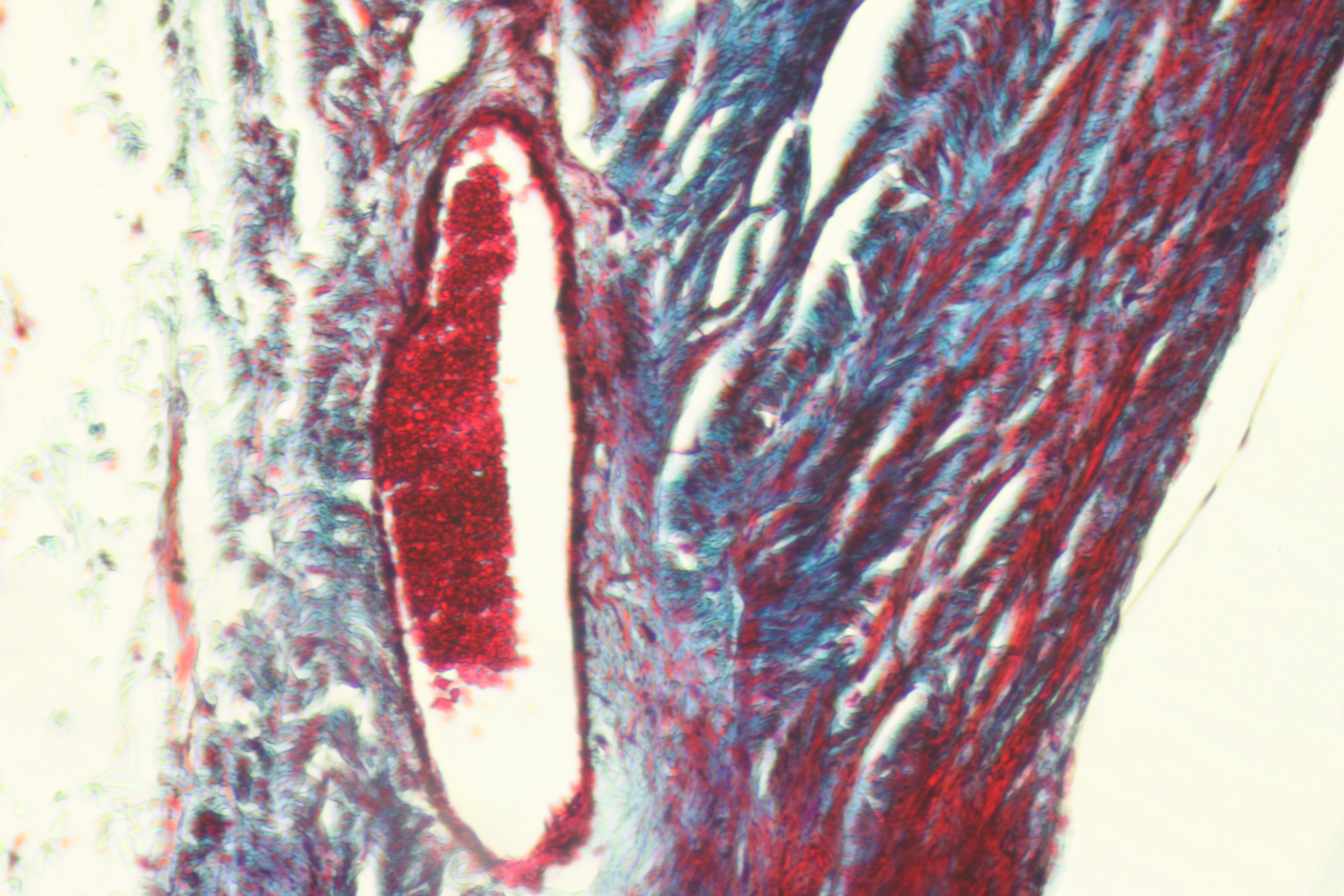

Supplement: S1 Photoset — (ZIP) [file pone.0138054.s002.zip › Multi Tx for Paper - BSS pics/IMG_6211.JPG]

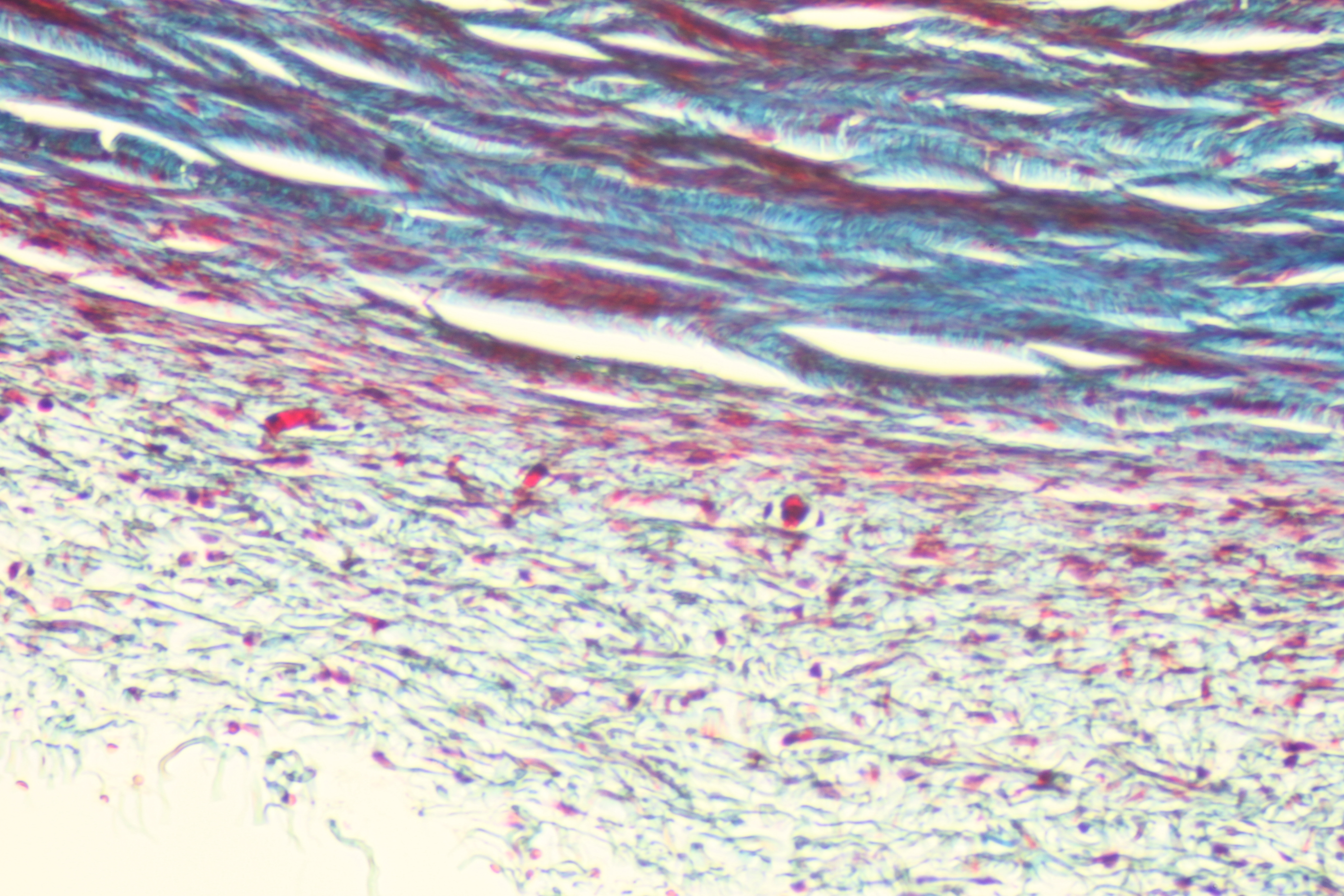

Supplement: S1 Photoset — (ZIP) [file pone.0138054.s002.zip › Multi Tx for Paper - BSS pics/IMG_6213.JPG]

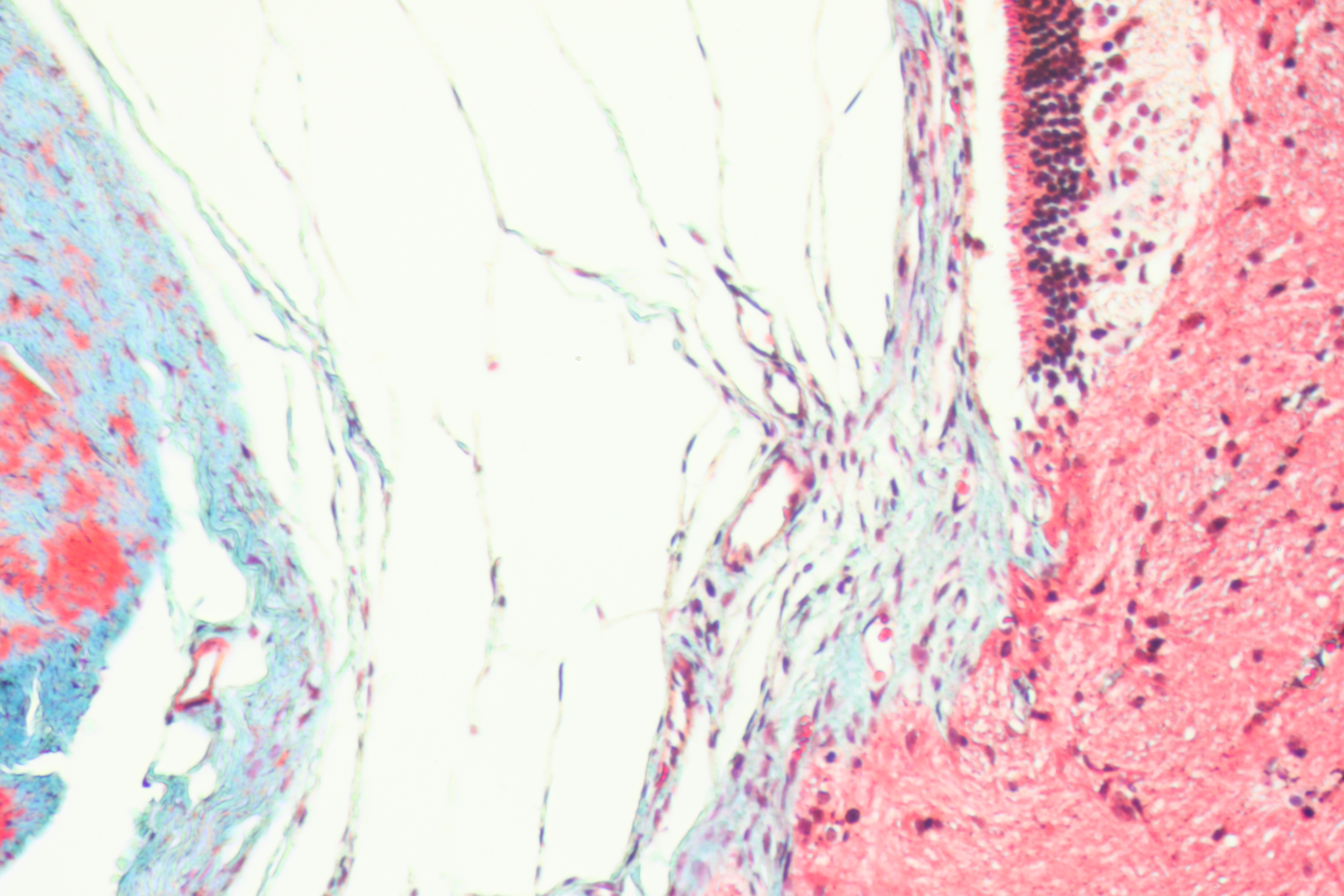

Supplement: S1 Photoset — (ZIP) [file pone.0138054.s002.zip › Multi Tx for Paper - BSS pics/IMG_6216.JPG]

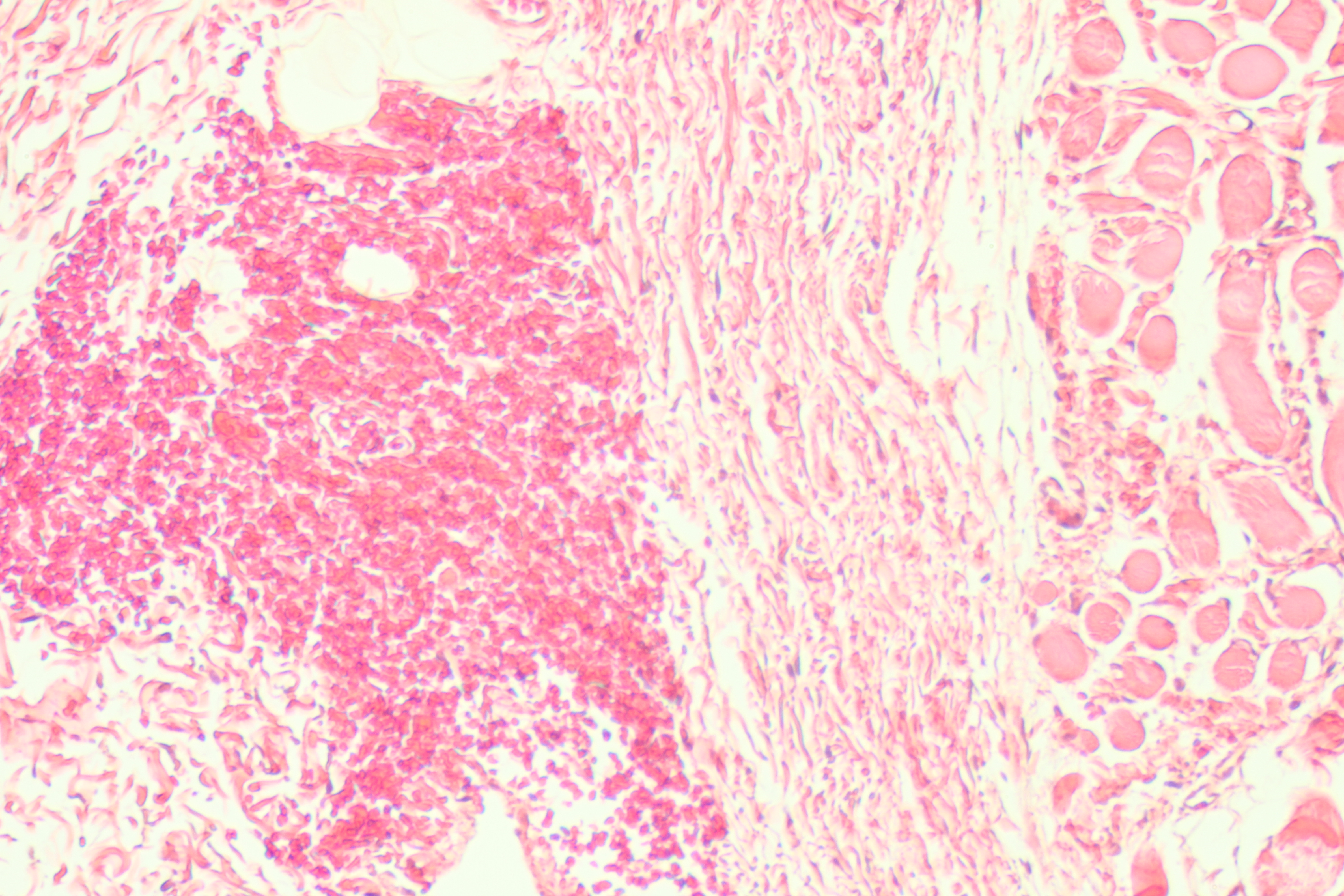

Supplement: S1 Photoset — (ZIP) [file pone.0138054.s002.zip › Multi Tx for Paper - BSS pics/IMG_6234.JPG]

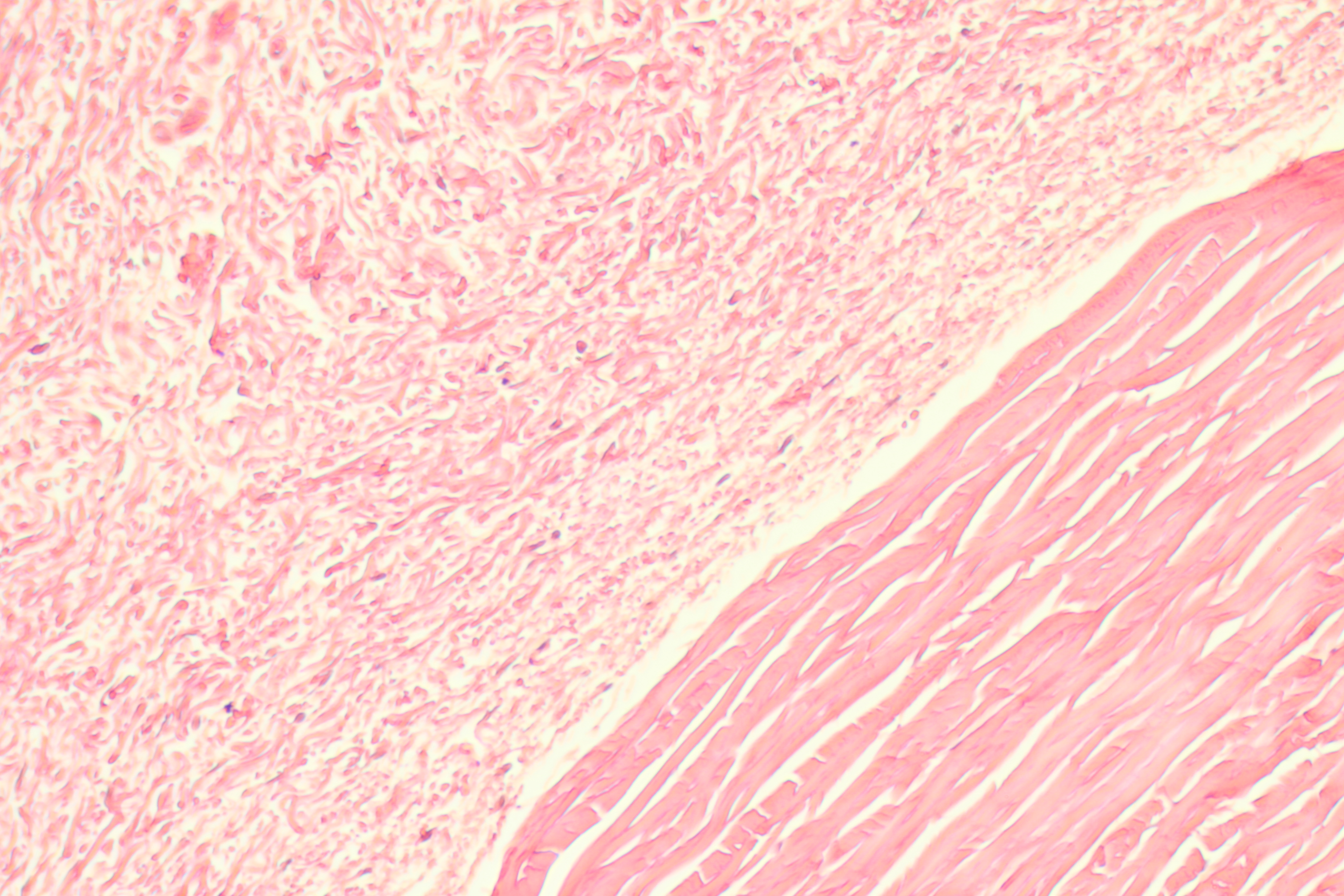

Supplement: S1 Photoset — (ZIP) [file pone.0138054.s002.zip › Multi Tx for Paper - BSS pics/IMG_6238.JPG]

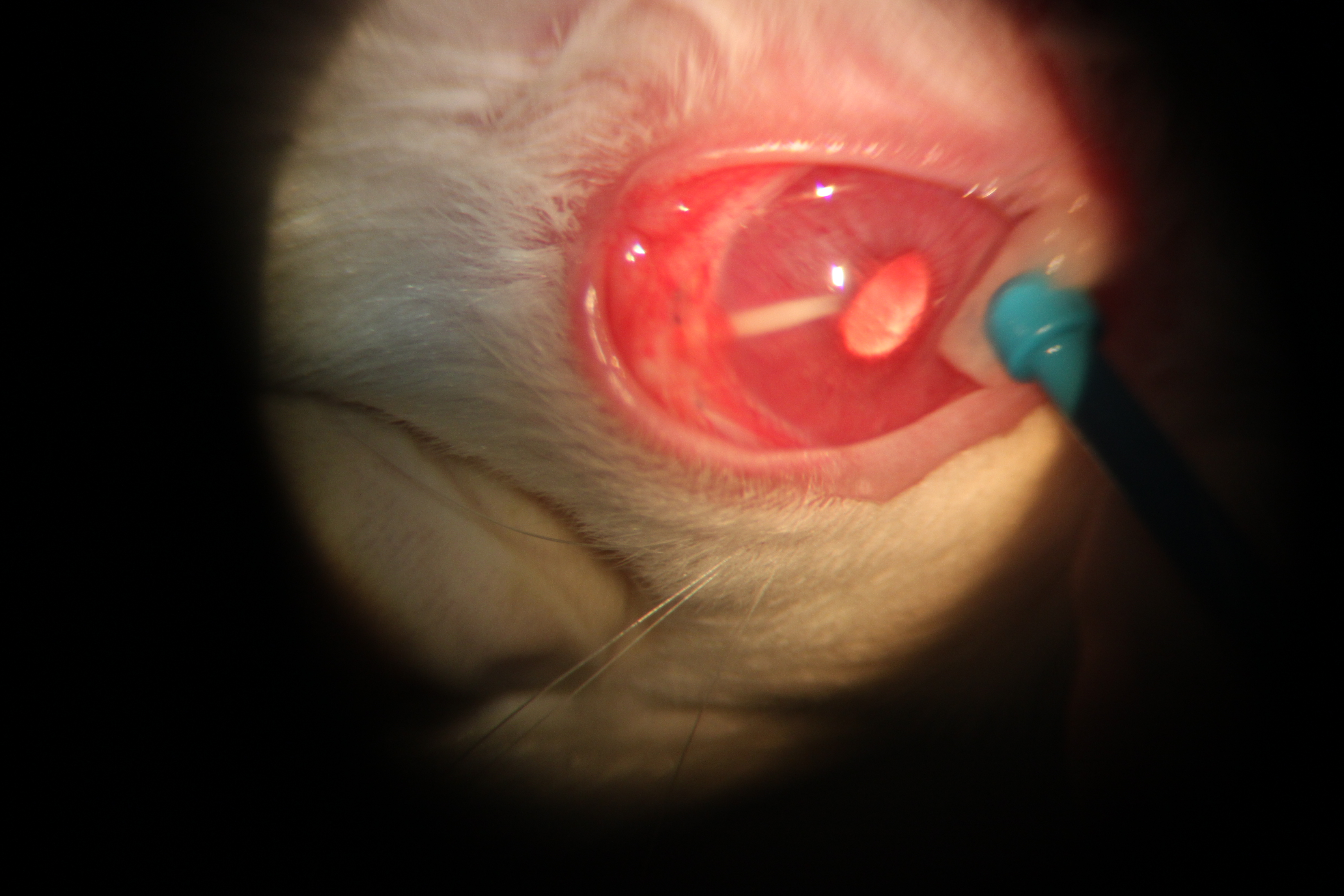

Supplement: S2 Photoset — (ZIP) [file pone.0138054.s003.zip › Multi Tx for Paper - MMC pics 1/IMG_1348.JPG]

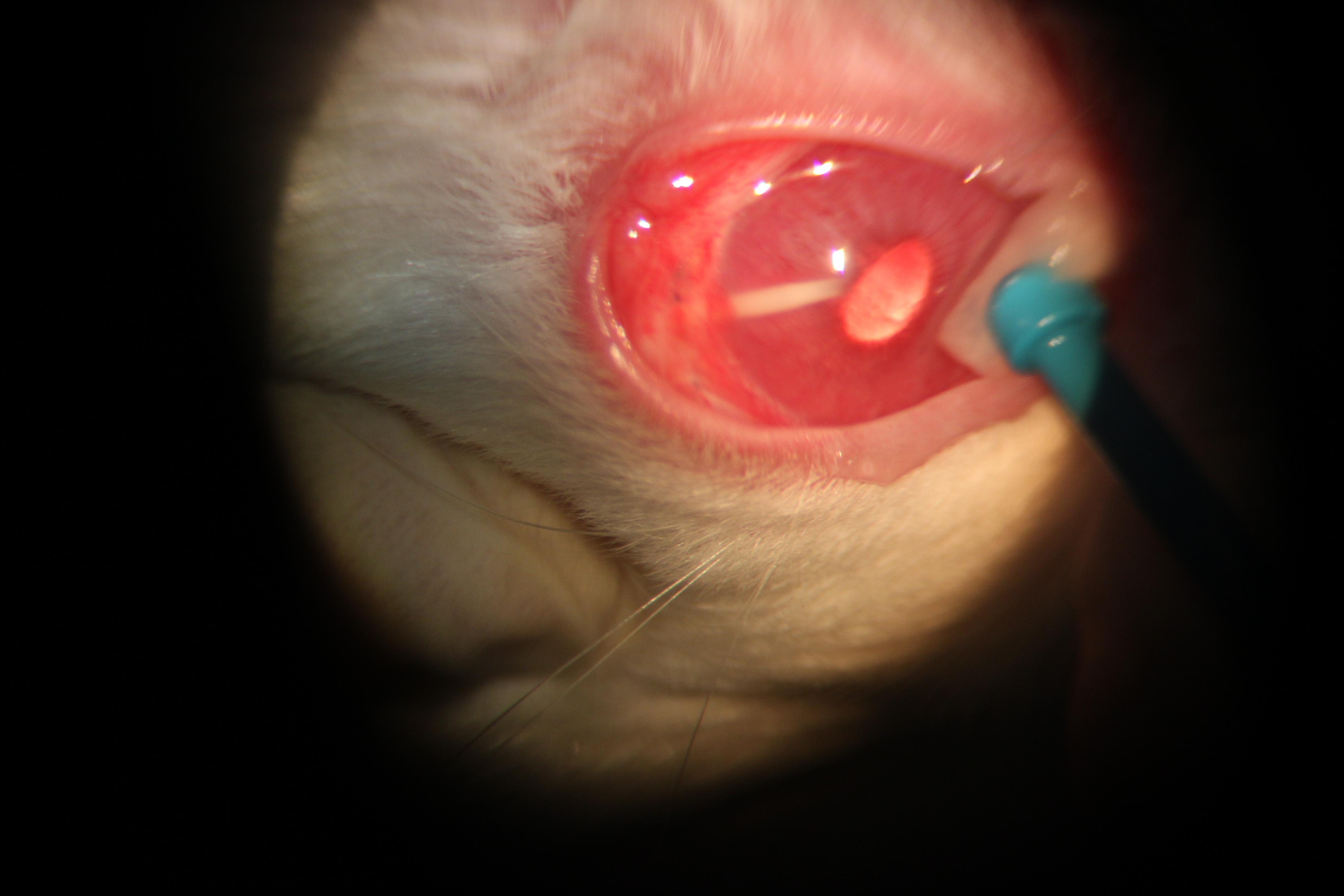

Supplement: S2 Photoset — (ZIP) [file pone.0138054.s003.zip › Multi Tx for Paper - MMC pics 1/IMG_1349.JPG]

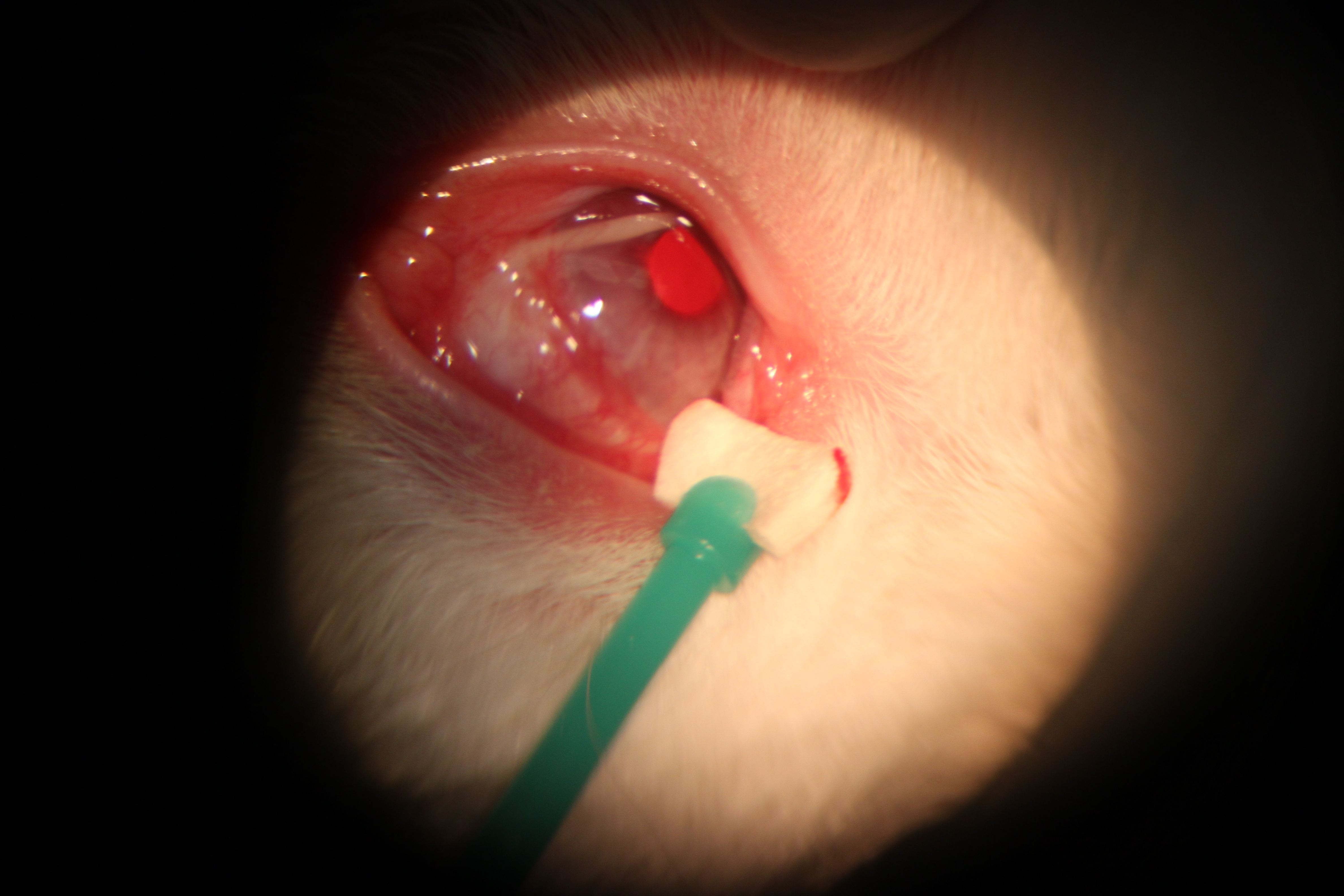

Supplement: S2 Photoset — (ZIP) [file pone.0138054.s003.zip › Multi Tx for Paper - MMC pics 1/IMG_1984.JPG]

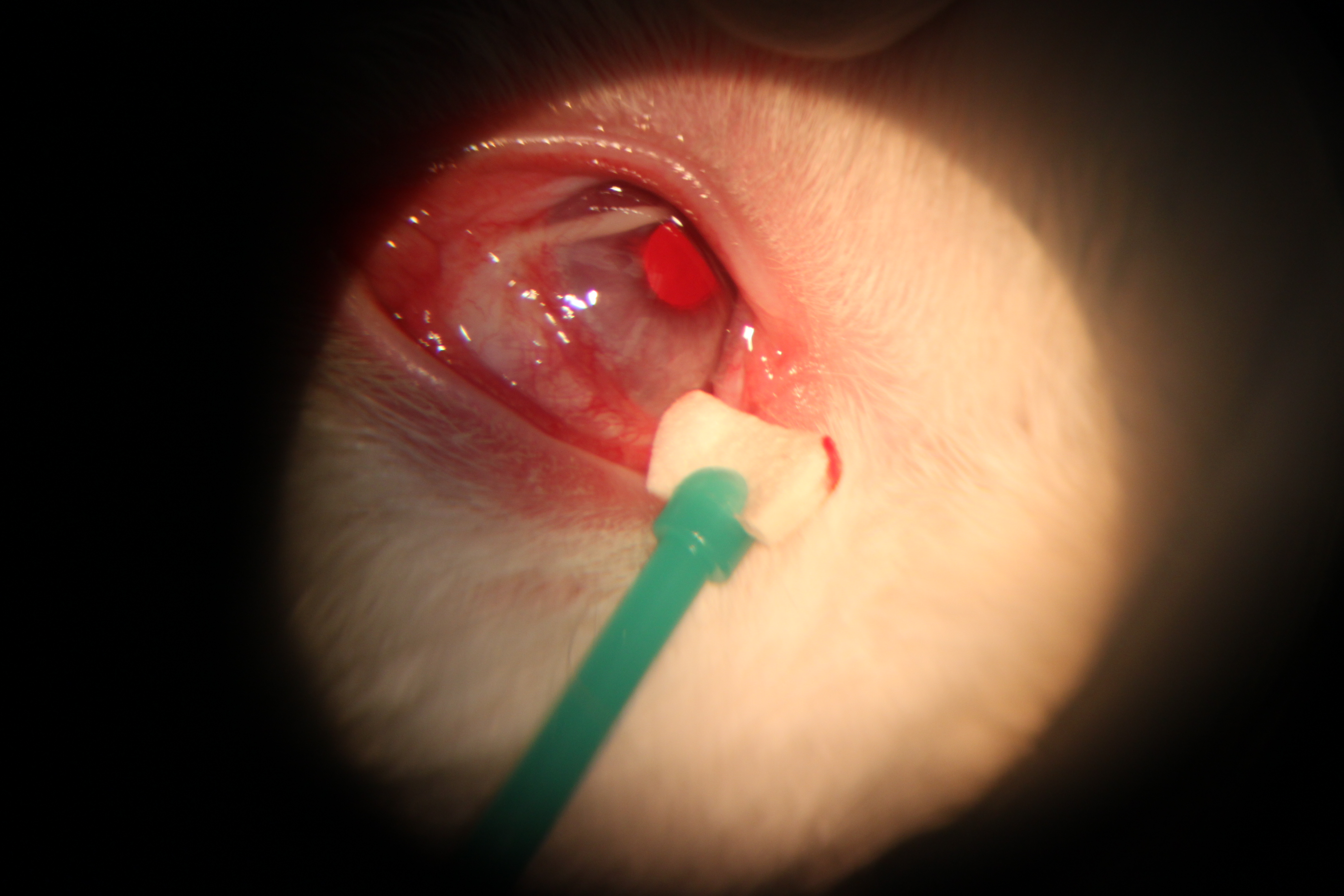

Supplement: S2 Photoset — (ZIP) [file pone.0138054.s003.zip › Multi Tx for Paper - MMC pics 1/IMG_1986.JPG]

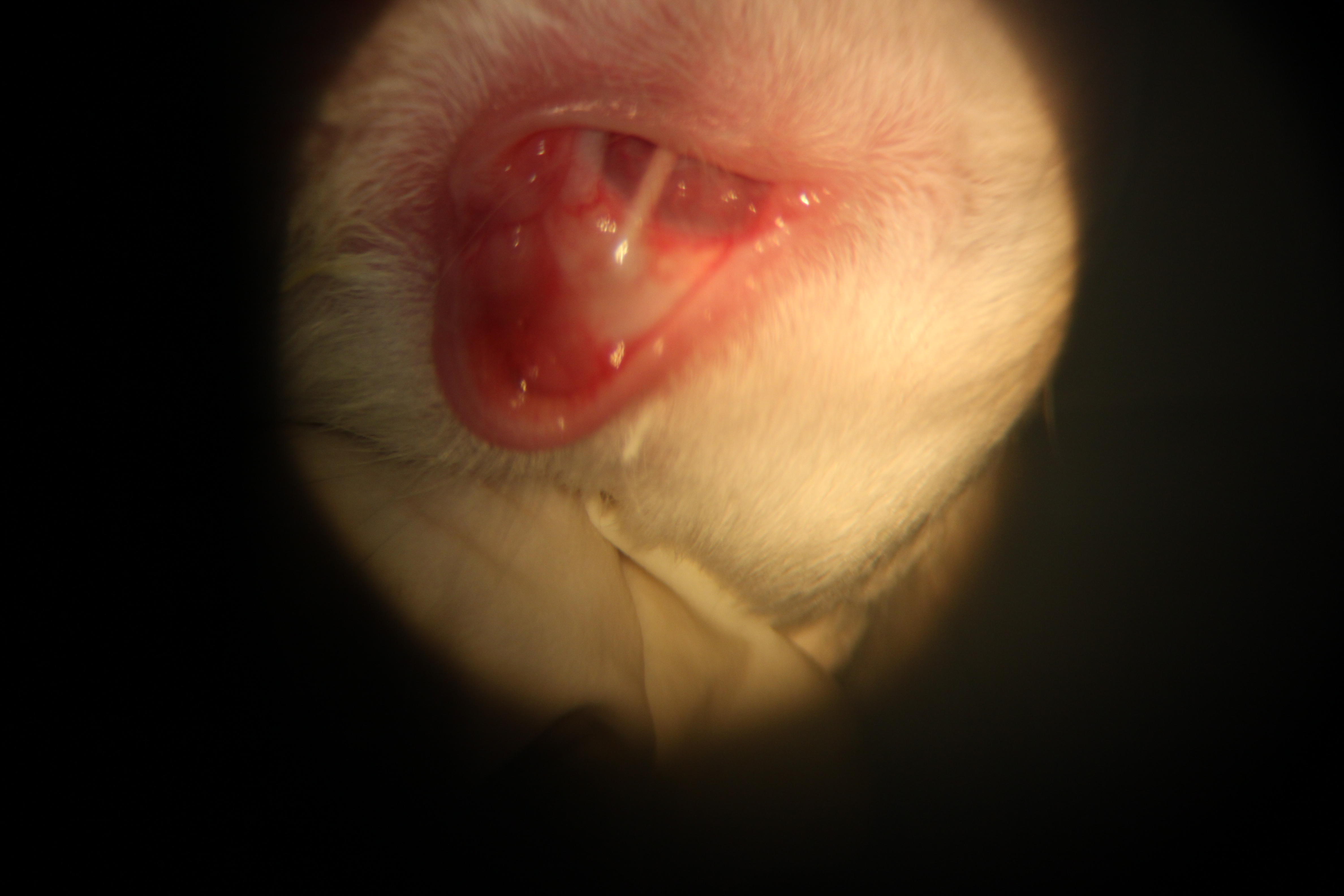

Supplement: S2 Photoset — (ZIP) [file pone.0138054.s003.zip › Multi Tx for Paper - MMC pics 1/IMG_2357.JPG]

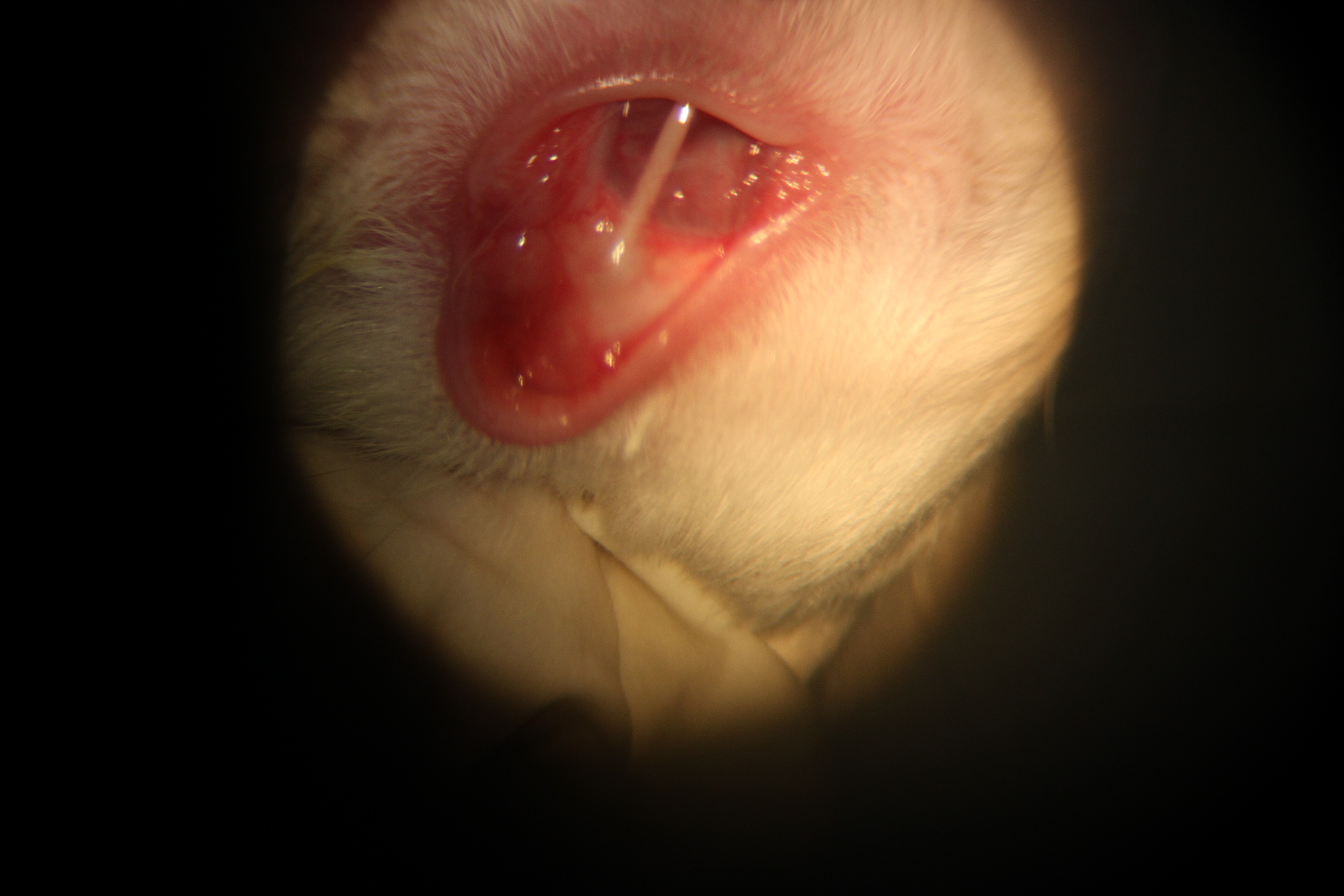

Supplement: S2 Photoset — (ZIP) [file pone.0138054.s003.zip › Multi Tx for Paper - MMC pics 1/IMG_2360.JPG]

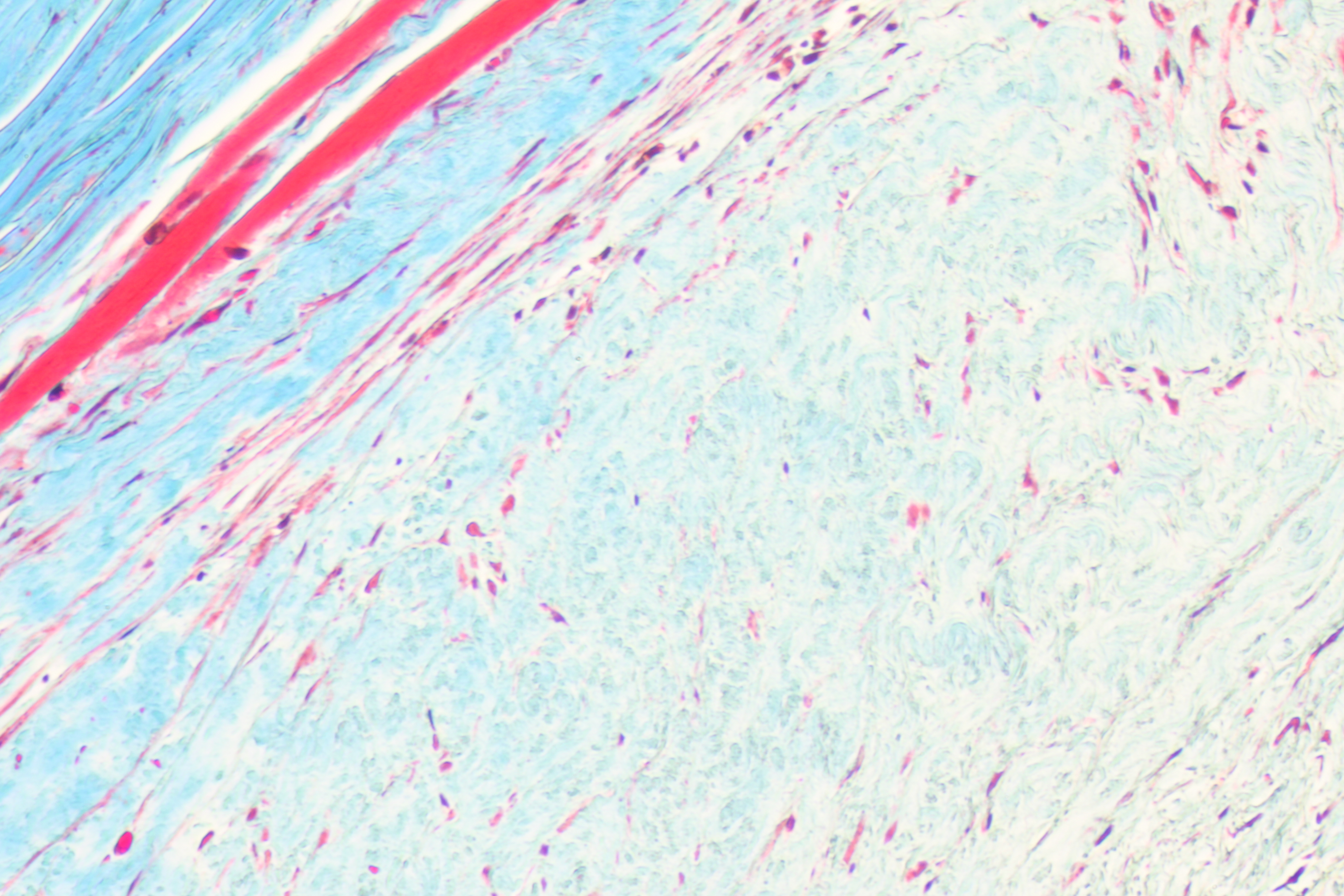

Supplement: S2 Photoset — (ZIP) [file pone.0138054.s003.zip › Multi Tx for Paper - MMC pics 1/IMG_6108.JPG]

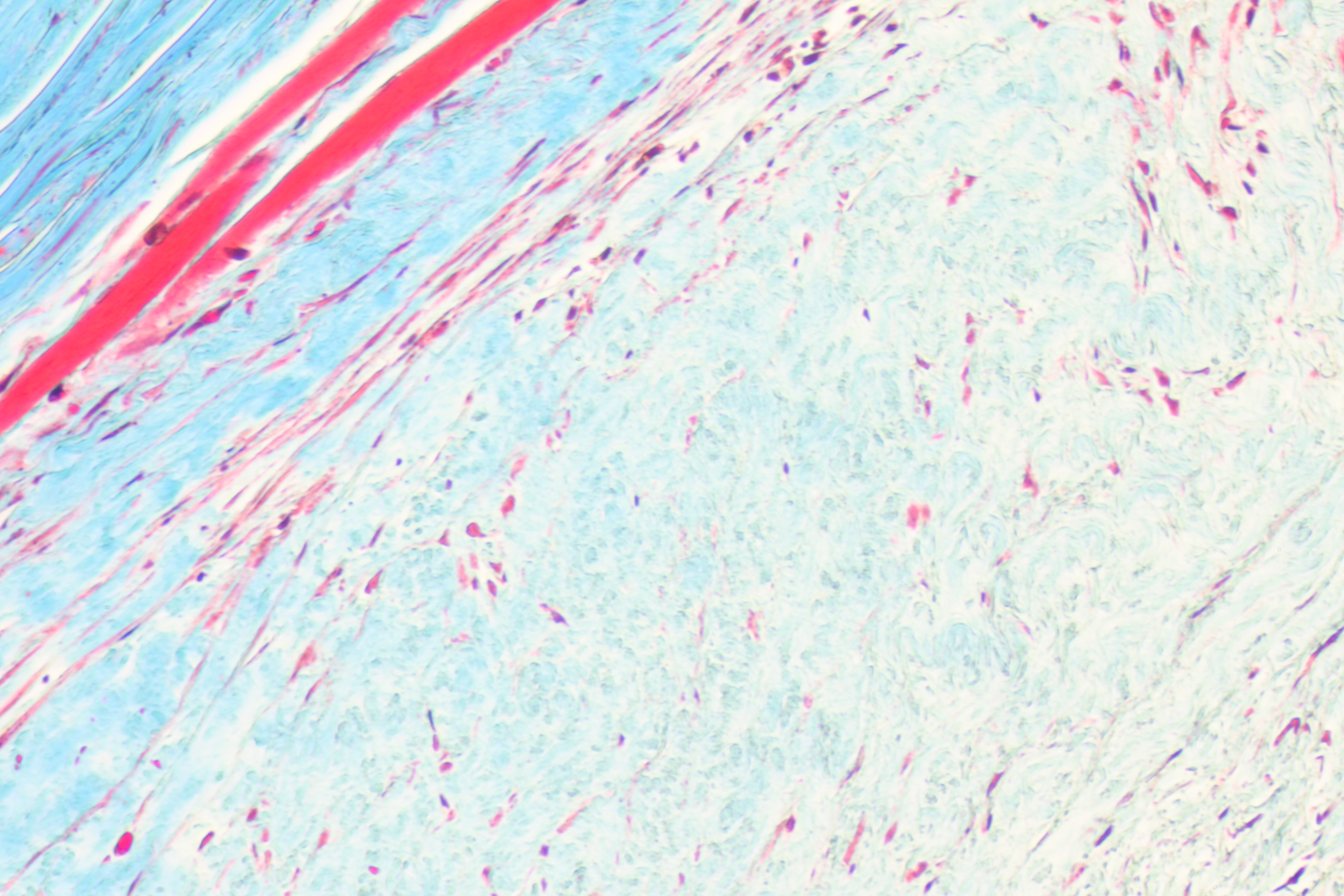

Supplement: S2 Photoset — (ZIP) [file pone.0138054.s003.zip › Multi Tx for Paper - MMC pics 1/IMG_6109.JPG]

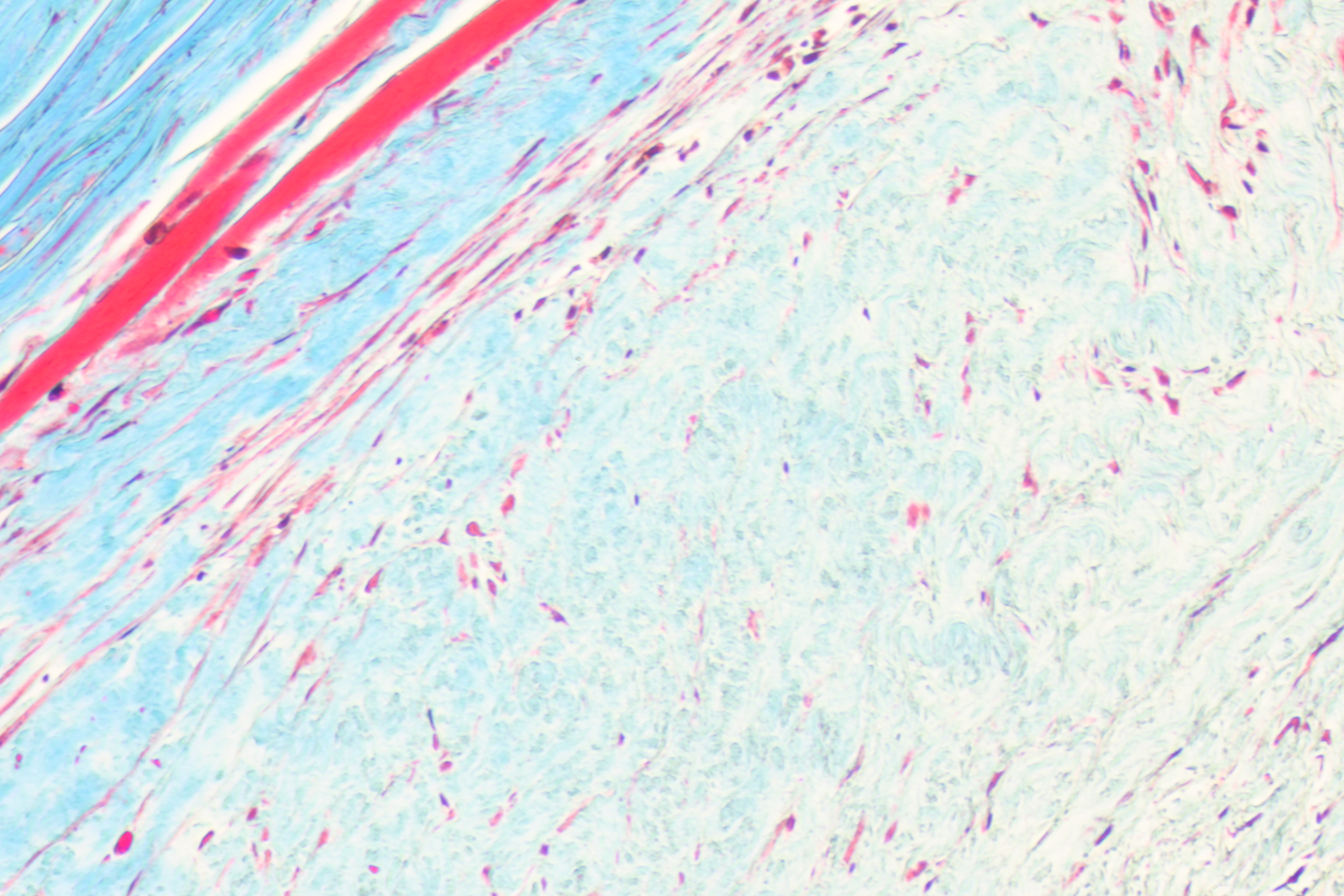

Supplement: S2 Photoset — (ZIP) [file pone.0138054.s003.zip › Multi Tx for Paper - MMC pics 1/IMG_6110.JPG]

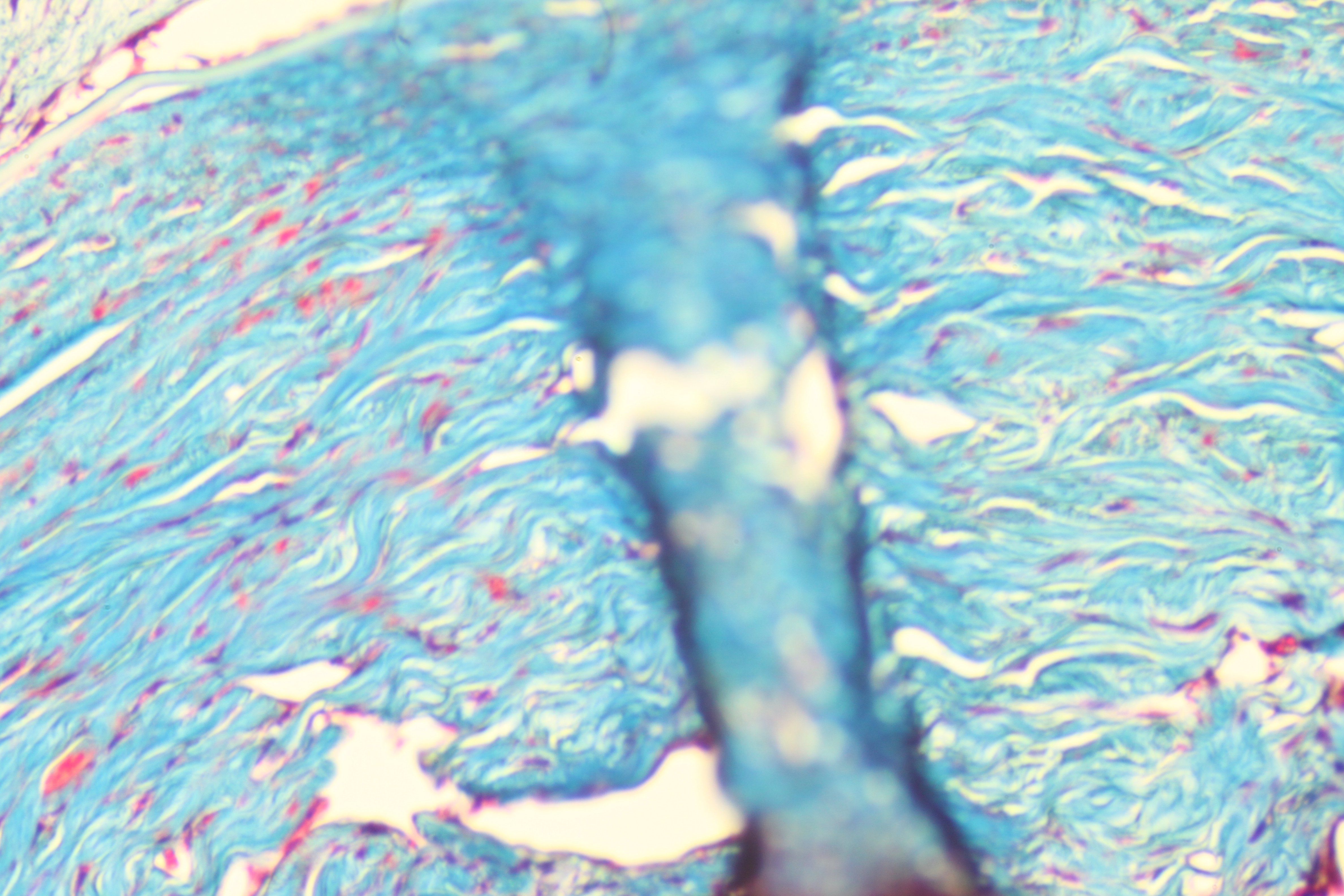

Supplement: S2 Photoset — (ZIP) [file pone.0138054.s003.zip › Multi Tx for Paper - MMC pics 1/IMG_6111.JPG]

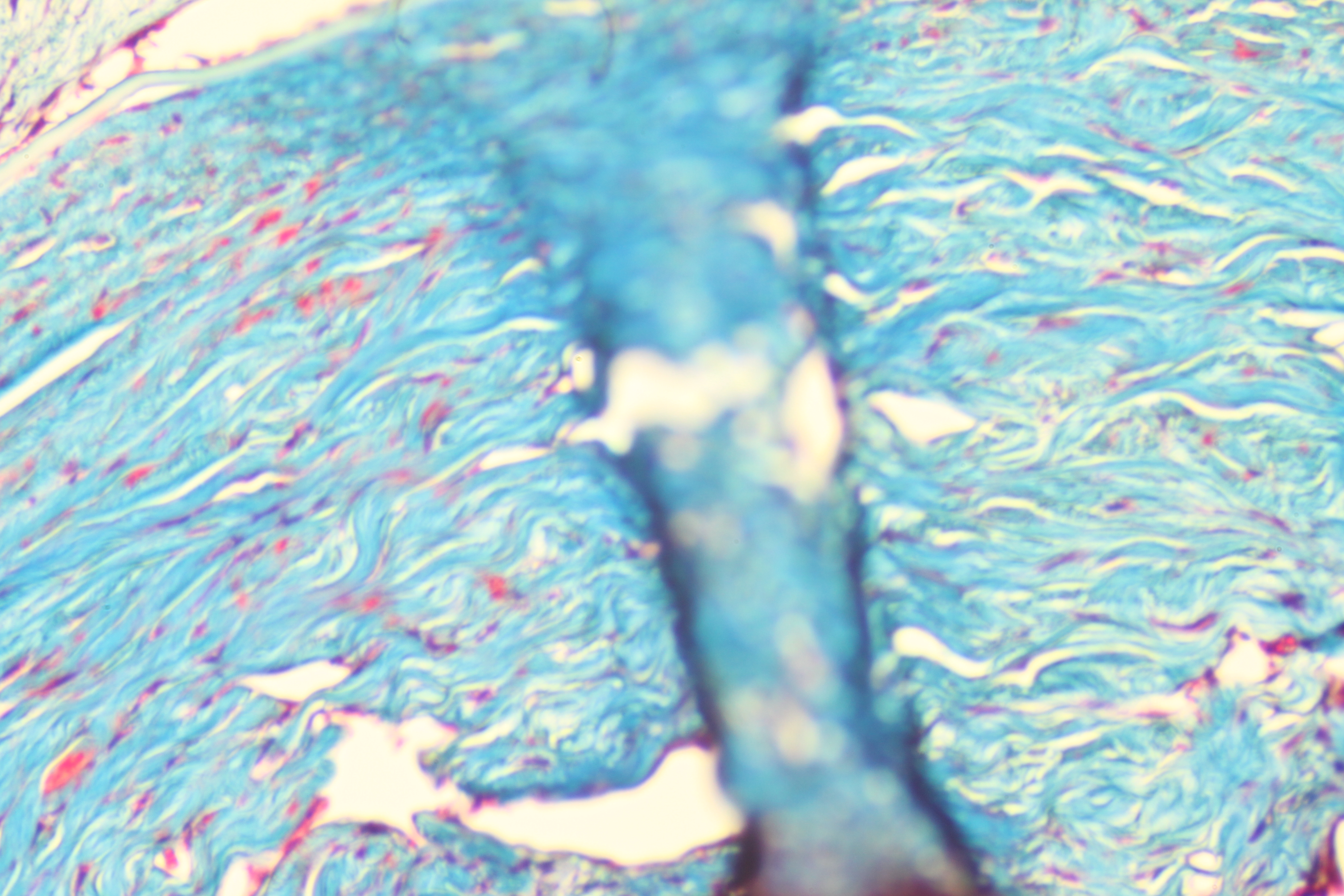

Supplement: S2 Photoset — (ZIP) [file pone.0138054.s003.zip › Multi Tx for Paper - MMC pics 1/IMG_6112.JPG]

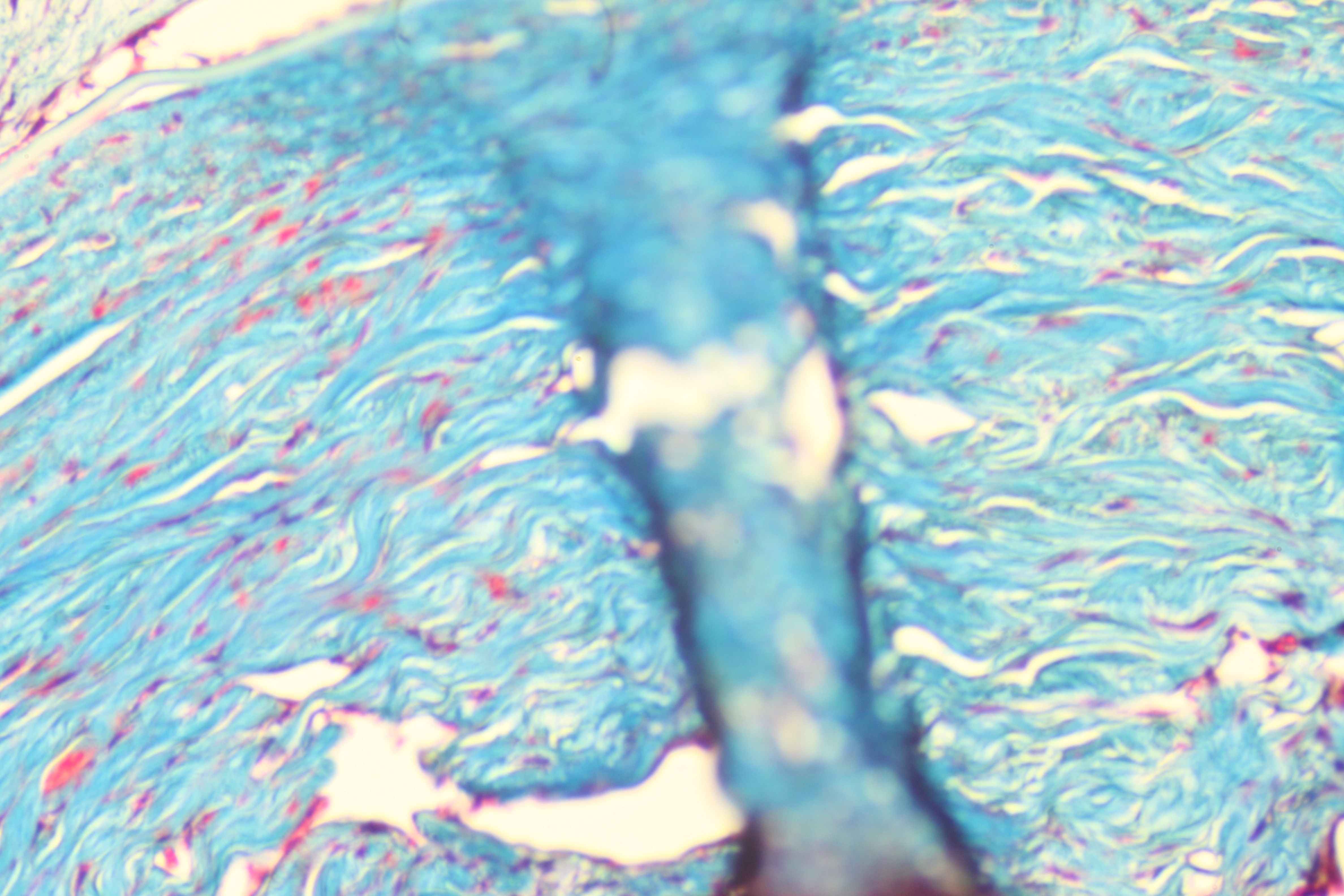

Supplement: S2 Photoset — (ZIP) [file pone.0138054.s003.zip › Multi Tx for Paper - MMC pics 1/IMG_6113.JPG]

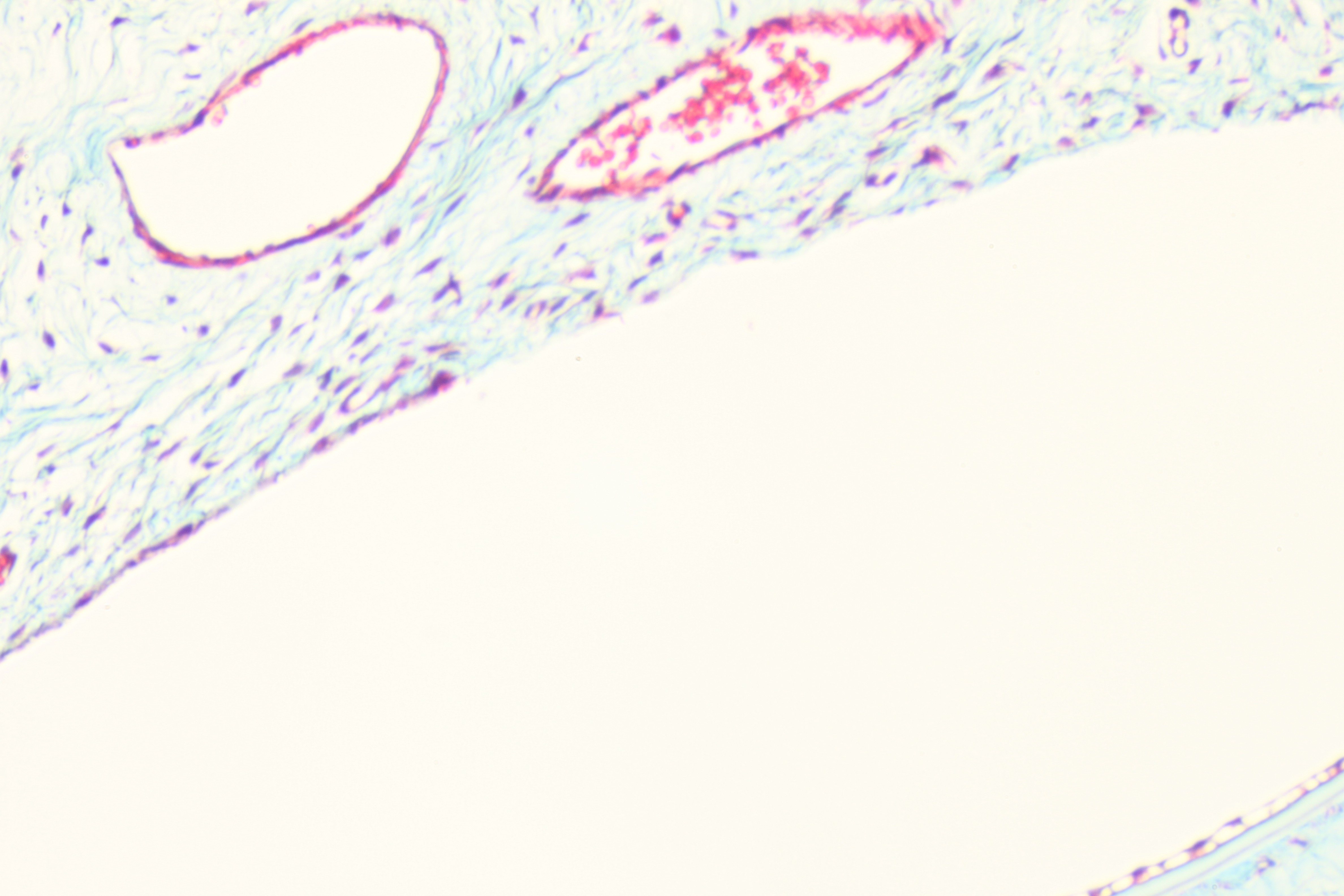

Supplement: S2 Photoset — (ZIP) [file pone.0138054.s003.zip › Multi Tx for Paper - MMC pics 1/IMG_6114.JPG]

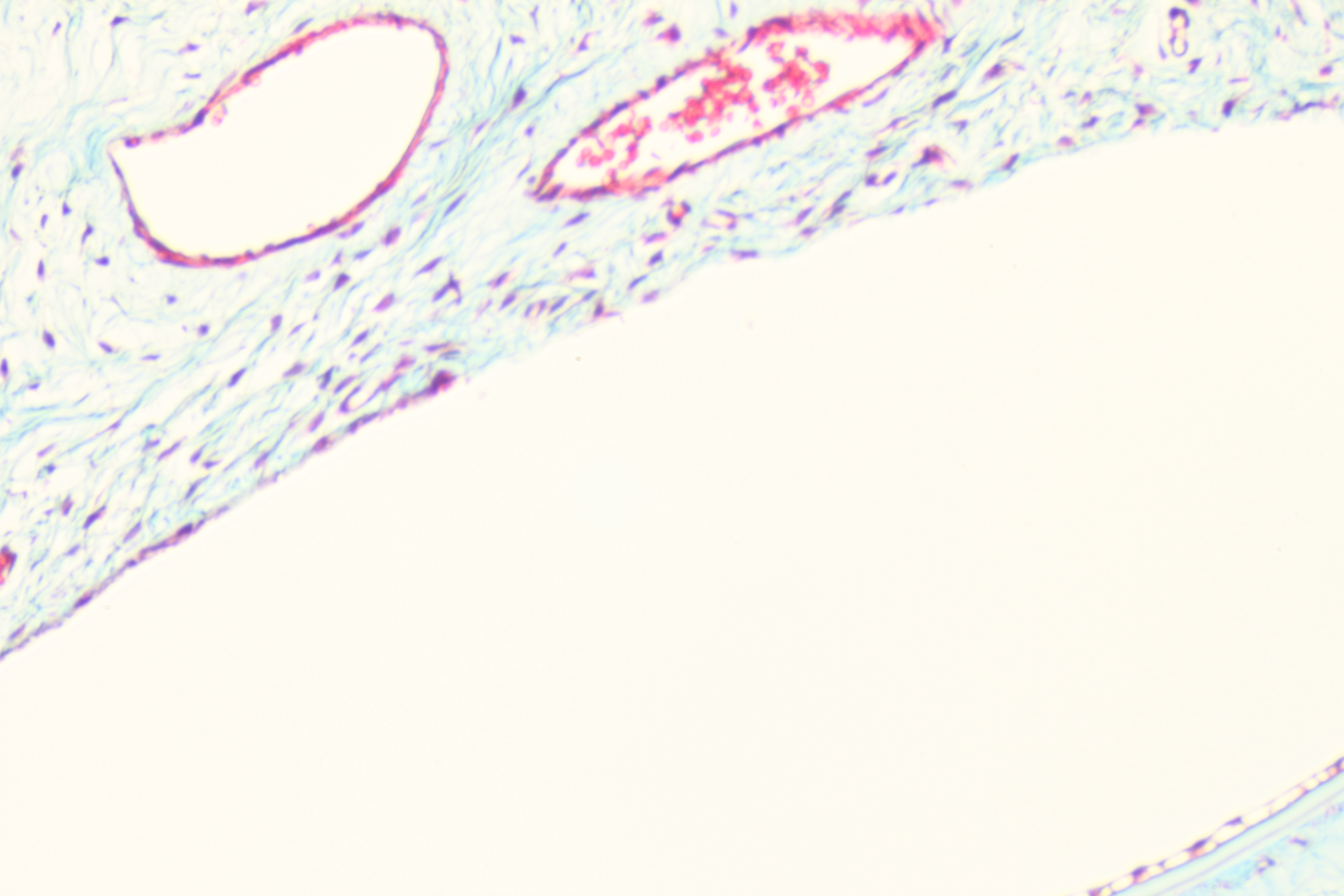

Supplement: S2 Photoset — (ZIP) [file pone.0138054.s003.zip › Multi Tx for Paper - MMC pics 1/IMG_6115.JPG]

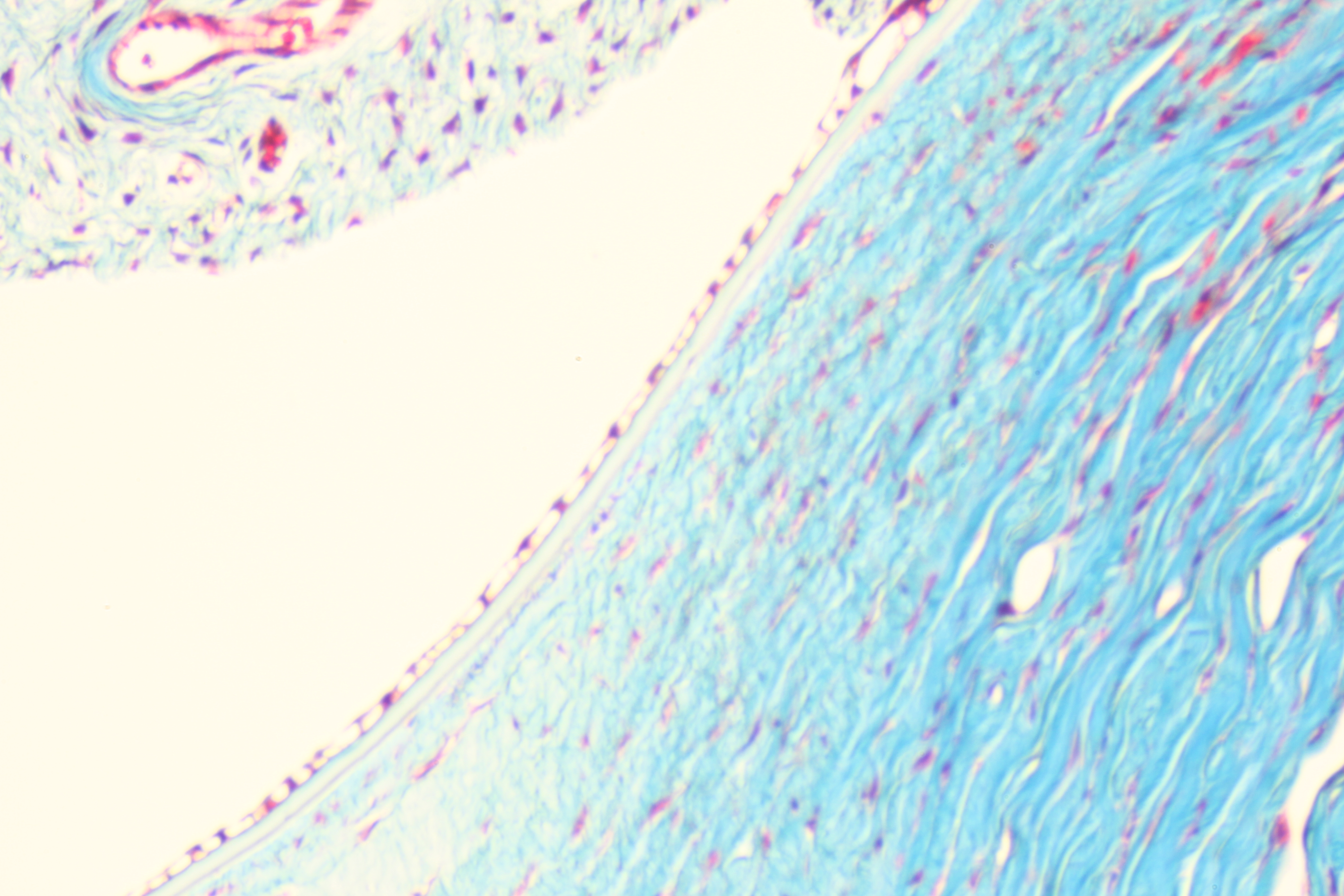

Supplement: S2 Photoset — (ZIP) [file pone.0138054.s003.zip › Multi Tx for Paper - MMC pics 1/IMG_6117.JPG]

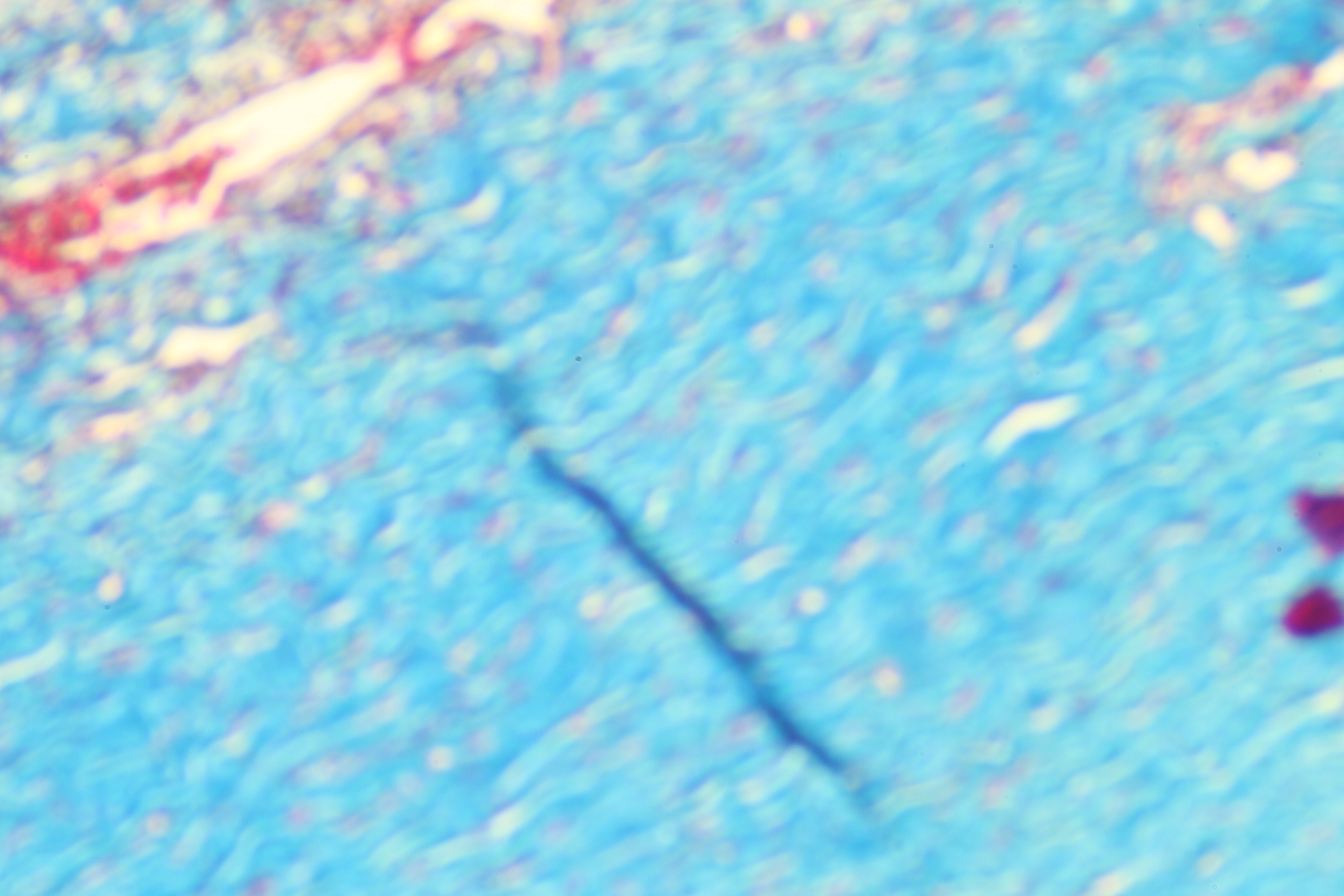

Supplement: S2 Photoset — (ZIP) [file pone.0138054.s003.zip › Multi Tx for Paper - MMC pics 1/IMG_6149.JPG]

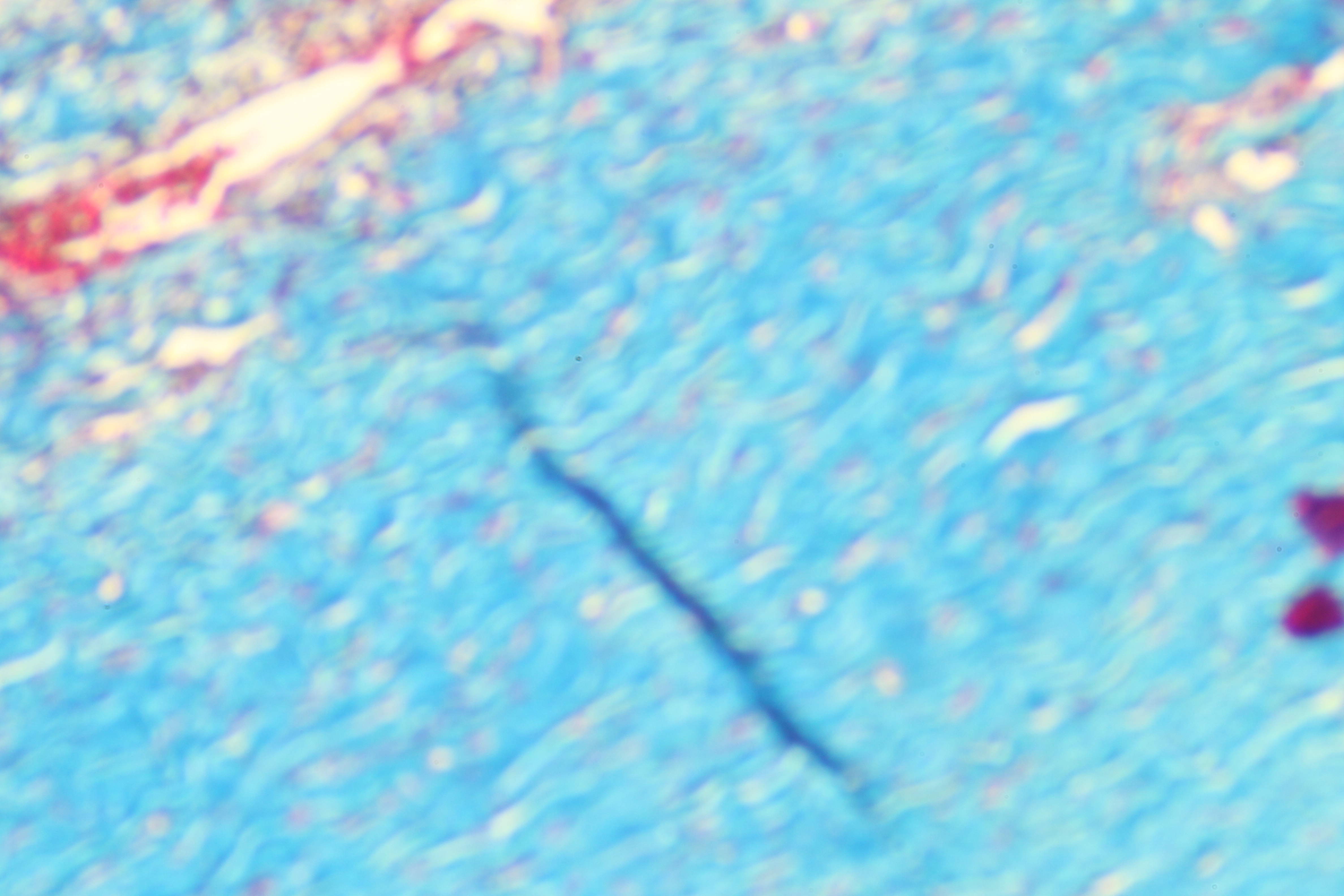

Supplement: S2 Photoset — (ZIP) [file pone.0138054.s003.zip › Multi Tx for Paper - MMC pics 1/IMG_6150.JPG]

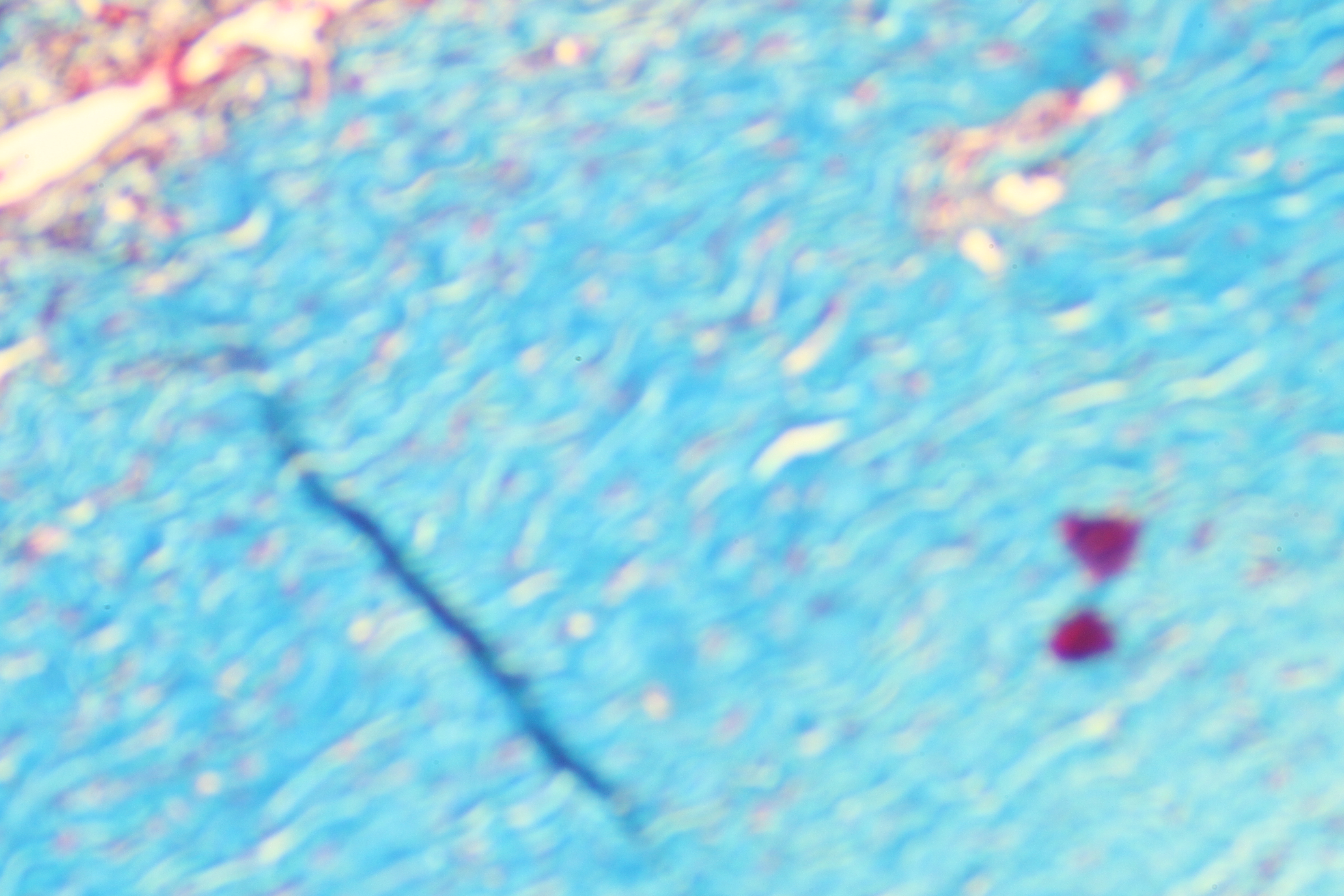

Supplement: S2 Photoset — (ZIP) [file pone.0138054.s003.zip › Multi Tx for Paper - MMC pics 1/IMG_6151.JPG]

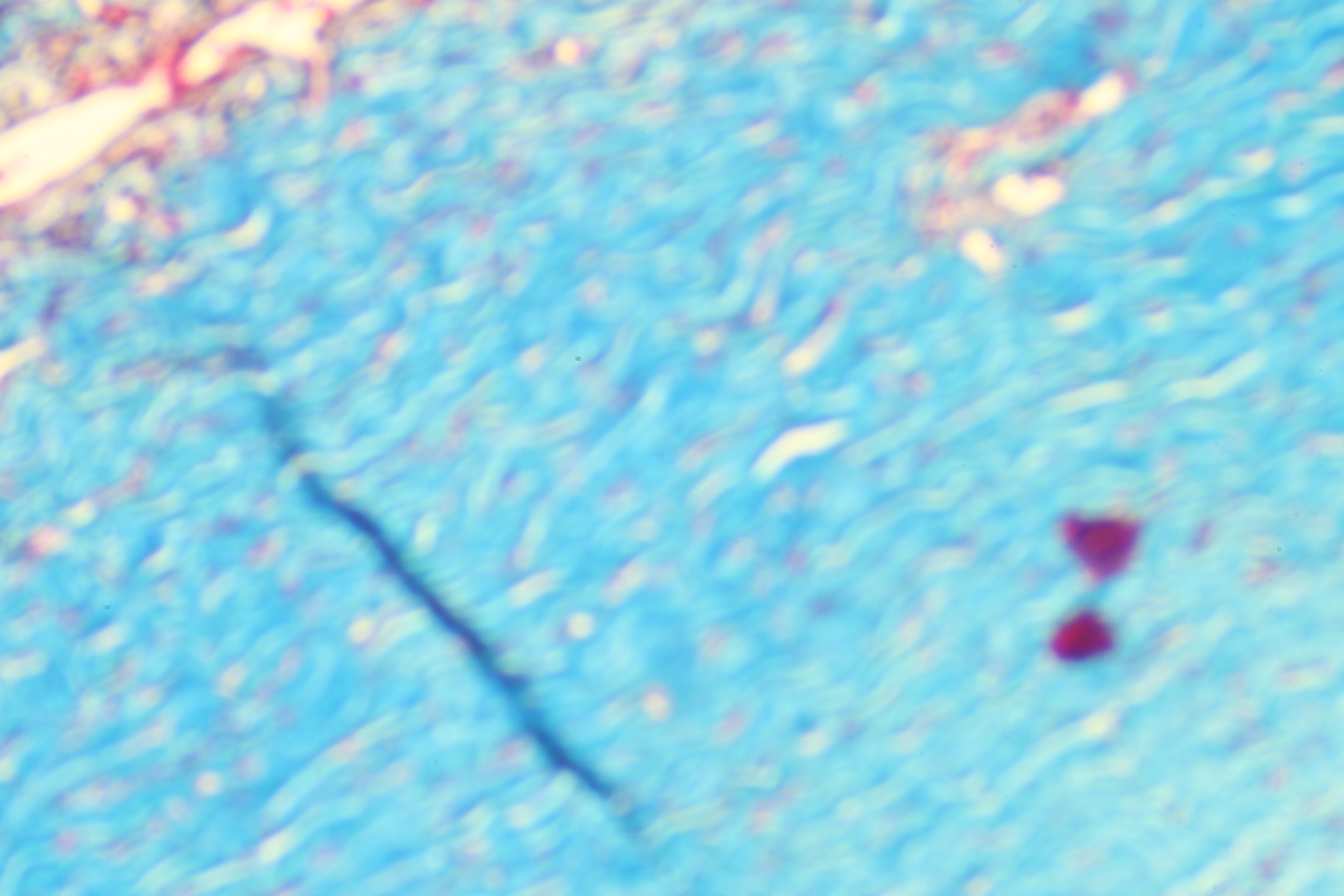

Supplement: S2 Photoset — (ZIP) [file pone.0138054.s003.zip › Multi Tx for Paper - MMC pics 1/IMG_6152.JPG]

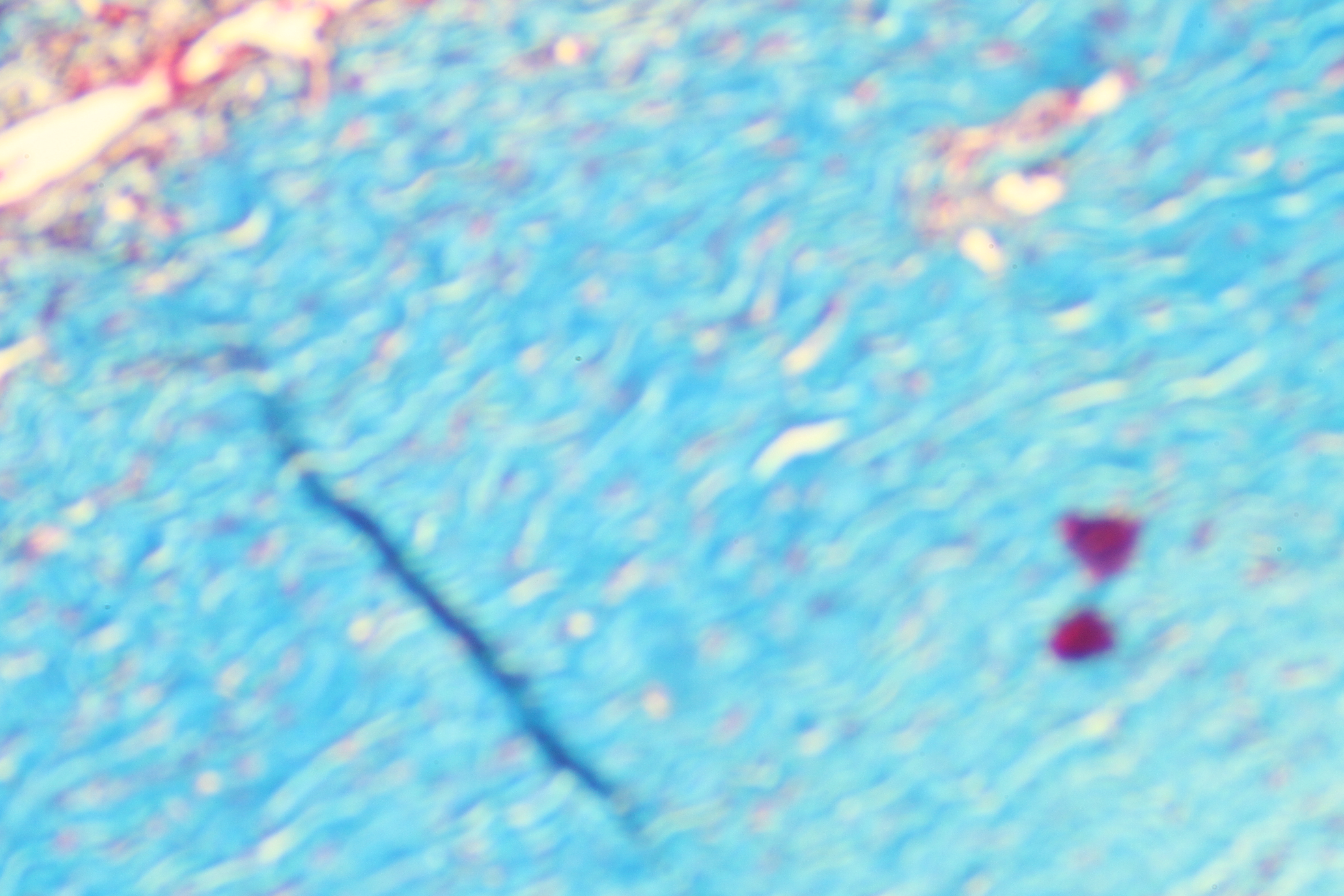

Supplement: S2 Photoset — (ZIP) [file pone.0138054.s003.zip › Multi Tx for Paper - MMC pics 1/IMG_6153.JPG]

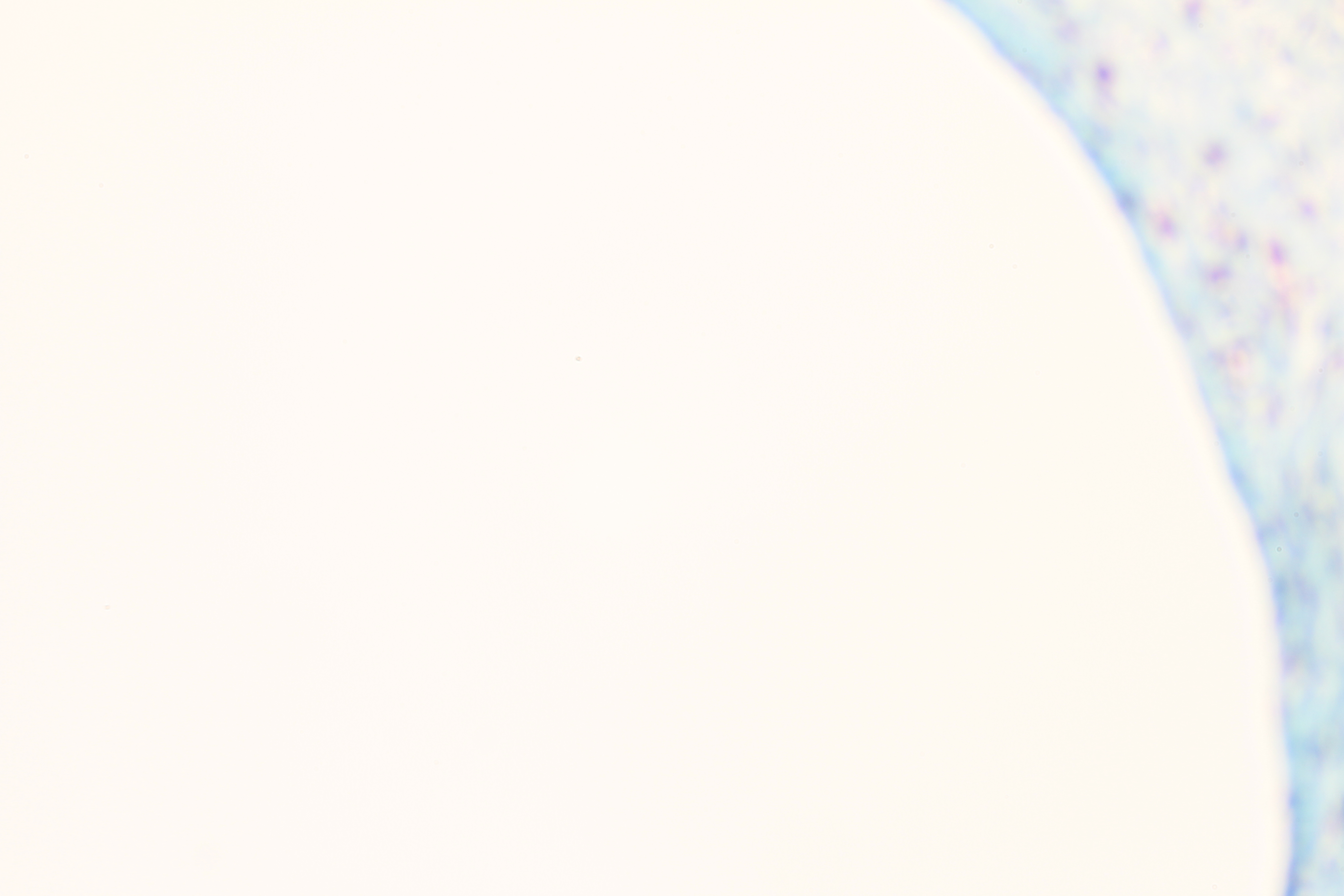

Supplement: S2 Photoset — (ZIP) [file pone.0138054.s003.zip › Multi Tx for Paper - MMC pics 1/IMG_6154.JPG]

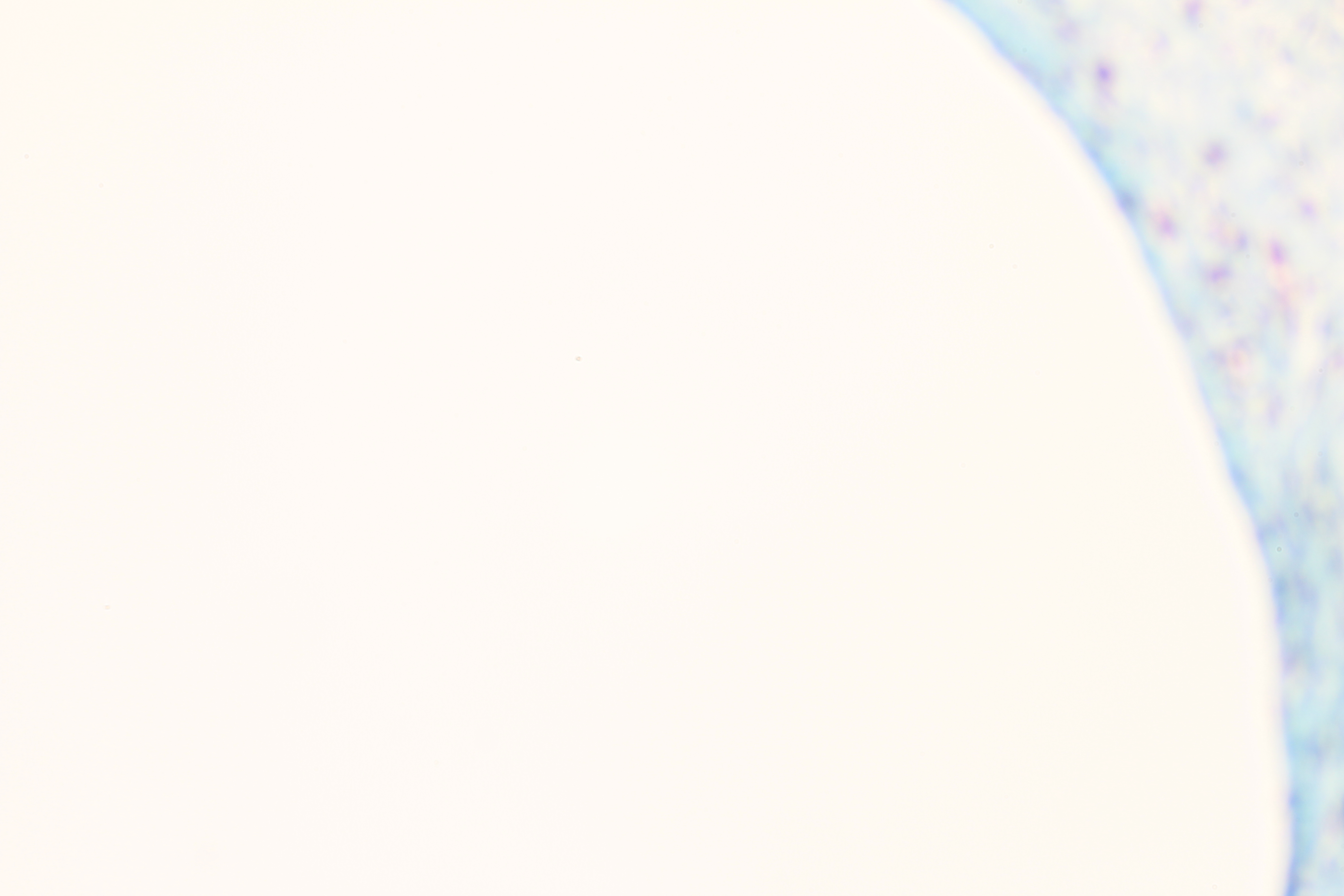

Supplement: S2 Photoset — (ZIP) [file pone.0138054.s003.zip › Multi Tx for Paper - MMC pics 1/IMG_6155.JPG]

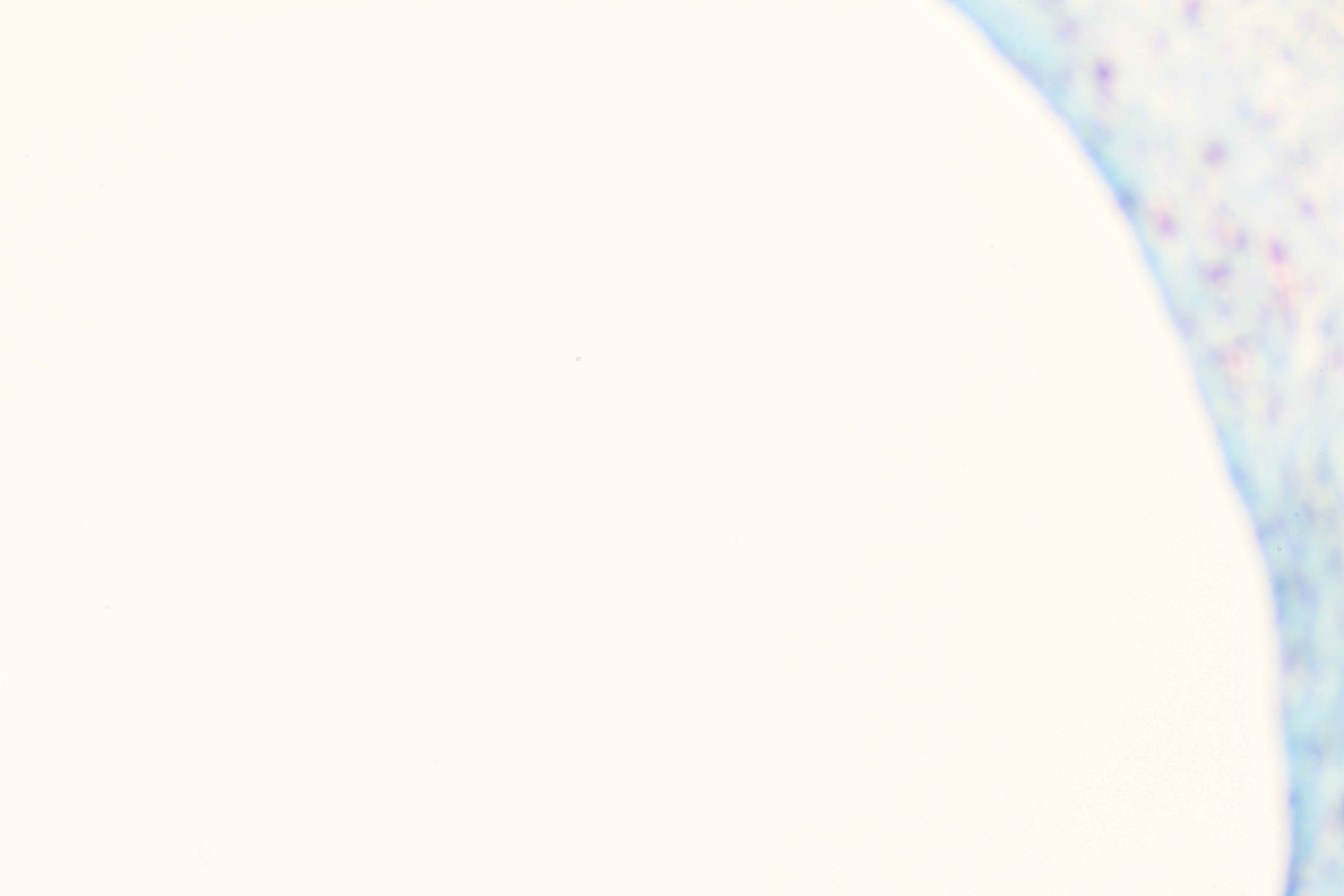

Supplement: S2 Photoset — (ZIP) [file pone.0138054.s003.zip › Multi Tx for Paper - MMC pics 1/IMG_6156.JPG]

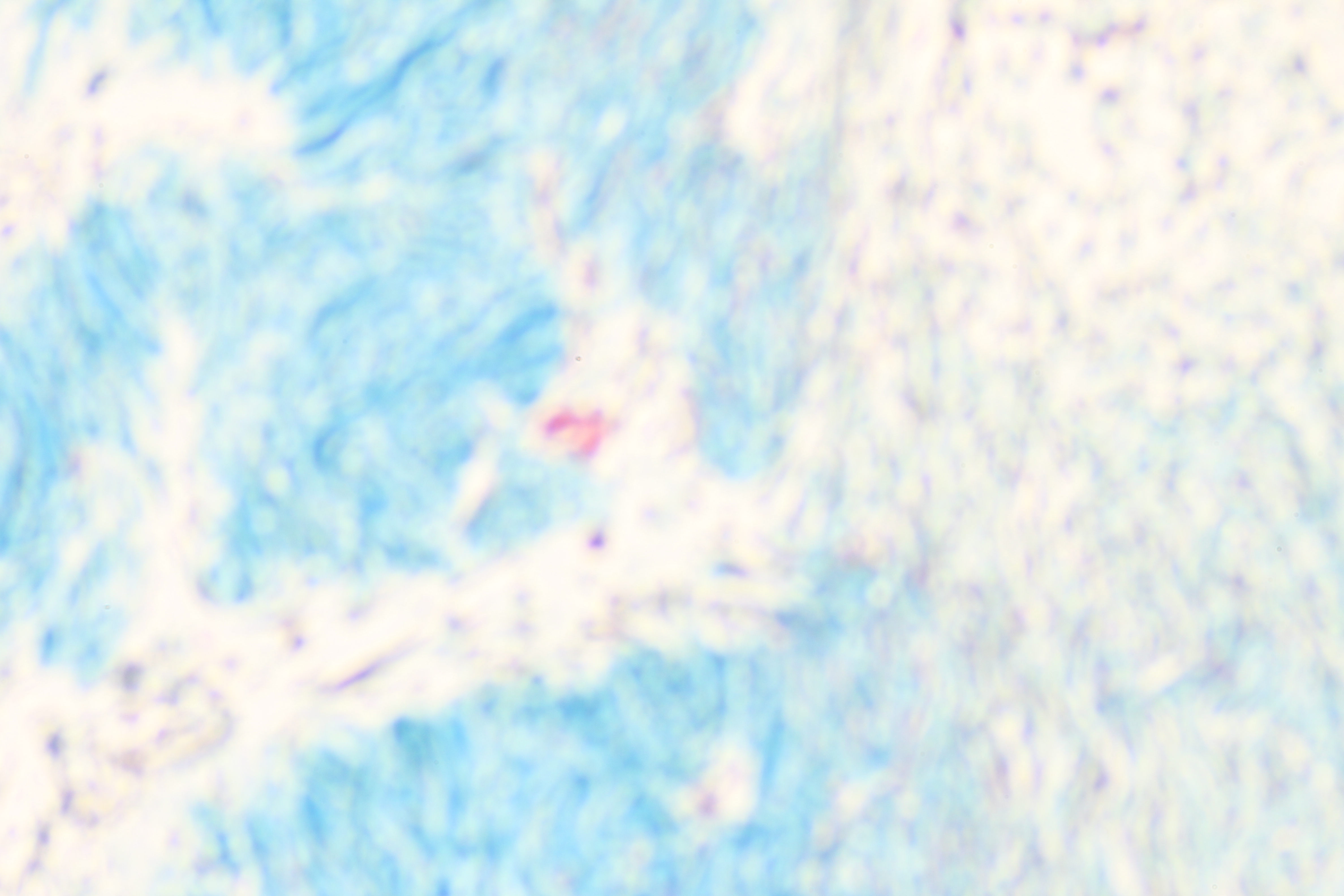

Supplement: S3 Photoset — (ZIP) [file pone.0138054.s004.zip › Multi Tx for Paper - MMC pics 2/IMG_6157.JPG]

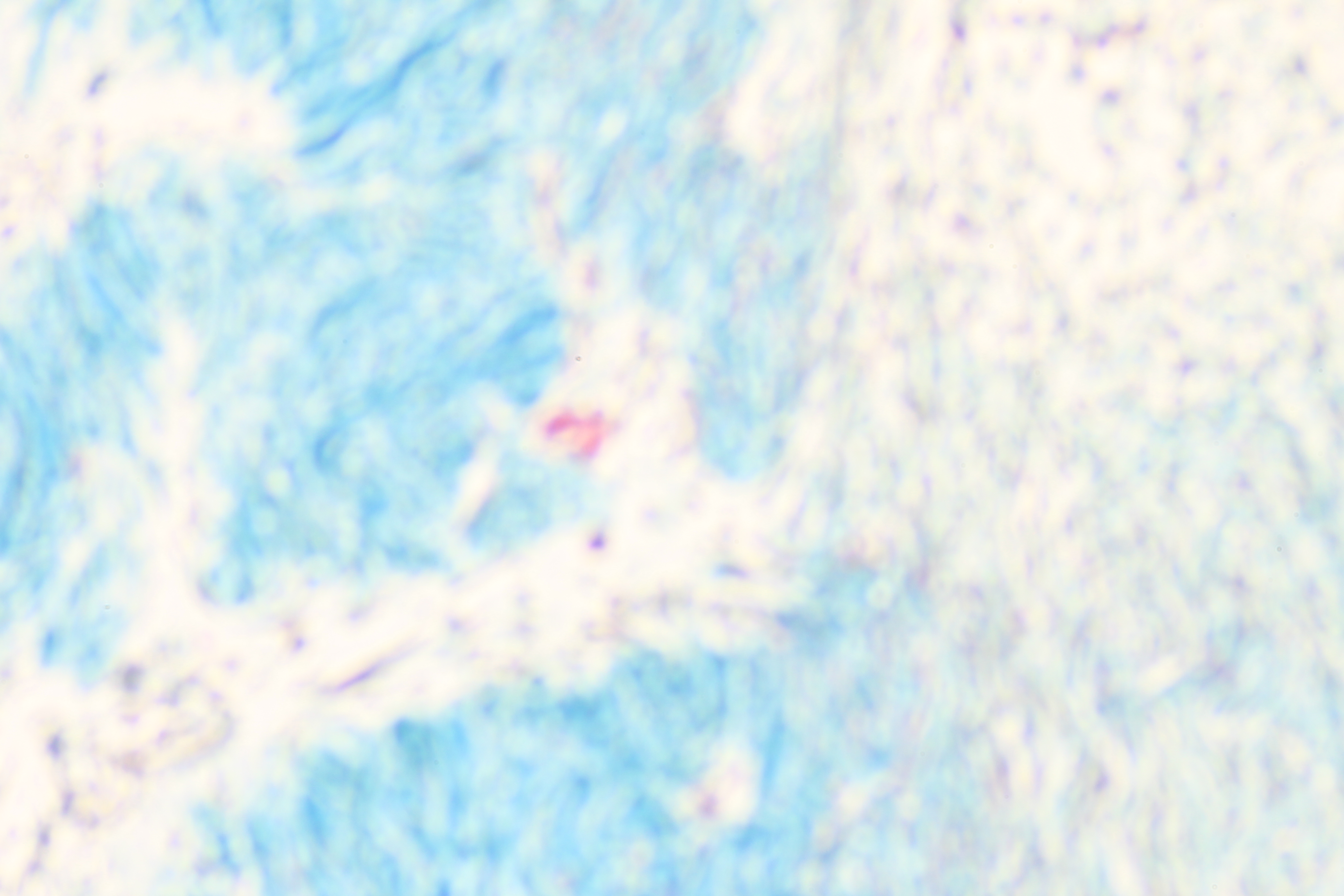

Supplement: S3 Photoset — (ZIP) [file pone.0138054.s004.zip › Multi Tx for Paper - MMC pics 2/IMG_6158.JPG]

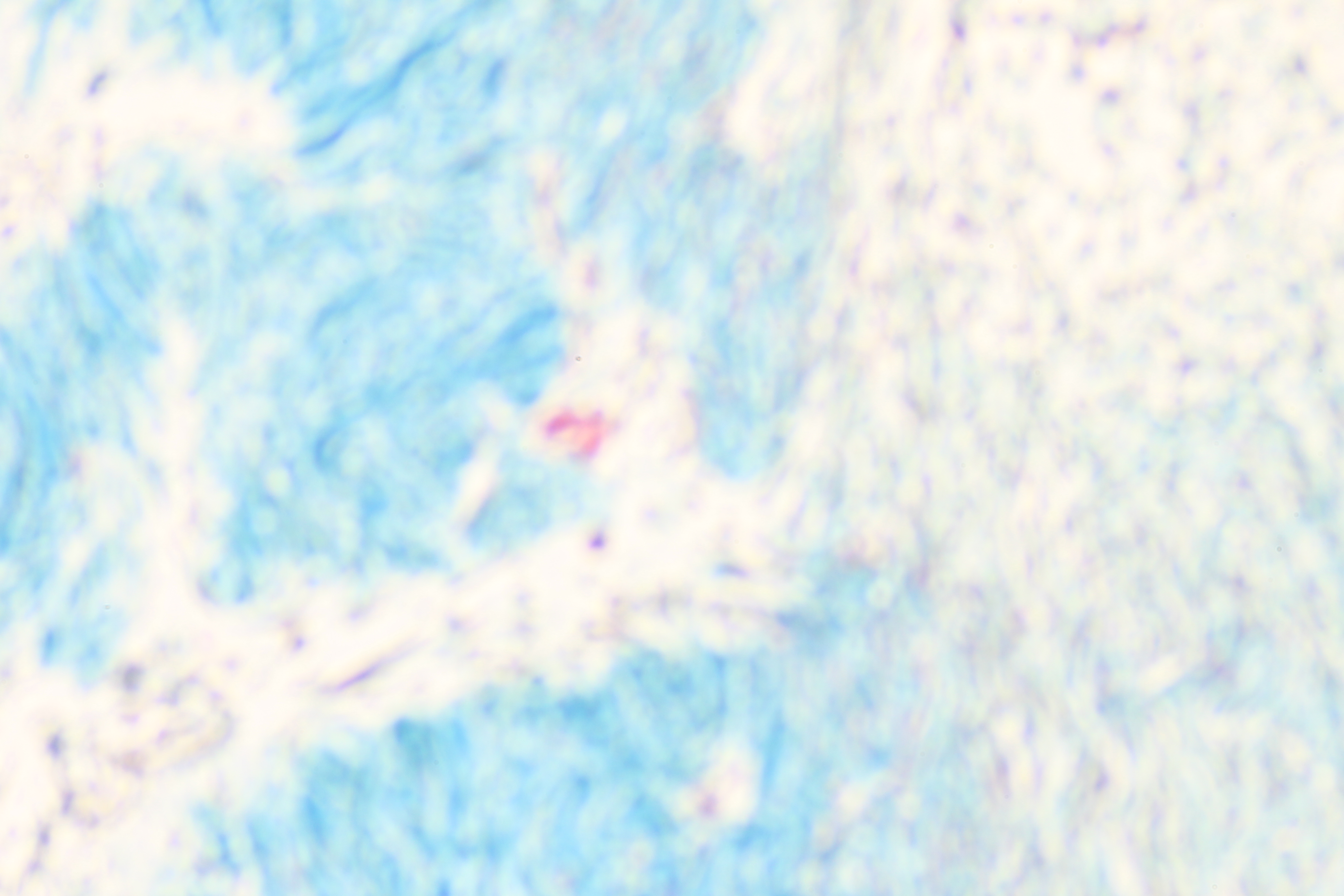

Supplement: S3 Photoset — (ZIP) [file pone.0138054.s004.zip › Multi Tx for Paper - MMC pics 2/IMG_6159.JPG]

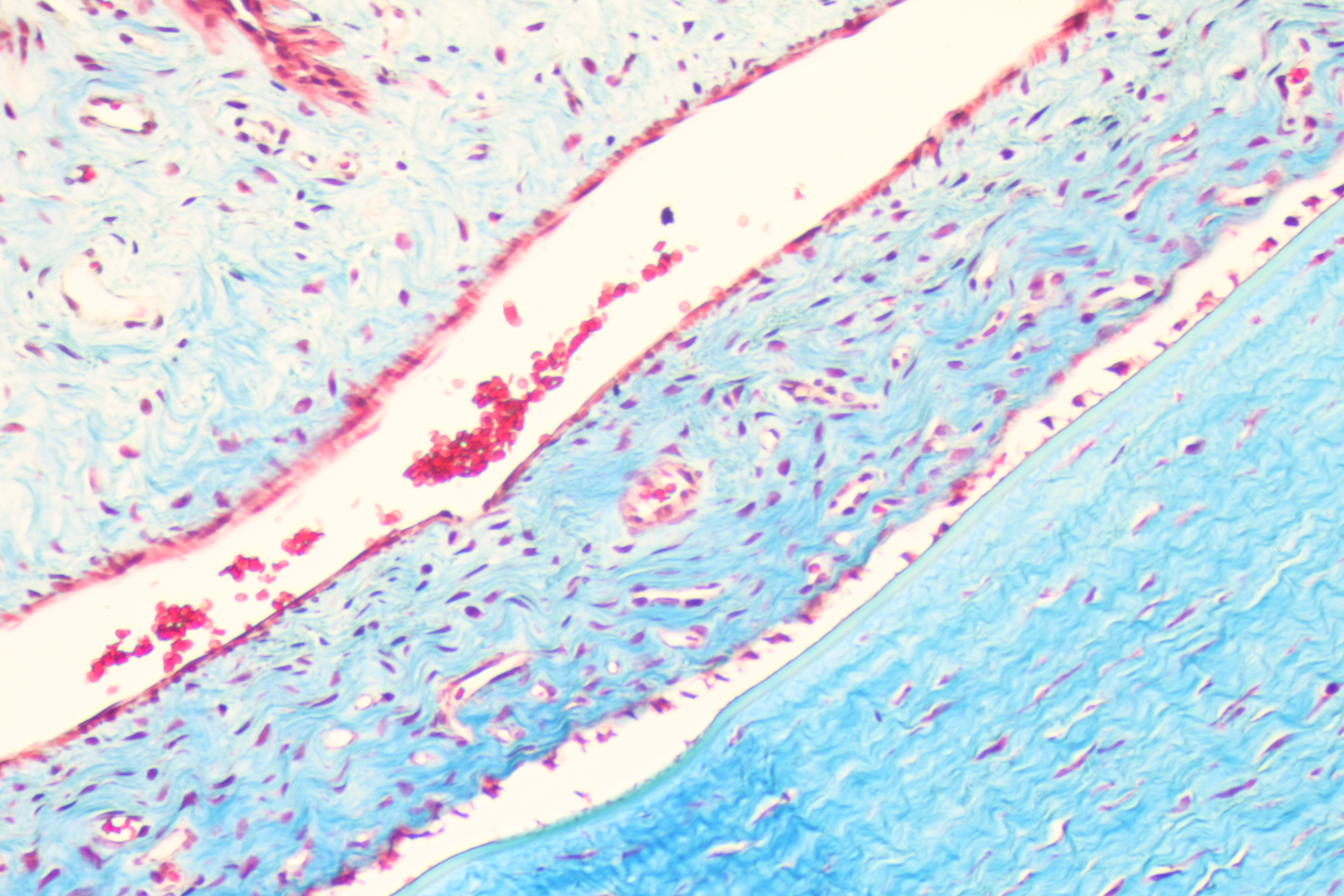

Supplement: S3 Photoset — (ZIP) [file pone.0138054.s004.zip › Multi Tx for Paper - MMC pics 2/IMG_6189.JPG]

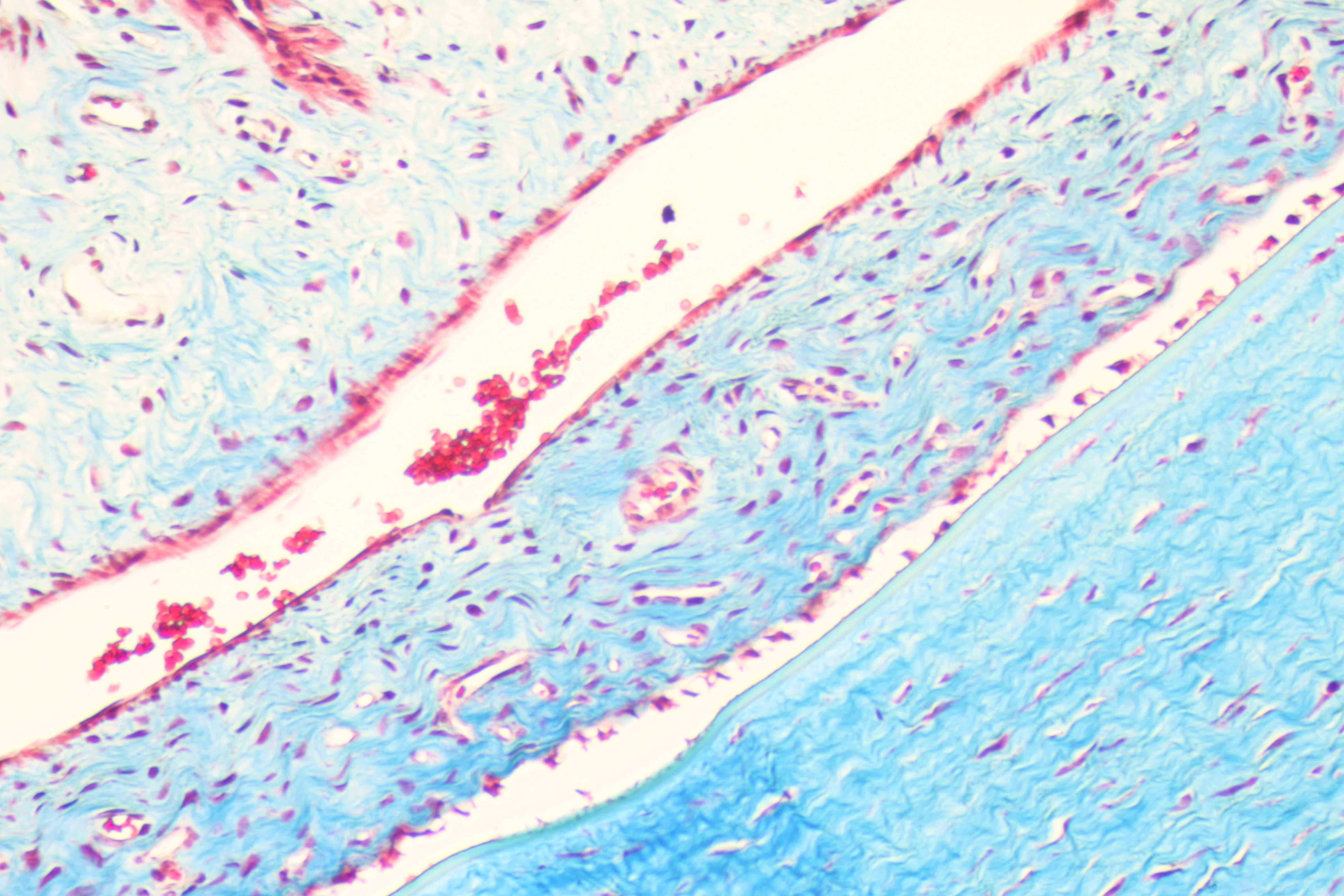

Supplement: S3 Photoset — (ZIP) [file pone.0138054.s004.zip › Multi Tx for Paper - MMC pics 2/IMG_6190.JPG]

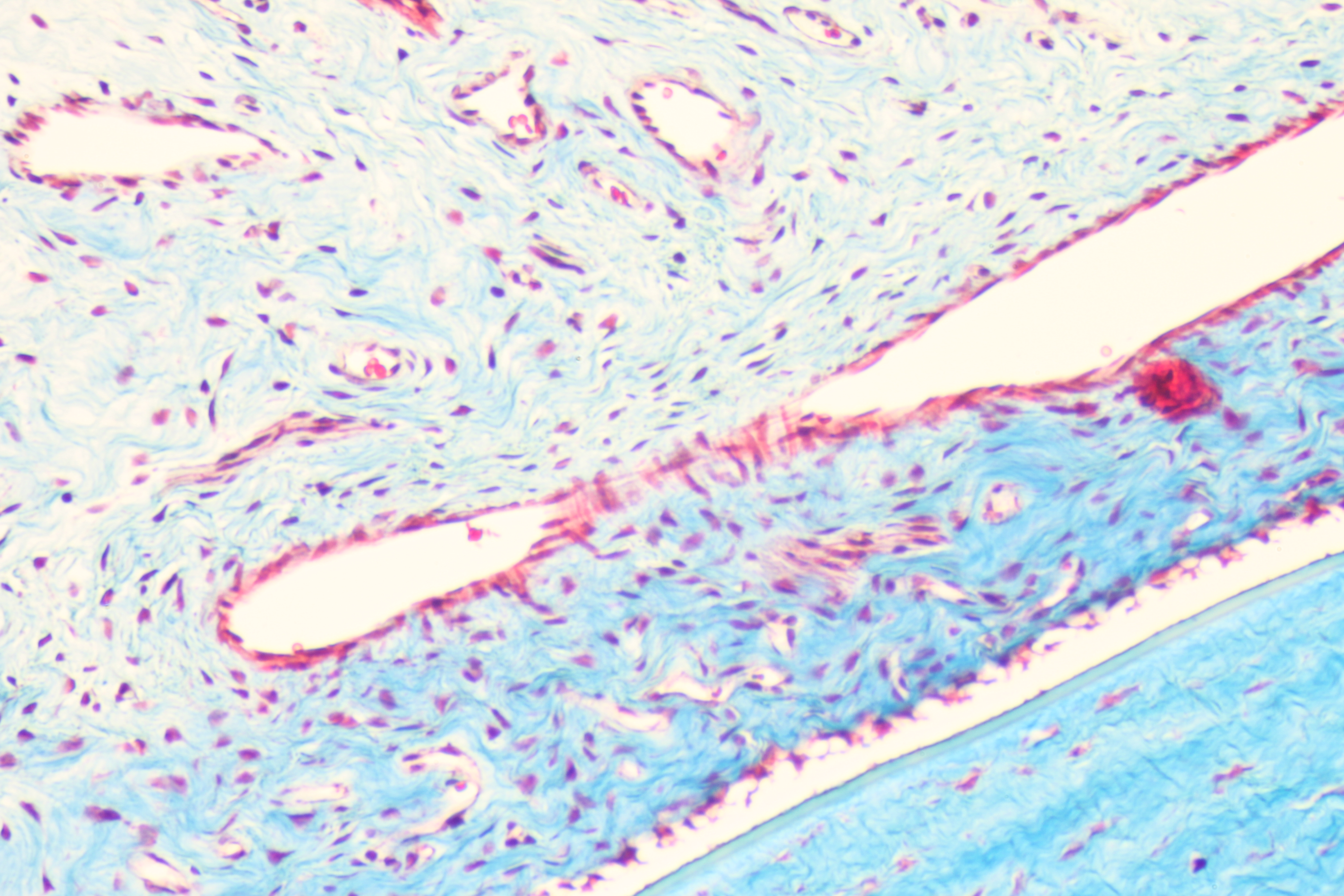

Supplement: S3 Photoset — (ZIP) [file pone.0138054.s004.zip › Multi Tx for Paper - MMC pics 2/IMG_6191.JPG]

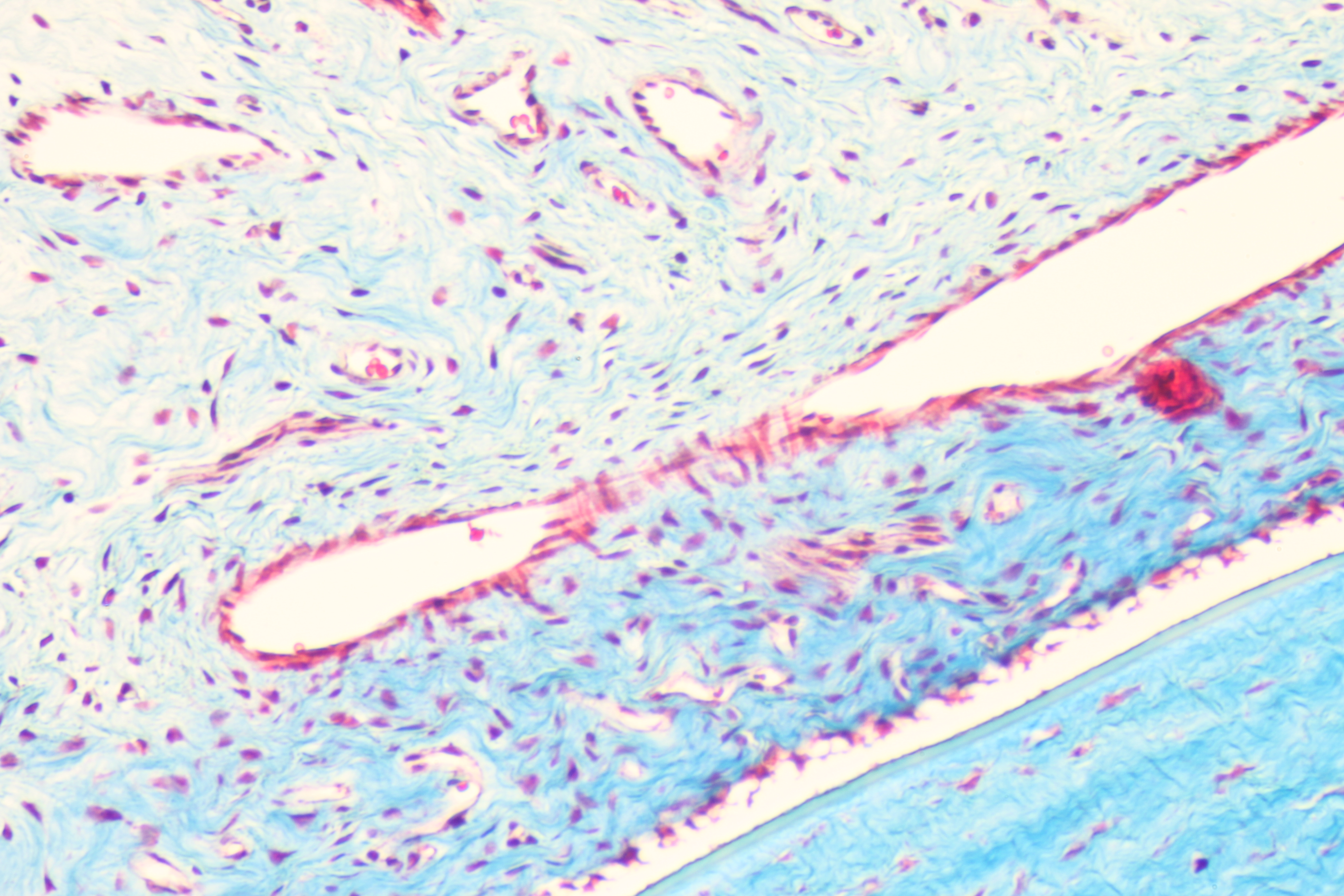

Supplement: S3 Photoset — (ZIP) [file pone.0138054.s004.zip › Multi Tx for Paper - MMC pics 2/IMG_6192.JPG]

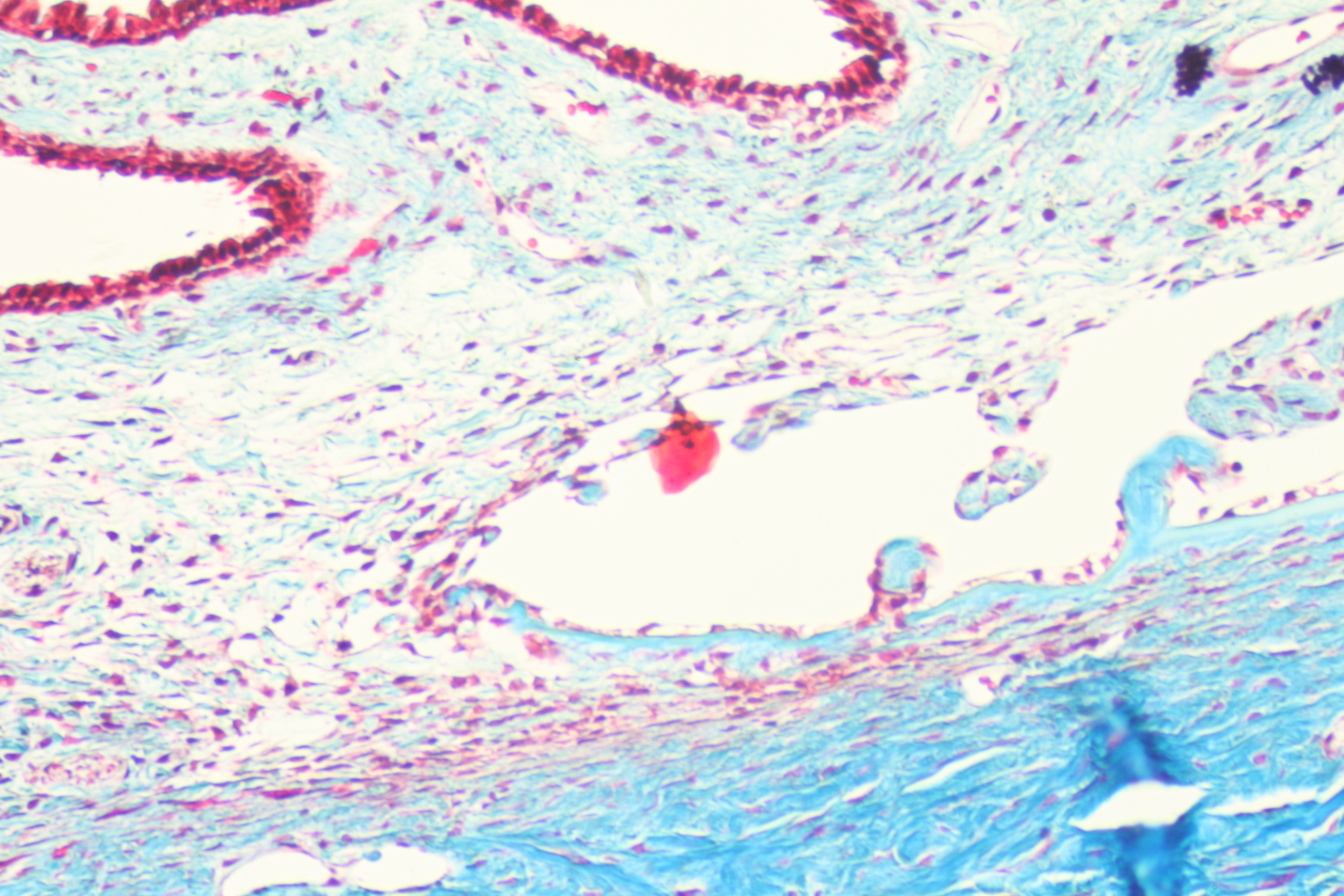

Supplement: S3 Photoset — (ZIP) [file pone.0138054.s004.zip › Multi Tx for Paper - MMC pics 2/IMG_6193.JPG]

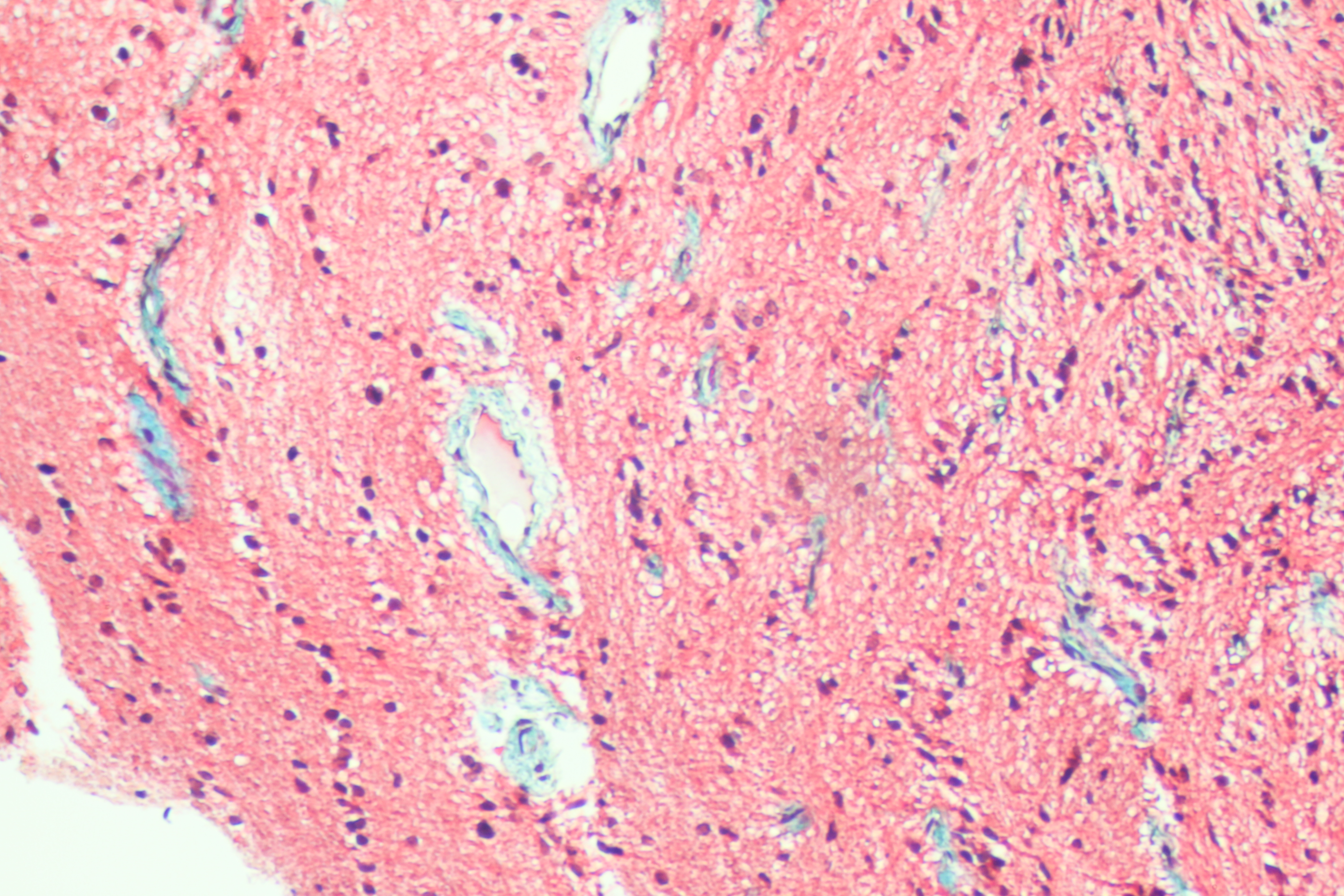

Supplement: S3 Photoset — (ZIP) [file pone.0138054.s004.zip › Multi Tx for Paper - MMC pics 2/IMG_6218.JPG]

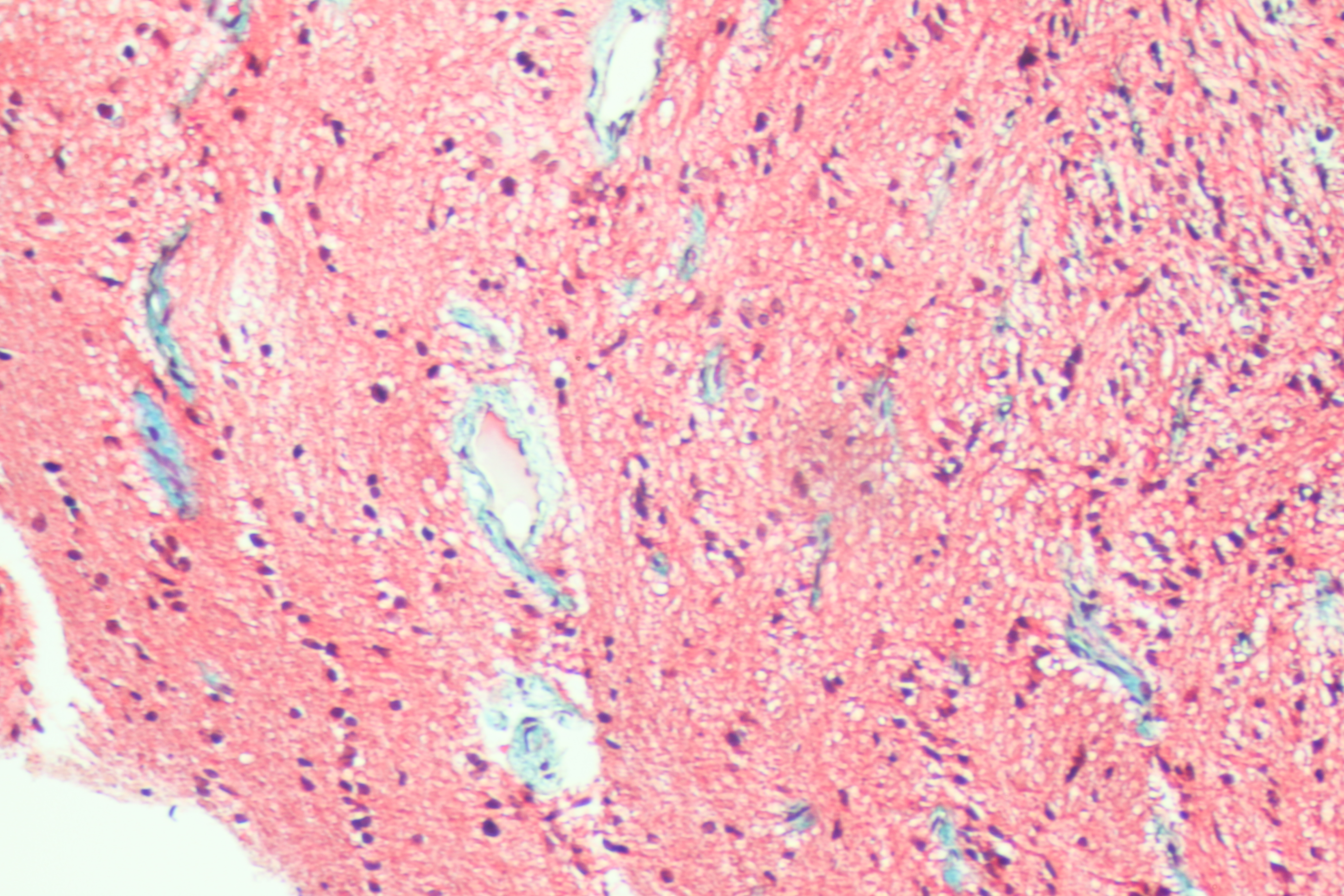

Supplement: S3 Photoset — (ZIP) [file pone.0138054.s004.zip › Multi Tx for Paper - MMC pics 2/IMG_6219.JPG]

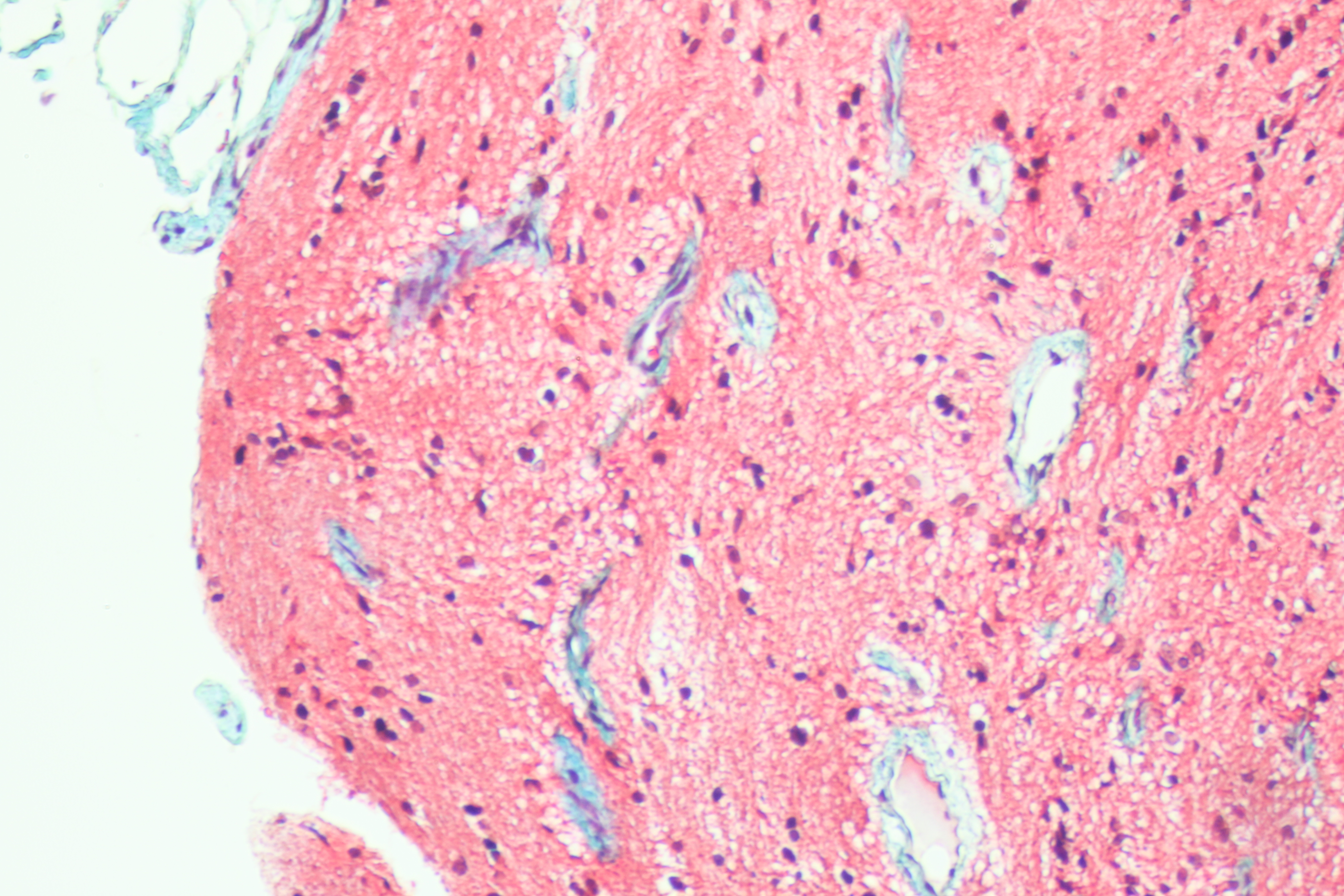

Supplement: S3 Photoset — (ZIP) [file pone.0138054.s004.zip › Multi Tx for Paper - MMC pics 2/IMG_6220.JPG]

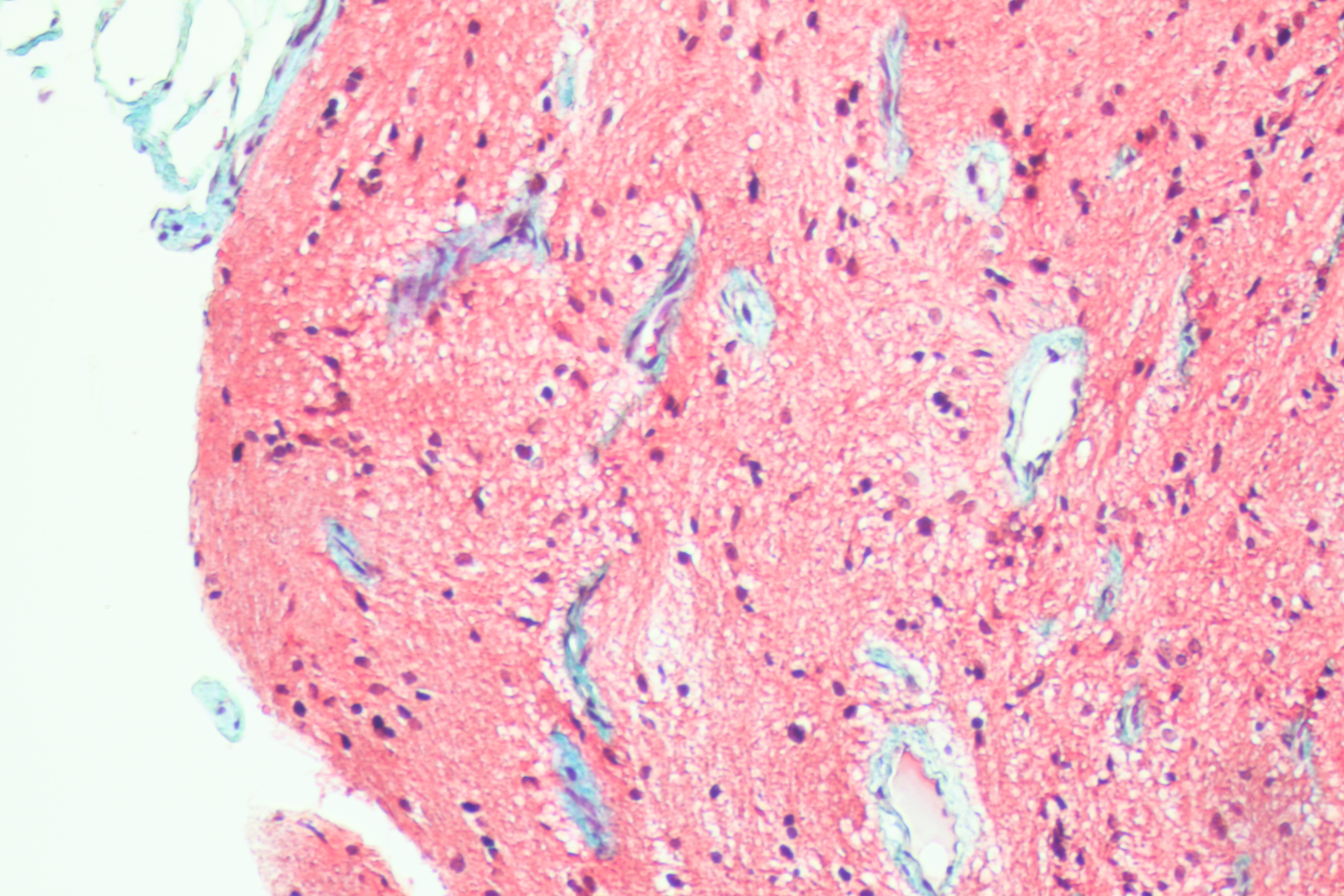

Supplement: S3 Photoset — (ZIP) [file pone.0138054.s004.zip › Multi Tx for Paper - MMC pics 2/IMG_6221.JPG]

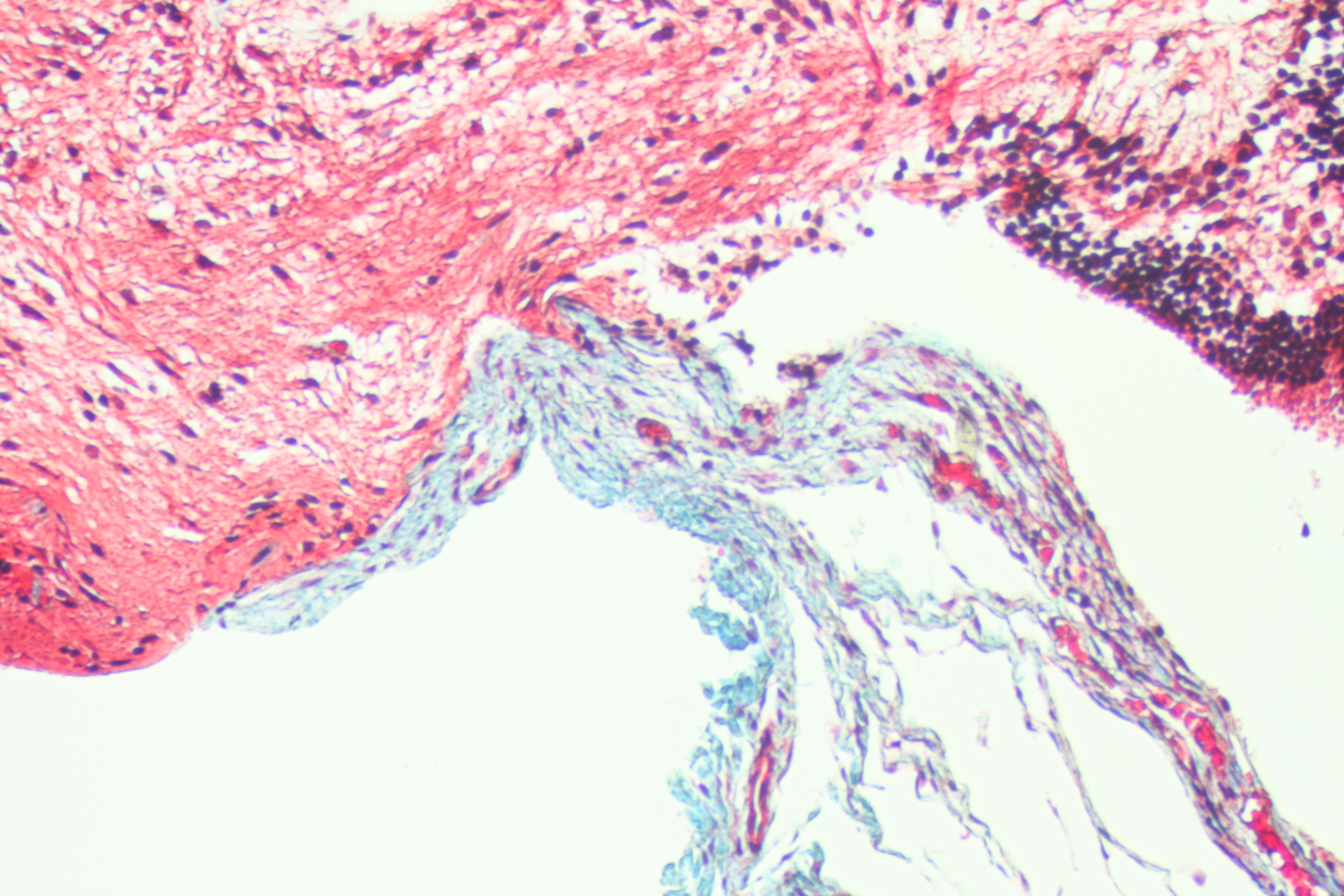

Supplement: S3 Photoset — (ZIP) [file pone.0138054.s004.zip › Multi Tx for Paper - MMC pics 2/IMG_6222.JPG]

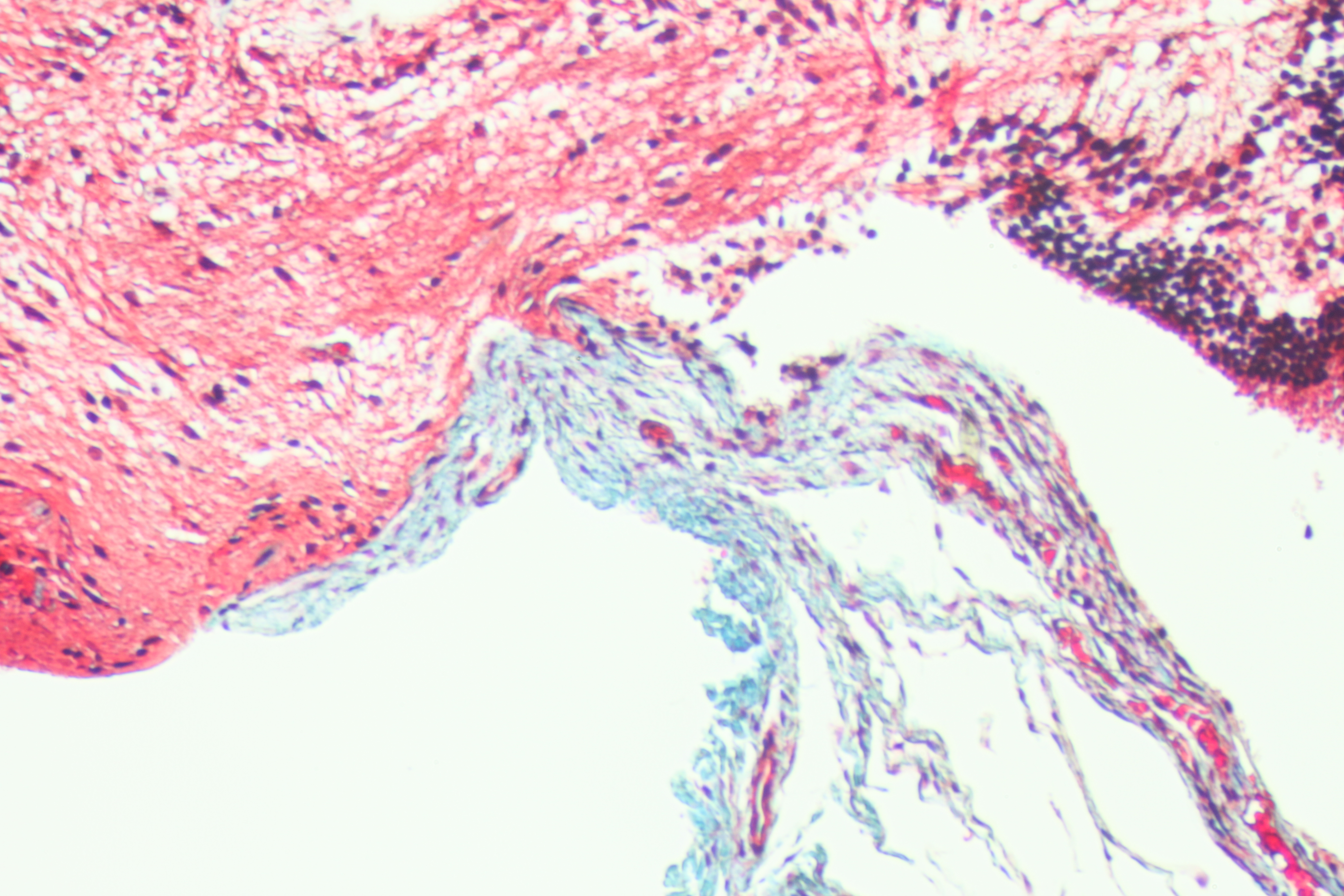

Supplement: S3 Photoset — (ZIP) [file pone.0138054.s004.zip › Multi Tx for Paper - MMC pics 2/IMG_6223.JPG]

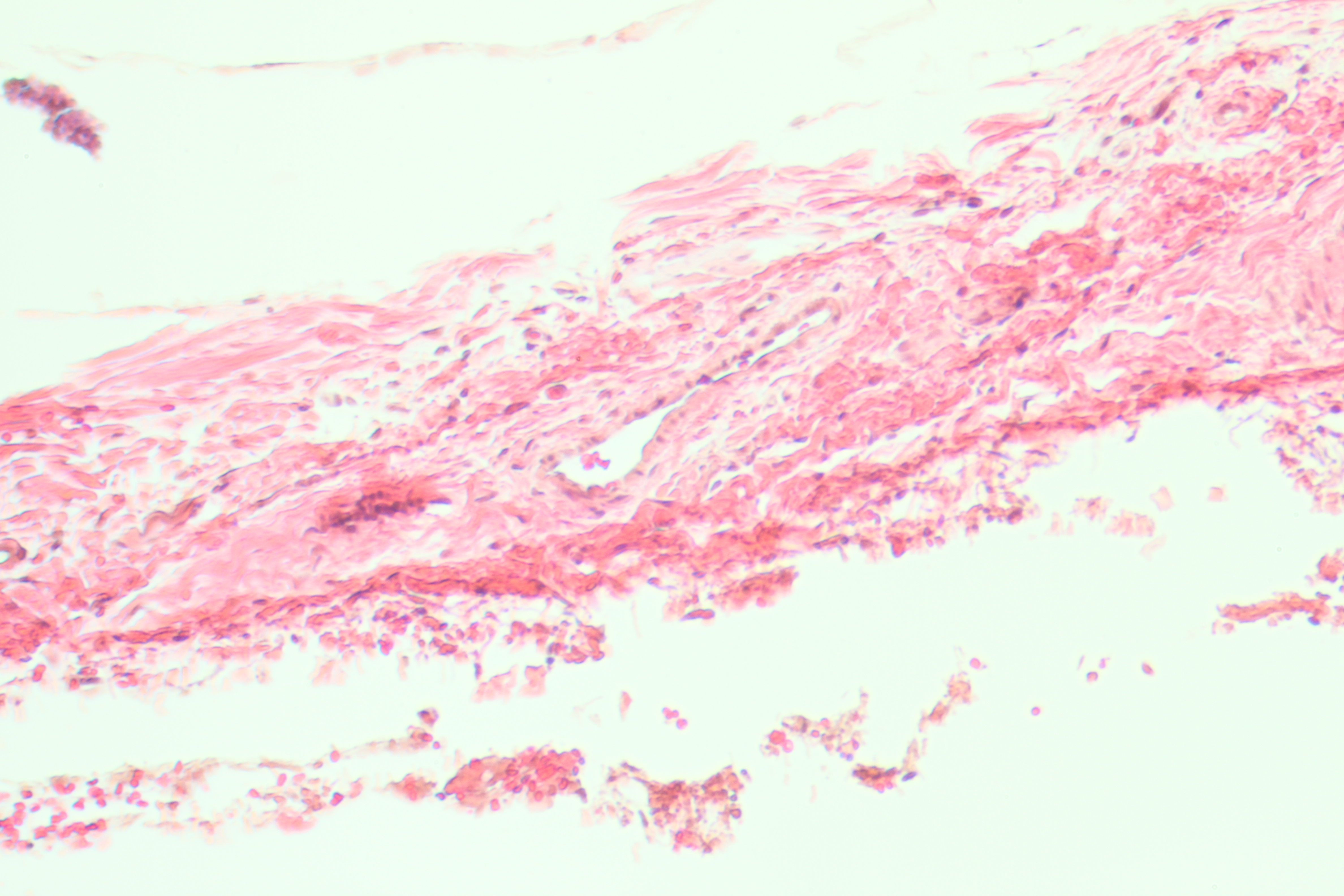

Supplement: S3 Photoset — (ZIP) [file pone.0138054.s004.zip › Multi Tx for Paper - MMC pics 2/IMG_6250.JPG]

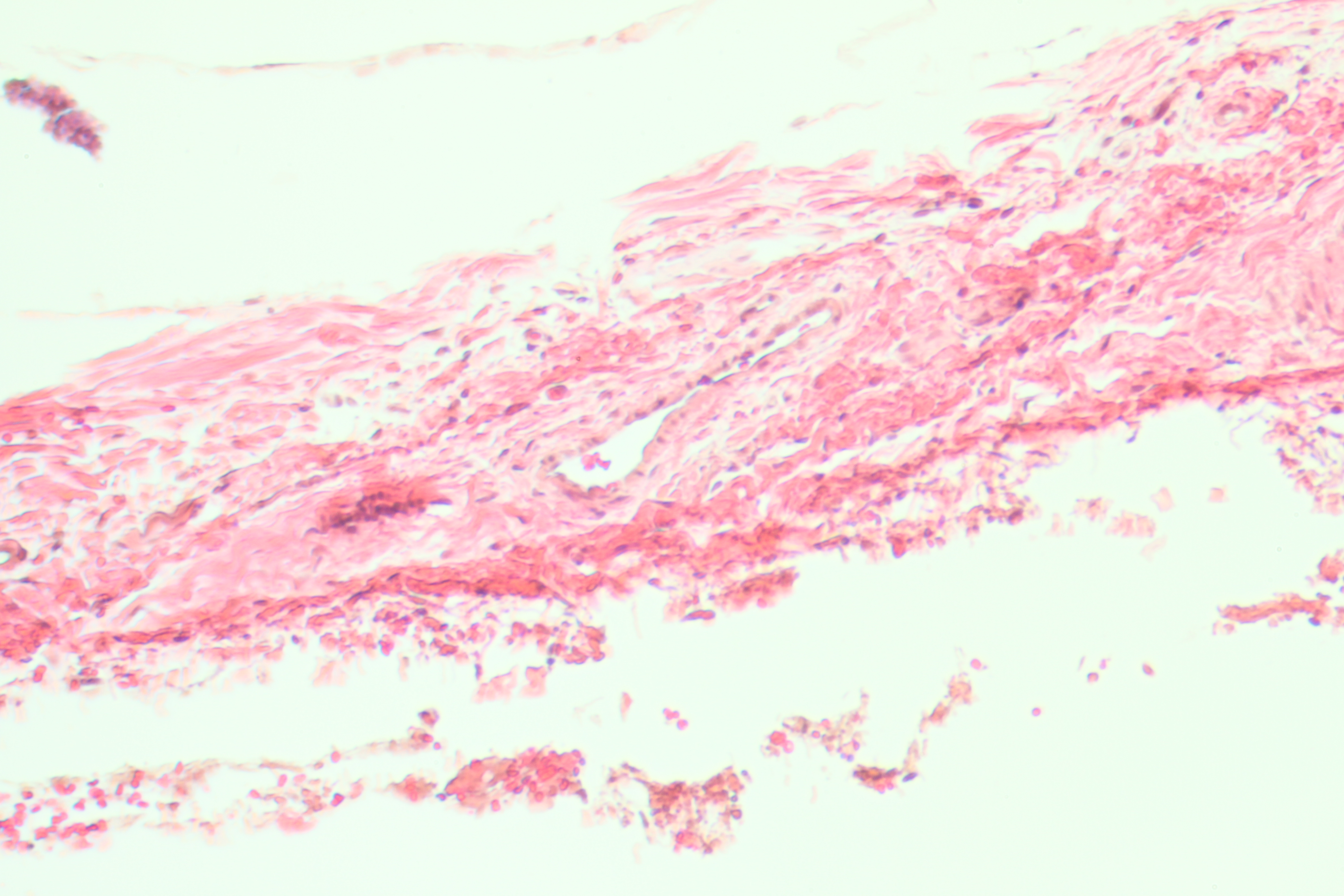

Supplement: S3 Photoset — (ZIP) [file pone.0138054.s004.zip › Multi Tx for Paper - MMC pics 2/IMG_6251.JPG]

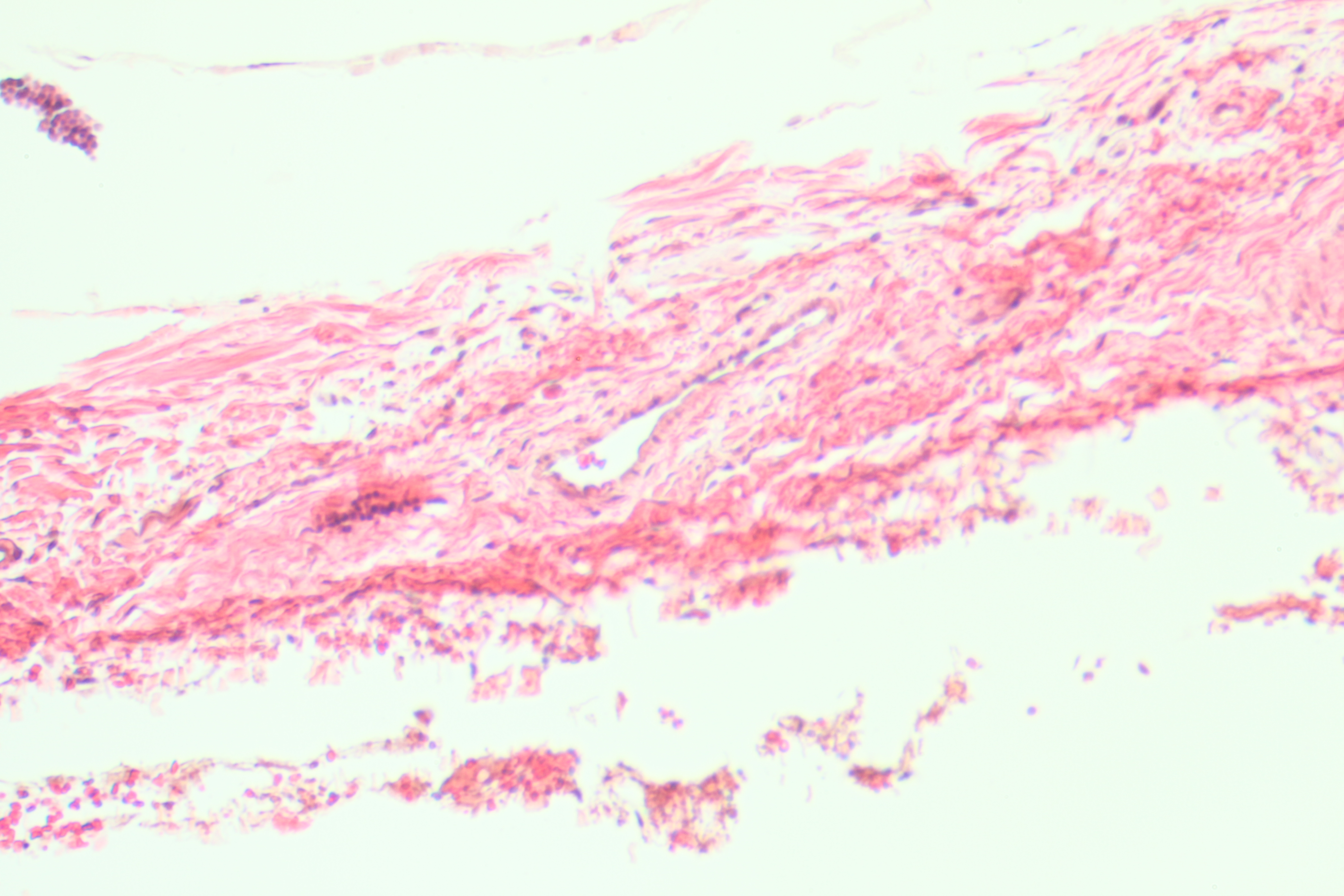

Supplement: S3 Photoset — (ZIP) [file pone.0138054.s004.zip › Multi Tx for Paper - MMC pics 2/IMG_6252.JPG]

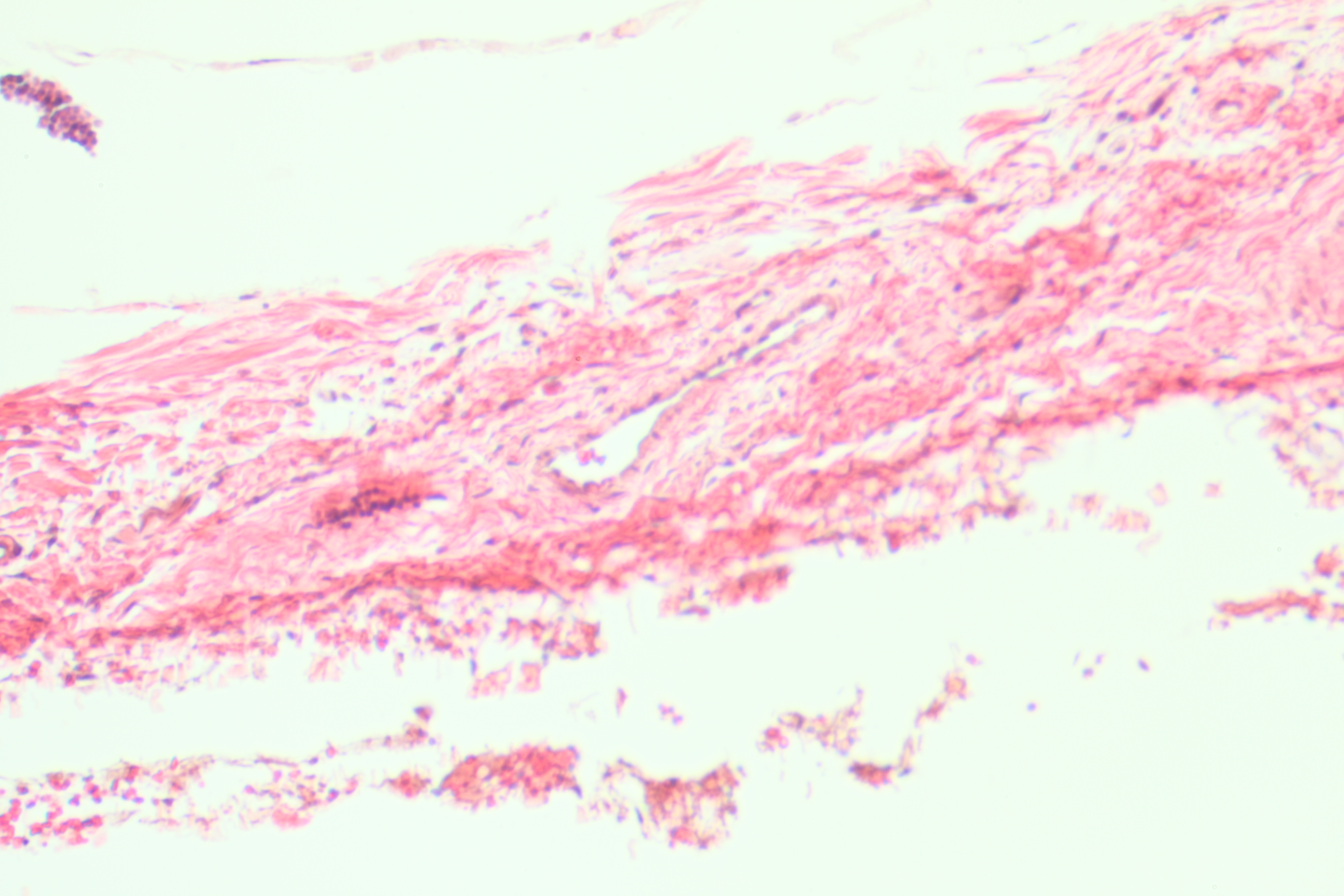

Supplement: S3 Photoset — (ZIP) [file pone.0138054.s004.zip › Multi Tx for Paper - MMC pics 2/IMG_6253.JPG]

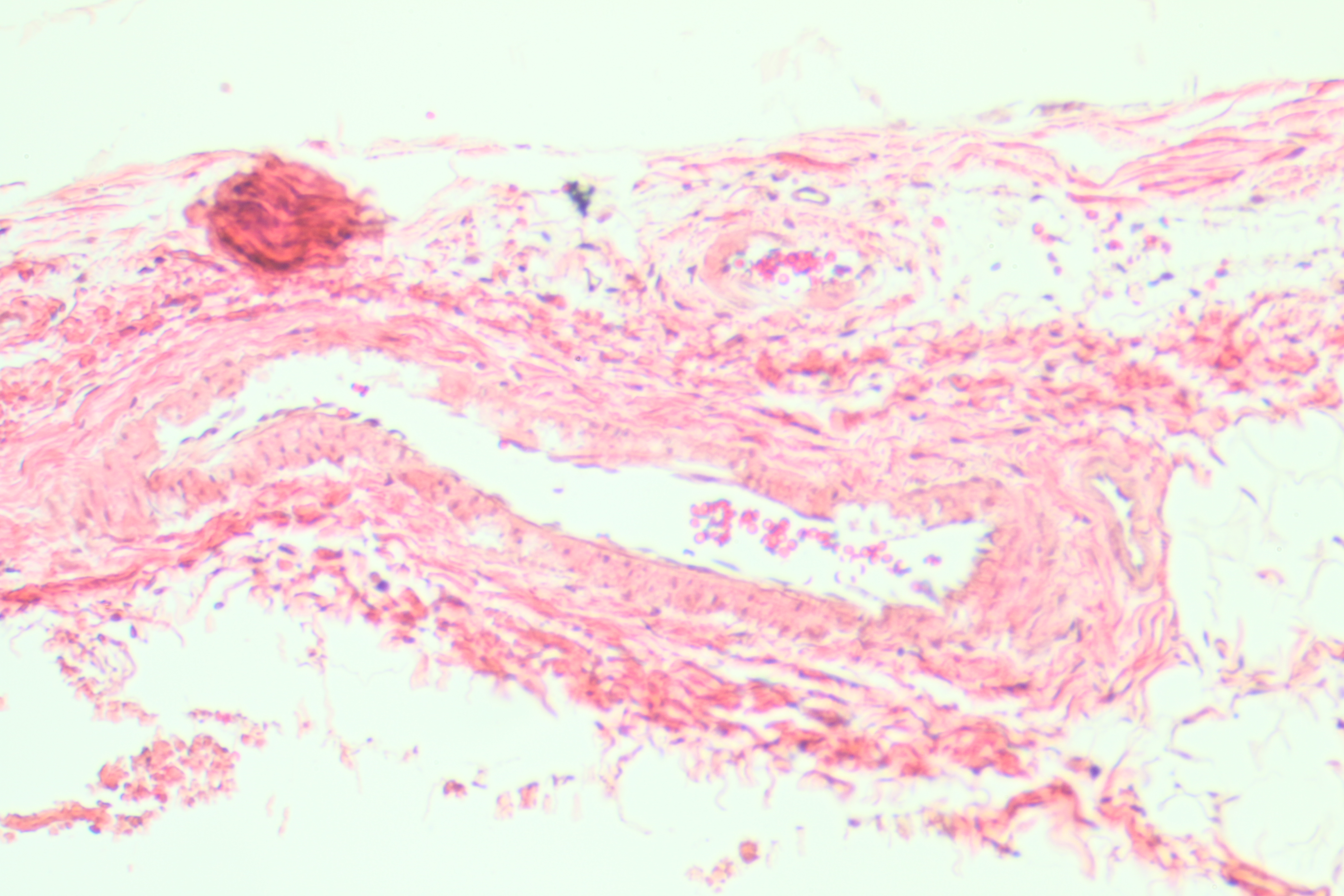

Supplement: S3 Photoset — (ZIP) [file pone.0138054.s004.zip › Multi Tx for Paper - MMC pics 2/IMG_6254.JPG]

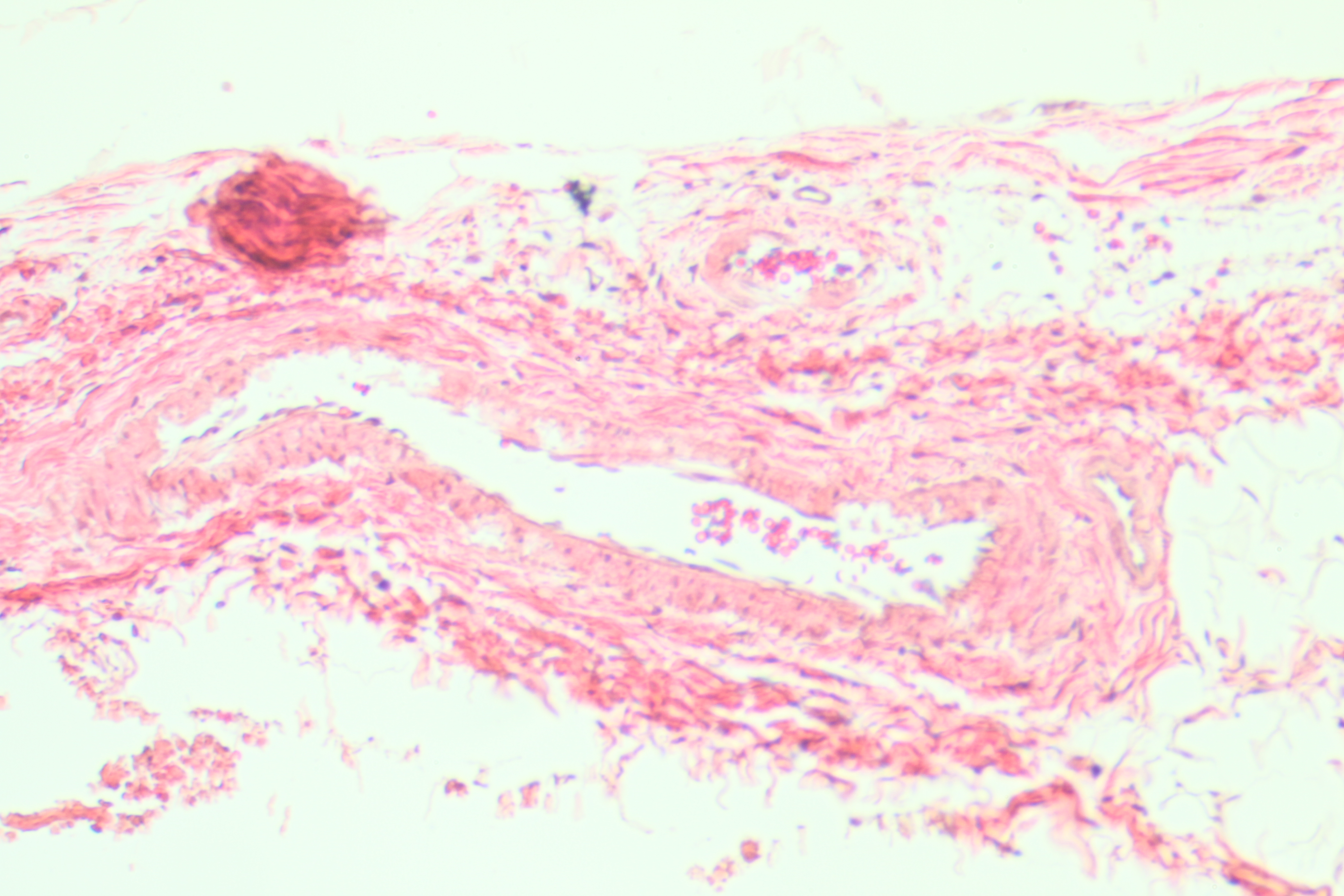

Supplement: S3 Photoset — (ZIP) [file pone.0138054.s004.zip › Multi Tx for Paper - MMC pics 2/IMG_6255.JPG]

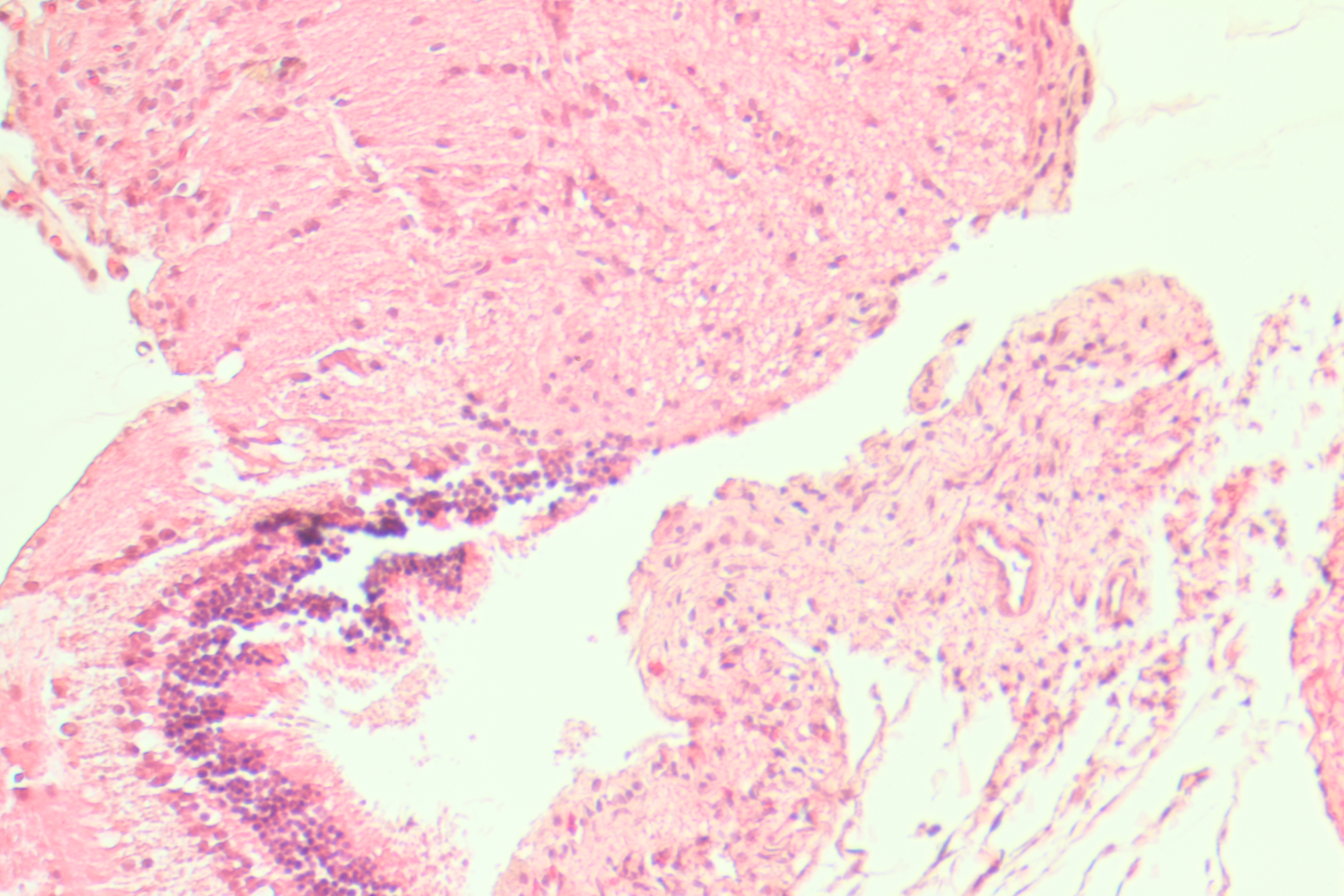

Supplement: S3 Photoset — (ZIP) [file pone.0138054.s004.zip › Multi Tx for Paper - MMC pics 2/IMG_6256.JPG]

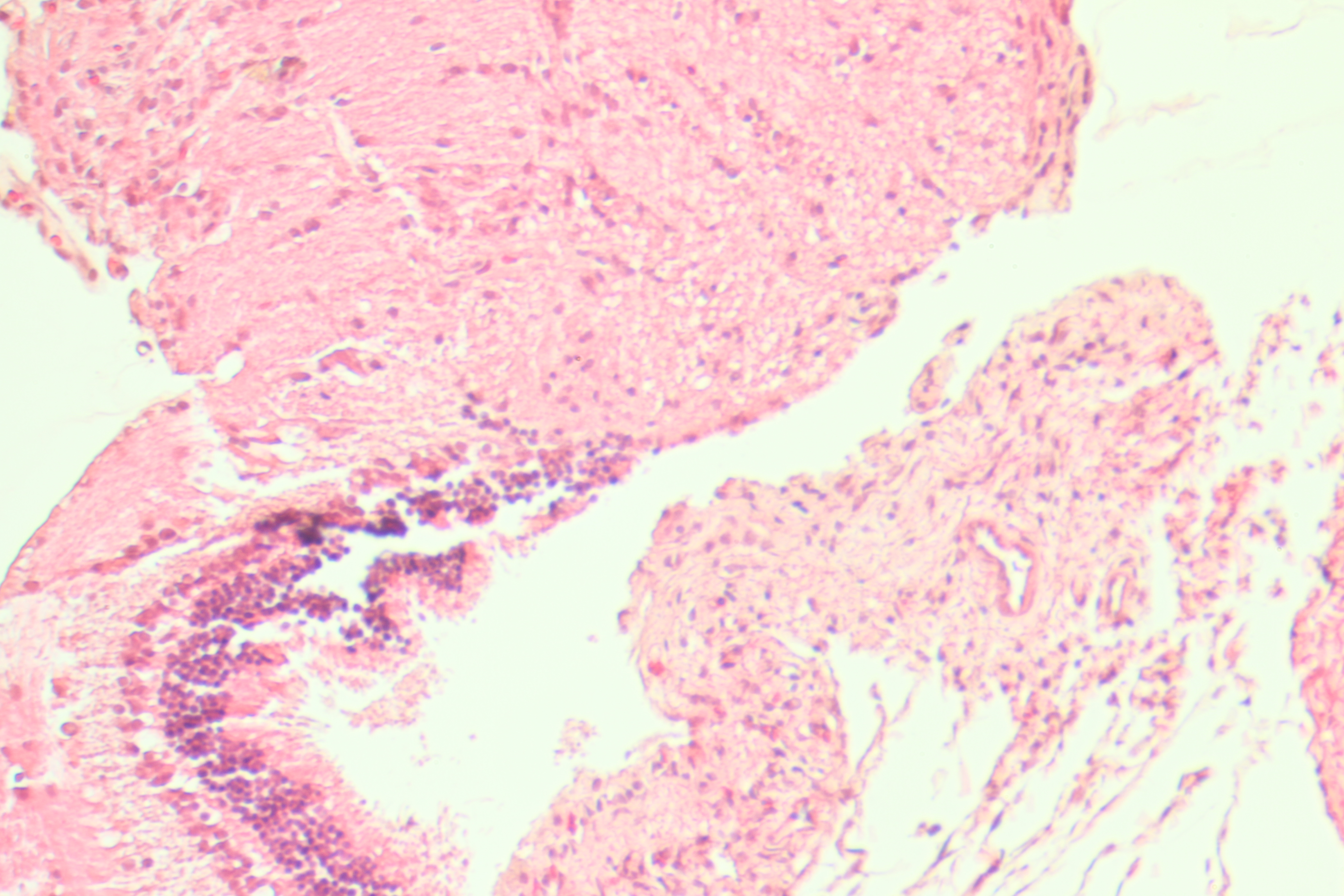

Supplement: S3 Photoset — (ZIP) [file pone.0138054.s004.zip › Multi Tx for Paper - MMC pics 2/IMG_6257.JPG]

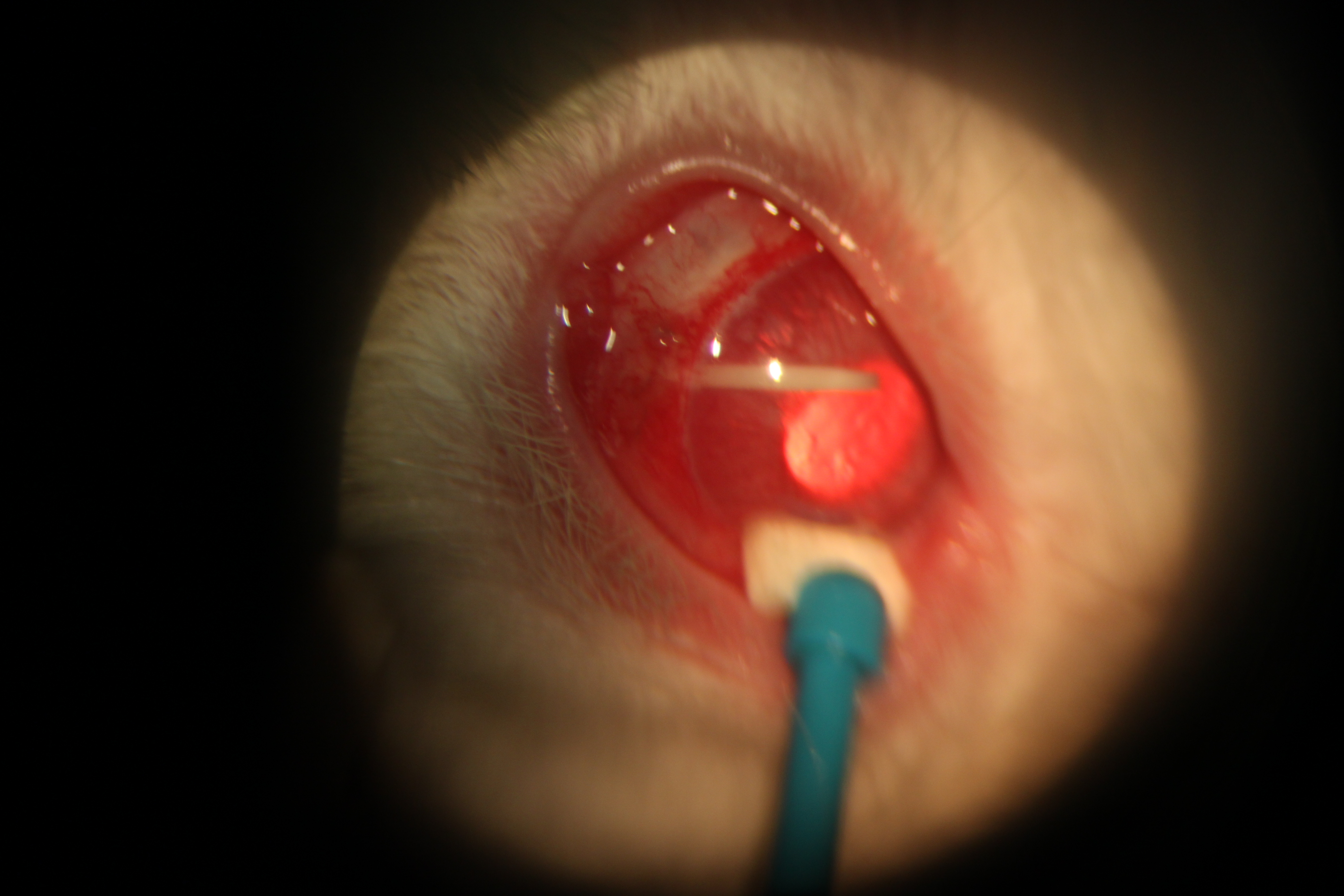

Supplement: S4 Photoset — (ZIP) [file pone.0138054.s005.zip › Multi Tx for Paper - SaratinIlomastat pics 1/IMG_1590.JPG]

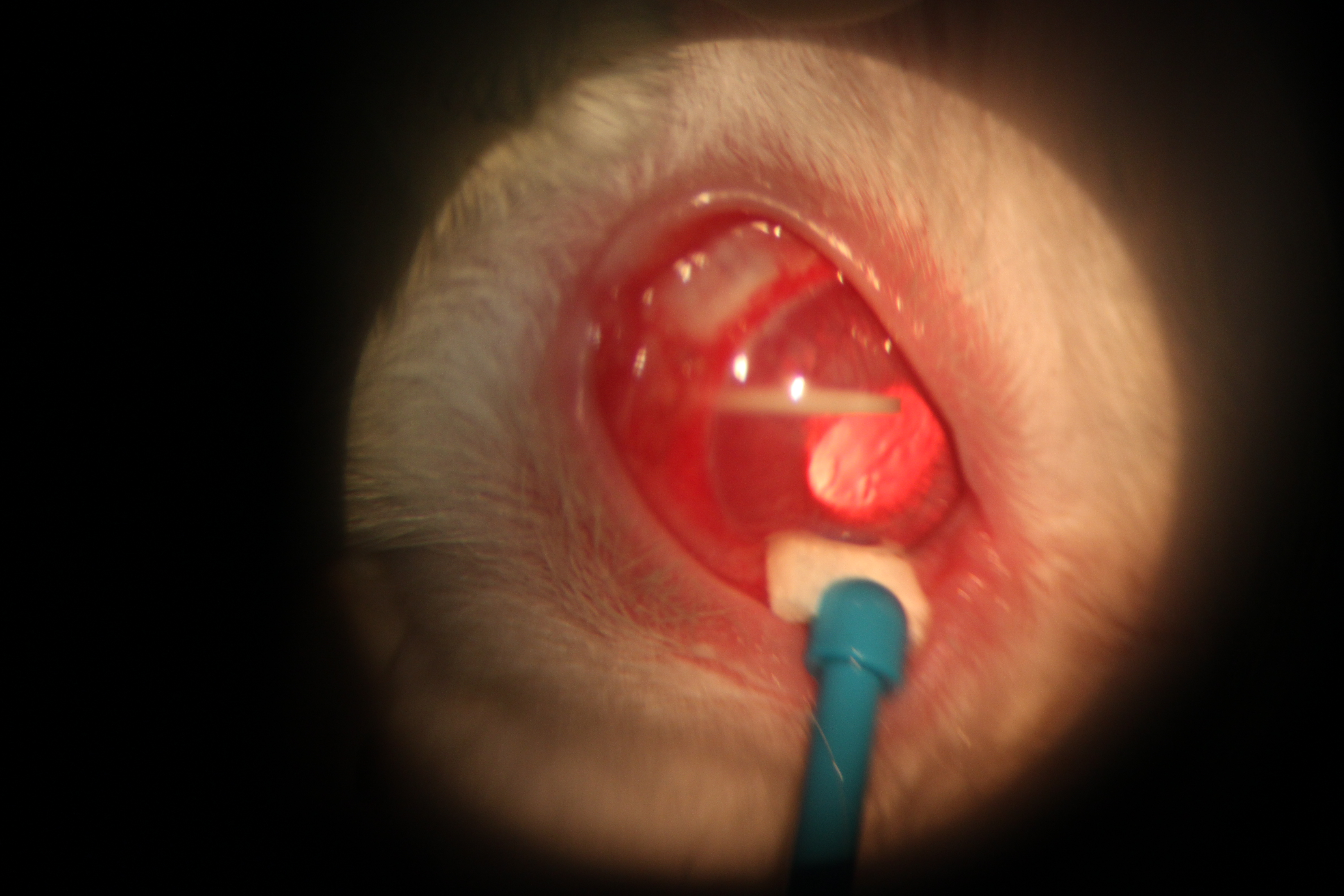

Supplement: S4 Photoset — (ZIP) [file pone.0138054.s005.zip › Multi Tx for Paper - SaratinIlomastat pics 1/IMG_1591.JPG]

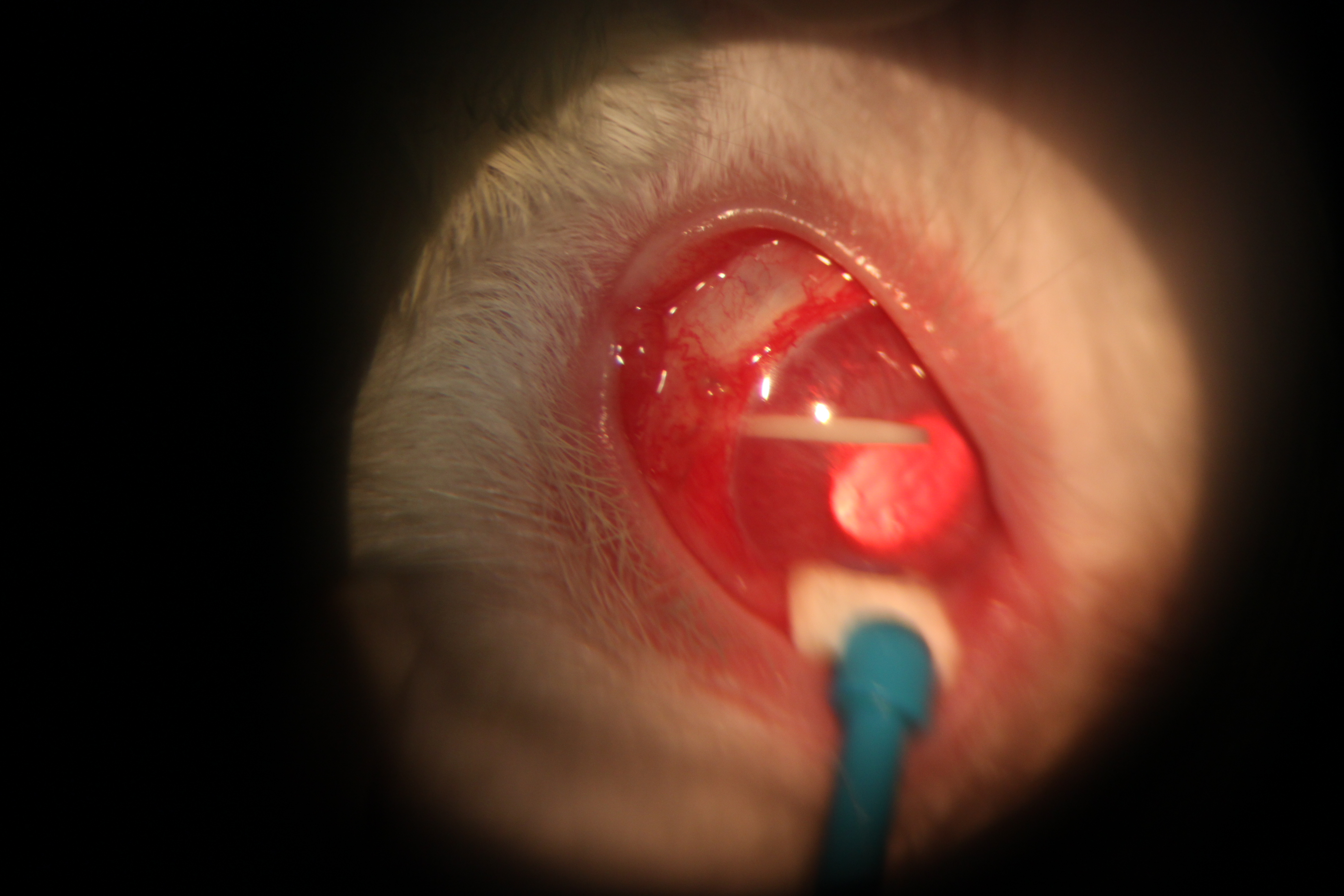

Supplement: S4 Photoset — (ZIP) [file pone.0138054.s005.zip › Multi Tx for Paper - SaratinIlomastat pics 1/IMG_1592.JPG]

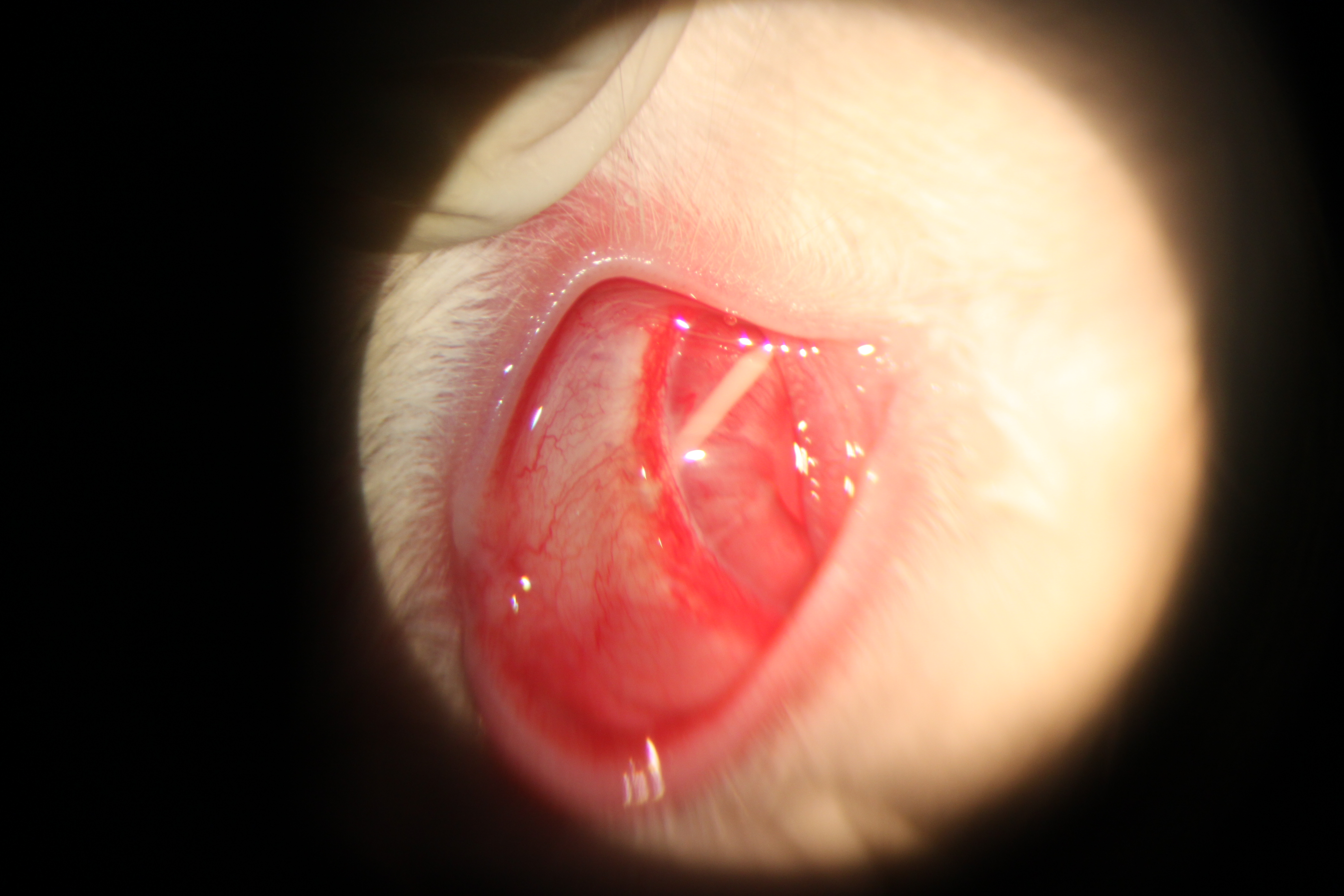

Supplement: S4 Photoset — (ZIP) [file pone.0138054.s005.zip › Multi Tx for Paper - SaratinIlomastat pics 1/IMG_2176.JPG]

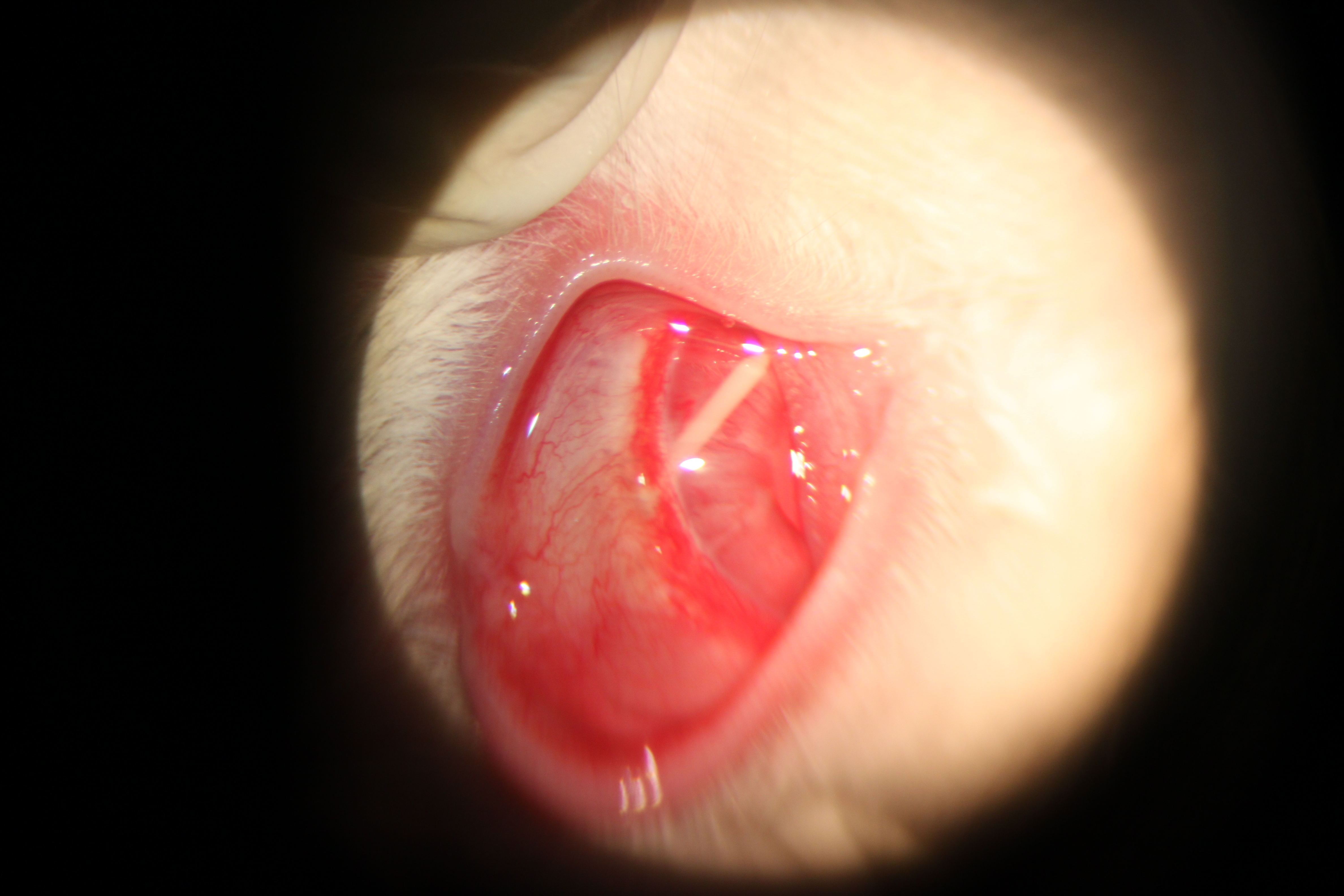

Supplement: S4 Photoset — (ZIP) [file pone.0138054.s005.zip › Multi Tx for Paper - SaratinIlomastat pics 1/IMG_2177.JPG]

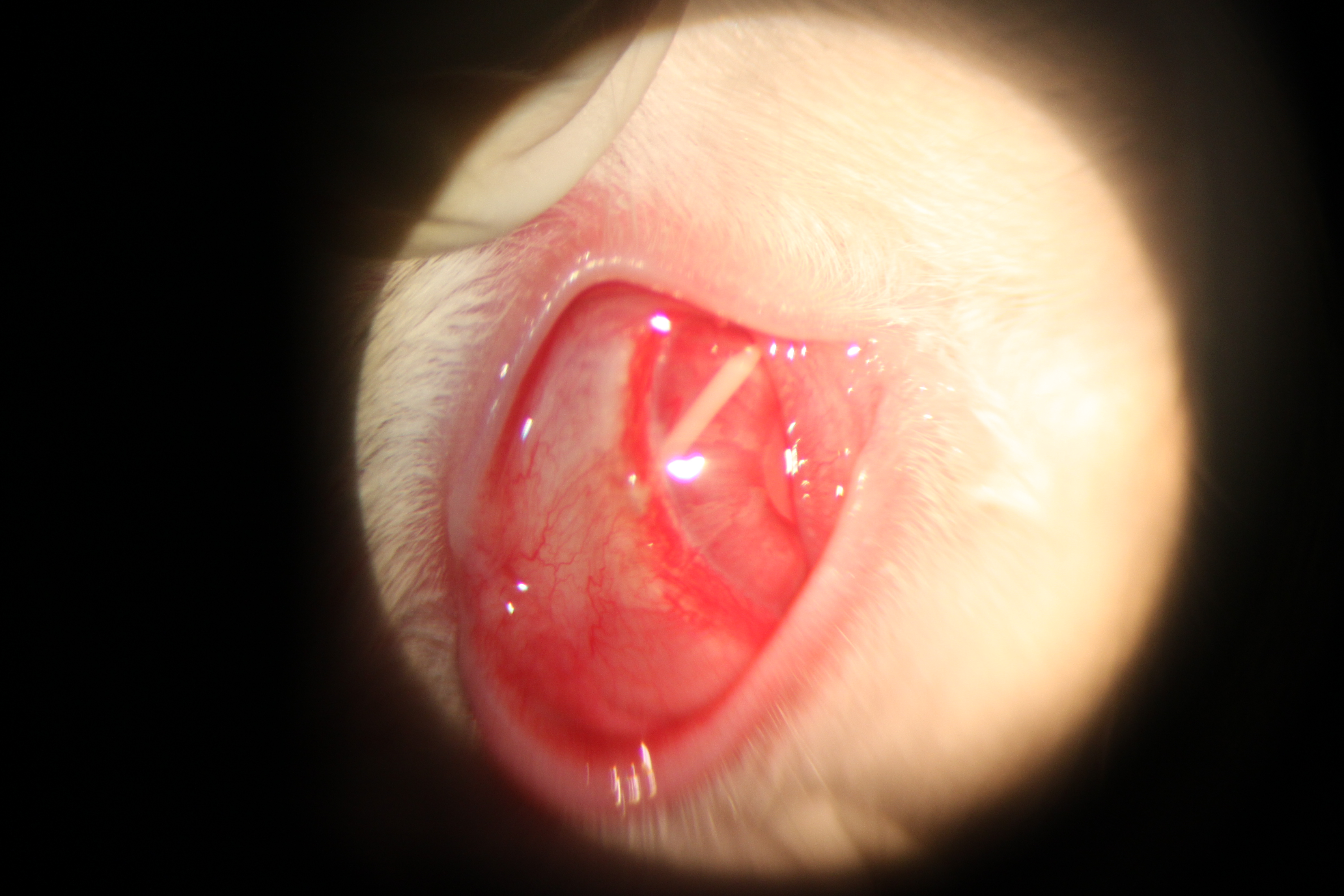

Supplement: S4 Photoset — (ZIP) [file pone.0138054.s005.zip › Multi Tx for Paper - SaratinIlomastat pics 1/IMG_2178.JPG]

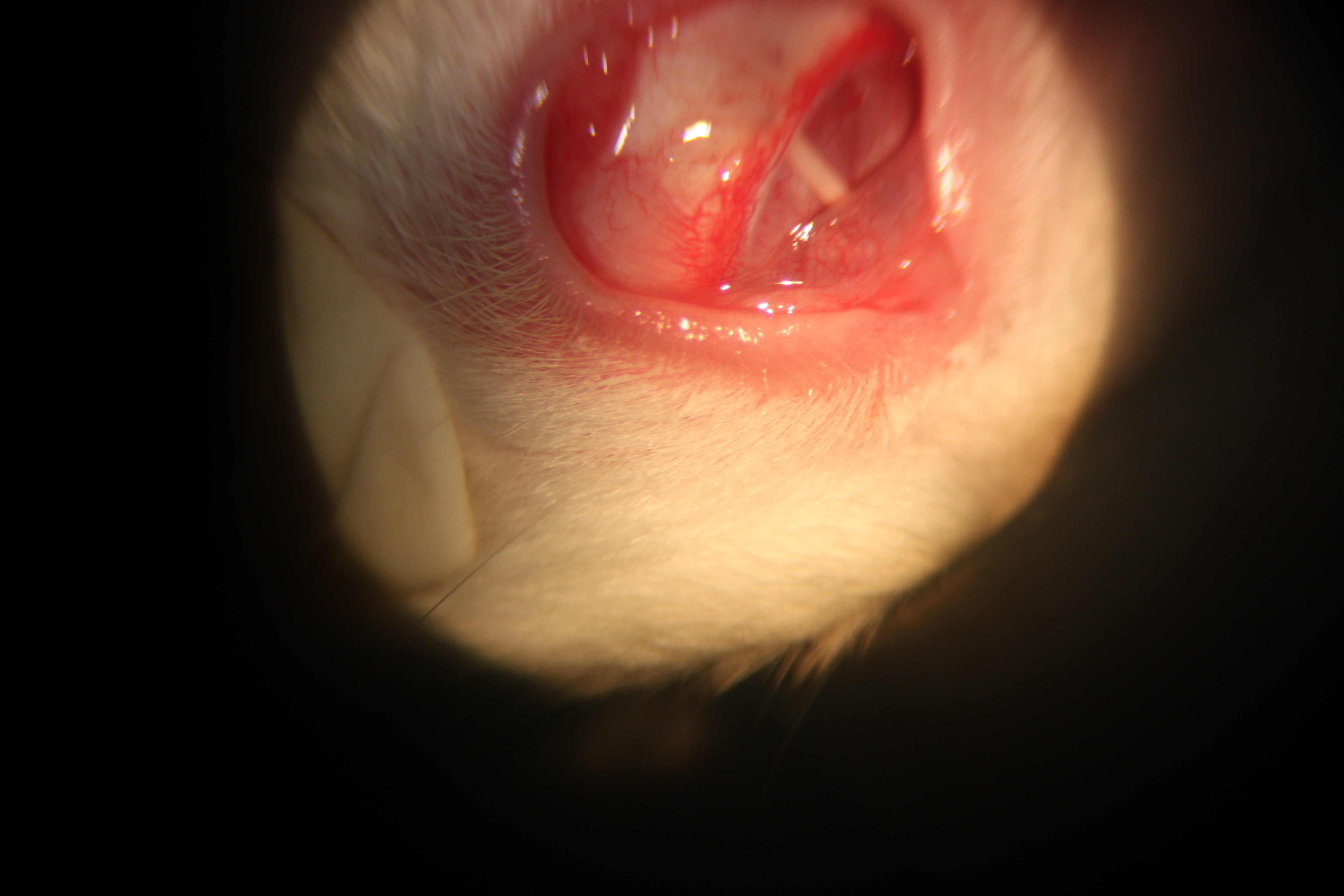

Supplement: S4 Photoset — (ZIP) [file pone.0138054.s005.zip › Multi Tx for Paper - SaratinIlomastat pics 1/IMG_2570.JPG]

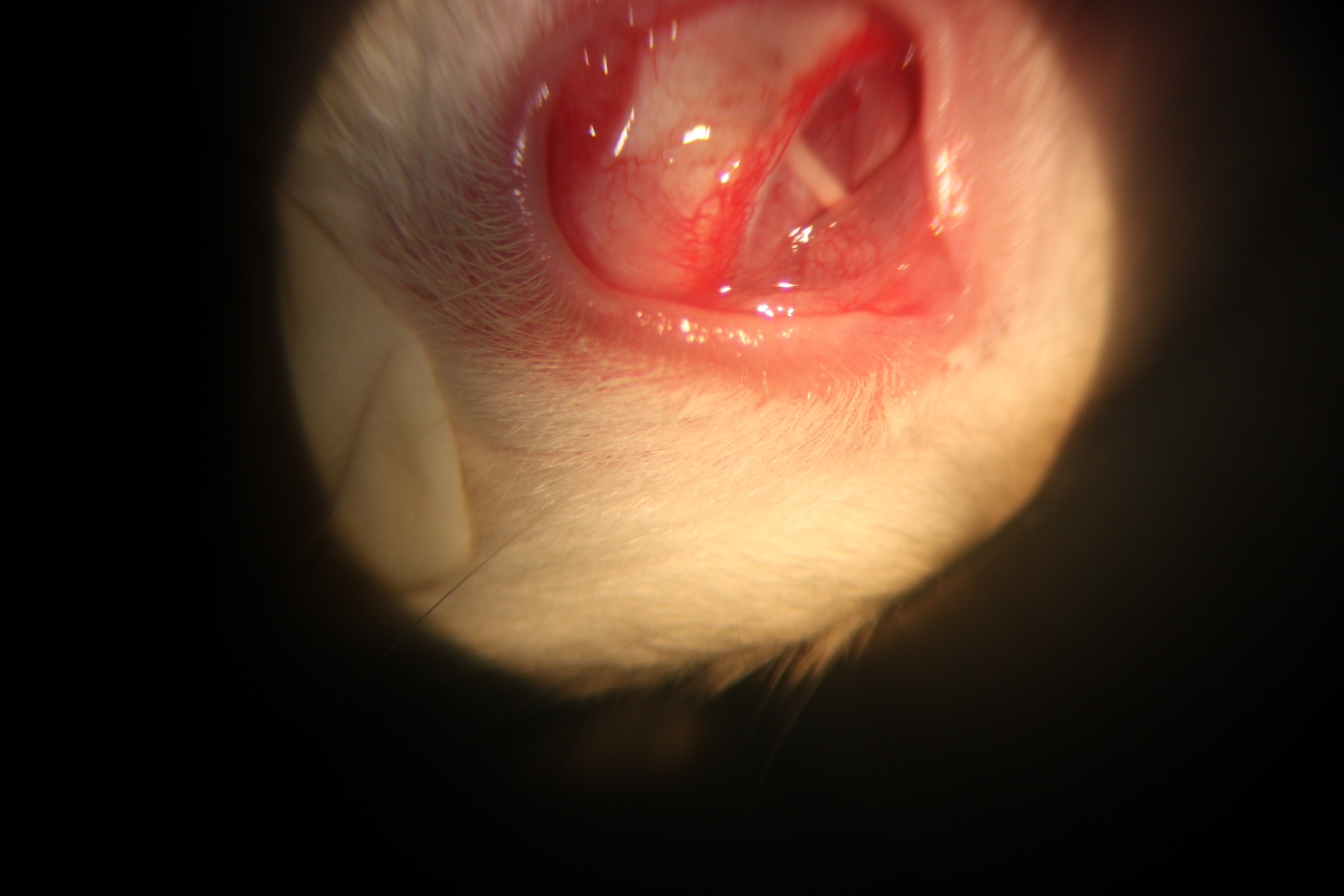

Supplement: S4 Photoset — (ZIP) [file pone.0138054.s005.zip › Multi Tx for Paper - SaratinIlomastat pics 1/IMG_2571.JPG]

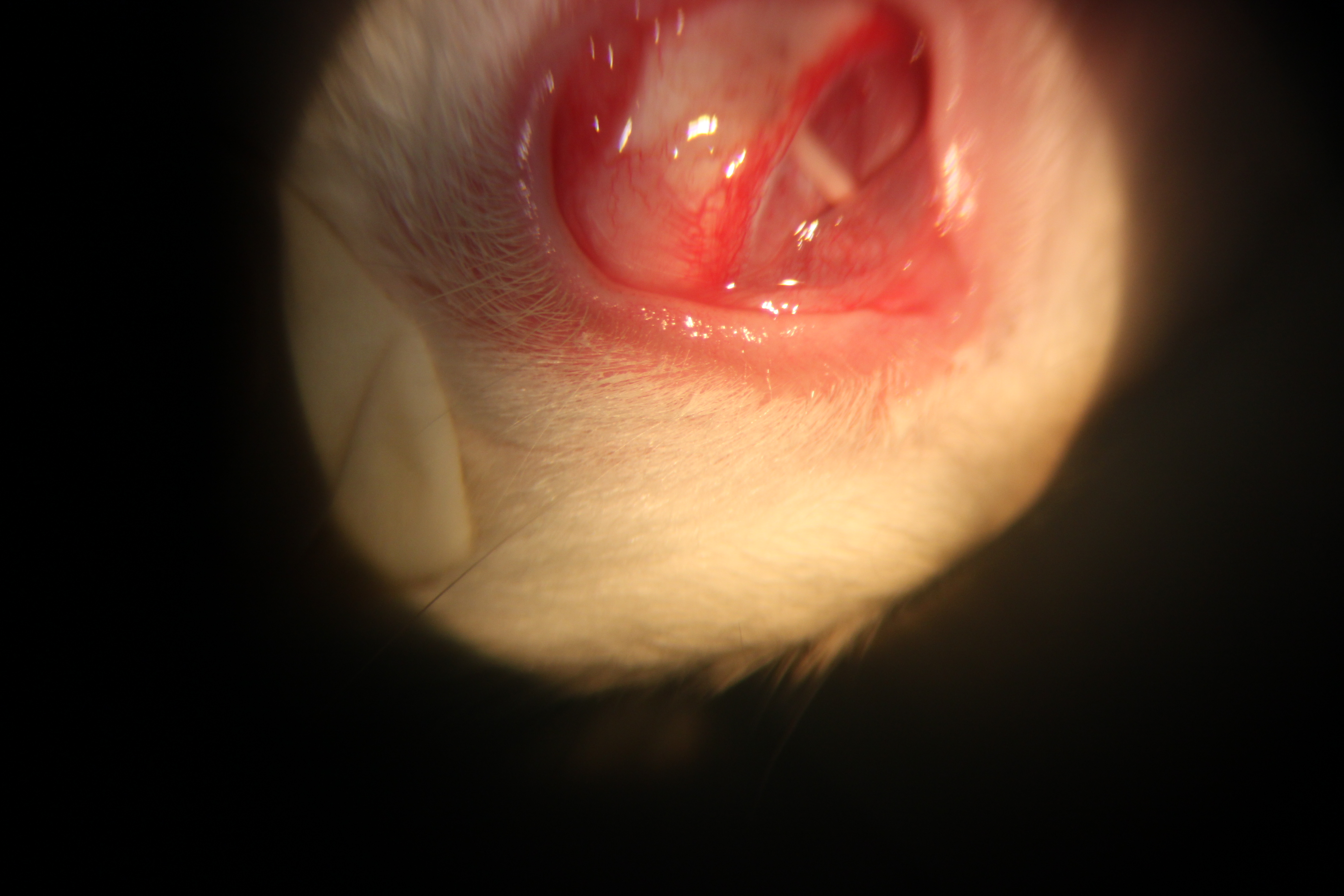

Supplement: S4 Photoset — (ZIP) [file pone.0138054.s005.zip › Multi Tx for Paper - SaratinIlomastat pics 1/IMG_2572.JPG]

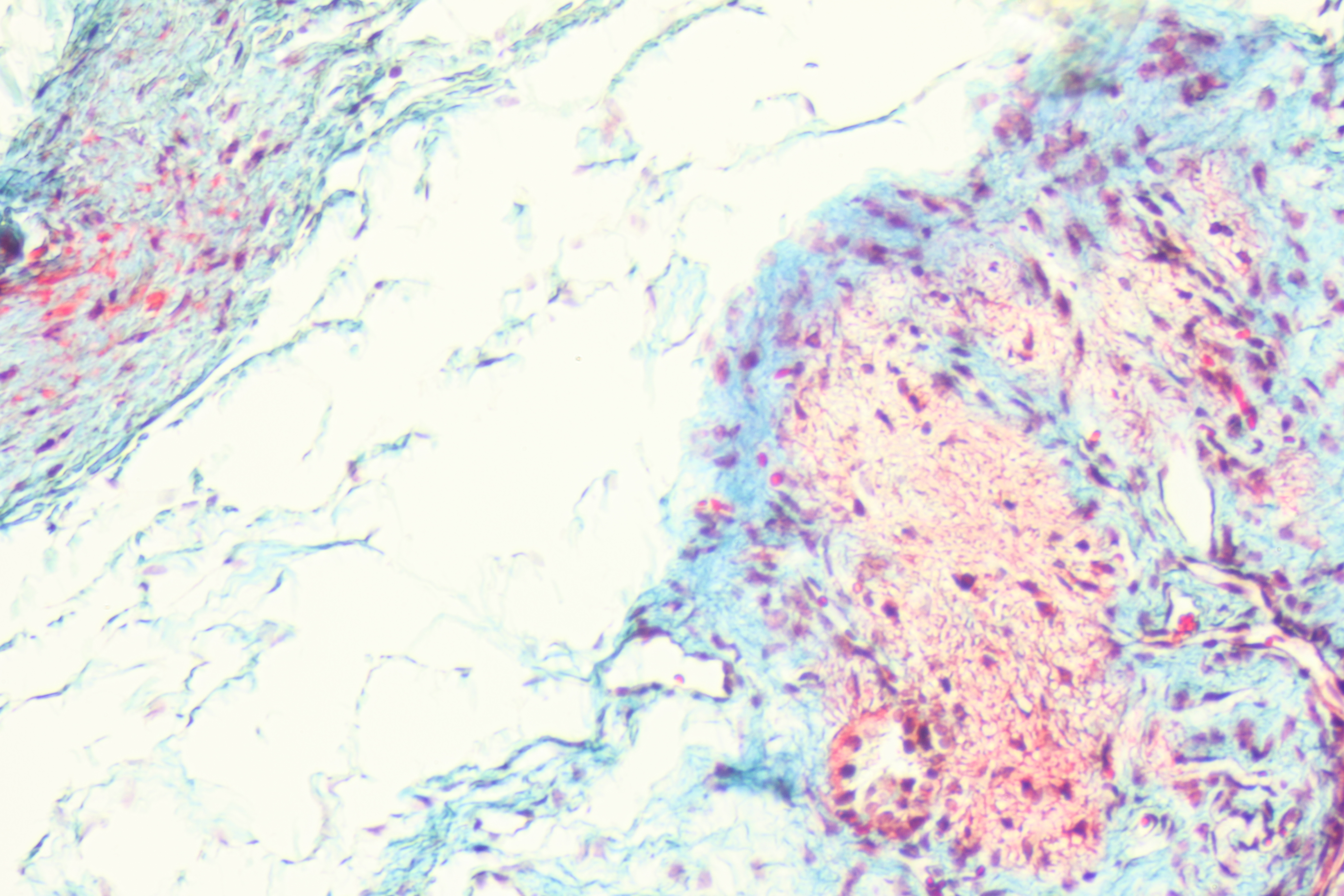

Supplement: S4 Photoset — (ZIP) [file pone.0138054.s005.zip › Multi Tx for Paper - SaratinIlomastat pics 1/IMG_6095.JPG]

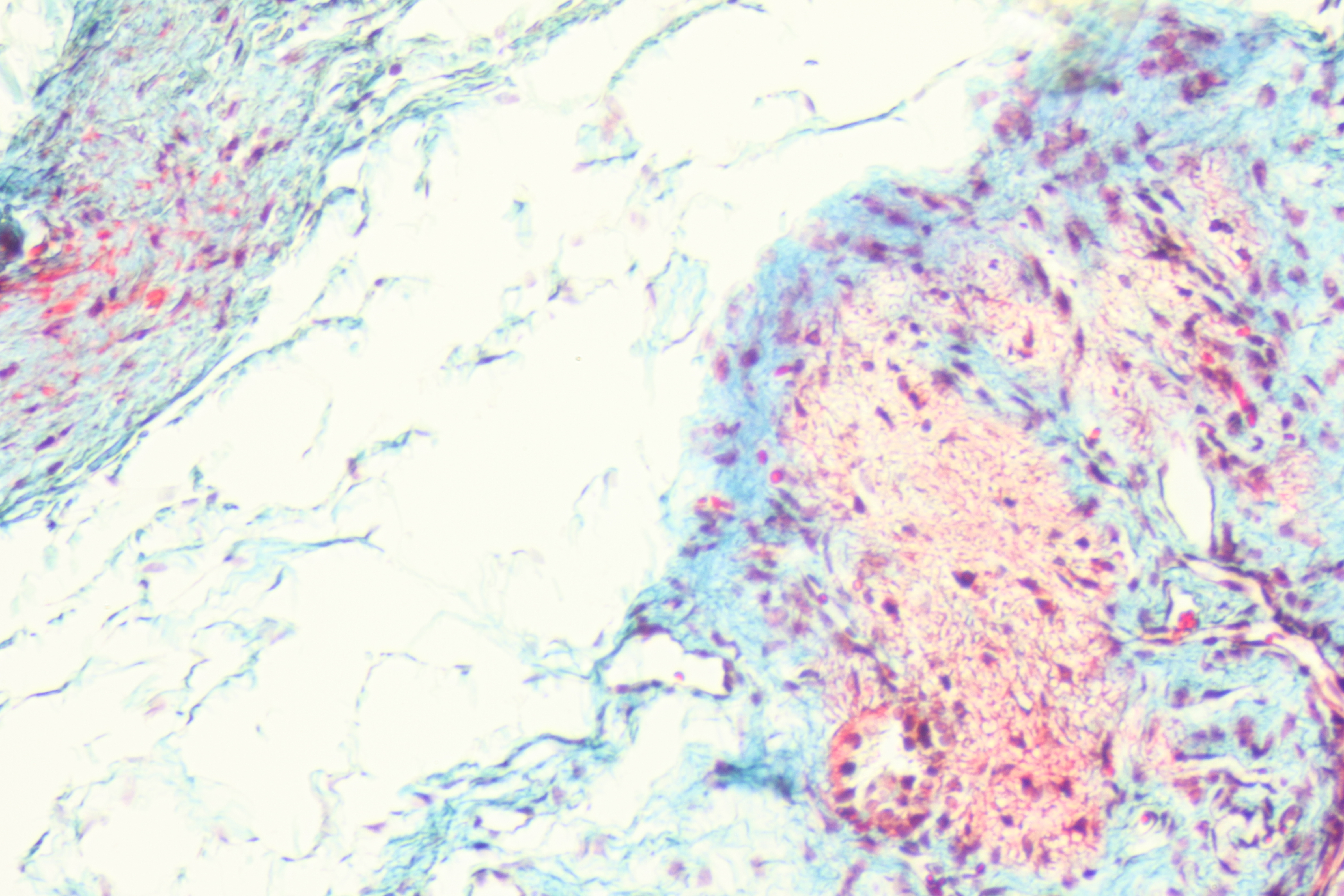

Supplement: S4 Photoset — (ZIP) [file pone.0138054.s005.zip › Multi Tx for Paper - SaratinIlomastat pics 1/IMG_6096.JPG]

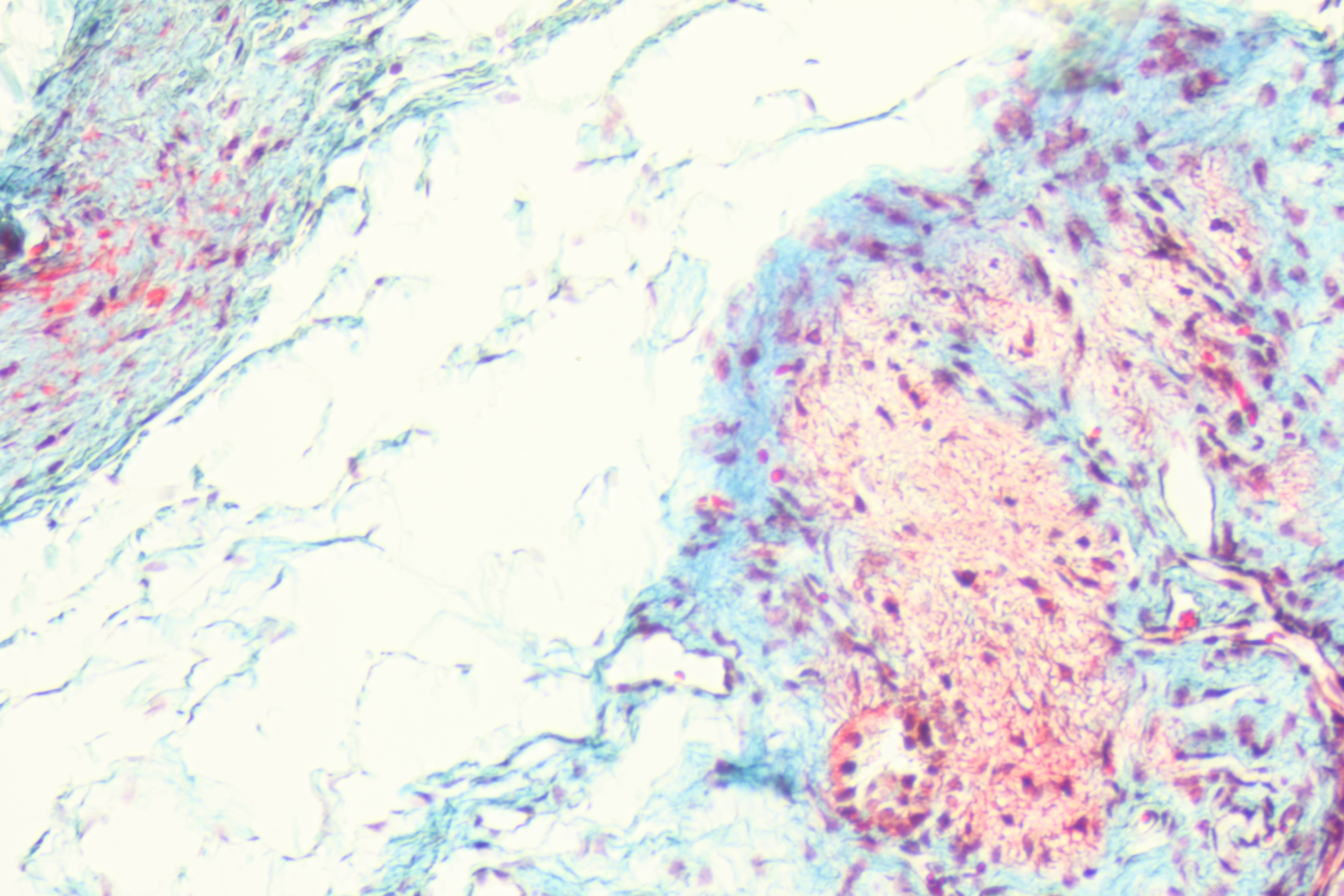

Supplement: S4 Photoset — (ZIP) [file pone.0138054.s005.zip › Multi Tx for Paper - SaratinIlomastat pics 1/IMG_6097.JPG]

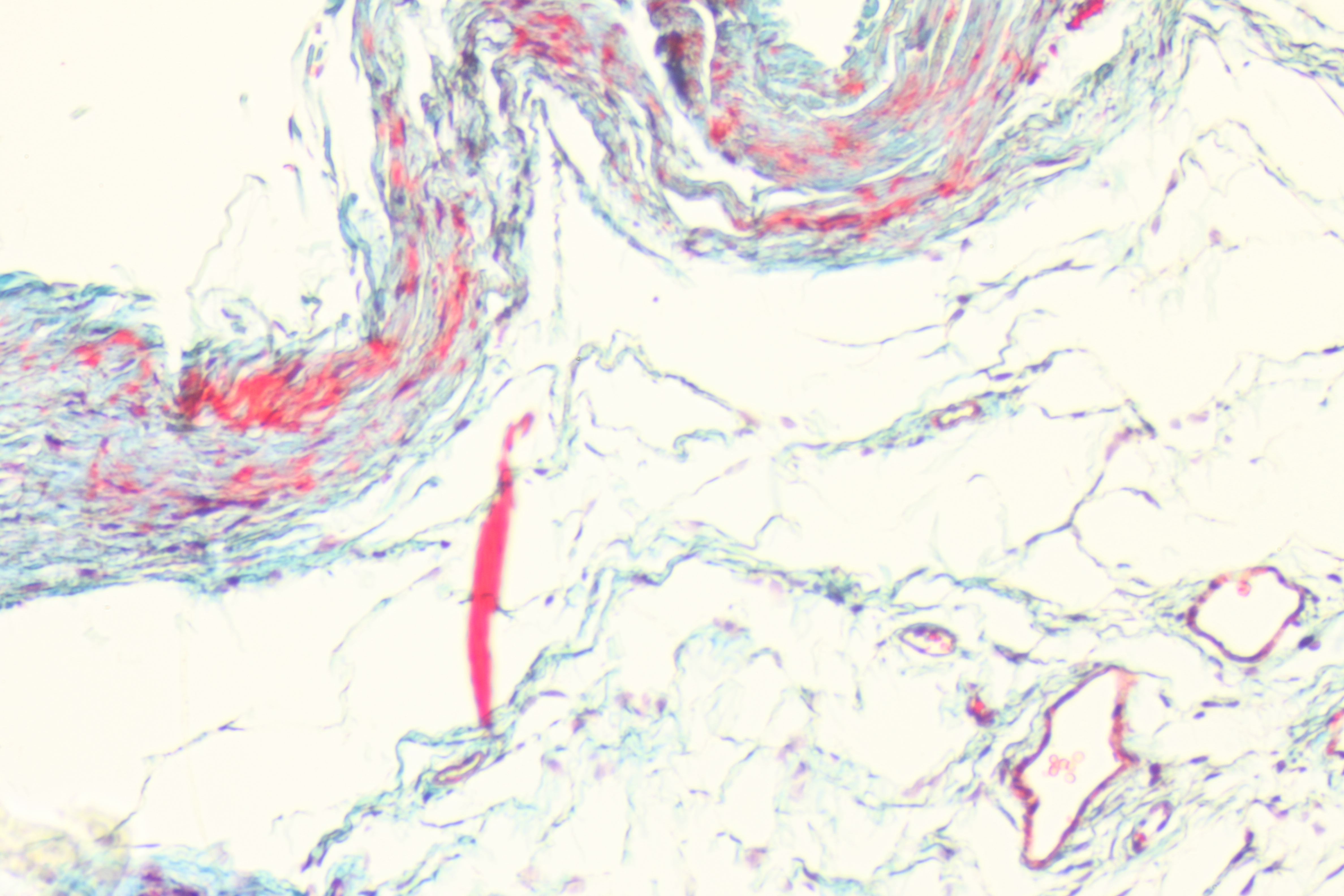

Supplement: S4 Photoset — (ZIP) [file pone.0138054.s005.zip › Multi Tx for Paper - SaratinIlomastat pics 1/IMG_6098.JPG]

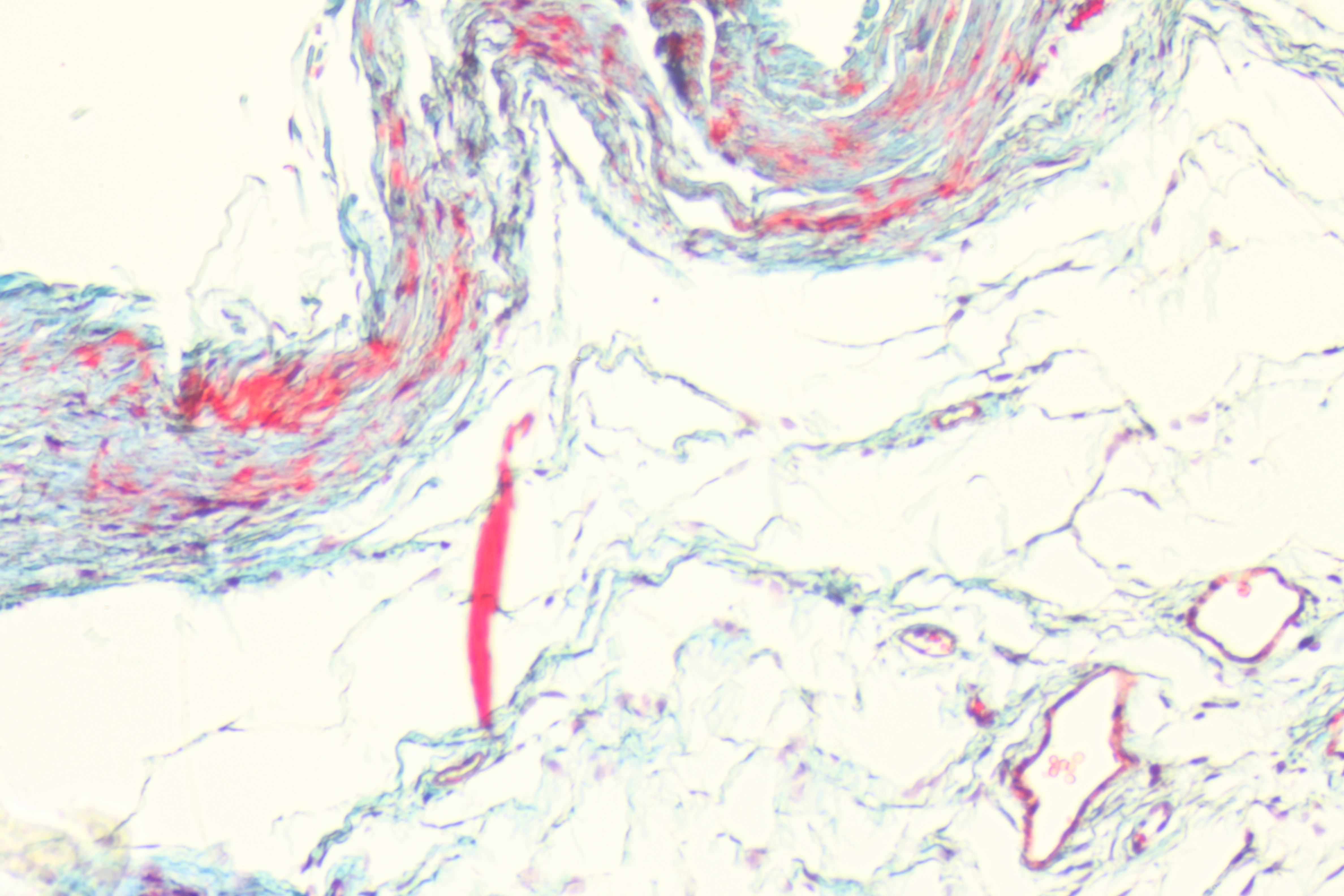

Supplement: S4 Photoset — (ZIP) [file pone.0138054.s005.zip › Multi Tx for Paper - SaratinIlomastat pics 1/IMG_6099.JPG]

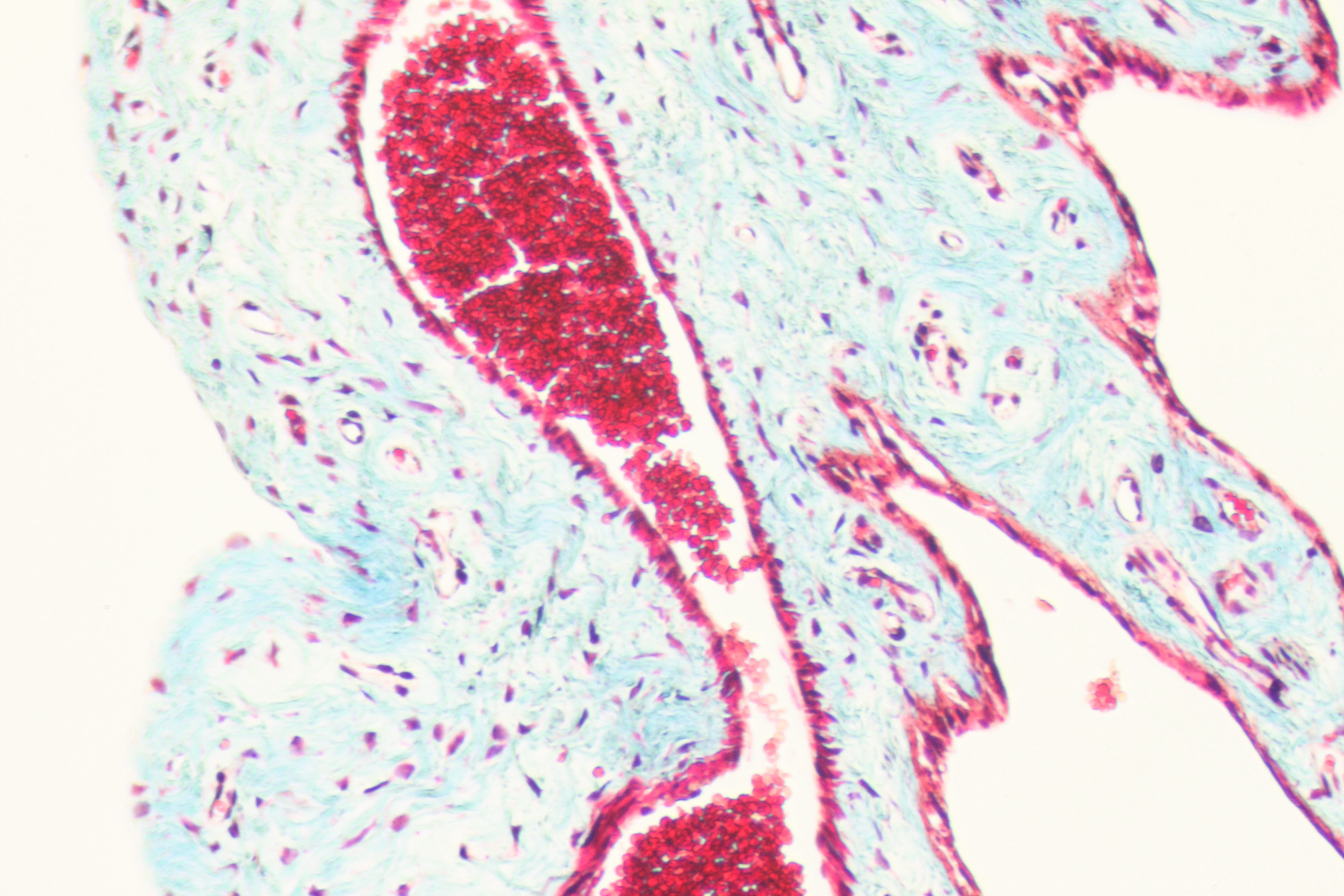

Supplement: S4 Photoset — (ZIP) [file pone.0138054.s005.zip › Multi Tx for Paper - SaratinIlomastat pics 1/IMG_6224.JPG]

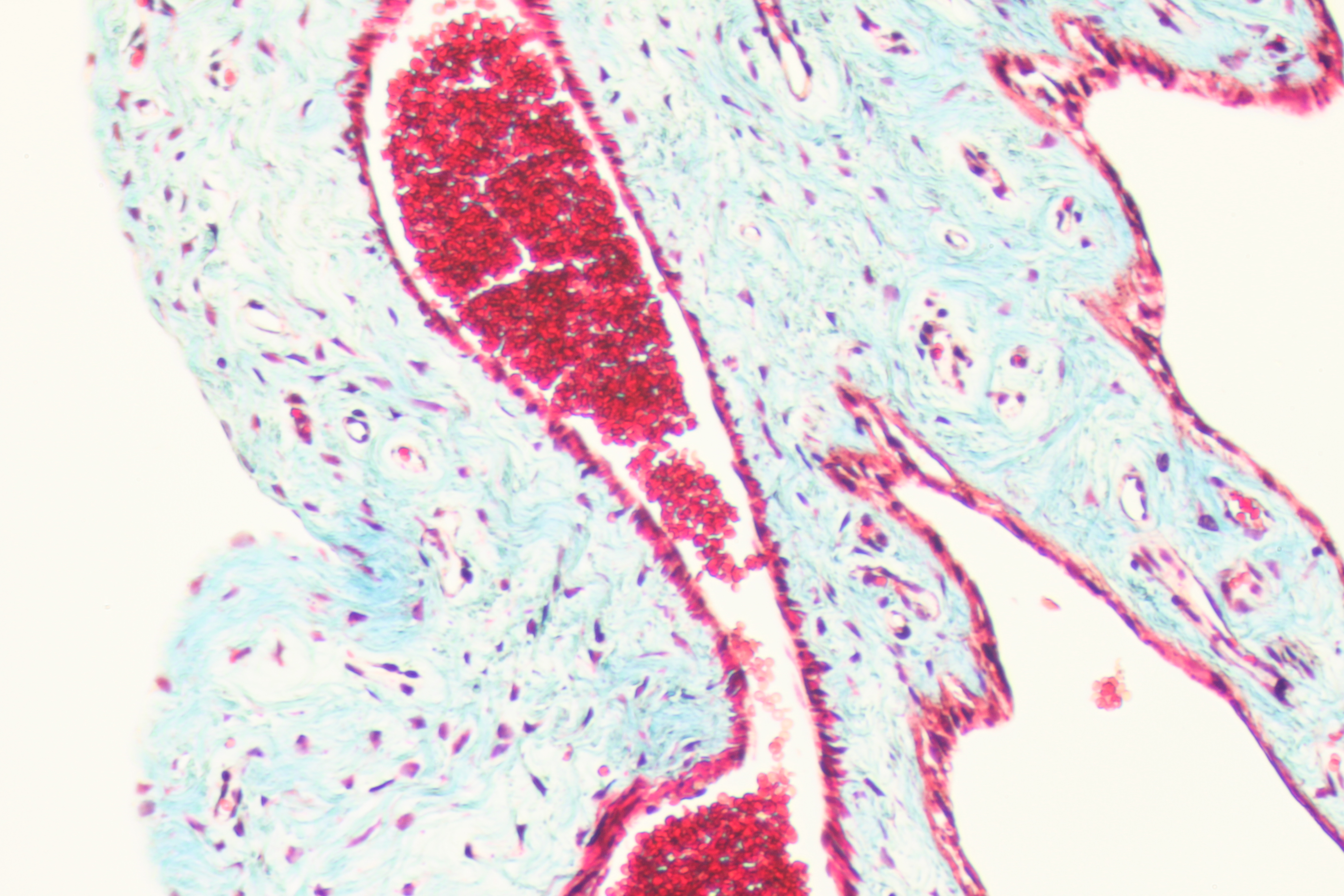

Supplement: S4 Photoset — (ZIP) [file pone.0138054.s005.zip › Multi Tx for Paper - SaratinIlomastat pics 1/IMG_6225.JPG]

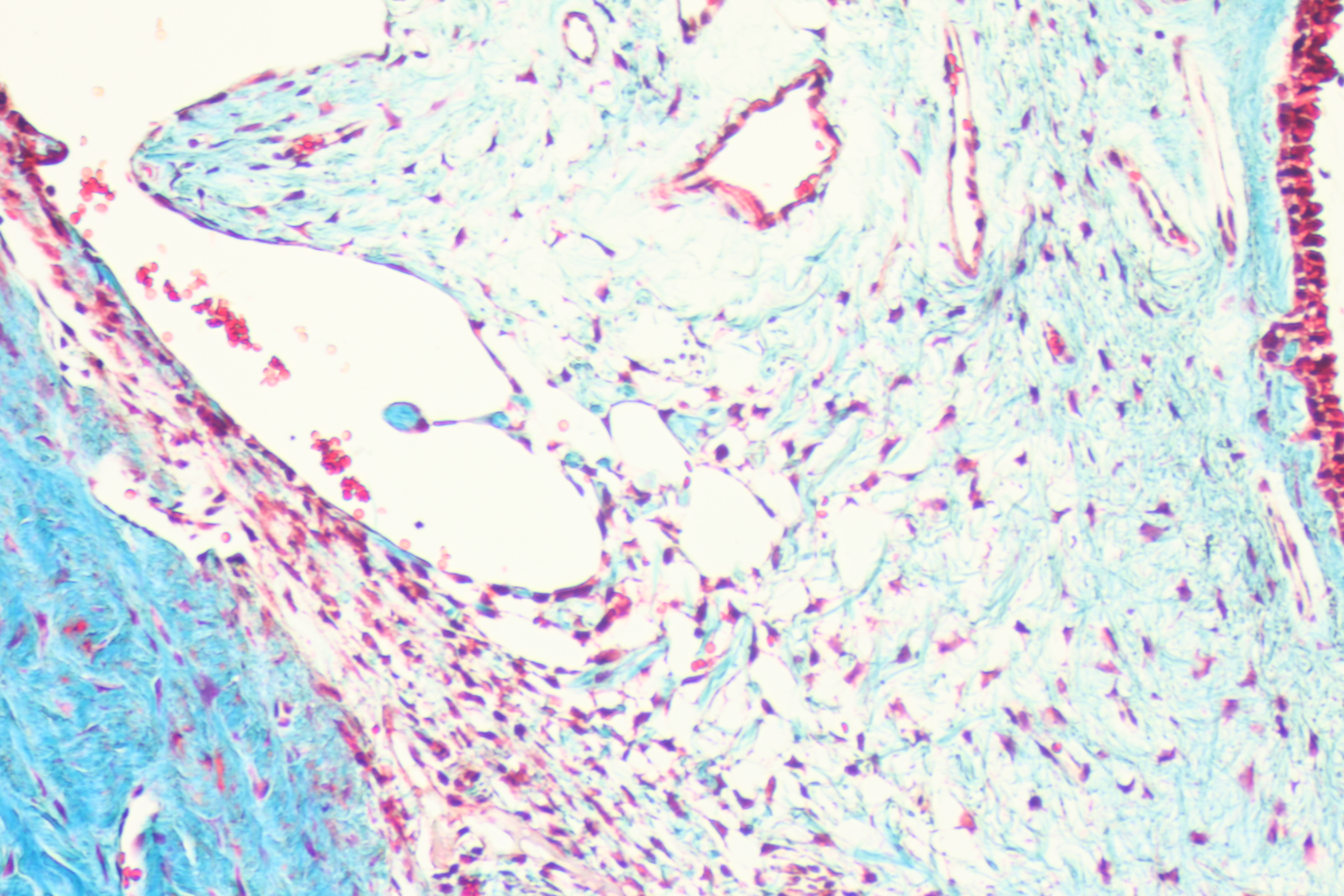

Supplement: S4 Photoset — (ZIP) [file pone.0138054.s005.zip › Multi Tx for Paper - SaratinIlomastat pics 1/IMG_6226.JPG]

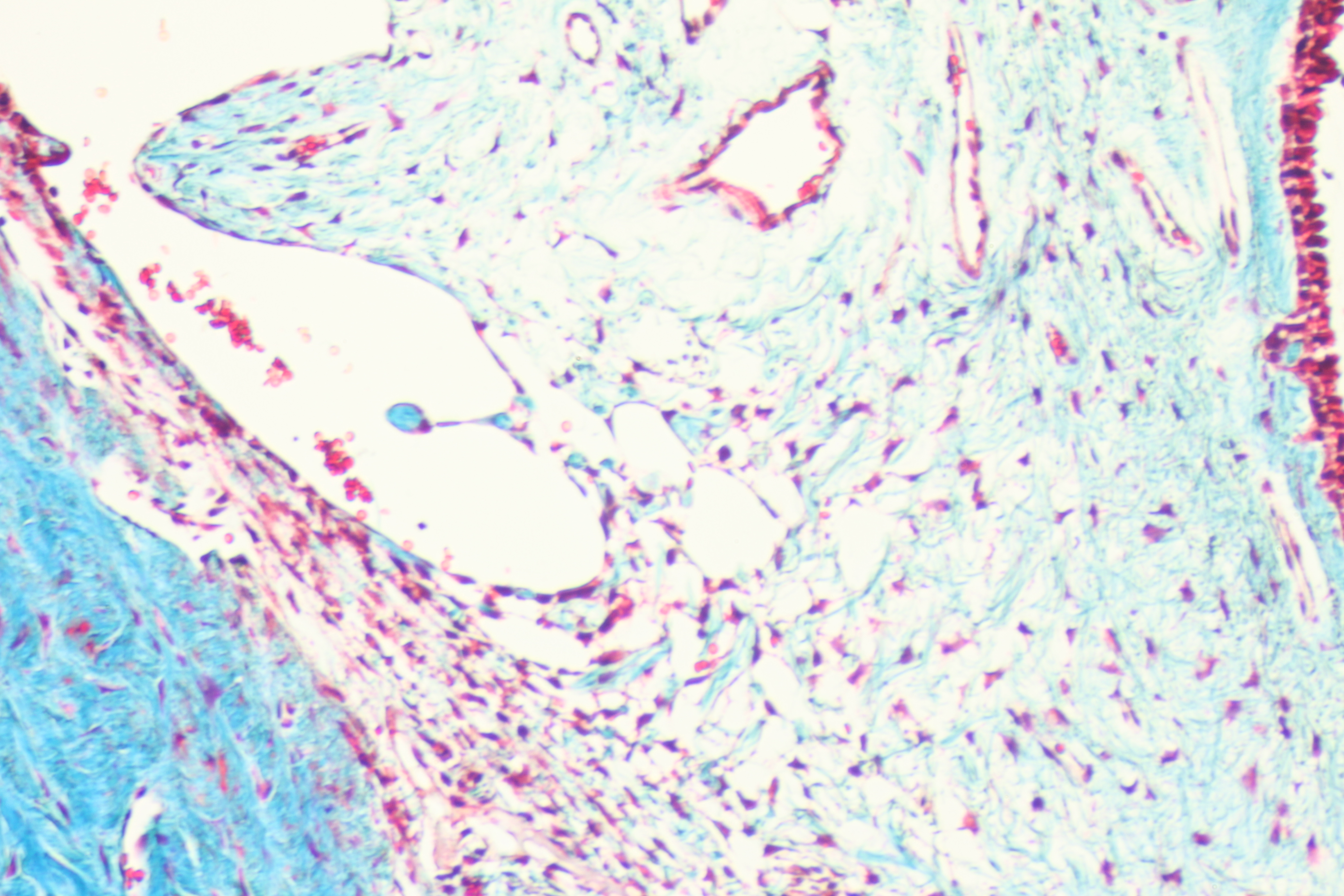

Supplement: S4 Photoset — (ZIP) [file pone.0138054.s005.zip › Multi Tx for Paper - SaratinIlomastat pics 1/IMG_6227.JPG]

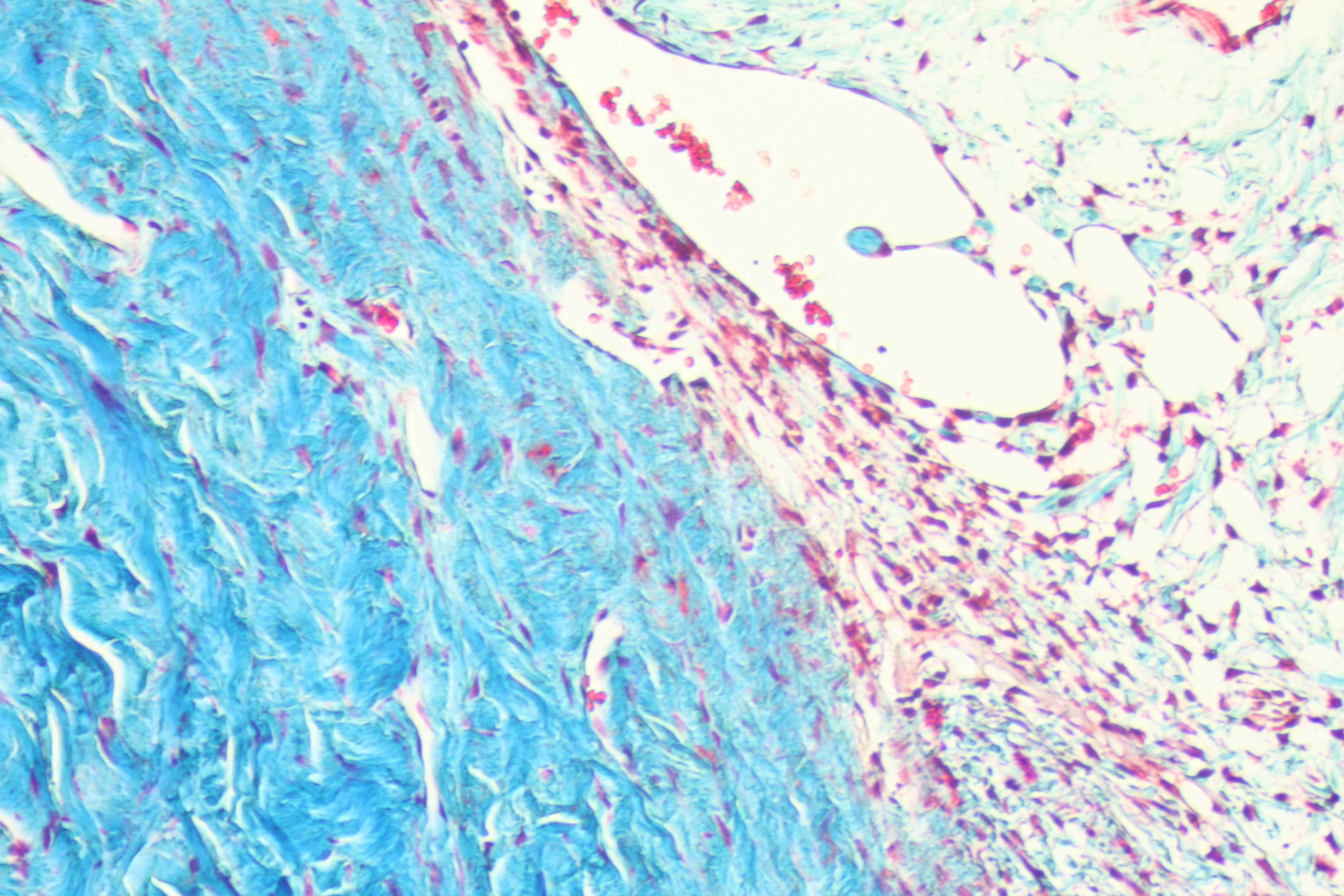

Supplement: S5 Photoset — (ZIP) [file pone.0138054.s006.zip › Multi Tx for Paper - SaratinIlomastat pics 2/IMG_6228.JPG]

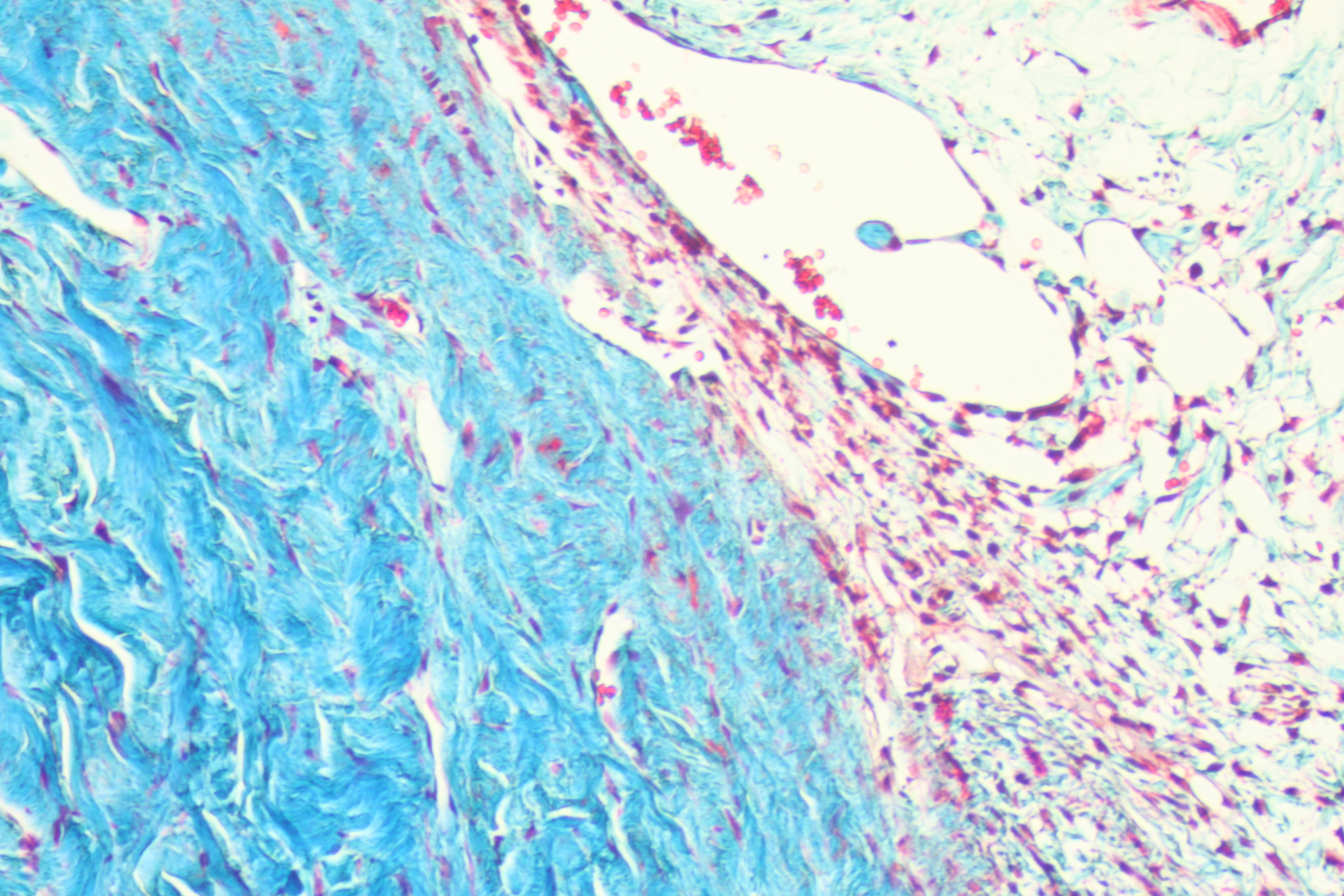

Supplement: S5 Photoset — (ZIP) [file pone.0138054.s006.zip › Multi Tx for Paper - SaratinIlomastat pics 2/IMG_6229.JPG]

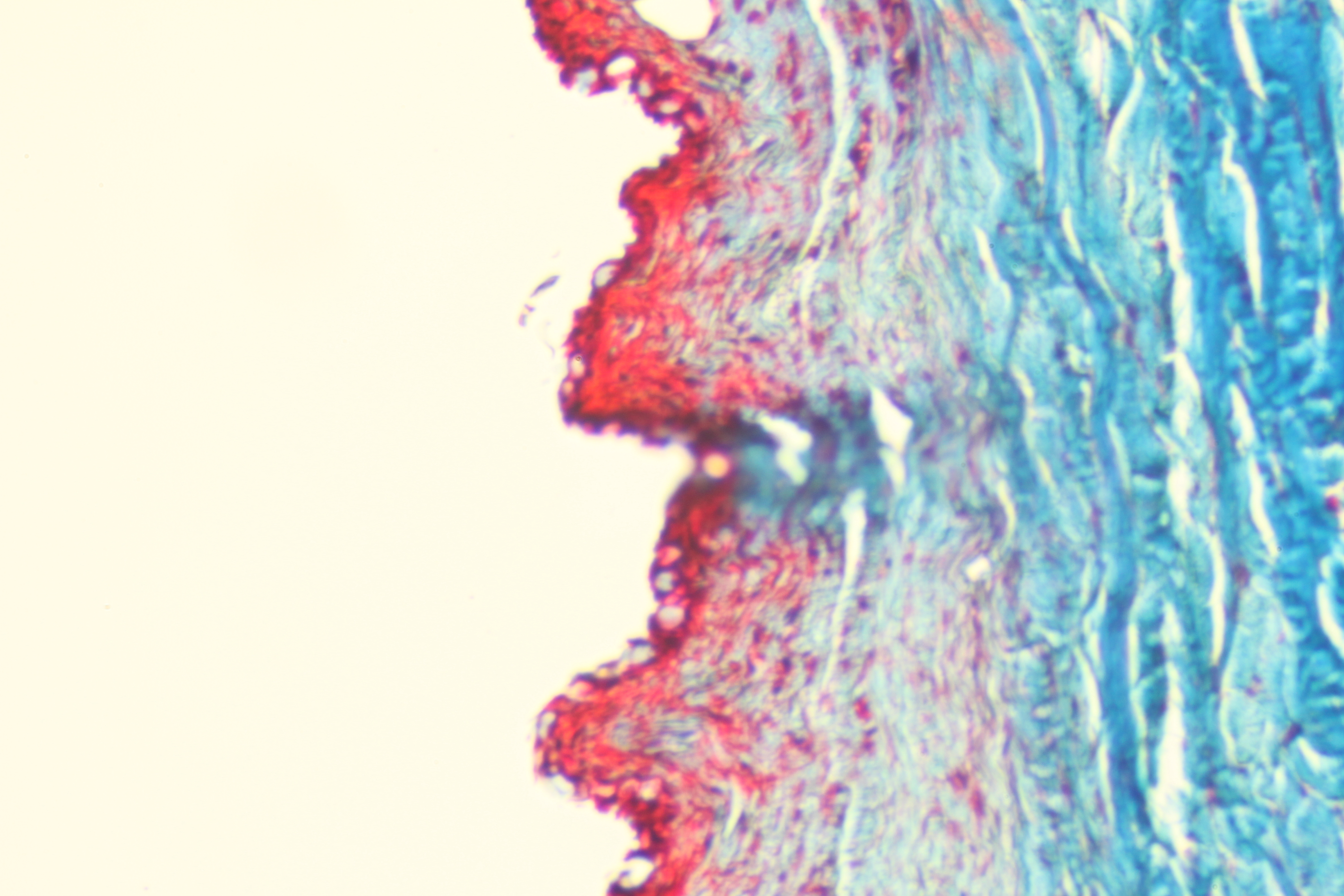

Supplement: S5 Photoset — (ZIP) [file pone.0138054.s006.zip › Multi Tx for Paper - SaratinIlomastat pics 2/IMG_6230.JPG]

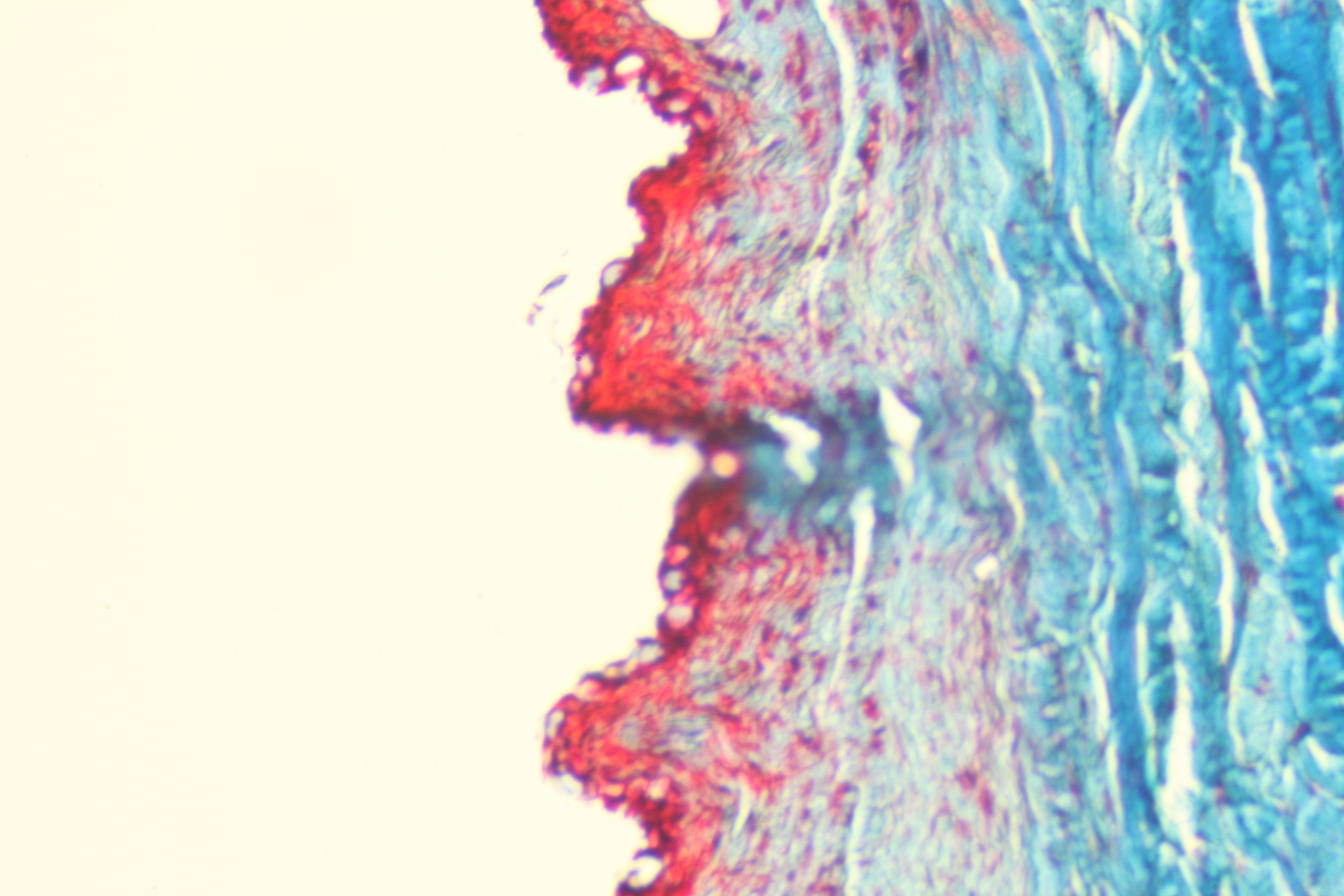

Supplement: S5 Photoset — (ZIP) [file pone.0138054.s006.zip › Multi Tx for Paper - SaratinIlomastat pics 2/IMG_6231.JPG]

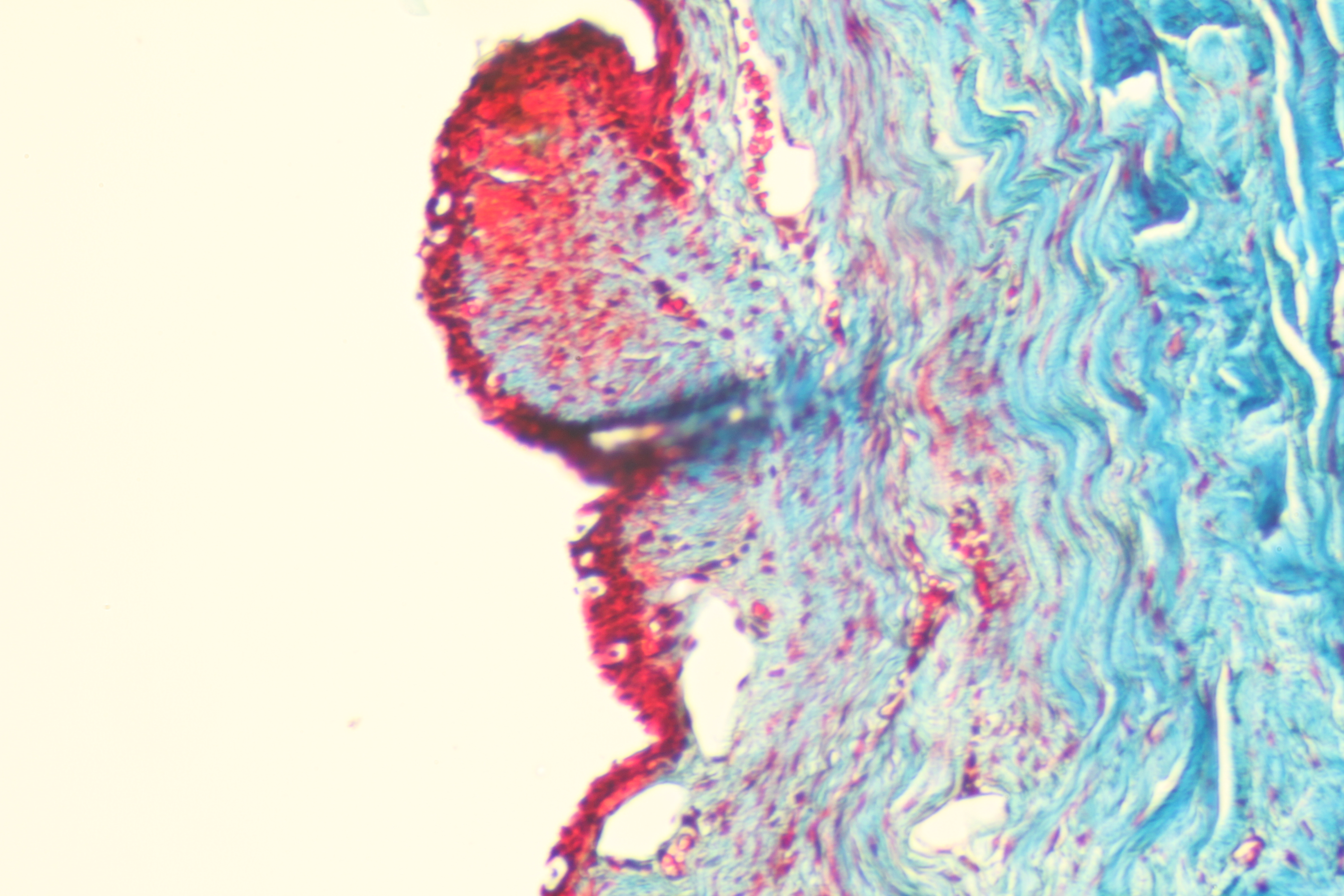

Supplement: S5 Photoset — (ZIP) [file pone.0138054.s006.zip › Multi Tx for Paper - SaratinIlomastat pics 2/IMG_6232.JPG]

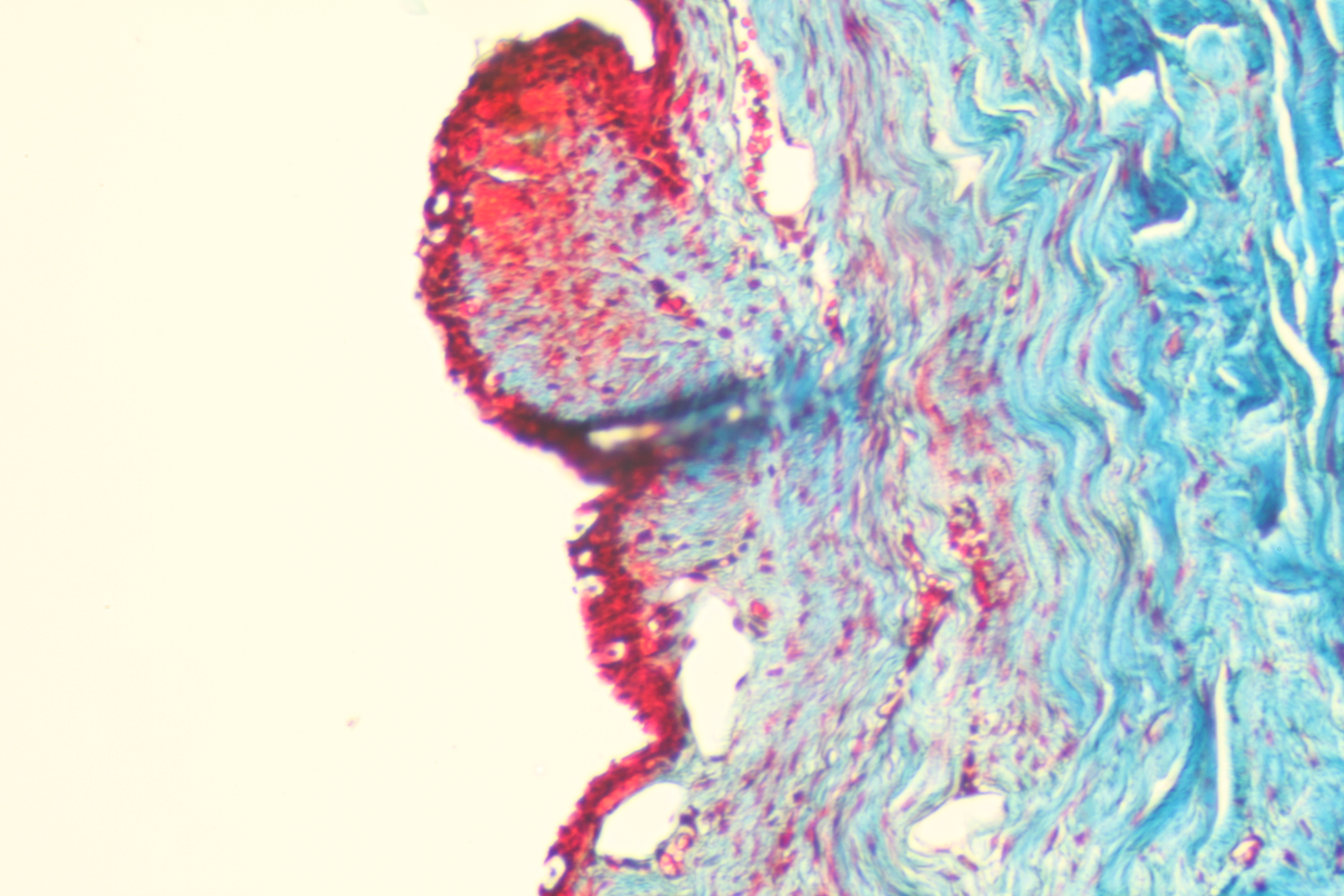

Supplement: S5 Photoset — (ZIP) [file pone.0138054.s006.zip › Multi Tx for Paper - SaratinIlomastat pics 2/IMG_6233.JPG]

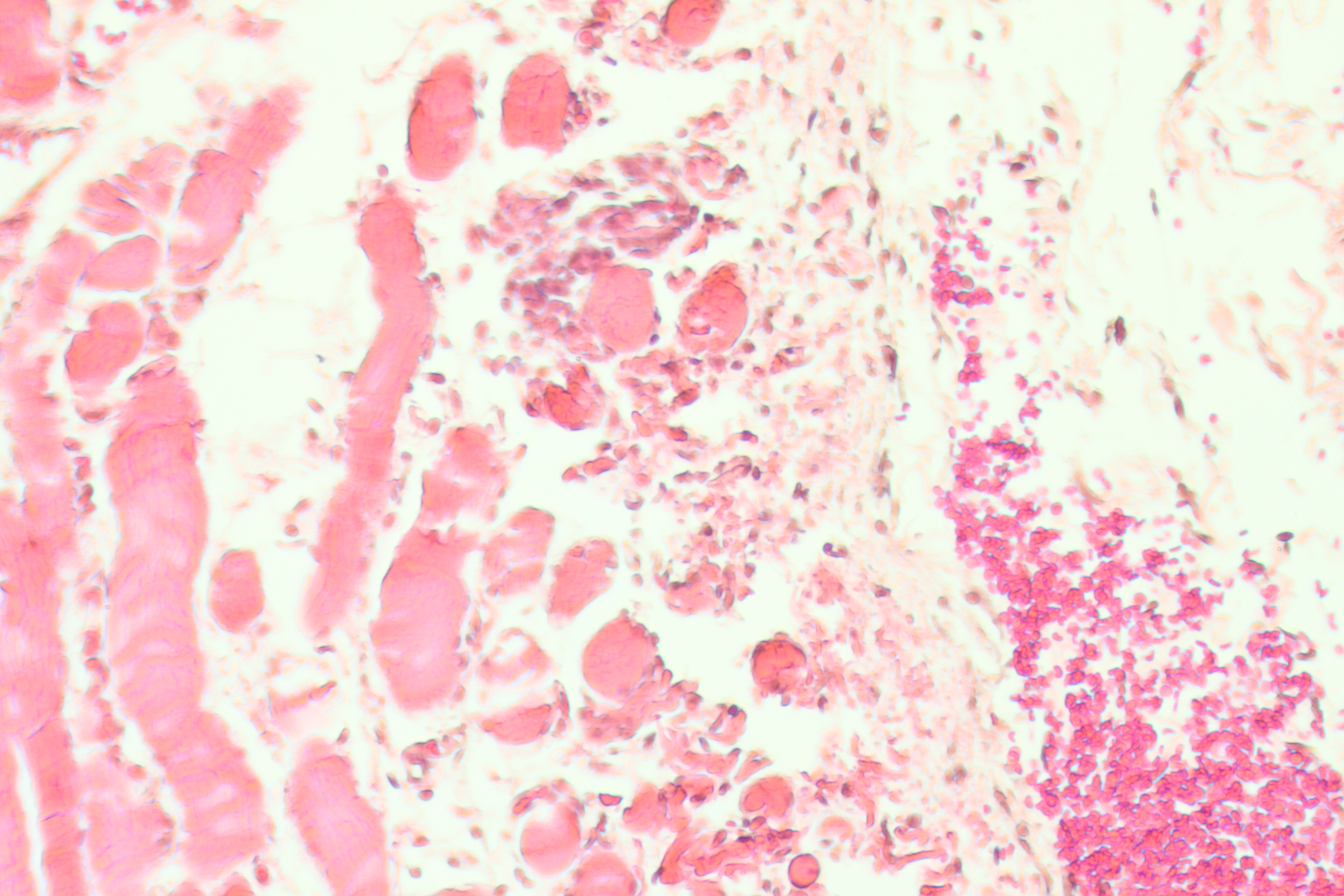

Supplement: S5 Photoset — (ZIP) [file pone.0138054.s006.zip › Multi Tx for Paper - SaratinIlomastat pics 2/IMG_6266.JPG]

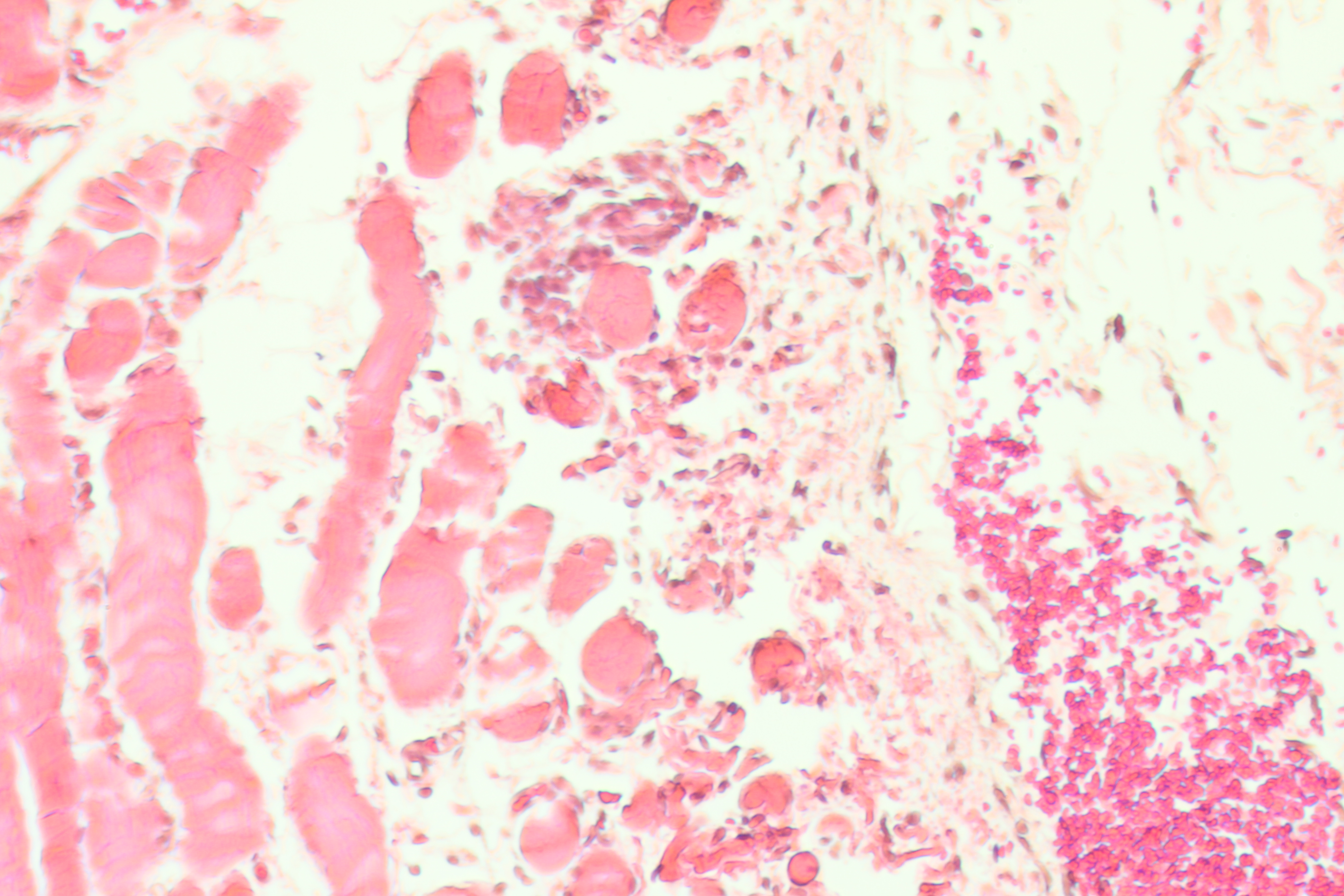

Supplement: S5 Photoset — (ZIP) [file pone.0138054.s006.zip › Multi Tx for Paper - SaratinIlomastat pics 2/IMG_6267.JPG]

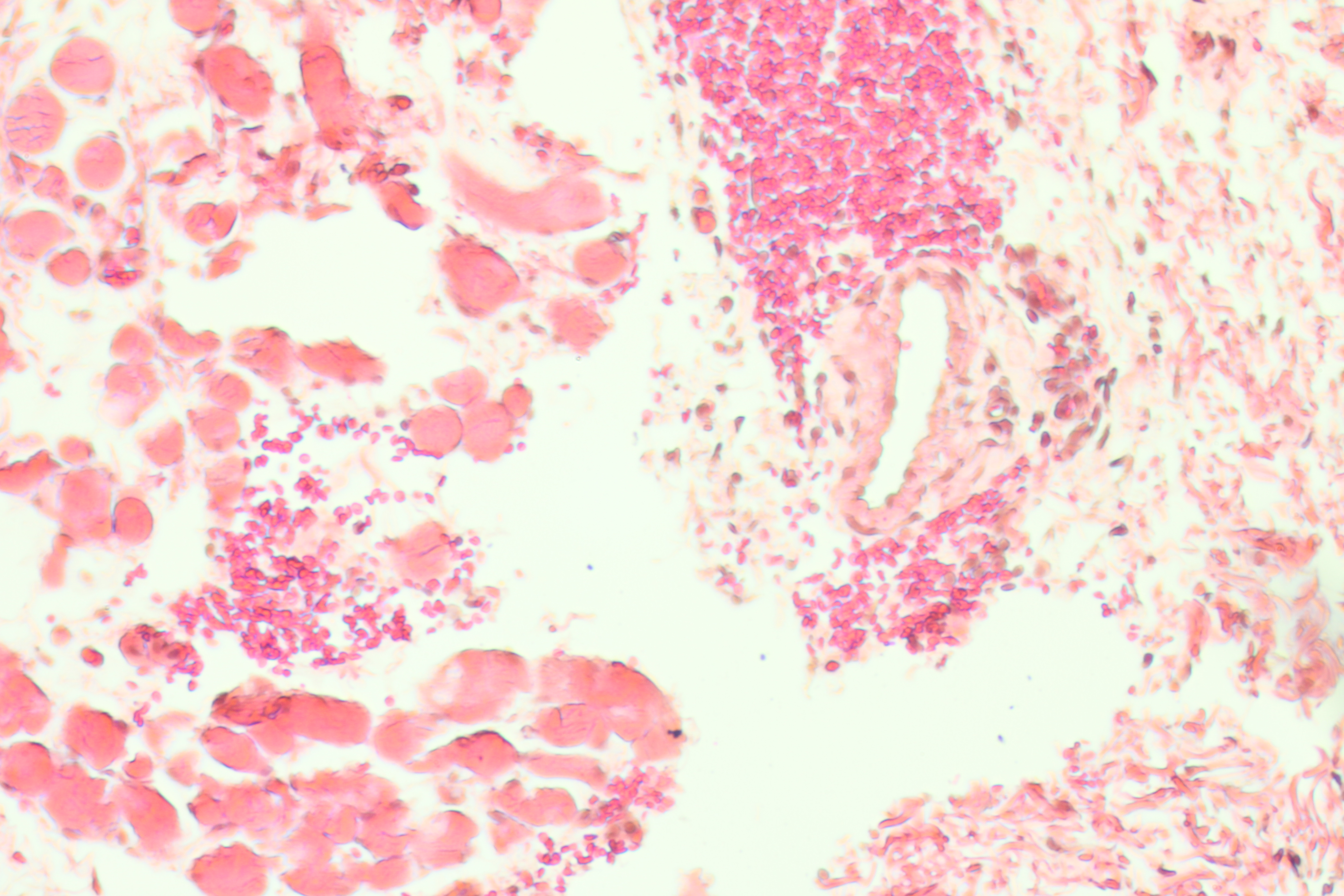

Supplement: S5 Photoset — (ZIP) [file pone.0138054.s006.zip › Multi Tx for Paper - SaratinIlomastat pics 2/IMG_6268.JPG]

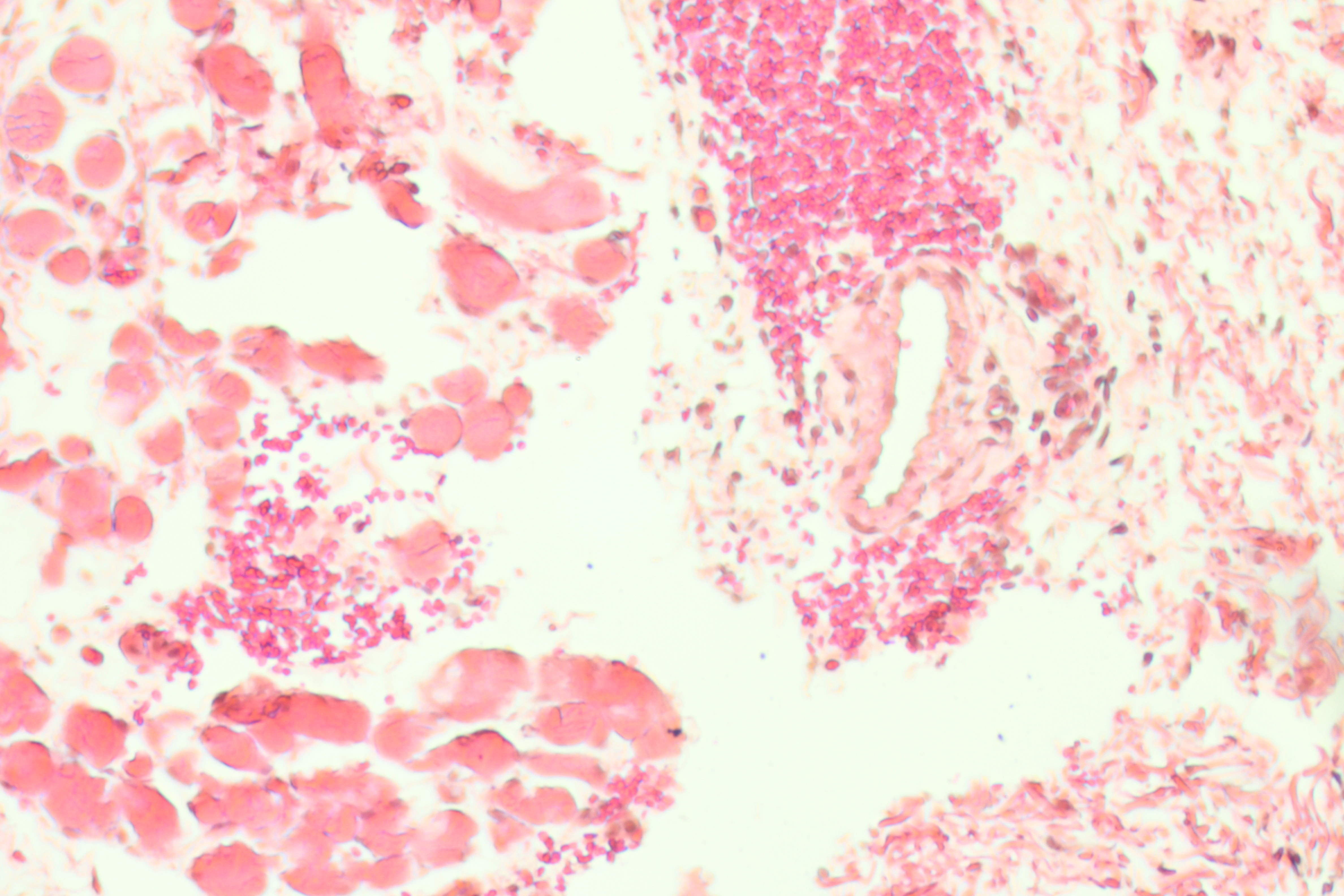

Supplement: S5 Photoset — (ZIP) [file pone.0138054.s006.zip › Multi Tx for Paper - SaratinIlomastat pics 2/IMG_6269.JPG]

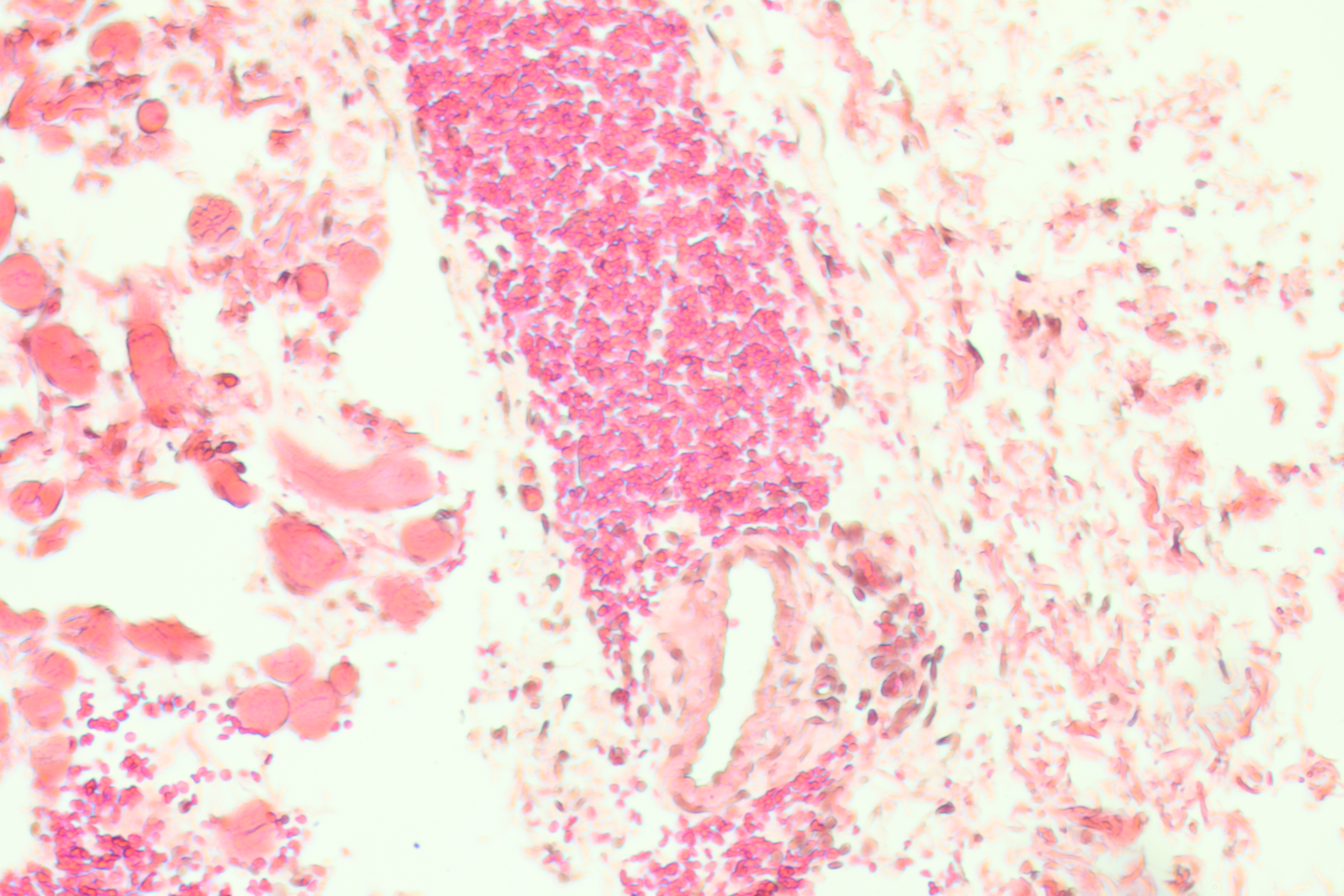

Supplement: S5 Photoset — (ZIP) [file pone.0138054.s006.zip › Multi Tx for Paper - SaratinIlomastat pics 2/IMG_6270.JPG]

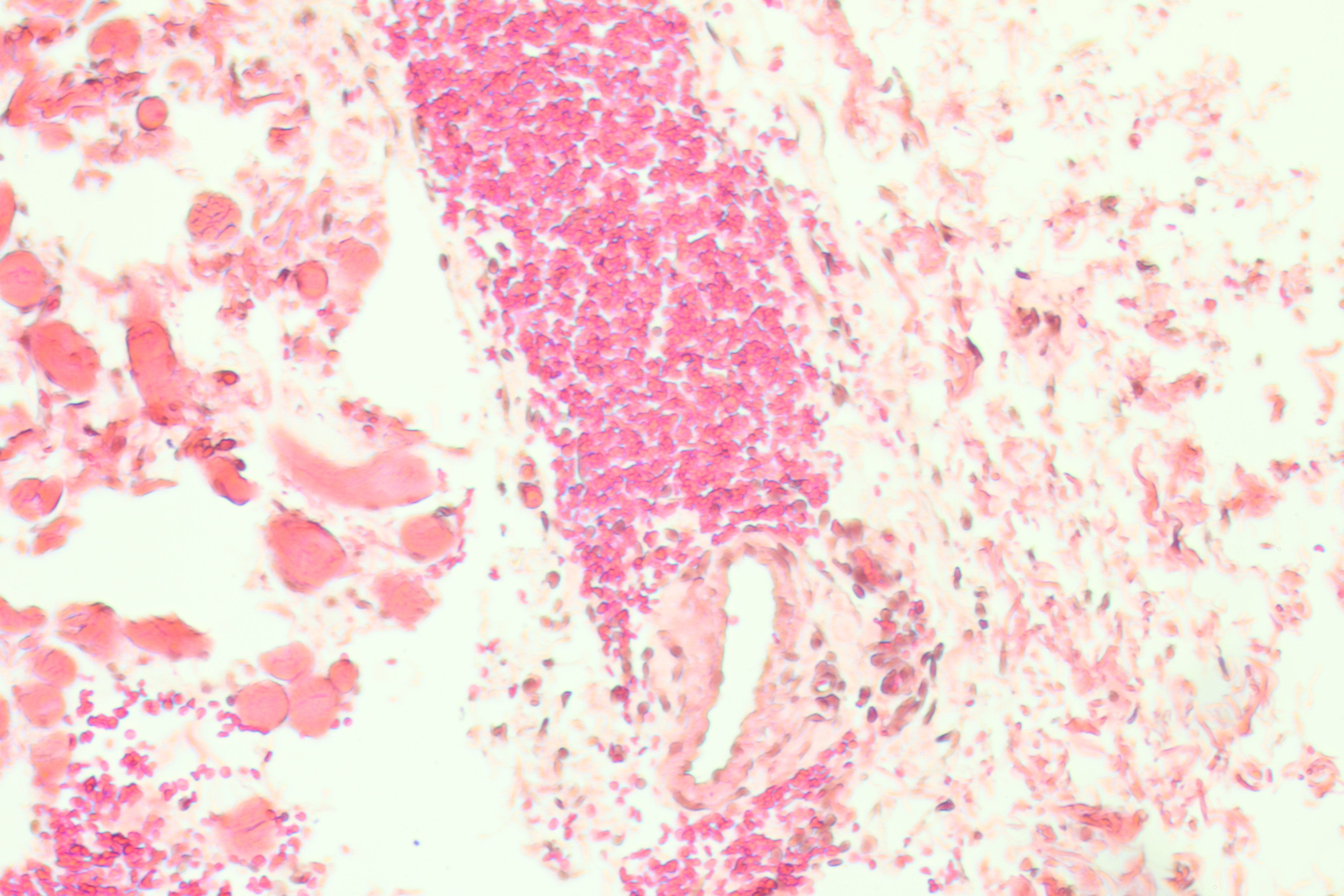

Supplement: S5 Photoset — (ZIP) [file pone.0138054.s006.zip › Multi Tx for Paper - SaratinIlomastat pics 2/IMG_6271.JPG]

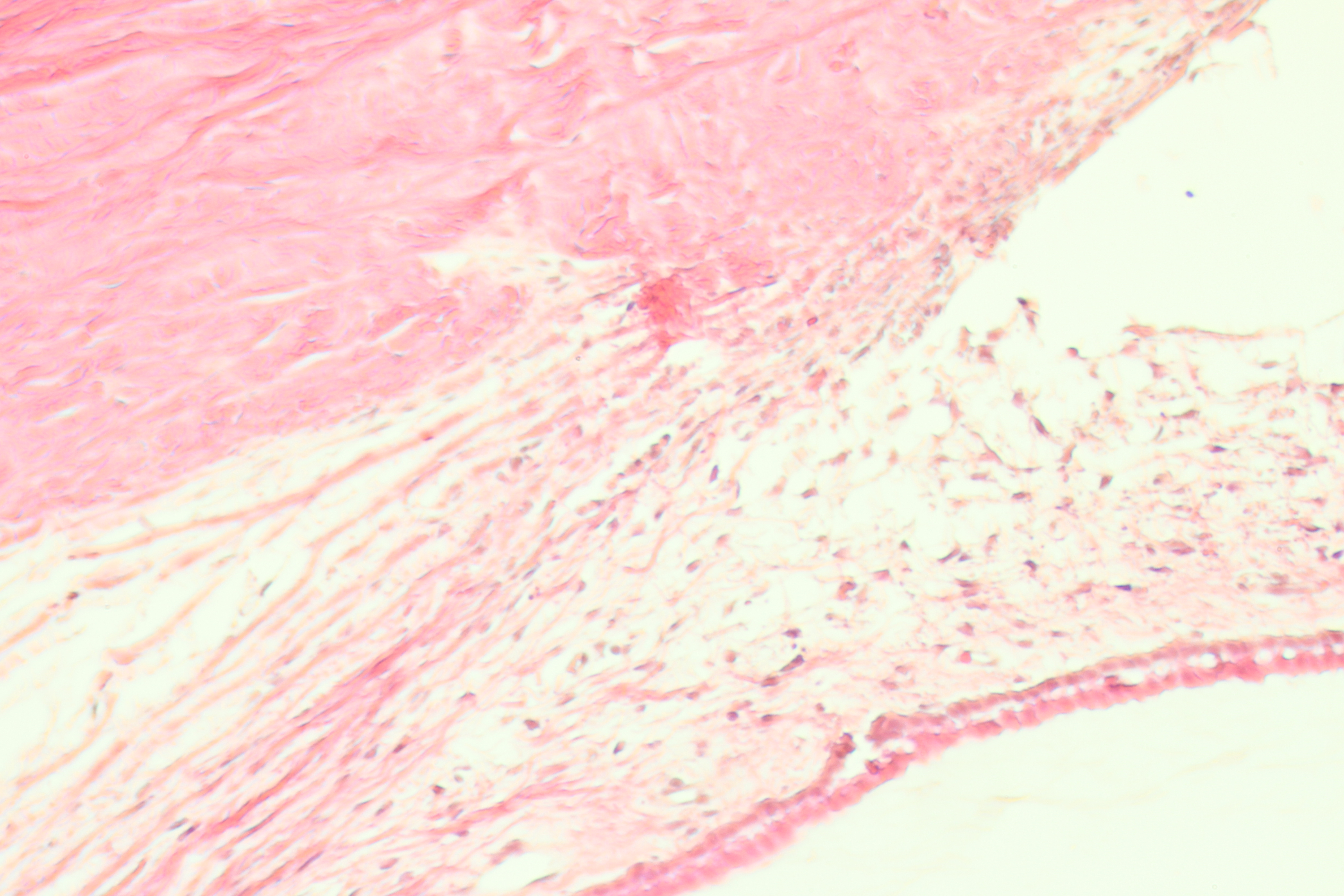

Supplement: S5 Photoset — (ZIP) [file pone.0138054.s006.zip › Multi Tx for Paper - SaratinIlomastat pics 2/IMG_6272.JPG]

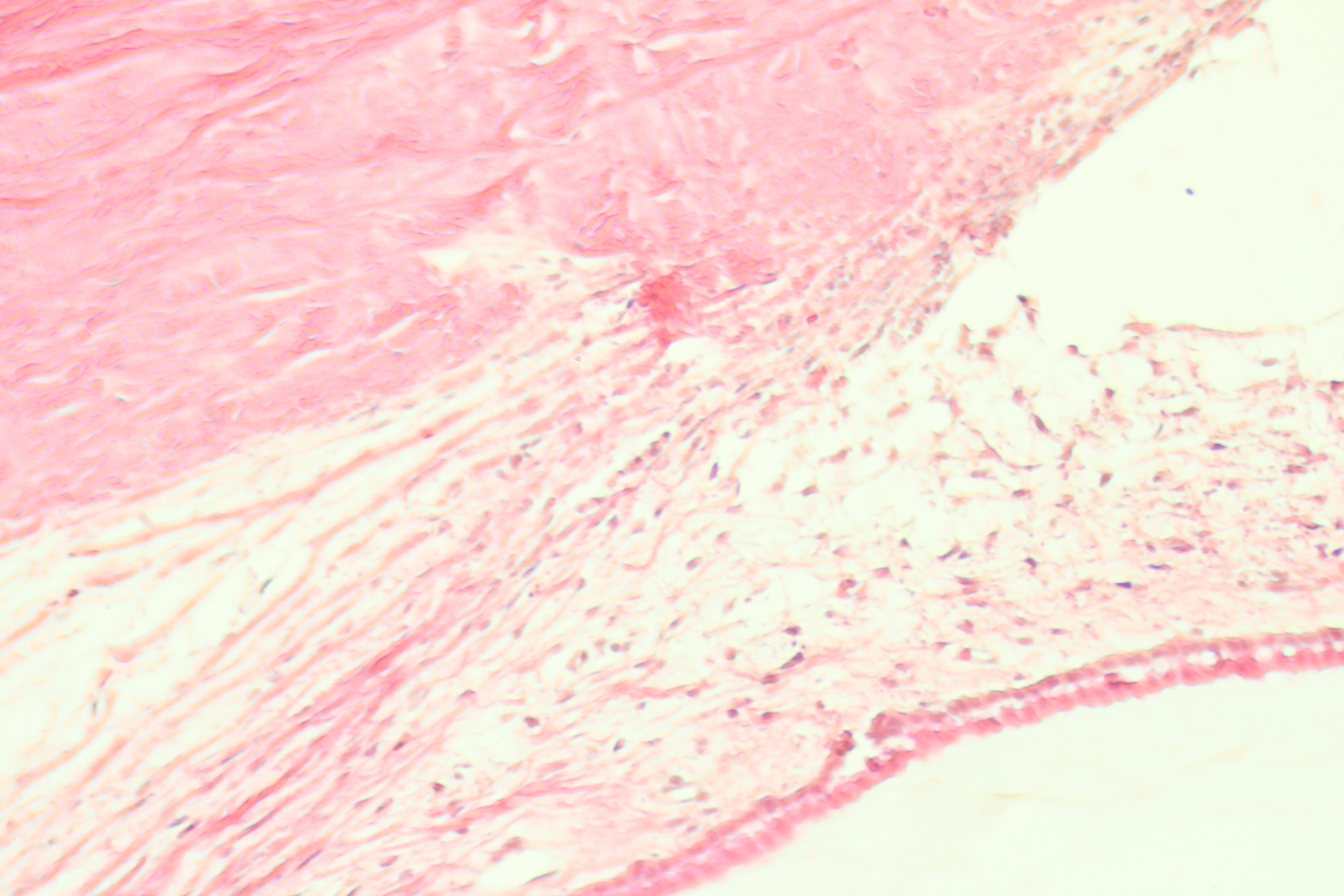

Supplement: S5 Photoset — (ZIP) [file pone.0138054.s006.zip › Multi Tx for Paper - SaratinIlomastat pics 2/IMG_6273.JPG]

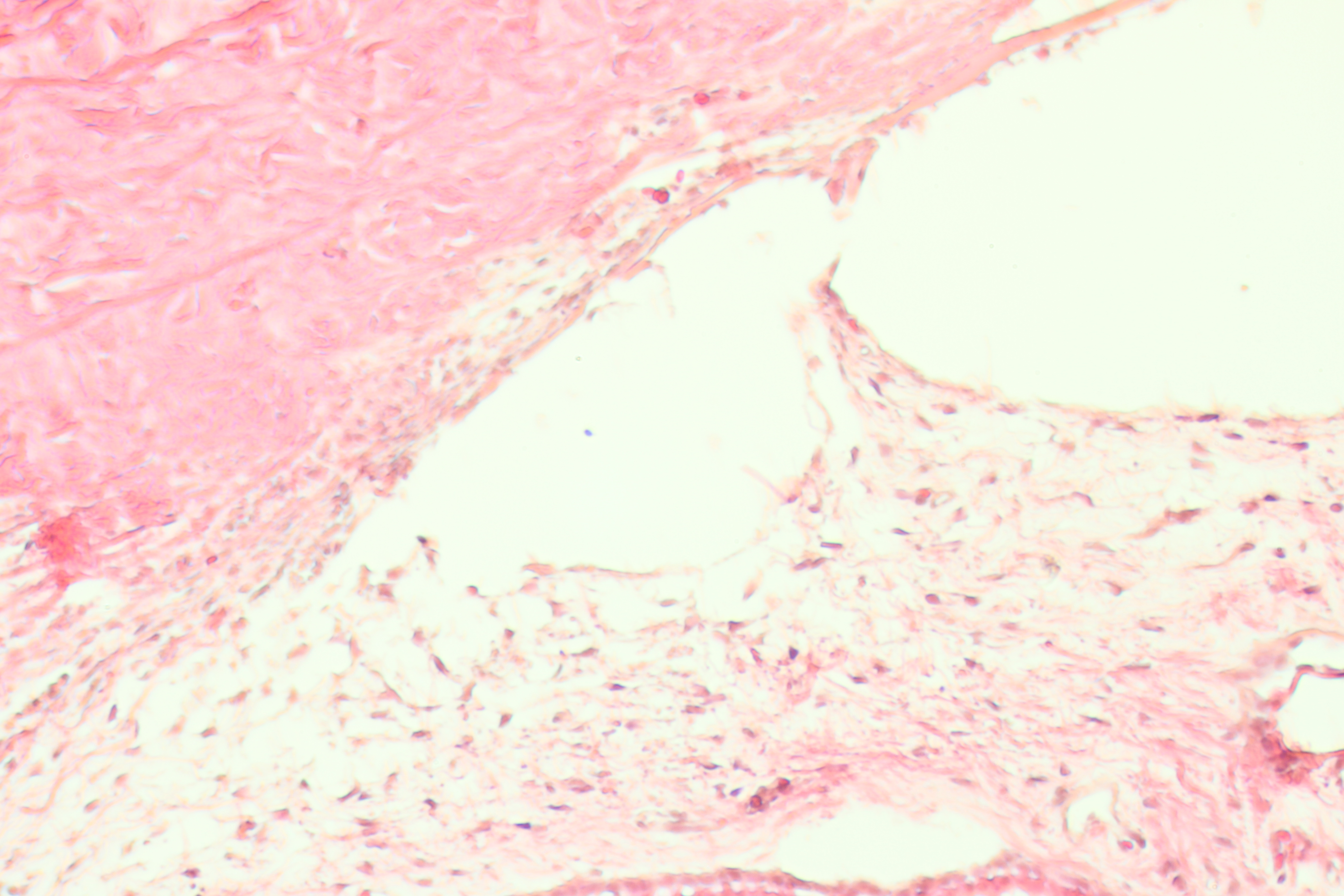

Supplement: S5 Photoset — (ZIP) [file pone.0138054.s006.zip › Multi Tx for Paper - SaratinIlomastat pics 2/IMG_6274.JPG]

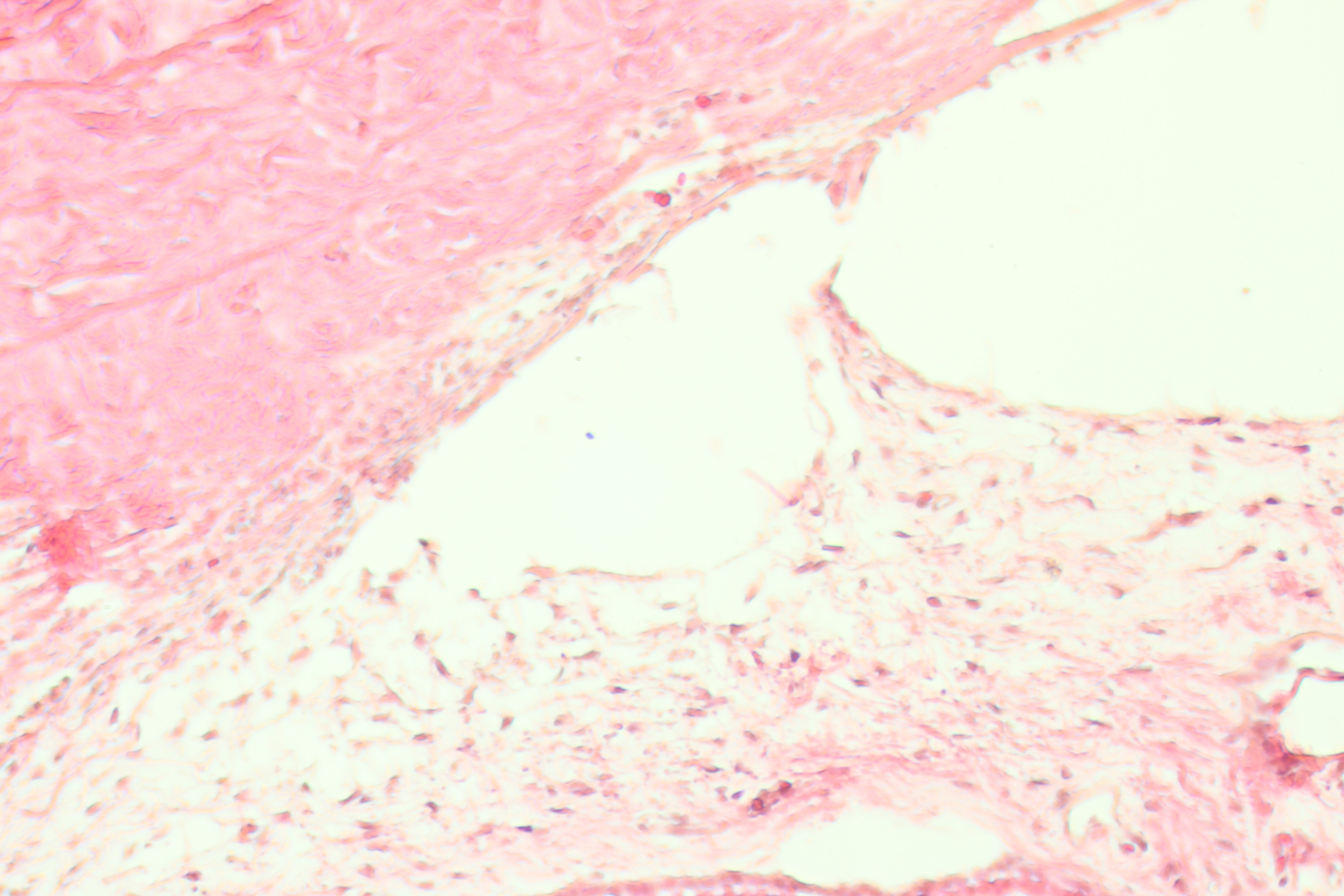

Supplement: S5 Photoset — (ZIP) [file pone.0138054.s006.zip › Multi Tx for Paper - SaratinIlomastat pics 2/IMG_6275.JPG]

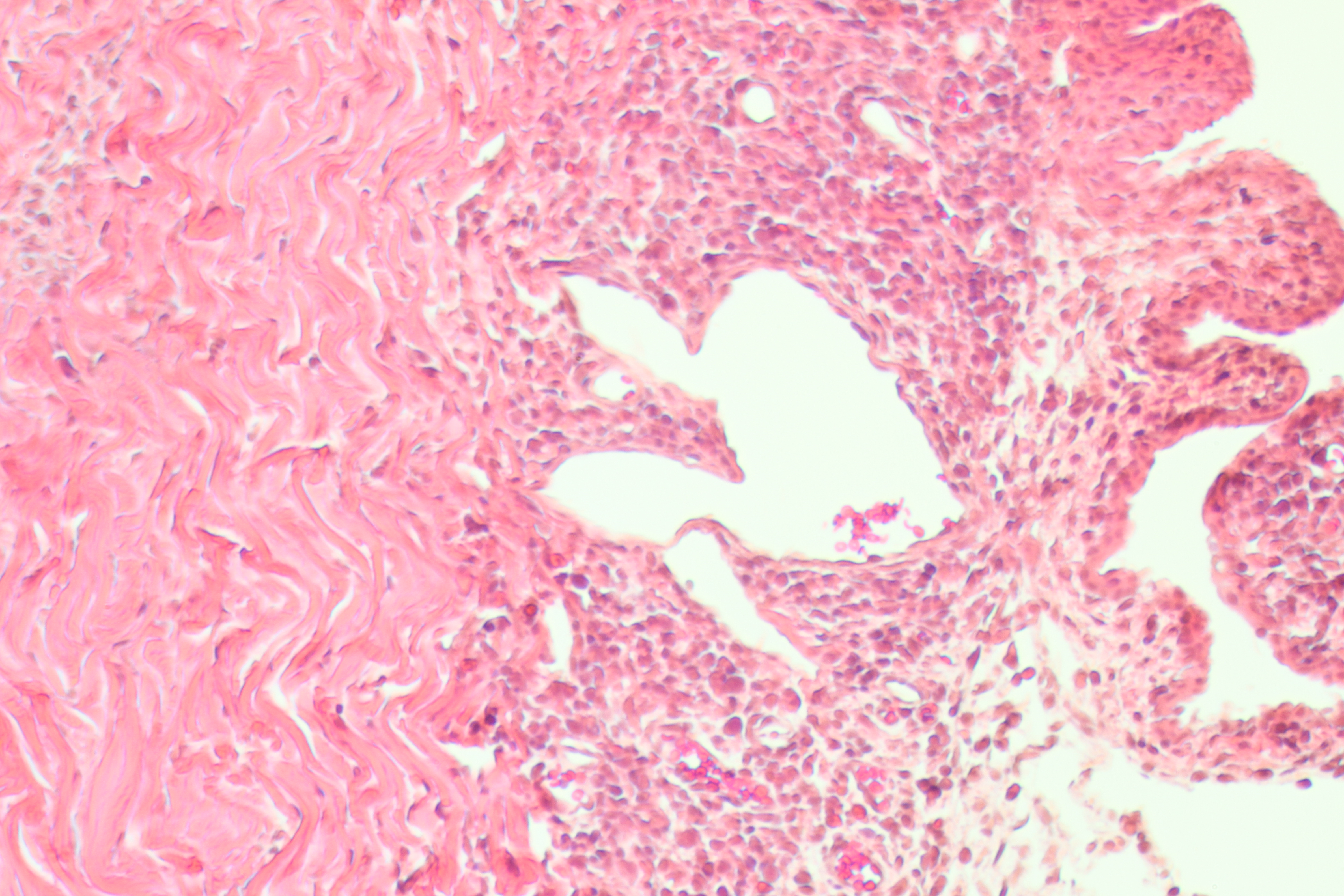

Supplement: S5 Photoset — (ZIP) [file pone.0138054.s006.zip › Multi Tx for Paper - SaratinIlomastat pics 2/IMG_6293.JPG]

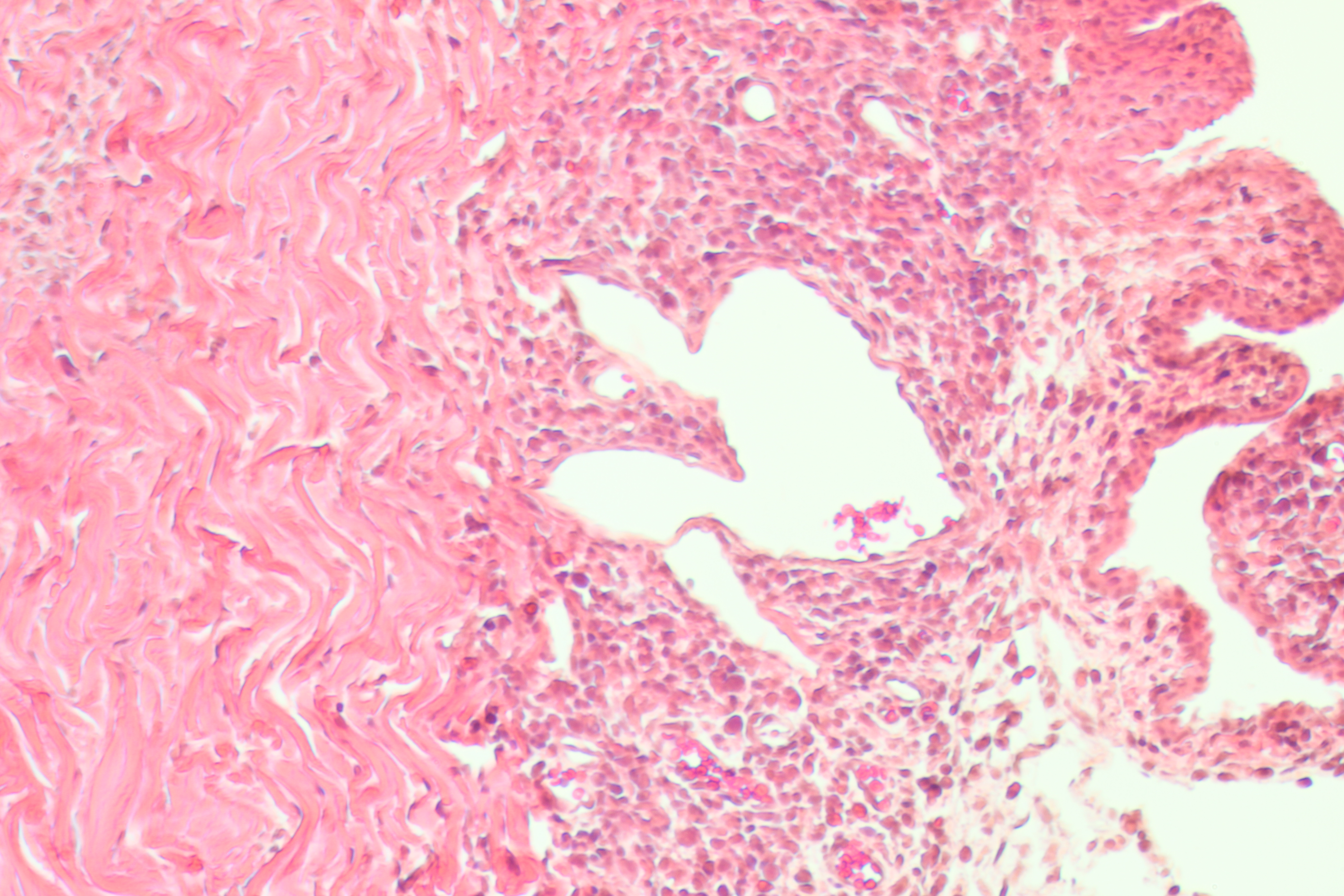

Supplement: S5 Photoset — (ZIP) [file pone.0138054.s006.zip › Multi Tx for Paper - SaratinIlomastat pics 2/IMG_6294.JPG]

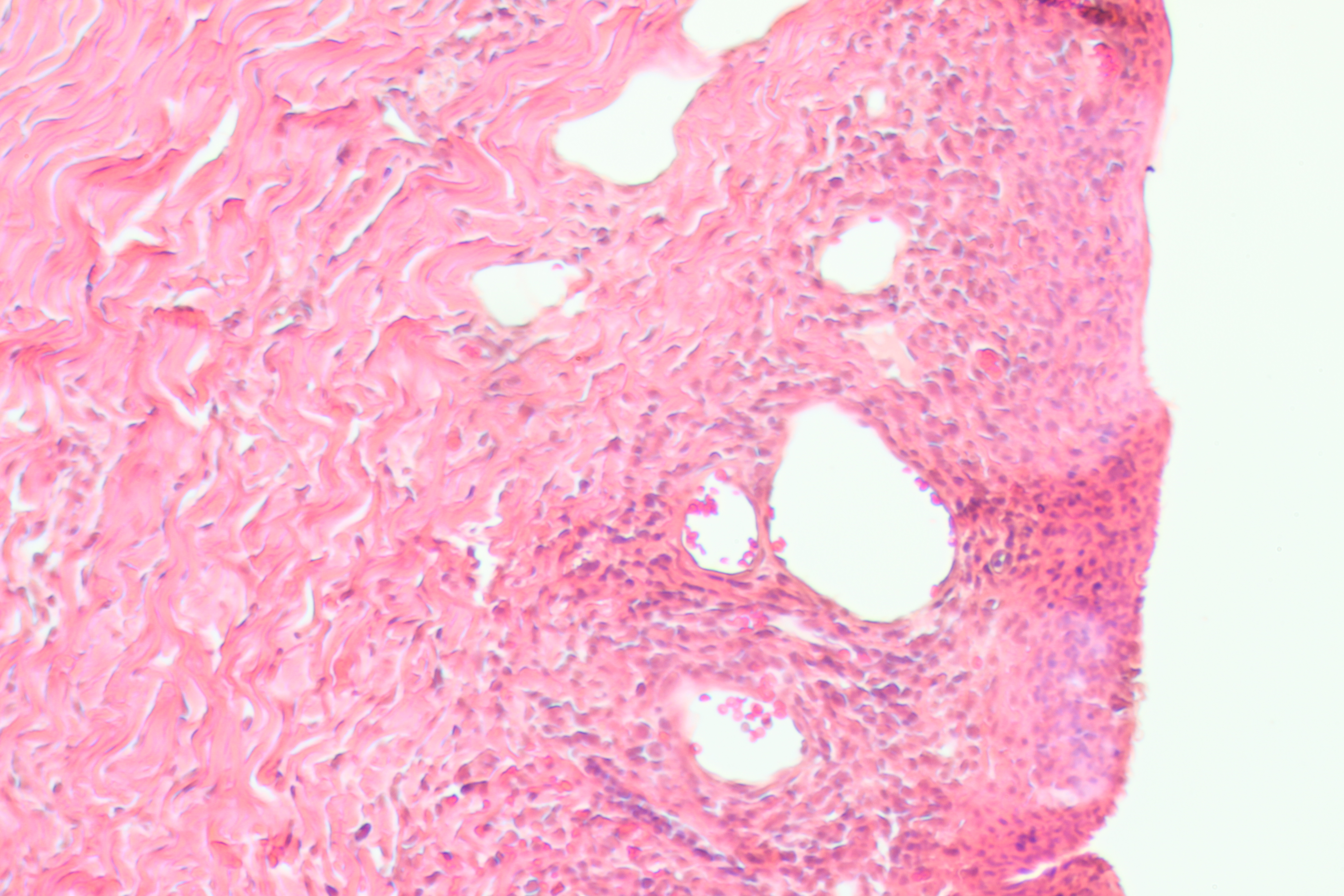

Supplement: S5 Photoset — (ZIP) [file pone.0138054.s006.zip › Multi Tx for Paper - SaratinIlomastat pics 2/IMG_6295.JPG]

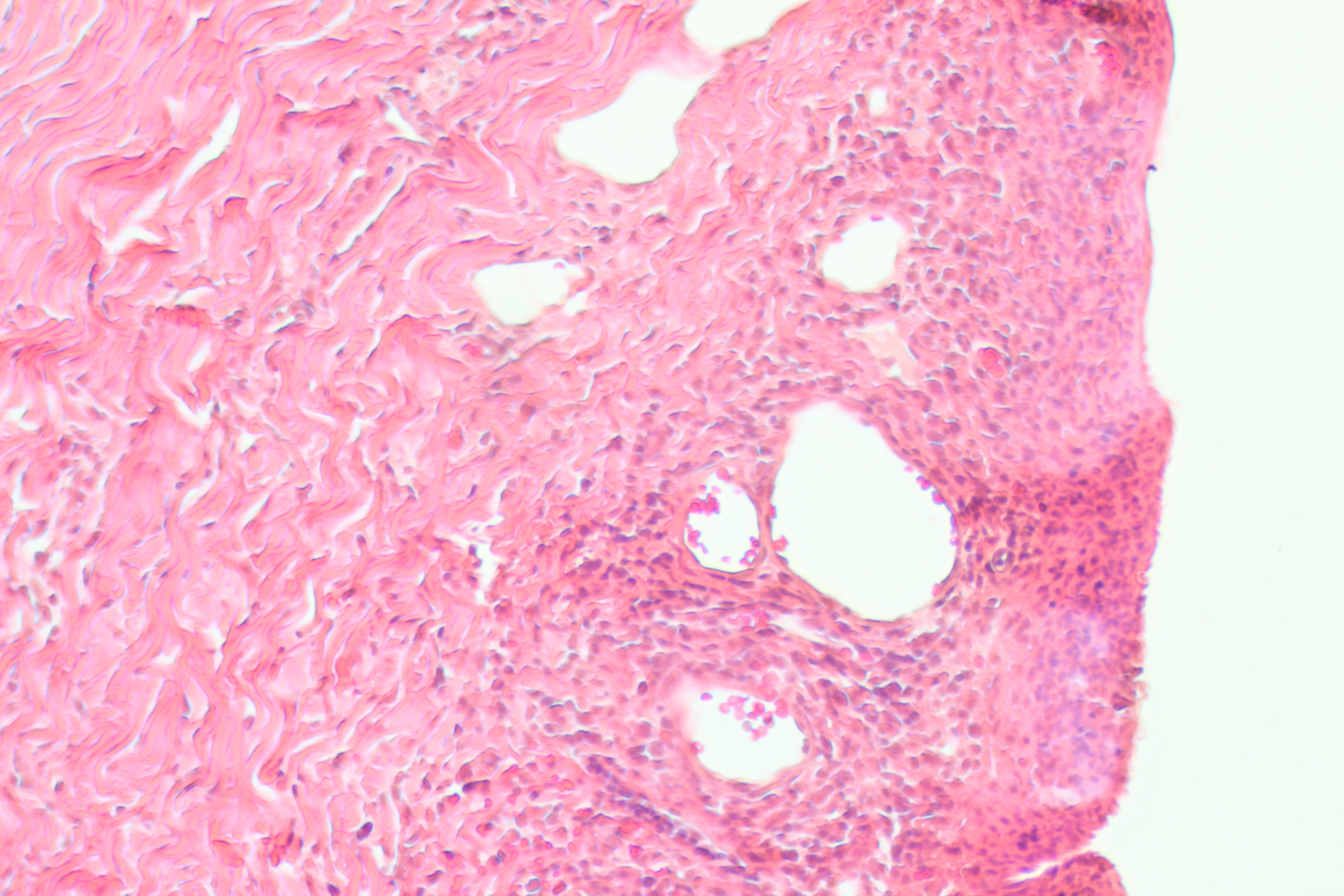

Supplement: S5 Photoset — (ZIP) [file pone.0138054.s006.zip › Multi Tx for Paper - SaratinIlomastat pics 2/IMG_6296.JPG]

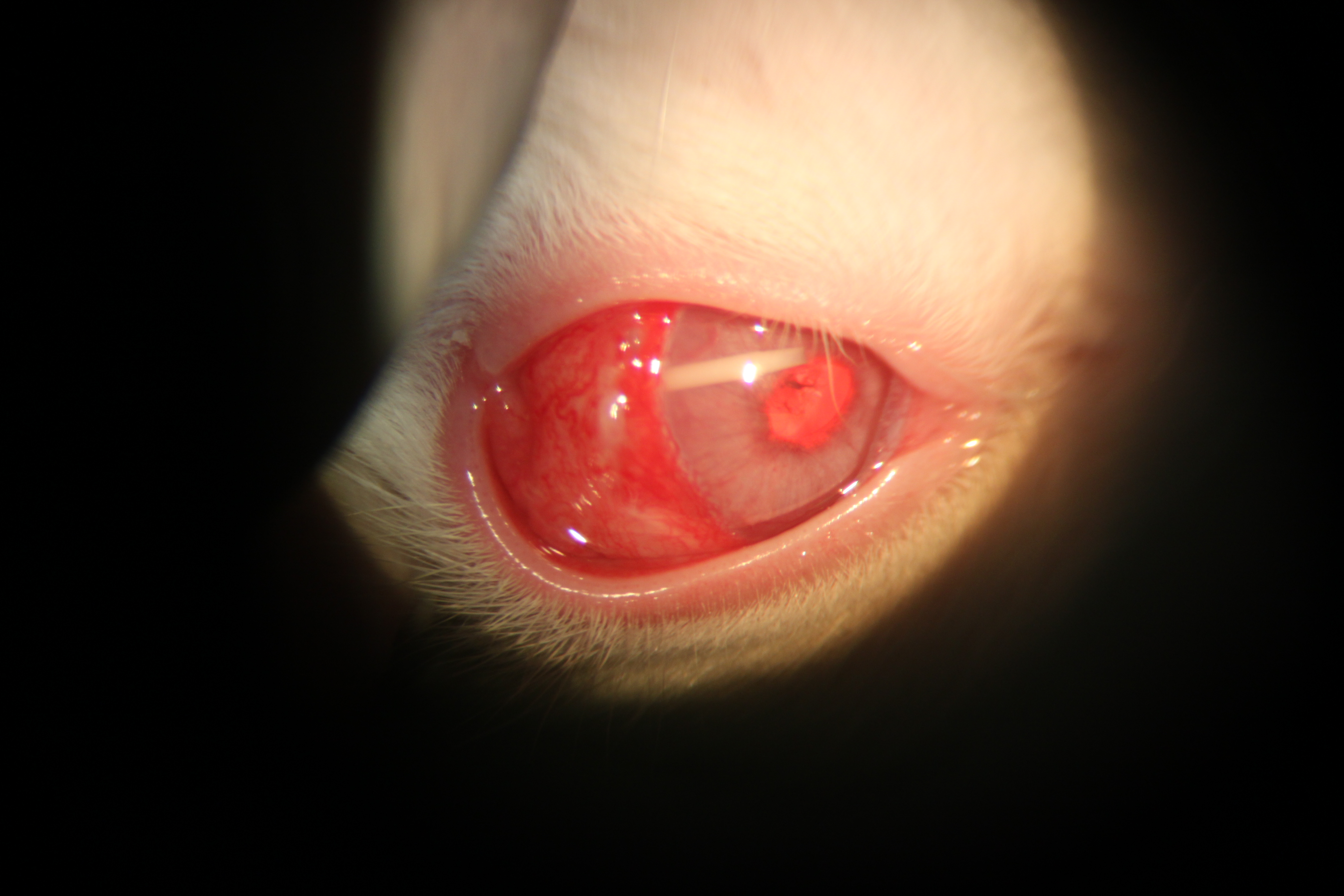

Supplement: S6 Photoset — (ZIP) [file pone.0138054.s007.zip › Multi Tx for Paper - SaratinIlomastatAvastin pics 1/IMG_1342.JPG]
