# Supplementary figures and images for: Sequential Therapy with Saratin, Bevacizumab and Ilomastat to Prolong Bleb Function following Glaucoma Filtration Surgery in a Rabbit Model (part 2 of 2)
Source: PLoS One. 2015 Sep 22;10(9):e0138054. doi: 10.1371/journal.pone.0138054 (PMC4578880; doi:10.1371/journal.pone.0138054)

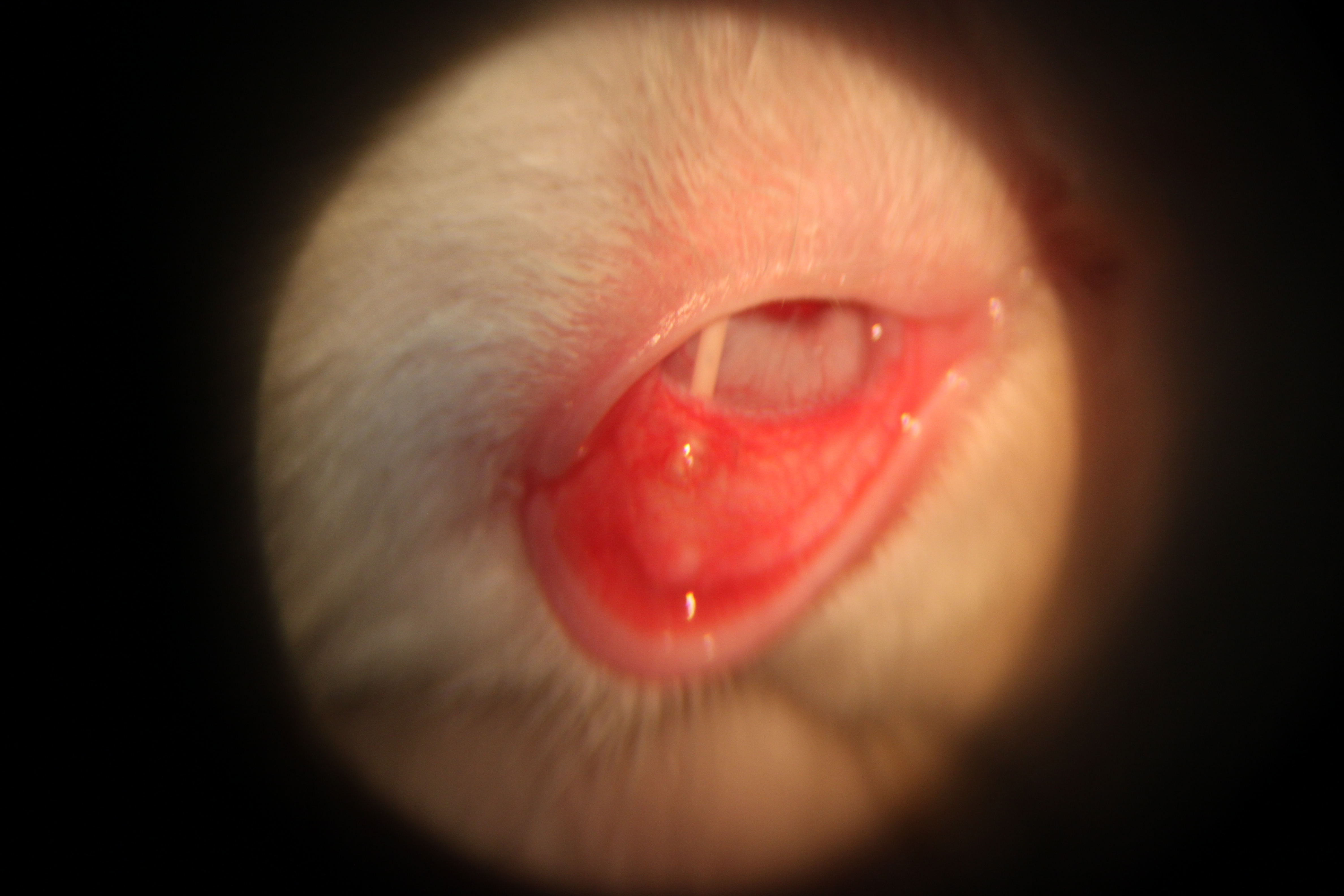

Supplement: S6 Photoset — (ZIP) [file pone.0138054.s007.zip › Multi Tx for Paper - SaratinIlomastatAvastin pics 1/IMG_2342.JPG]

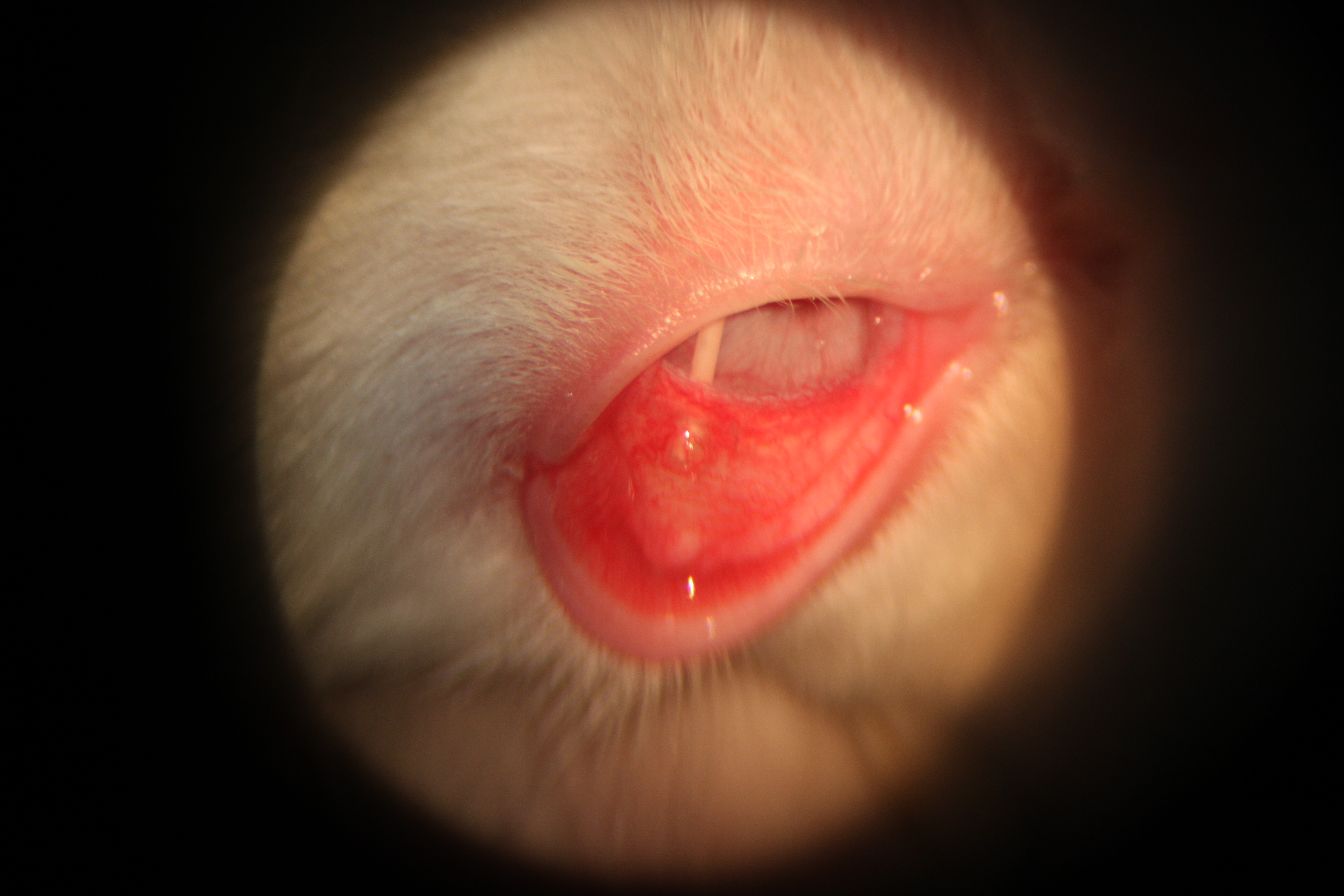

Supplement: S6 Photoset — (ZIP) [file pone.0138054.s007.zip › Multi Tx for Paper - SaratinIlomastatAvastin pics 1/IMG_2343.JPG]

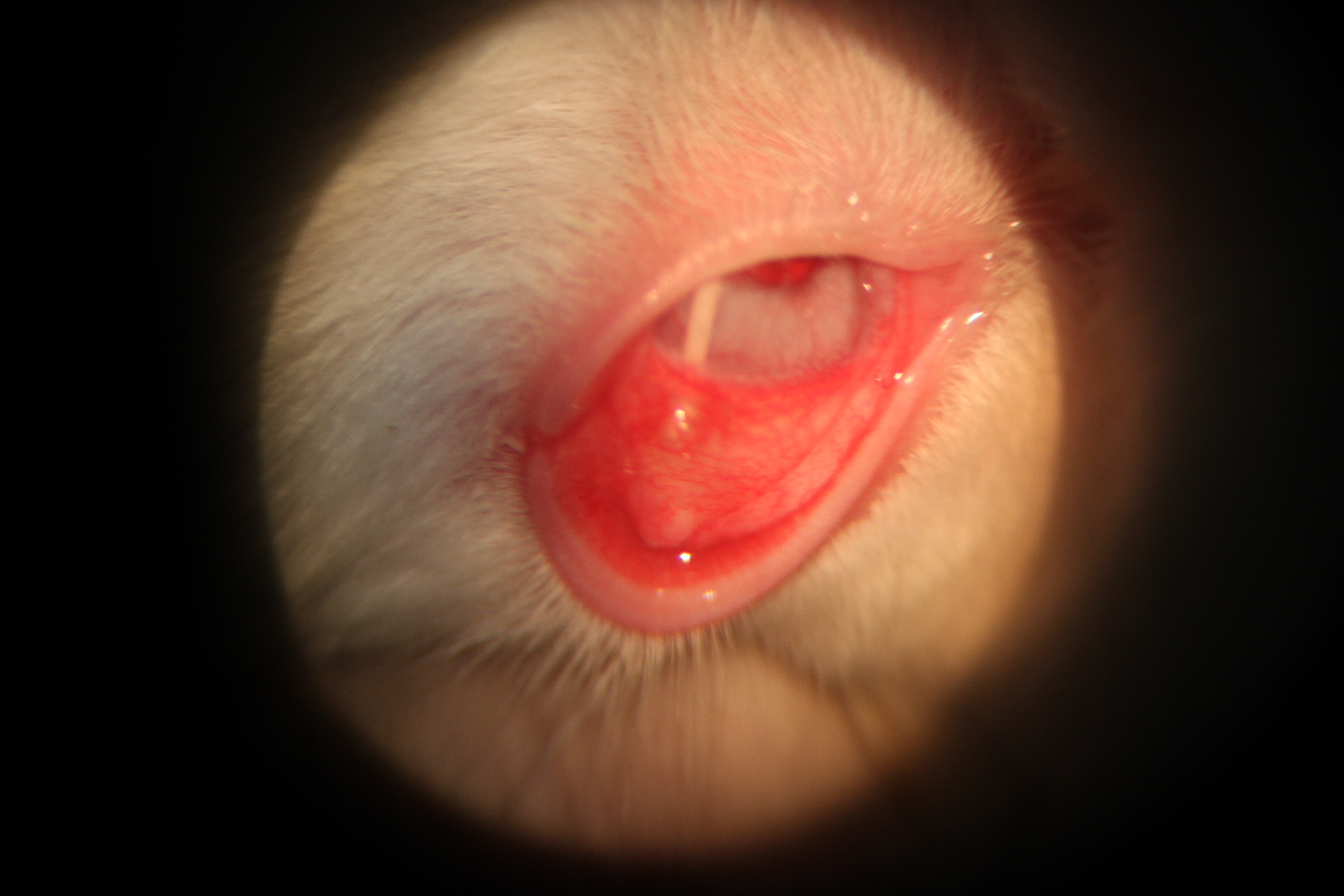

Supplement: S6 Photoset — (ZIP) [file pone.0138054.s007.zip › Multi Tx for Paper - SaratinIlomastatAvastin pics 1/IMG_2344.JPG]

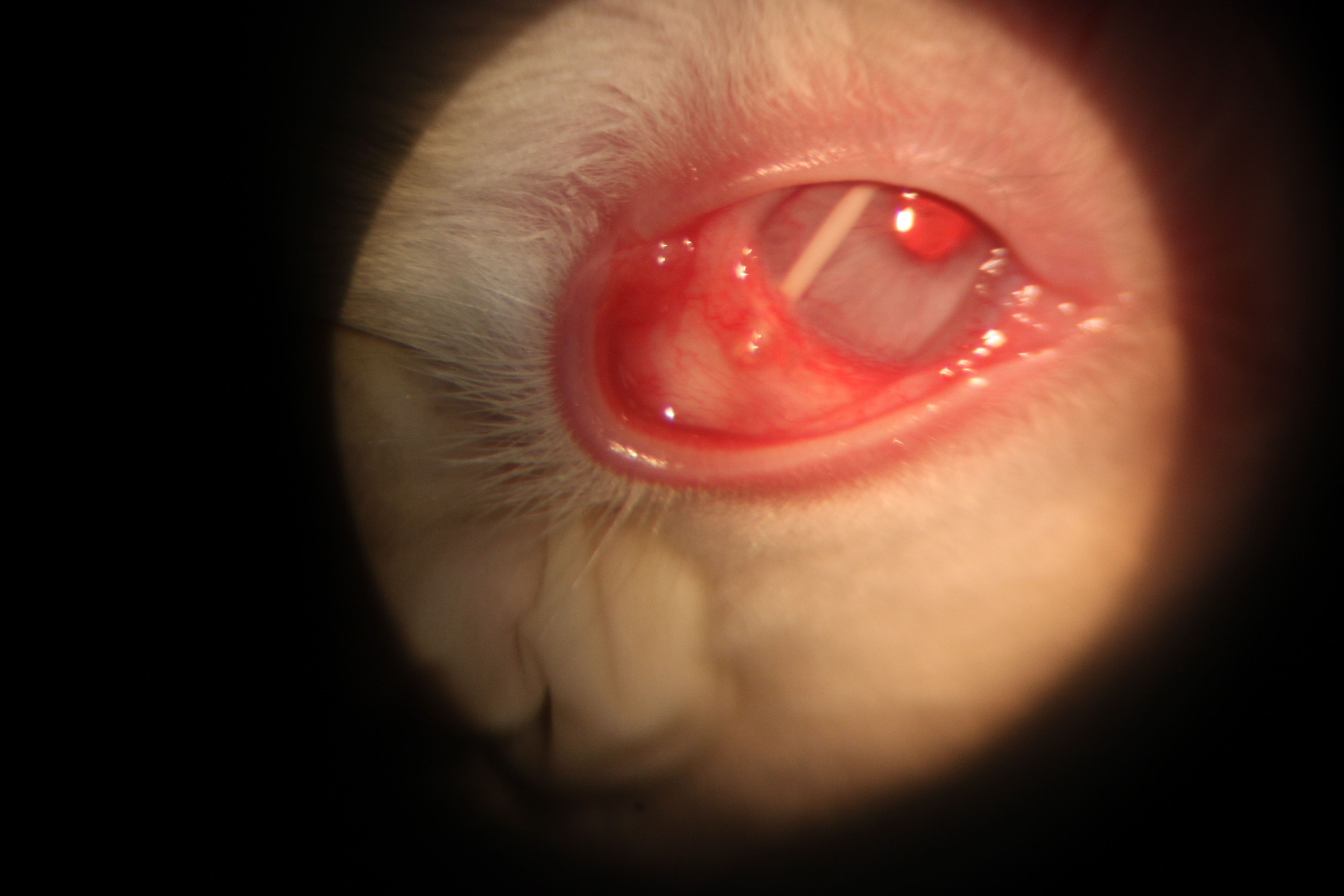

Supplement: S6 Photoset — (ZIP) [file pone.0138054.s007.zip › Multi Tx for Paper - SaratinIlomastatAvastin pics 1/IMG_2481.JPG]

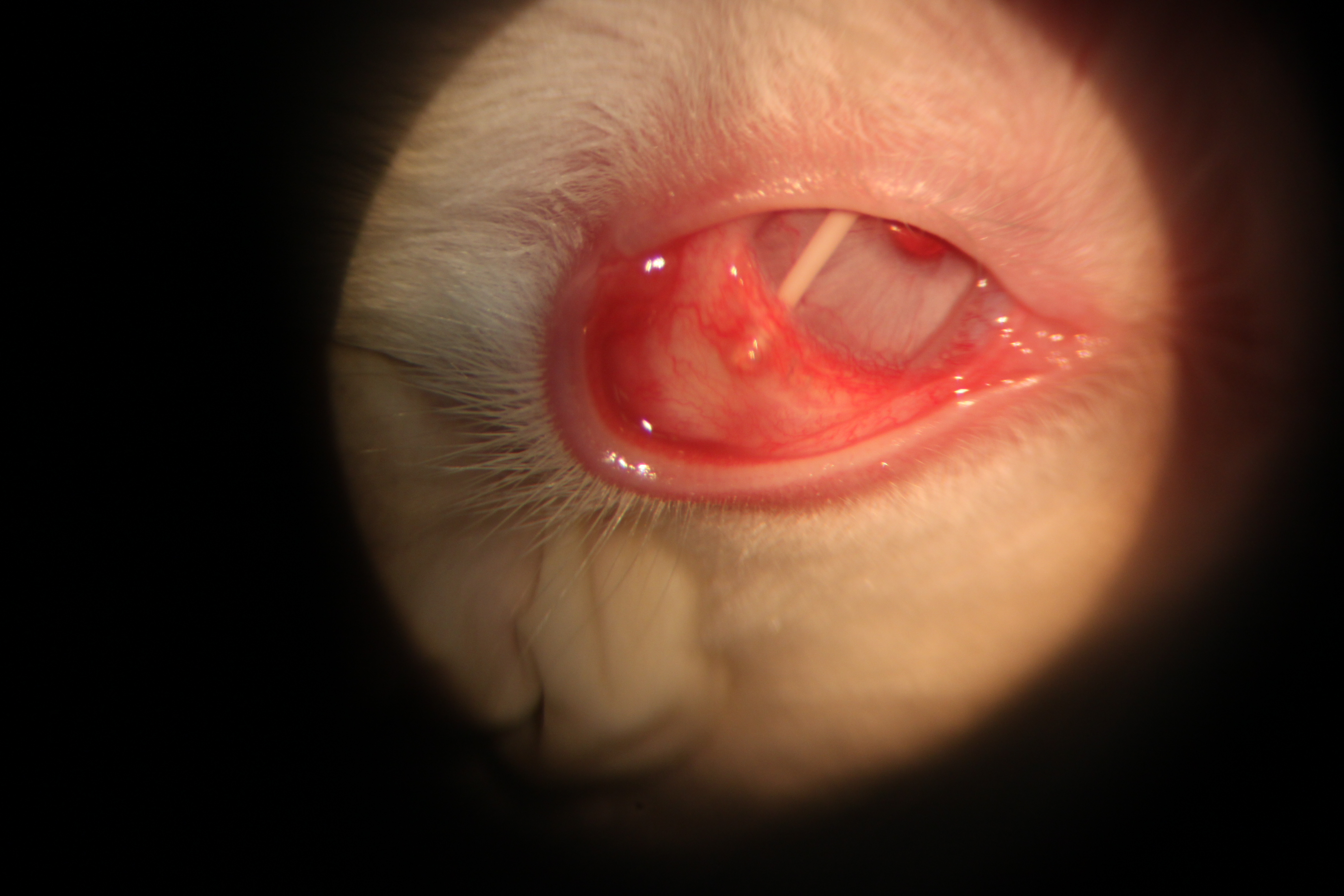

Supplement: S6 Photoset — (ZIP) [file pone.0138054.s007.zip › Multi Tx for Paper - SaratinIlomastatAvastin pics 1/IMG_2485.JPG]

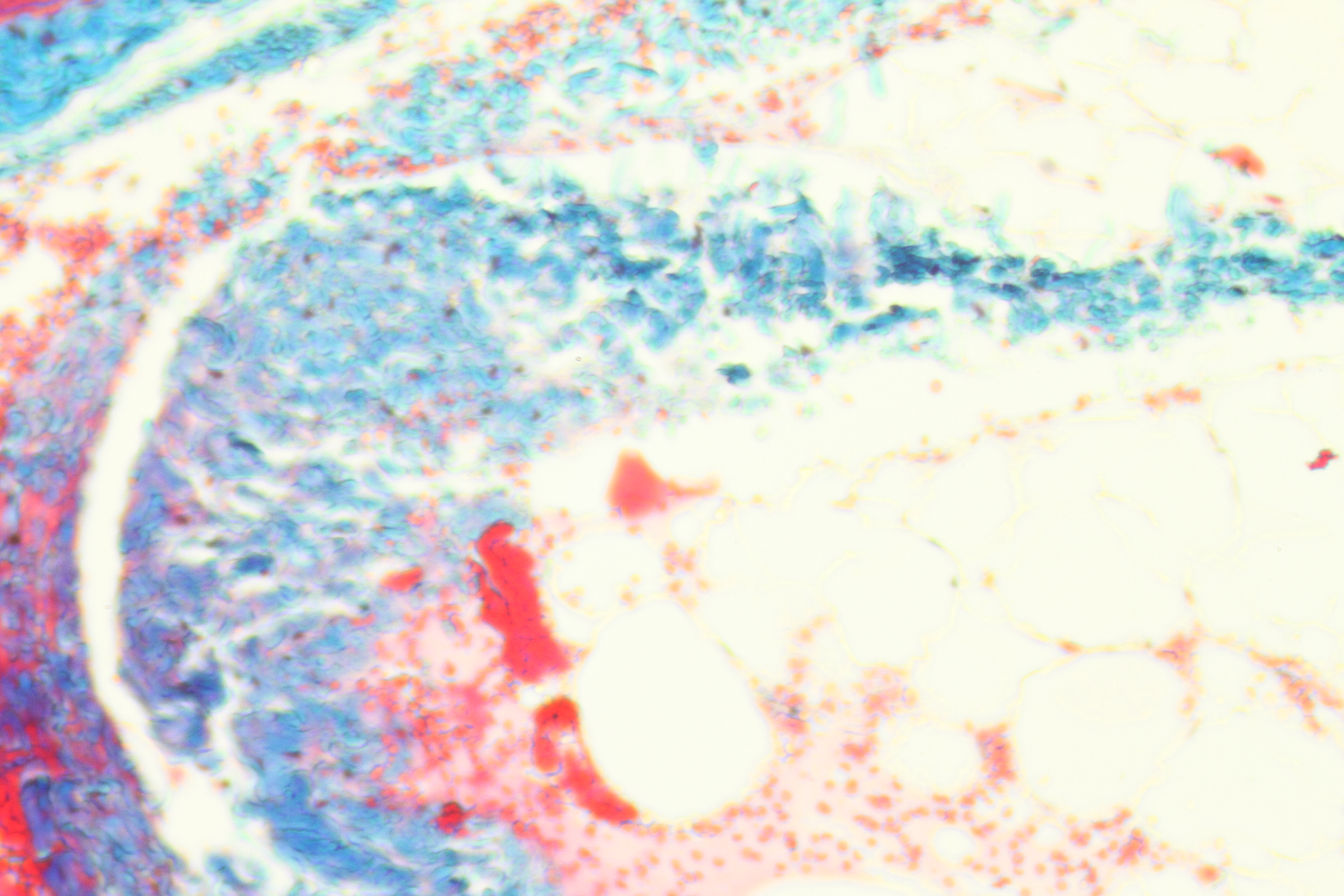

Supplement: S6 Photoset — (ZIP) [file pone.0138054.s007.zip › Multi Tx for Paper - SaratinIlomastatAvastin pics 1/IMG_6138.JPG]

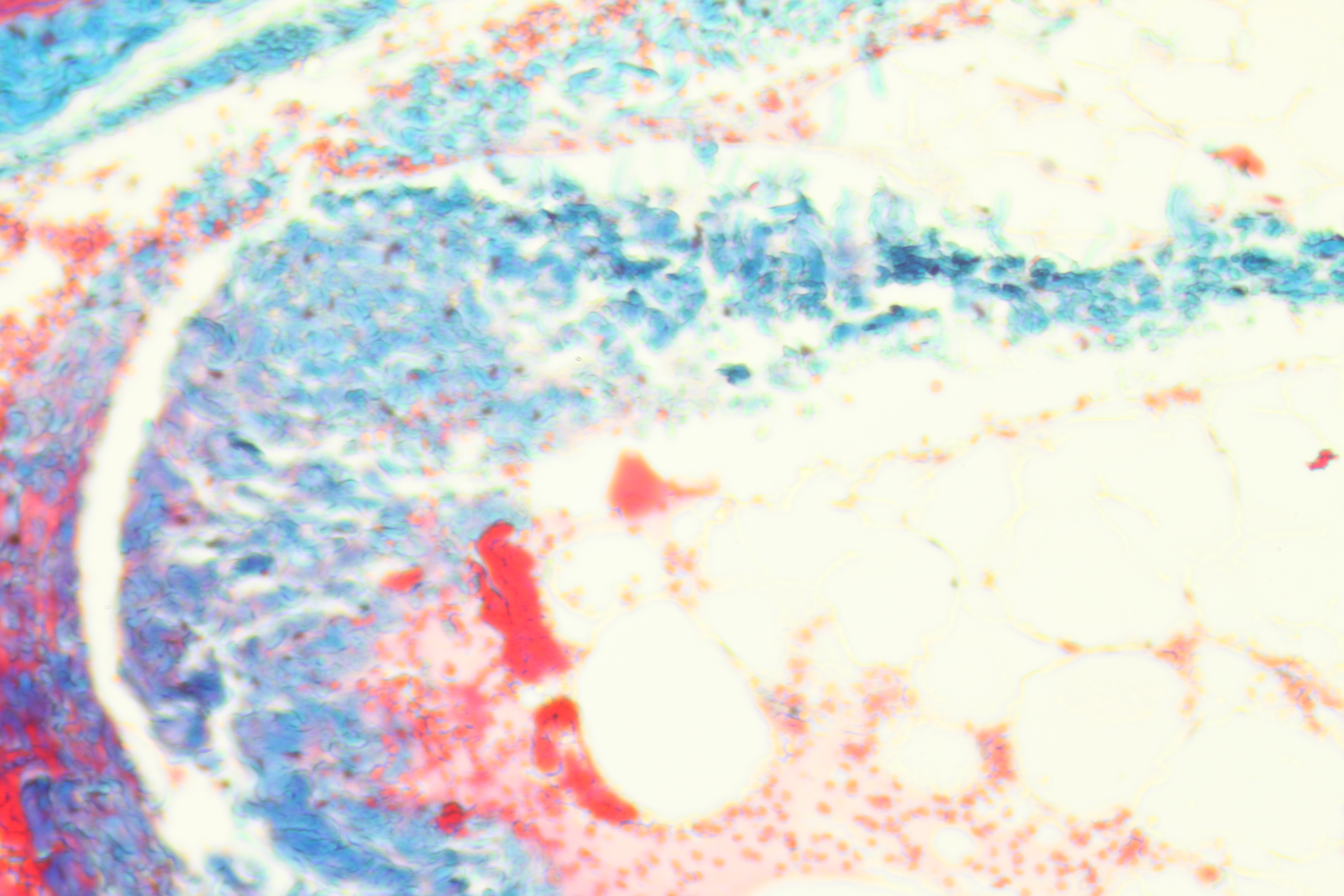

Supplement: S6 Photoset — (ZIP) [file pone.0138054.s007.zip › Multi Tx for Paper - SaratinIlomastatAvastin pics 1/IMG_6139.JPG]

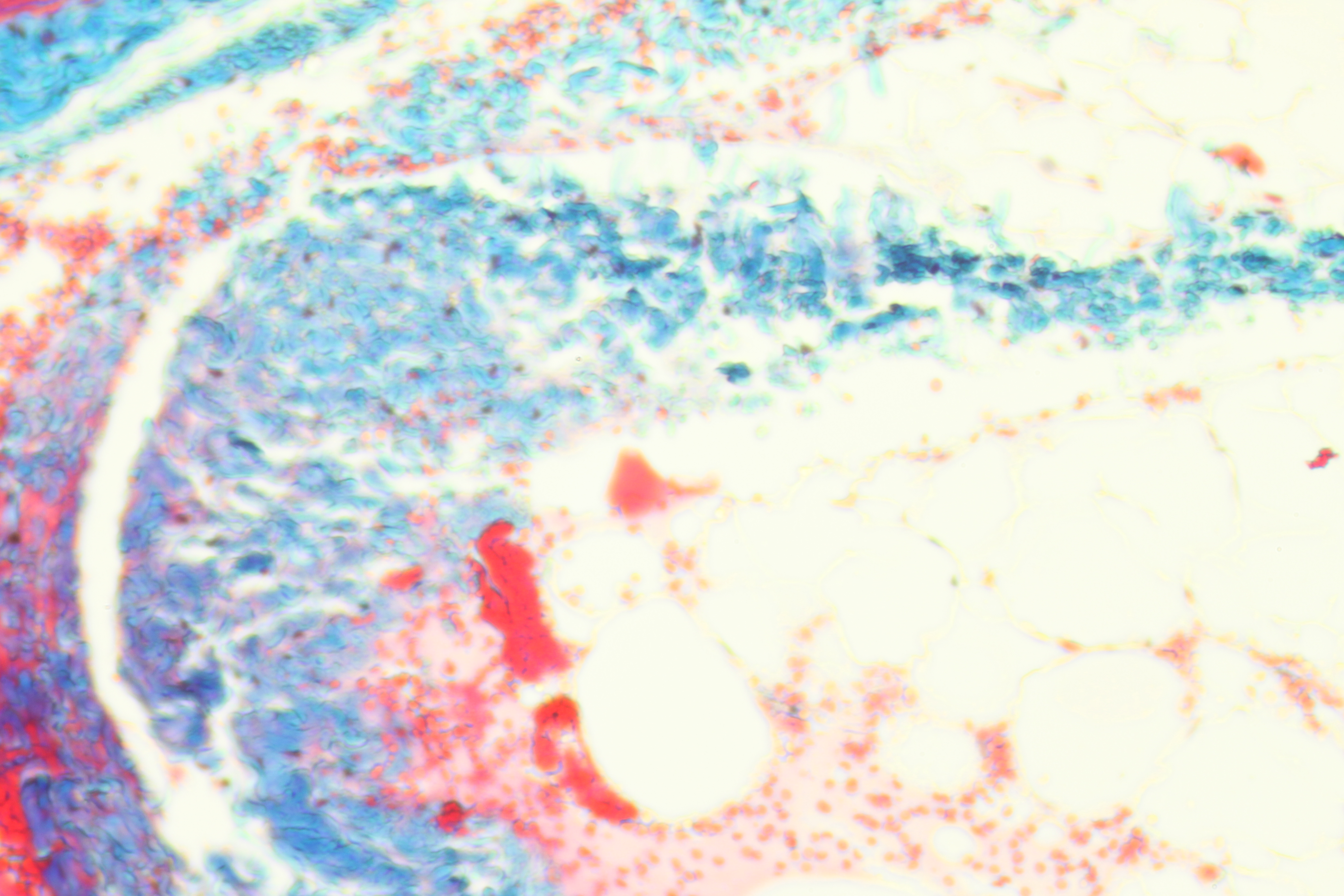

Supplement: S6 Photoset — (ZIP) [file pone.0138054.s007.zip › Multi Tx for Paper - SaratinIlomastatAvastin pics 1/IMG_6140.JPG]

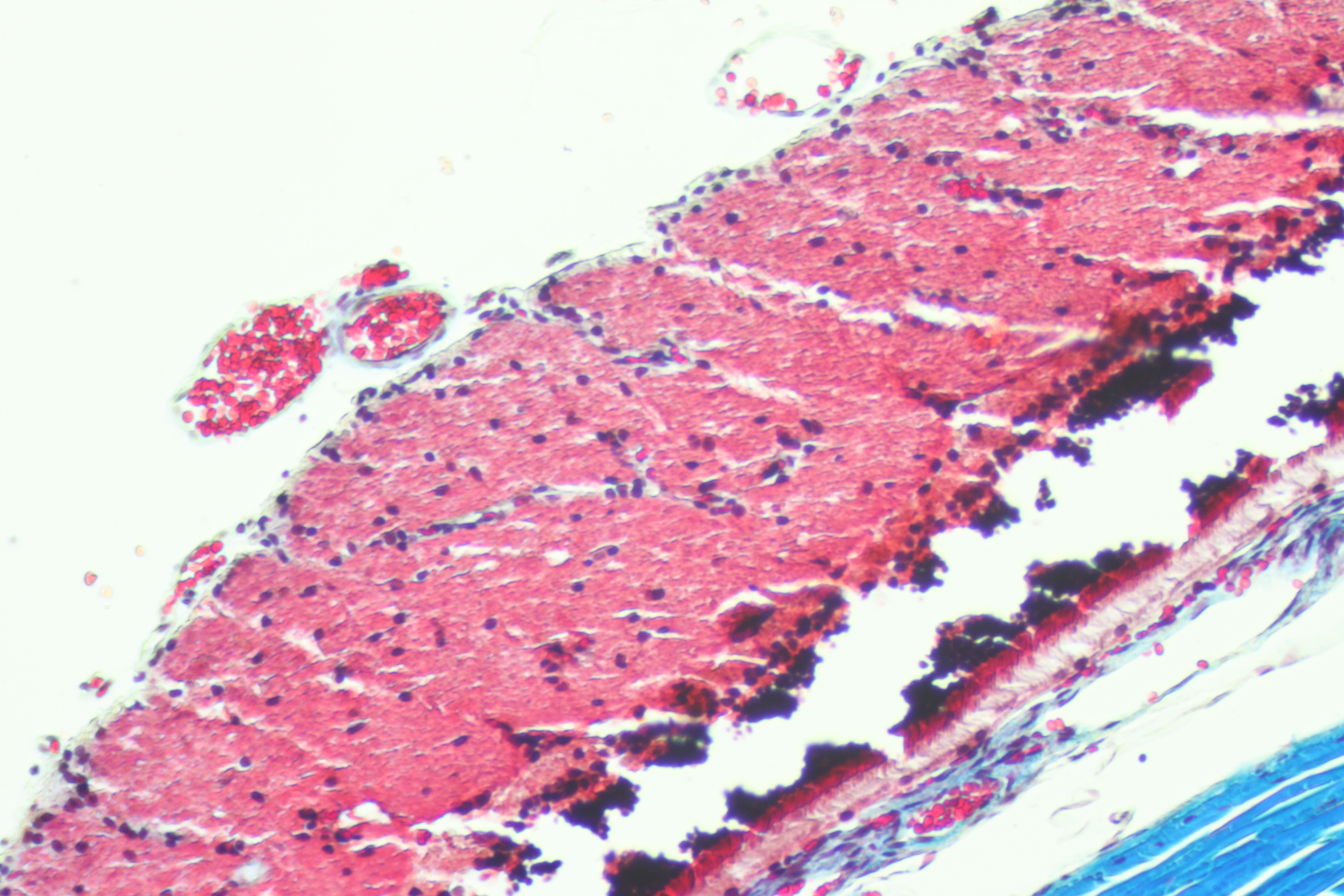

Supplement: S6 Photoset — (ZIP) [file pone.0138054.s007.zip › Multi Tx for Paper - SaratinIlomastatAvastin pics 1/IMG_6141.JPG]

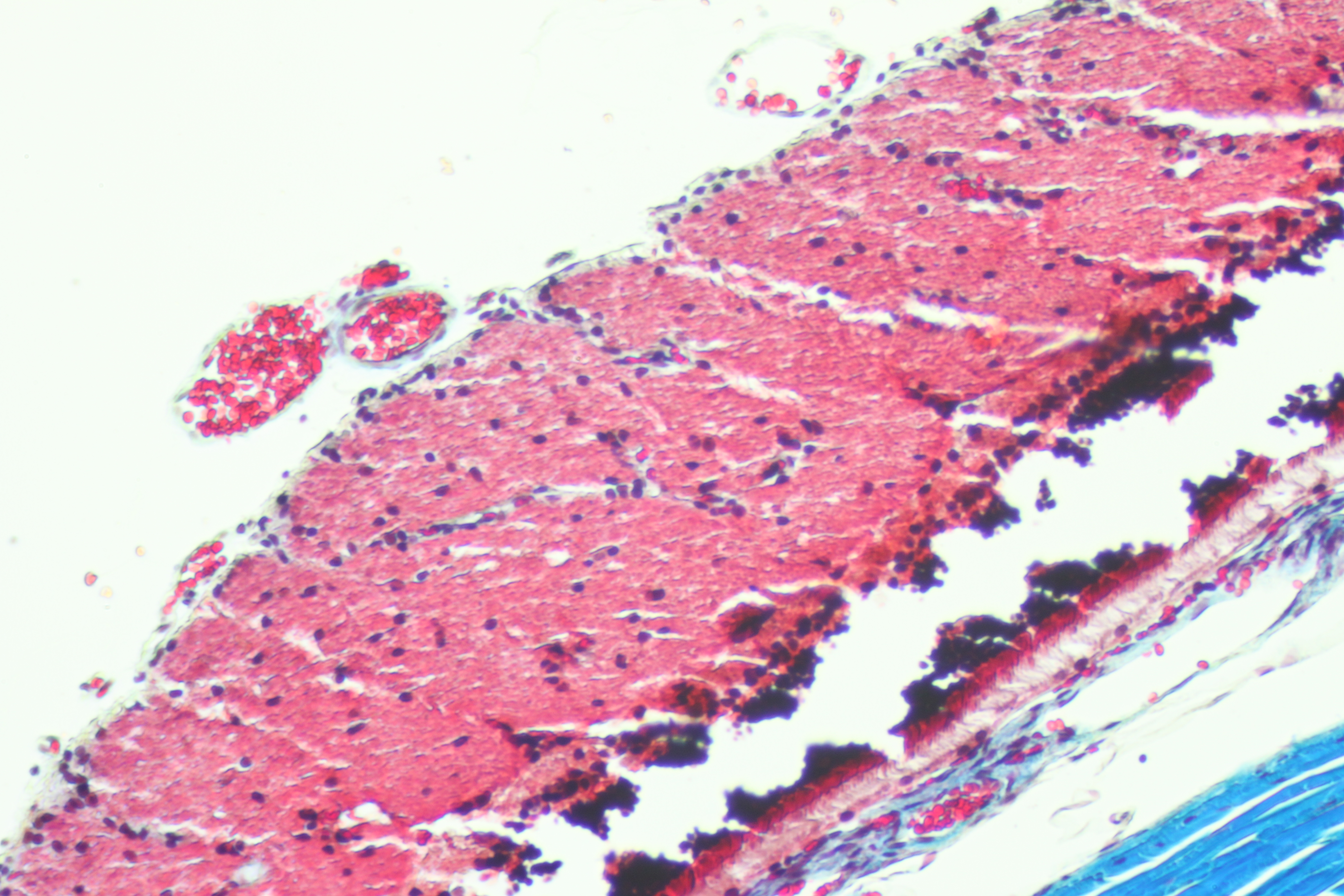

Supplement: S6 Photoset — (ZIP) [file pone.0138054.s007.zip › Multi Tx for Paper - SaratinIlomastatAvastin pics 1/IMG_6142.JPG]

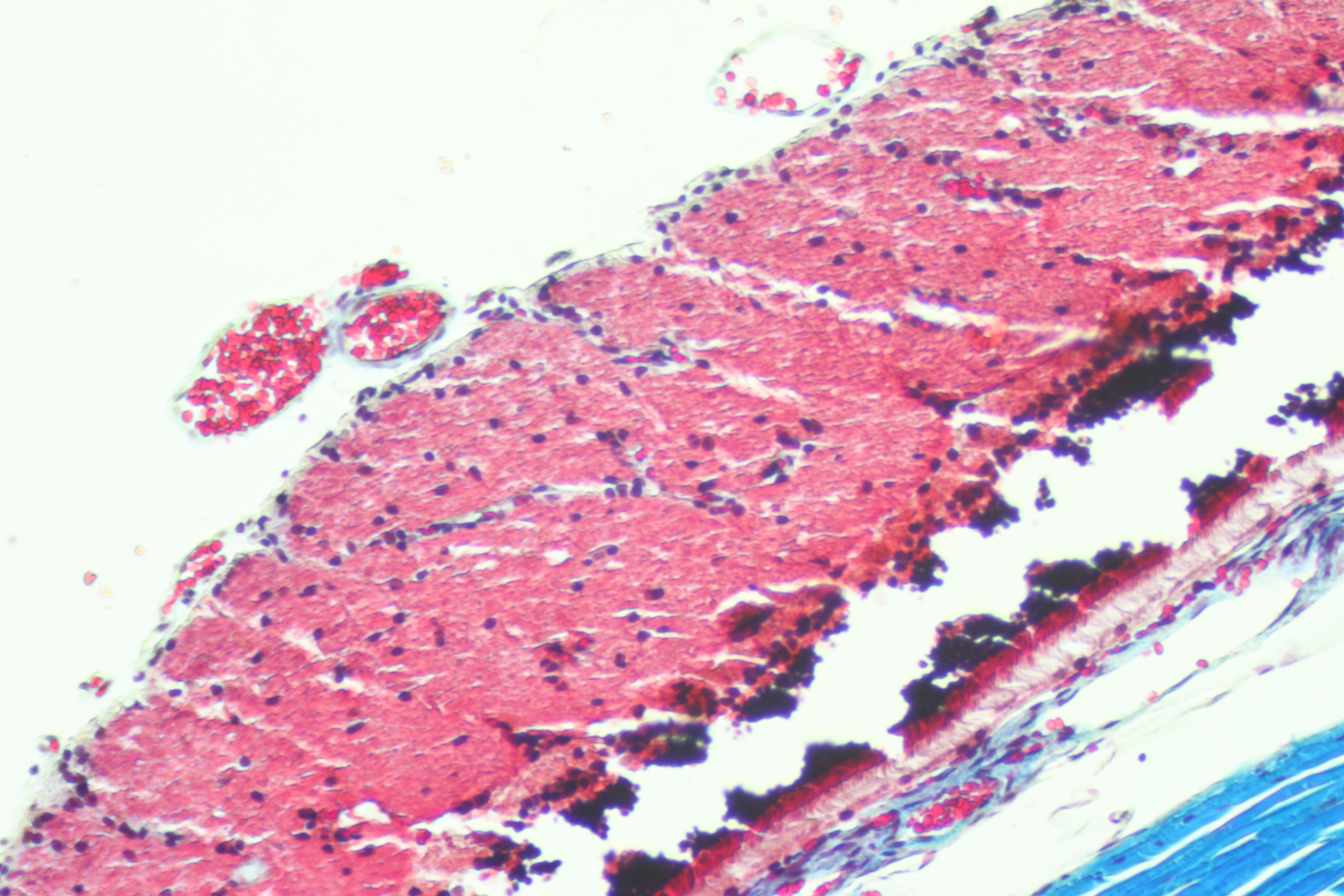

Supplement: S6 Photoset — (ZIP) [file pone.0138054.s007.zip › Multi Tx for Paper - SaratinIlomastatAvastin pics 1/IMG_6143.JPG]

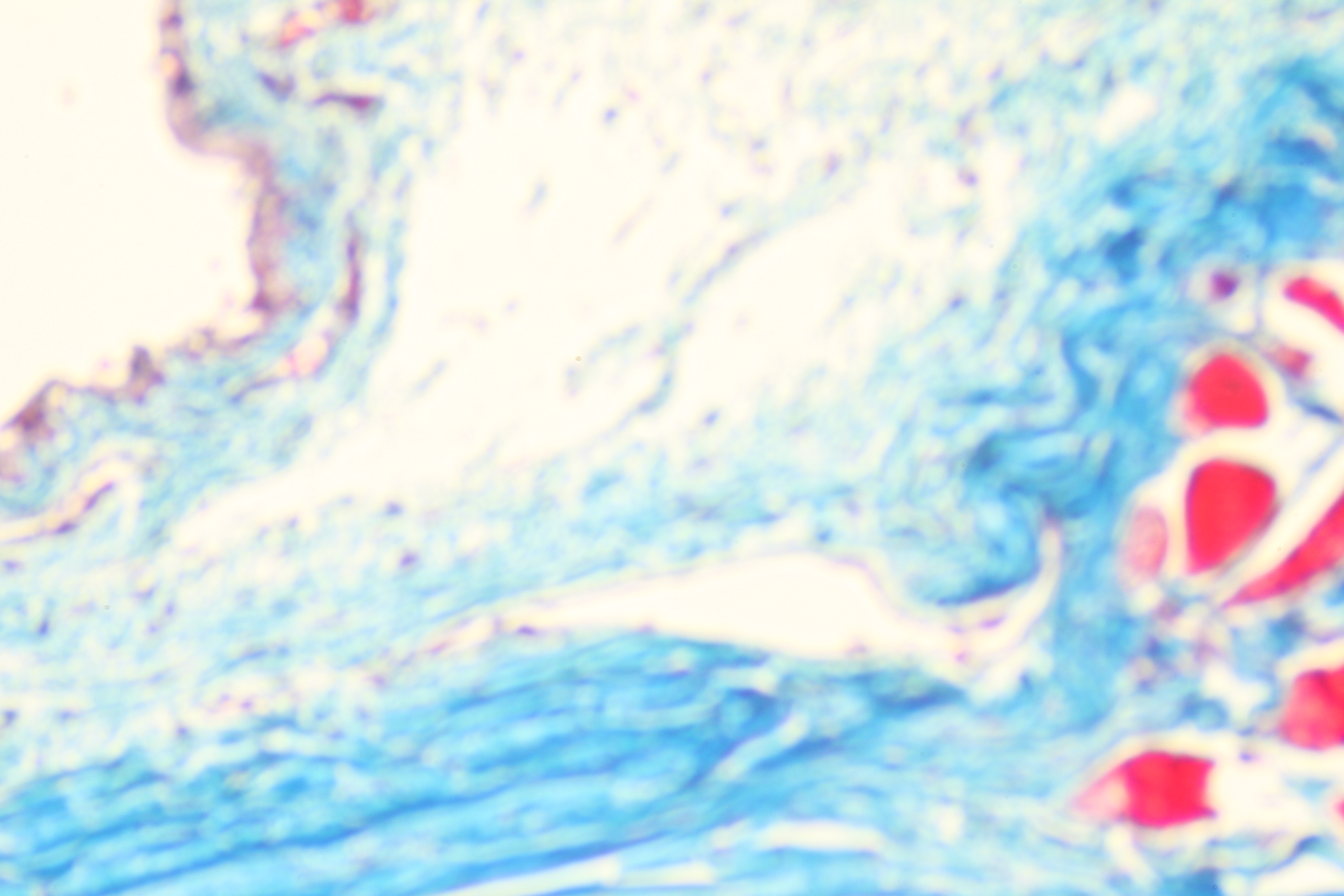

Supplement: S6 Photoset — (ZIP) [file pone.0138054.s007.zip › Multi Tx for Paper - SaratinIlomastatAvastin pics 1/IMG_6144.JPG]

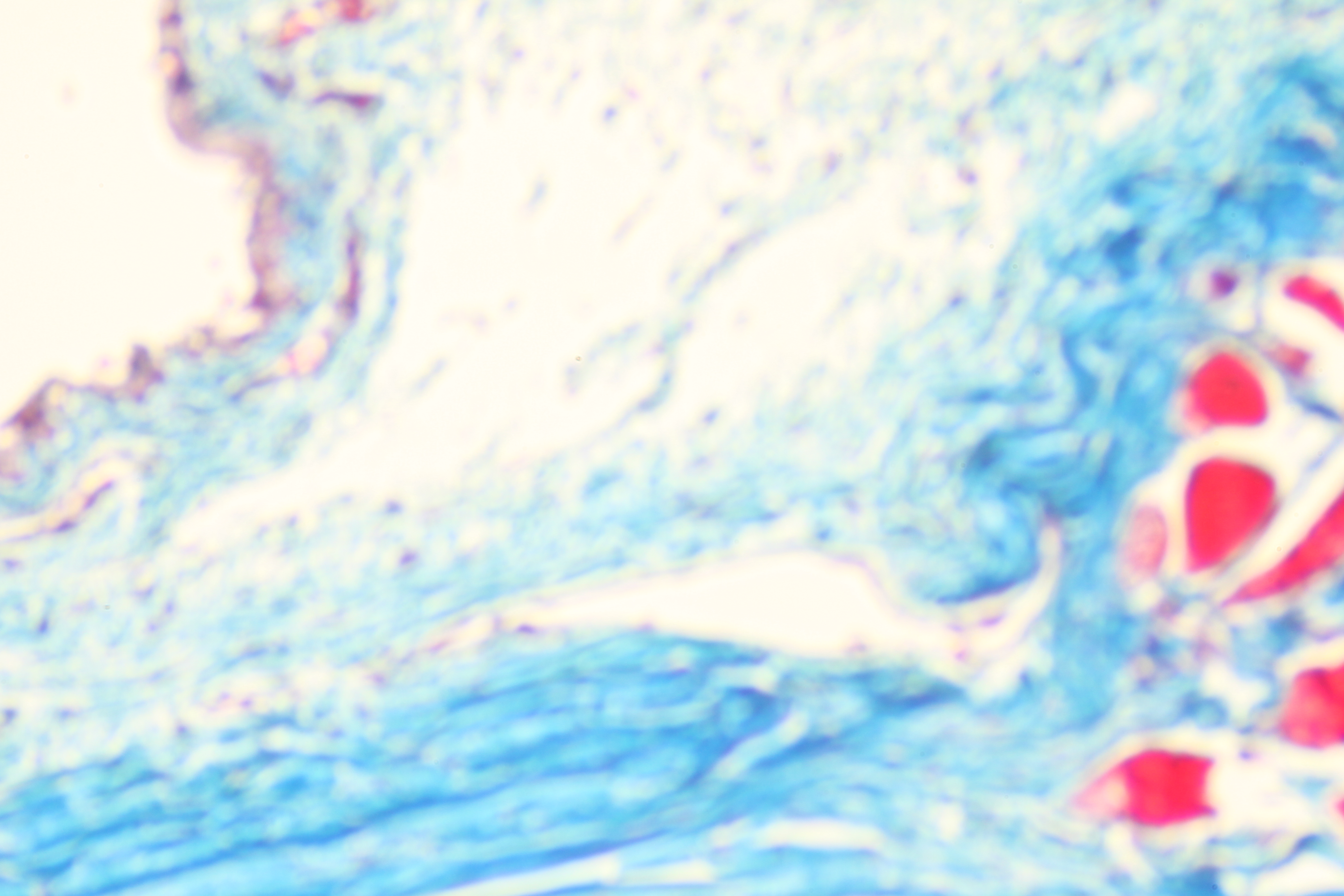

Supplement: S6 Photoset — (ZIP) [file pone.0138054.s007.zip › Multi Tx for Paper - SaratinIlomastatAvastin pics 1/IMG_6145.JPG]

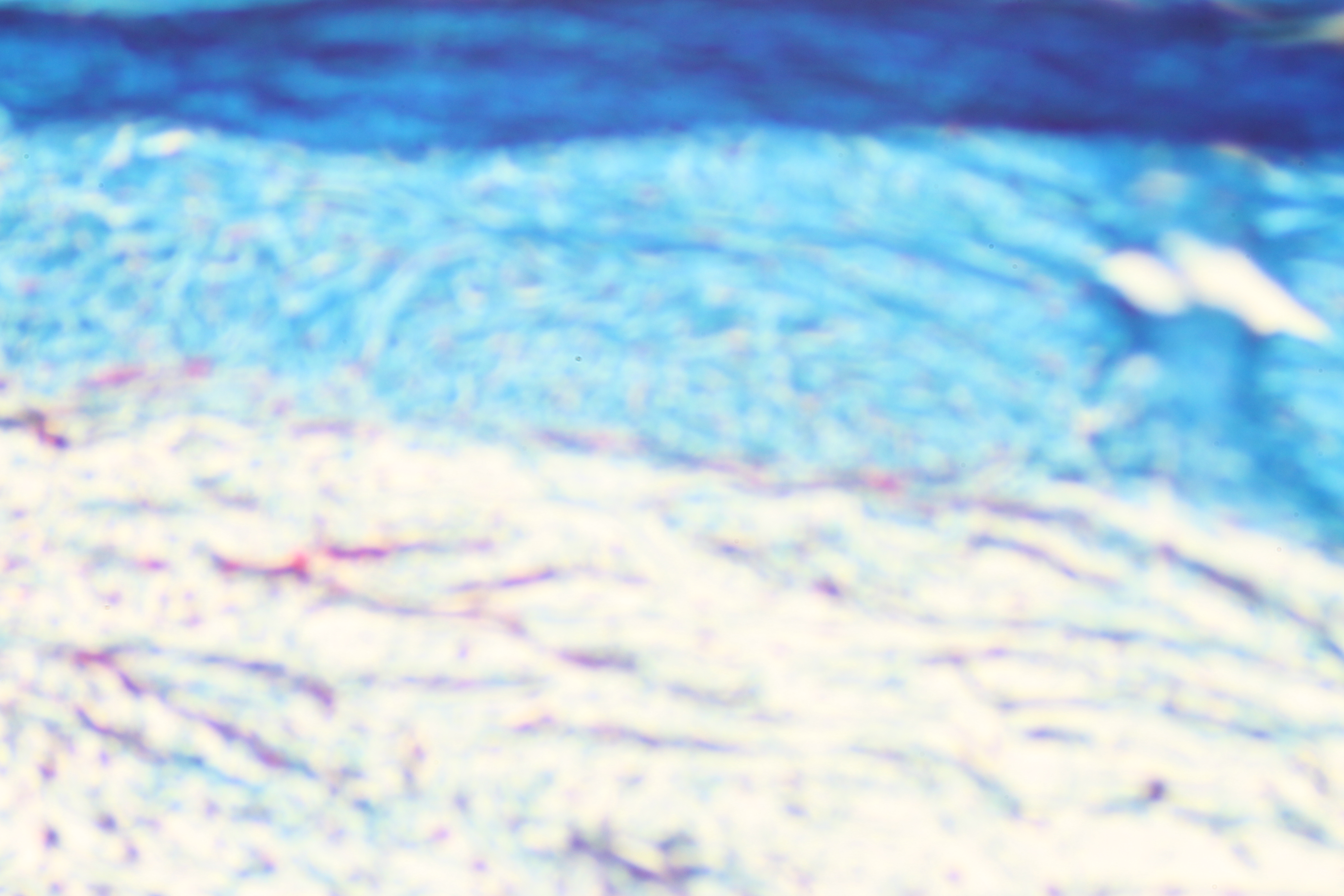

Supplement: S6 Photoset — (ZIP) [file pone.0138054.s007.zip › Multi Tx for Paper - SaratinIlomastatAvastin pics 1/IMG_6146.JPG]

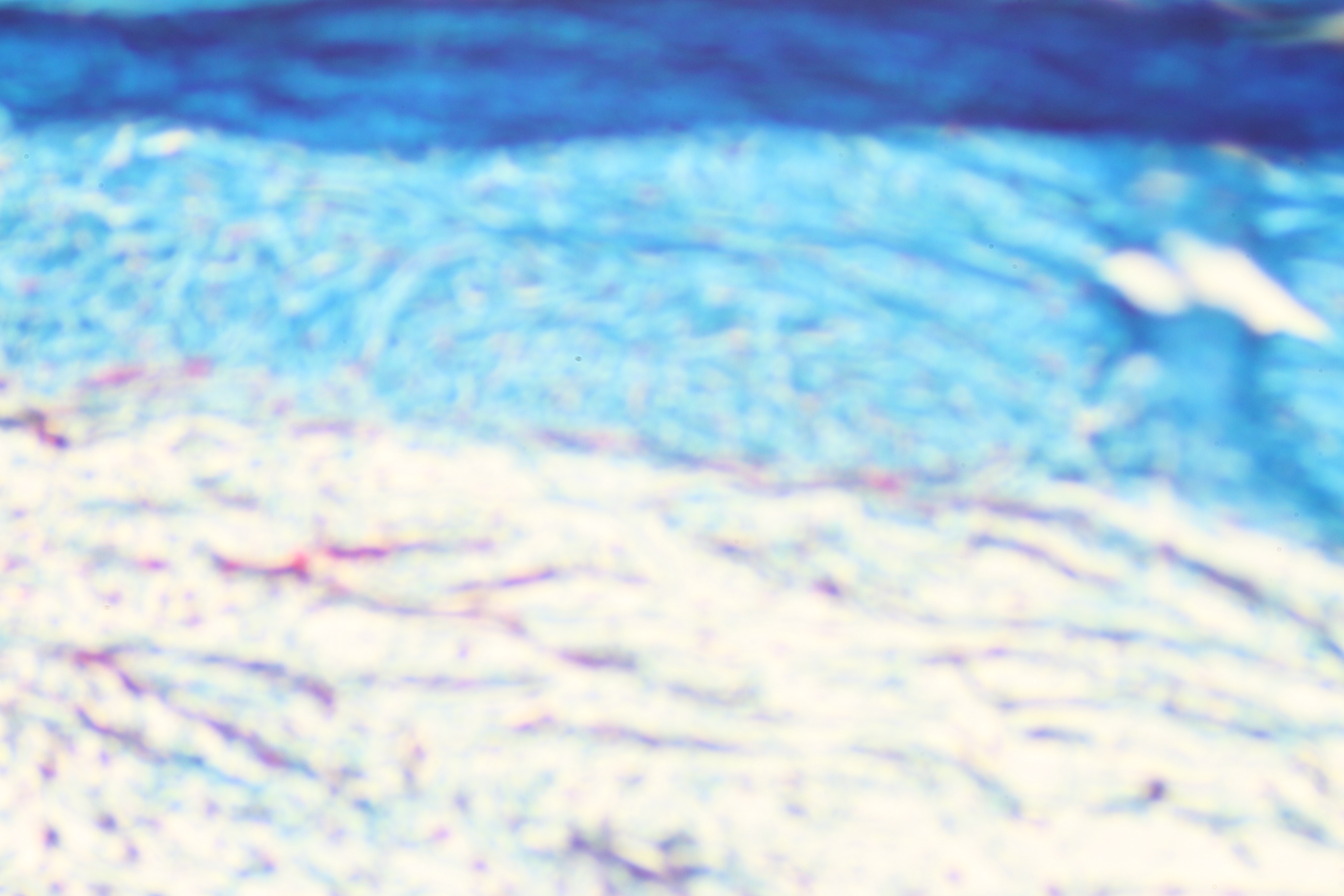

Supplement: S6 Photoset — (ZIP) [file pone.0138054.s007.zip › Multi Tx for Paper - SaratinIlomastatAvastin pics 1/IMG_6147.JPG]

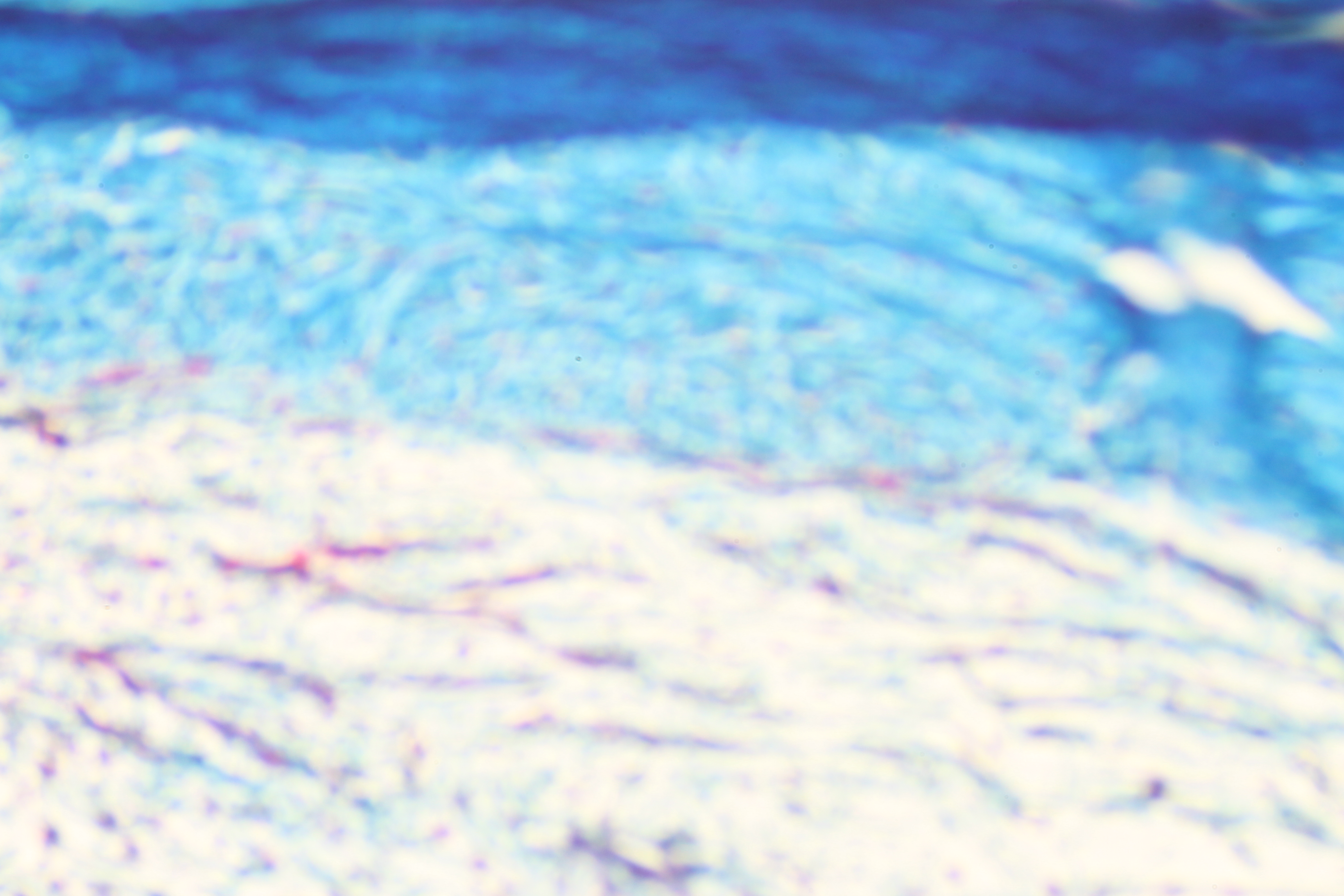

Supplement: S6 Photoset — (ZIP) [file pone.0138054.s007.zip › Multi Tx for Paper - SaratinIlomastatAvastin pics 1/IMG_6148.JPG]

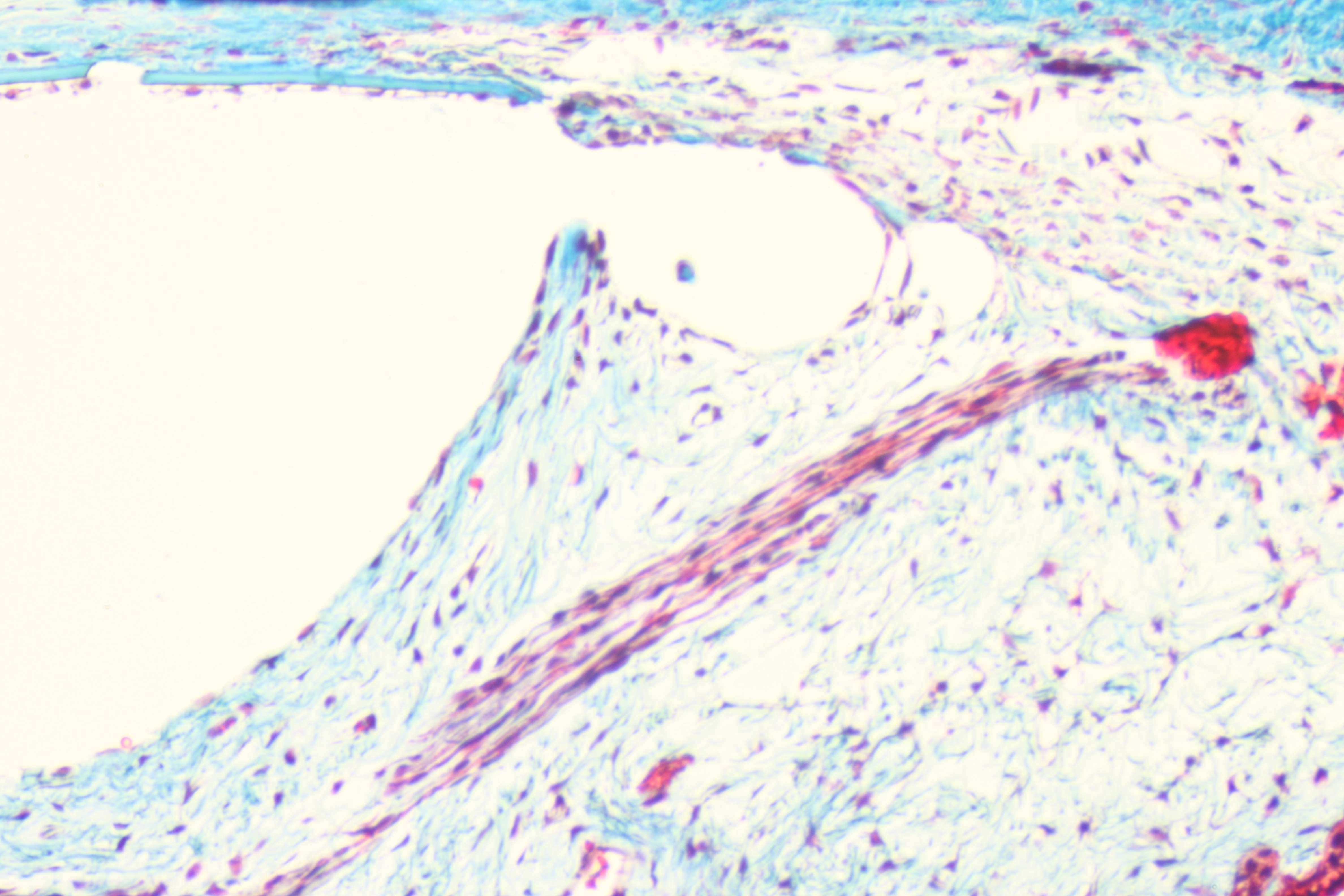

Supplement: S6 Photoset — (ZIP) [file pone.0138054.s007.zip › Multi Tx for Paper - SaratinIlomastatAvastin pics 1/IMG_6195.JPG]

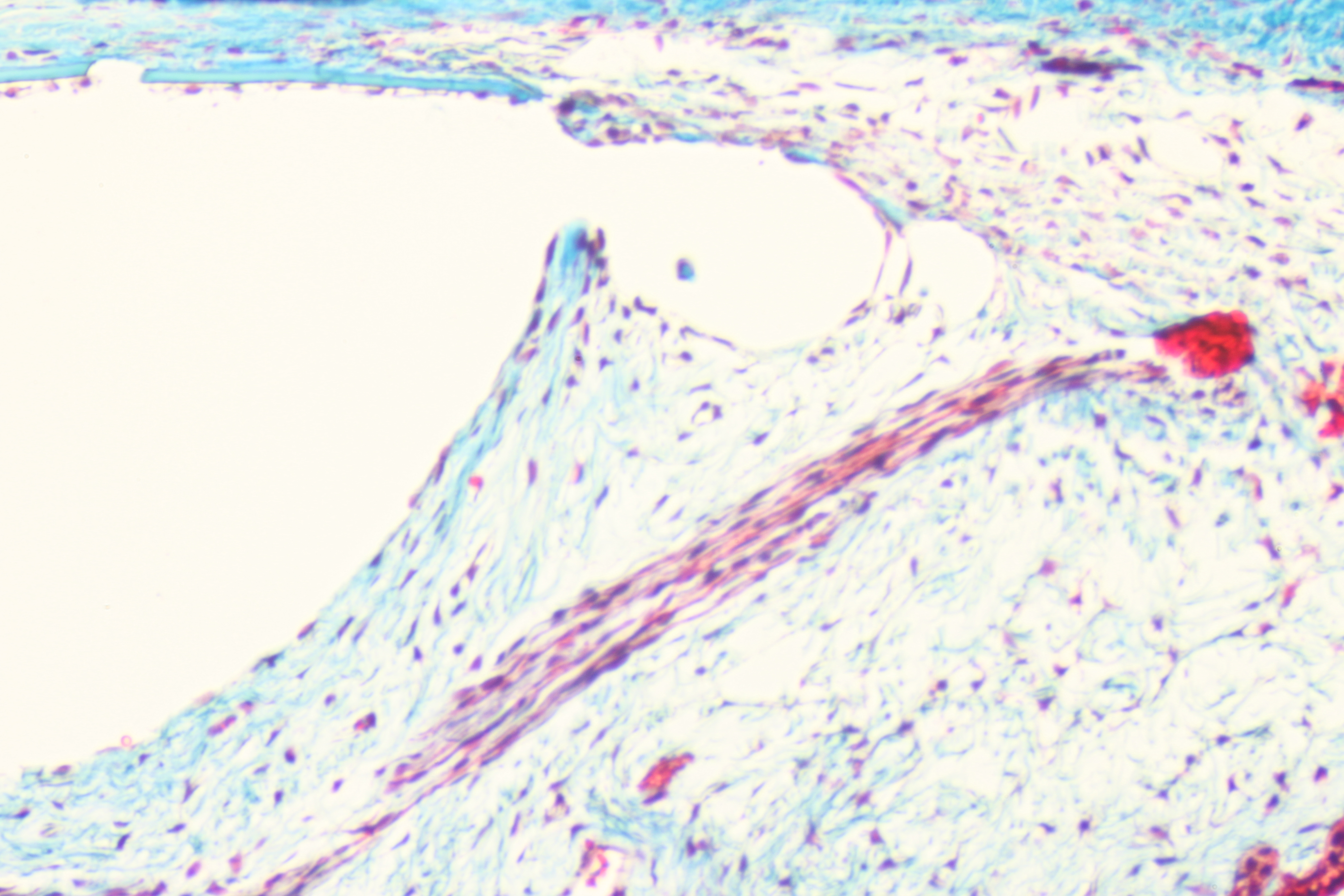

Supplement: S6 Photoset — (ZIP) [file pone.0138054.s007.zip › Multi Tx for Paper - SaratinIlomastatAvastin pics 1/IMG_6196.JPG]

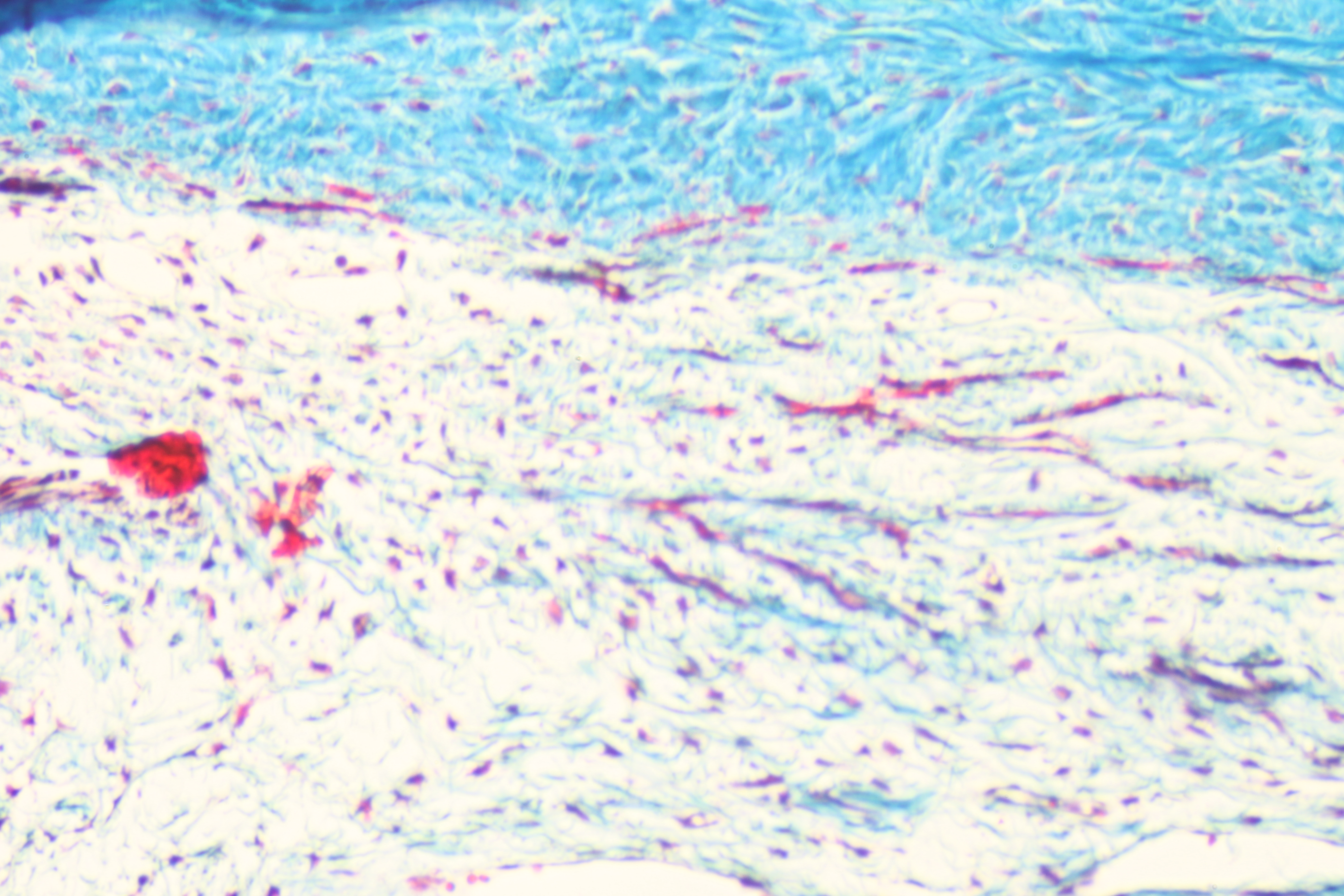

Supplement: S6 Photoset — (ZIP) [file pone.0138054.s007.zip › Multi Tx for Paper - SaratinIlomastatAvastin pics 1/IMG_6197.JPG]

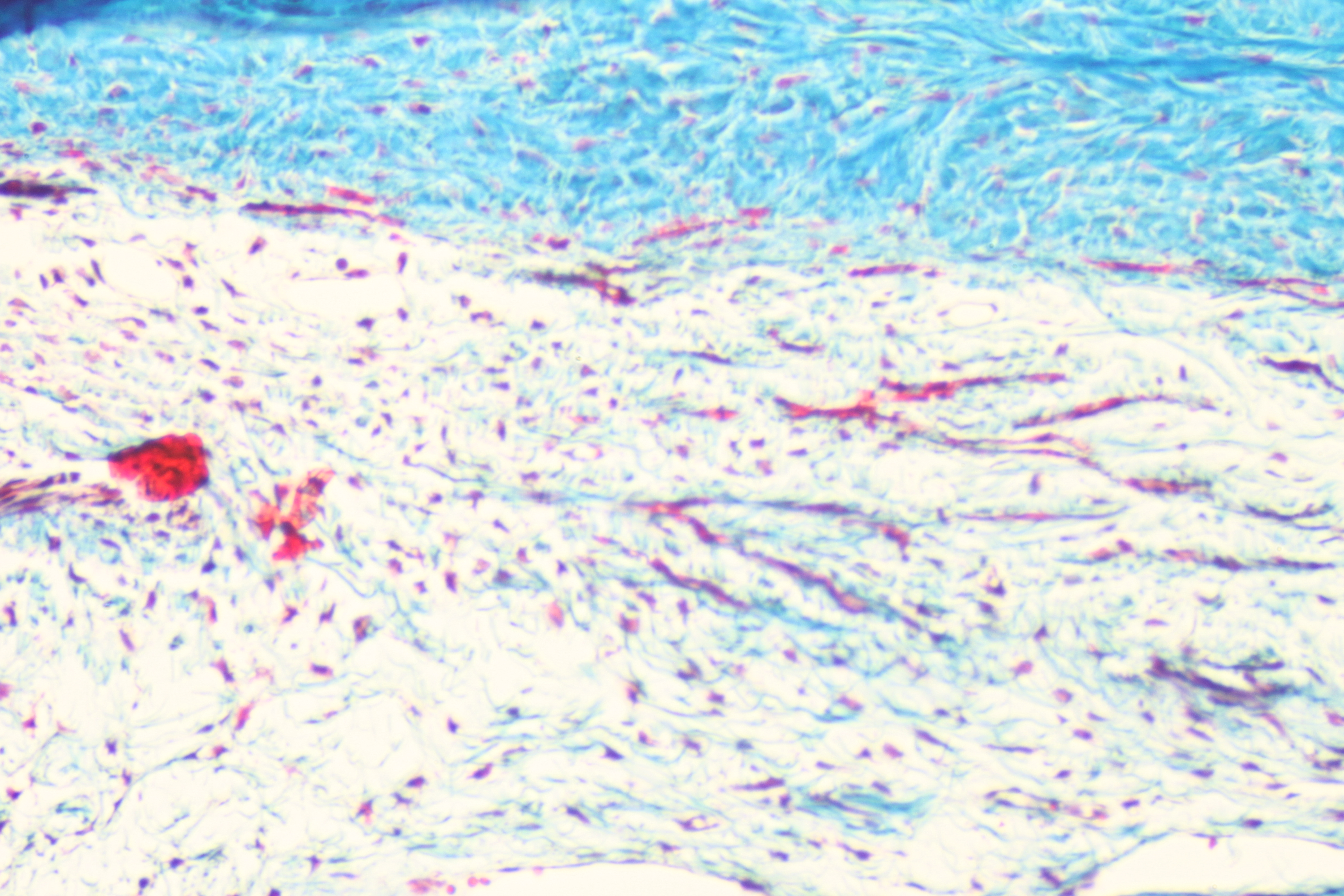

Supplement: S7 Photoset — (ZIP) [file pone.0138054.s008.zip › Multi Tx for Paper - SaratinIlomastatAvastin pics 2/IMG_6198.JPG]

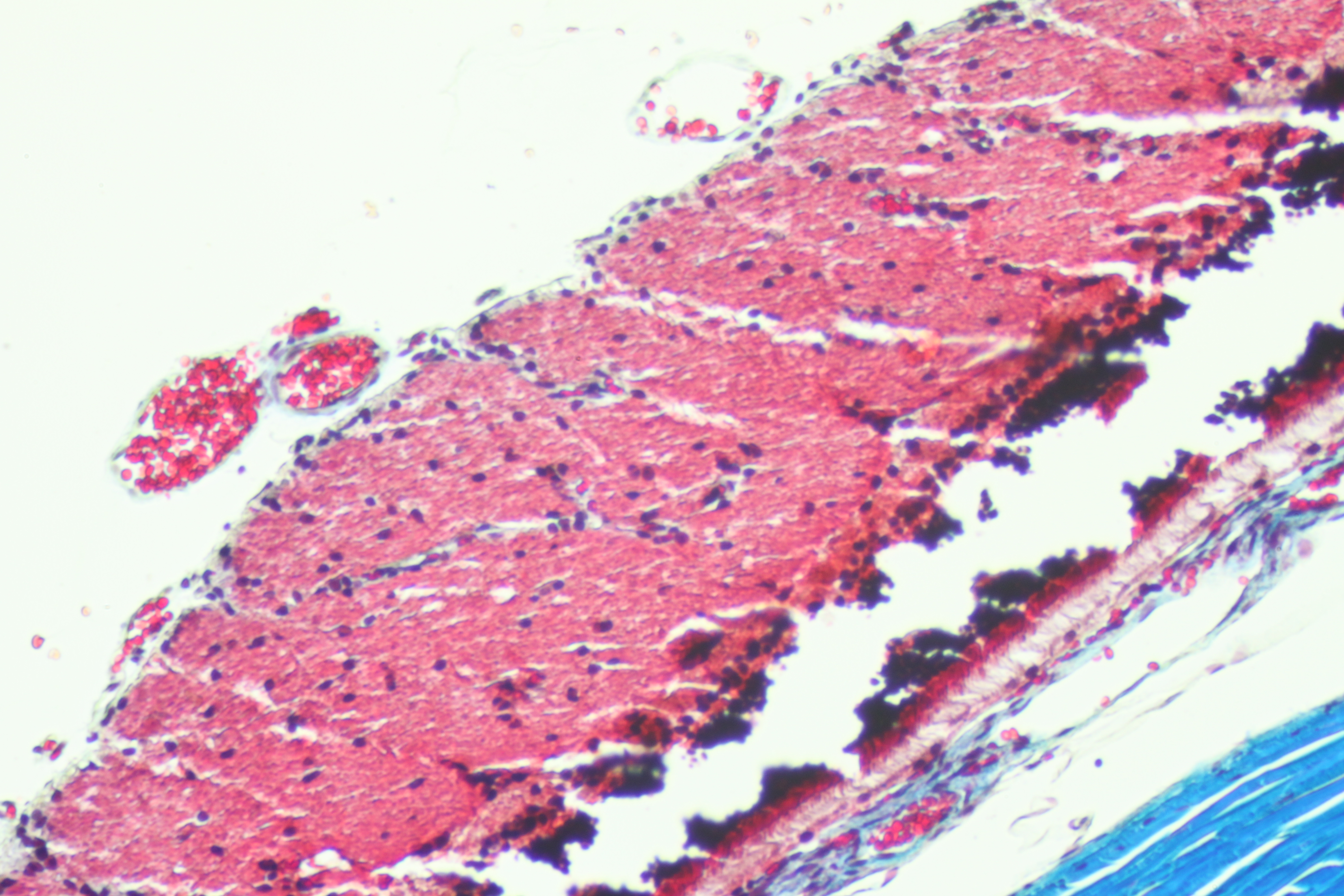

Supplement: S7 Photoset — (ZIP) [file pone.0138054.s008.zip › Multi Tx for Paper - SaratinIlomastatAvastin pics 2/IMG_6199.JPG]

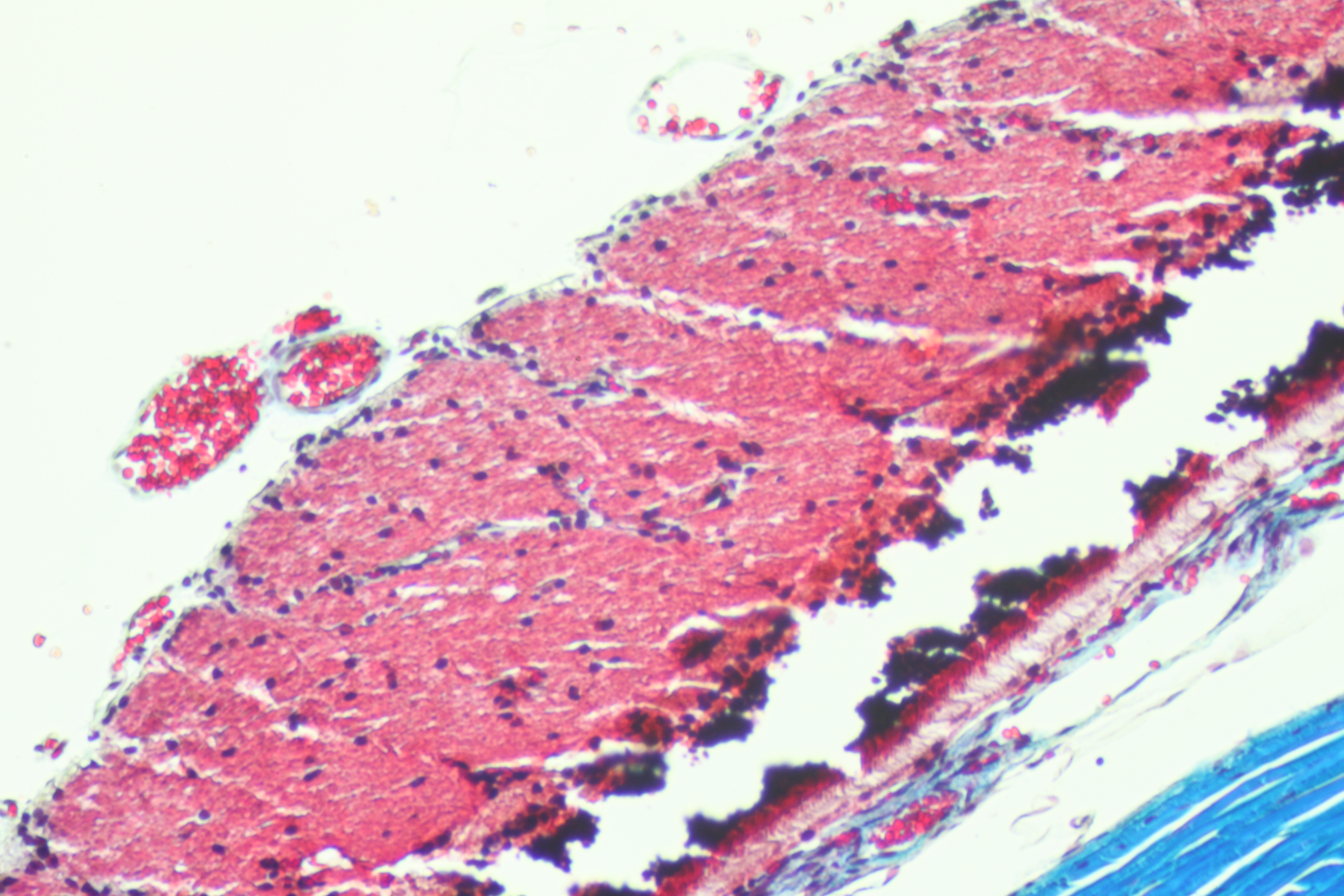

Supplement: S7 Photoset — (ZIP) [file pone.0138054.s008.zip › Multi Tx for Paper - SaratinIlomastatAvastin pics 2/IMG_6200.JPG]

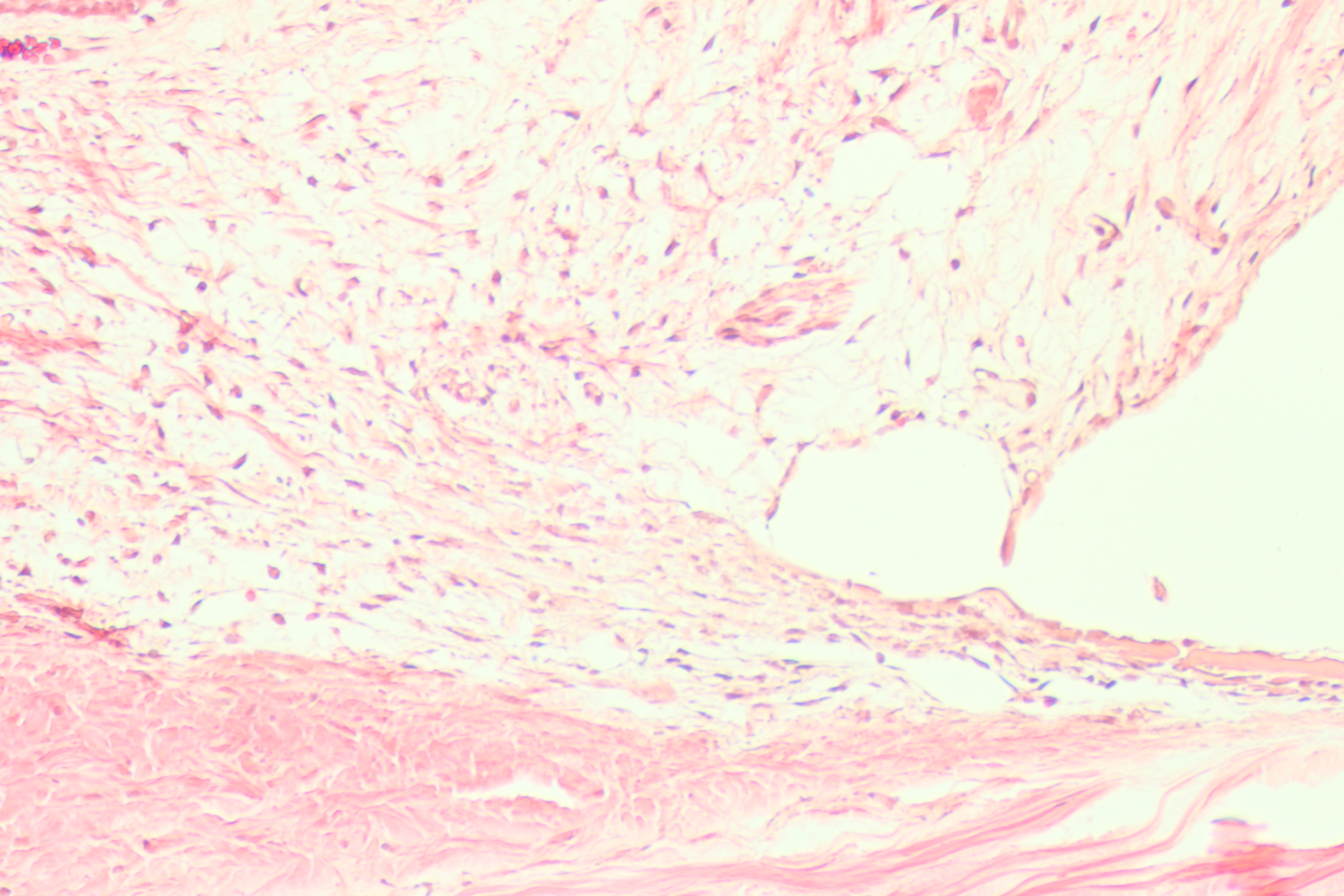

Supplement: S7 Photoset — (ZIP) [file pone.0138054.s008.zip › Multi Tx for Paper - SaratinIlomastatAvastin pics 2/IMG_6297.JPG]

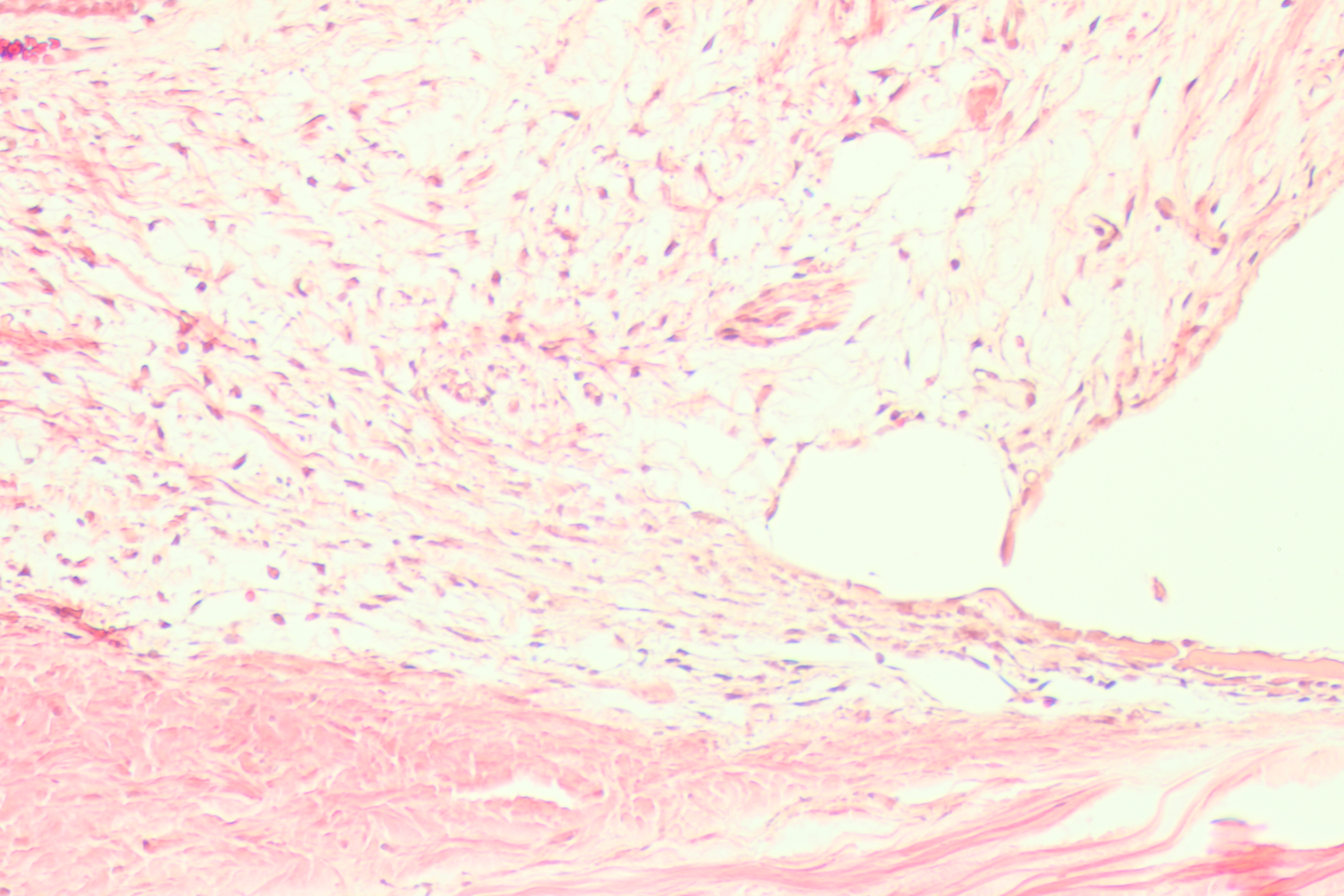

Supplement: S7 Photoset — (ZIP) [file pone.0138054.s008.zip › Multi Tx for Paper - SaratinIlomastatAvastin pics 2/IMG_6298.JPG]

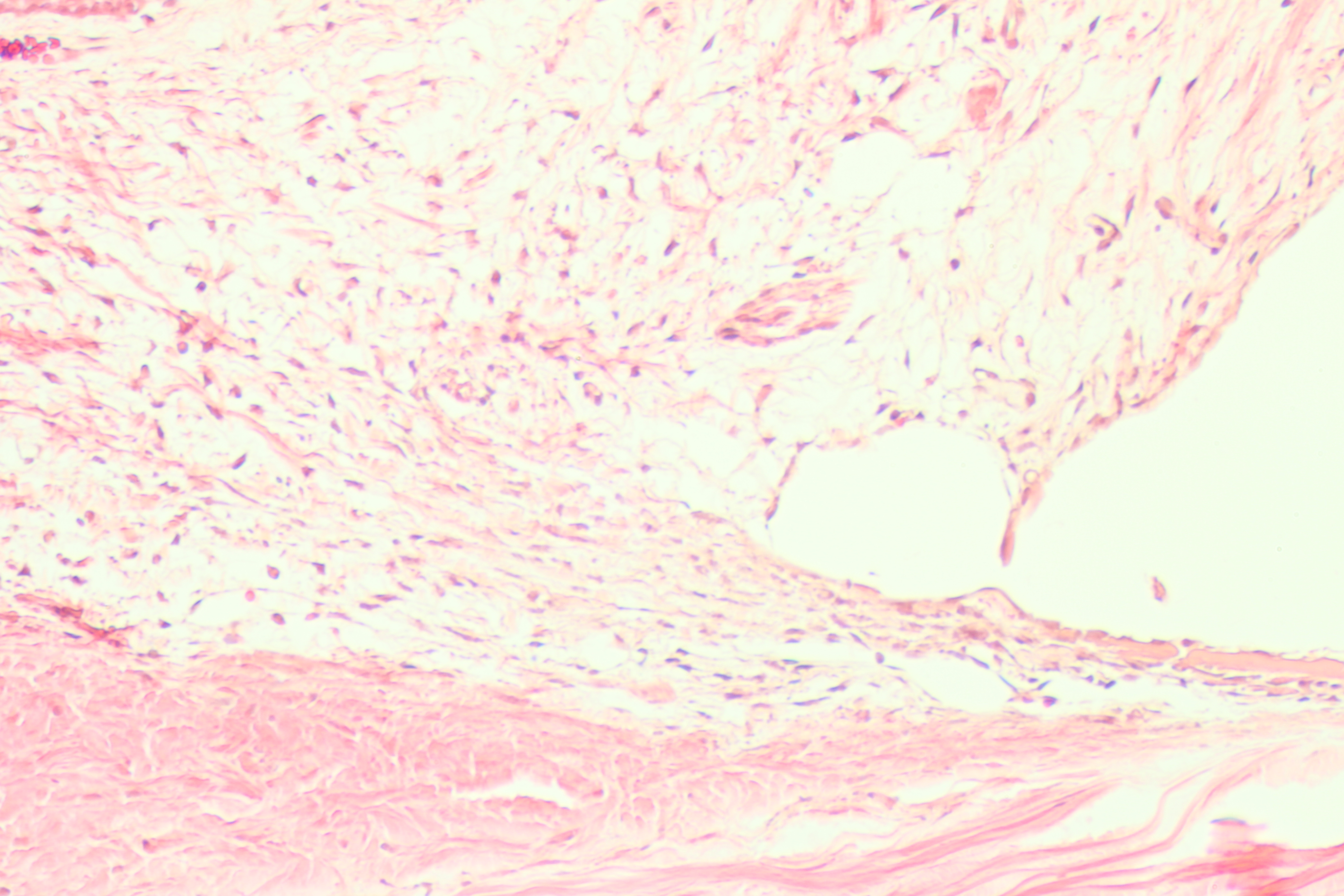

Supplement: S7 Photoset — (ZIP) [file pone.0138054.s008.zip › Multi Tx for Paper - SaratinIlomastatAvastin pics 2/IMG_6299.JPG]

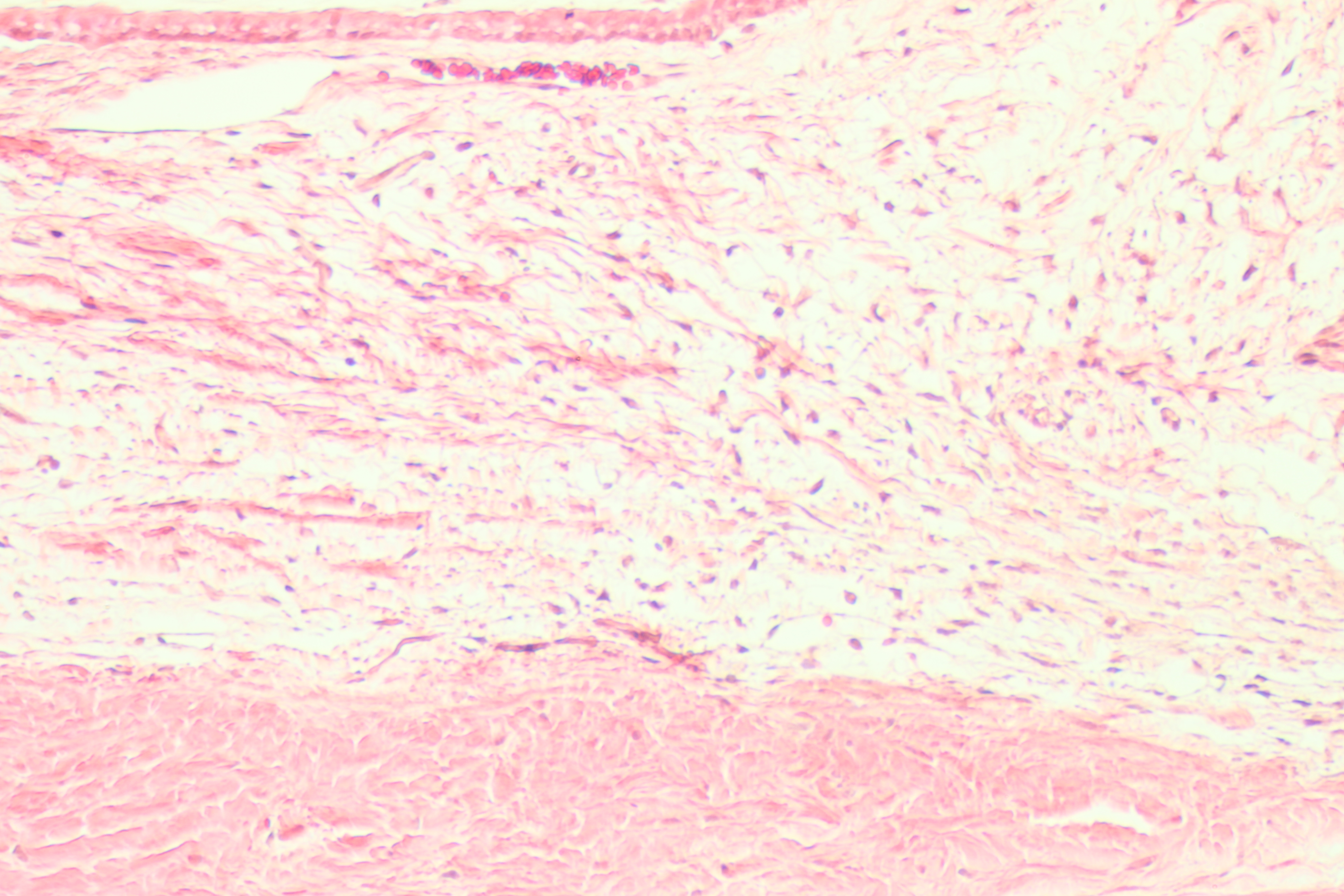

Supplement: S7 Photoset — (ZIP) [file pone.0138054.s008.zip › Multi Tx for Paper - SaratinIlomastatAvastin pics 2/IMG_6300.JPG]

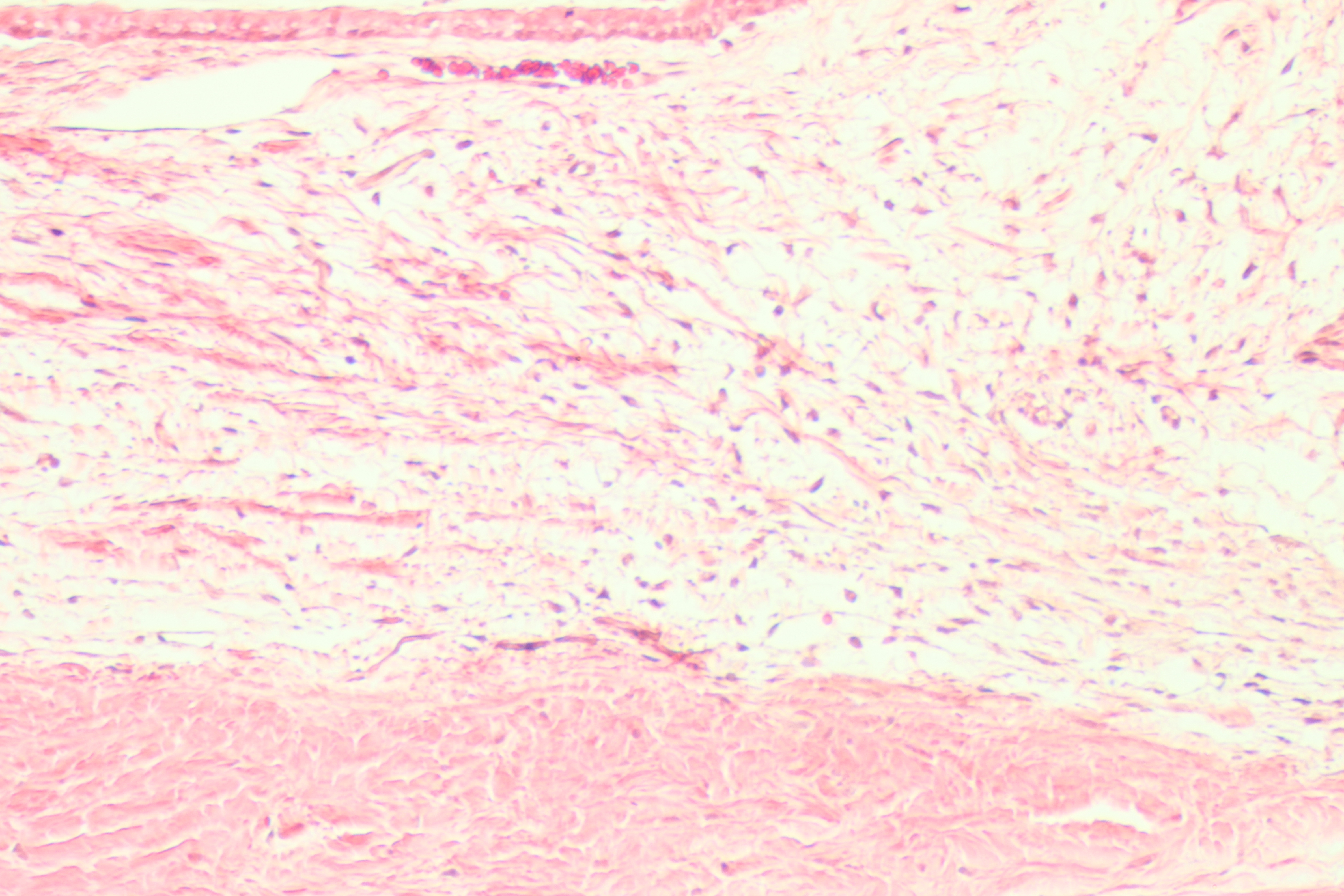

Supplement: S7 Photoset — (ZIP) [file pone.0138054.s008.zip › Multi Tx for Paper - SaratinIlomastatAvastin pics 2/IMG_6301.JPG]

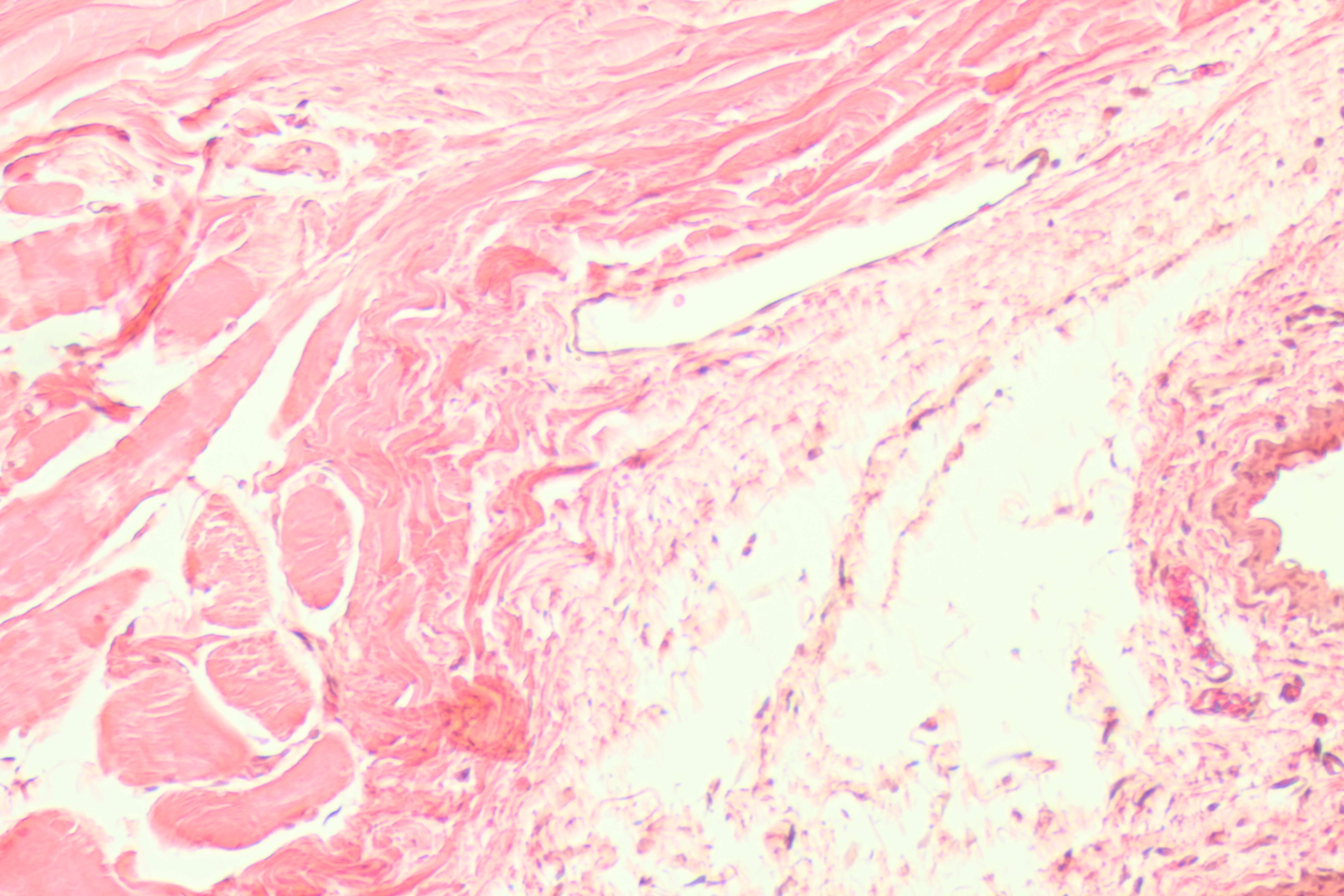

Supplement: S7 Photoset — (ZIP) [file pone.0138054.s008.zip › Multi Tx for Paper - SaratinIlomastatAvastin pics 2/IMG_6302.JPG]

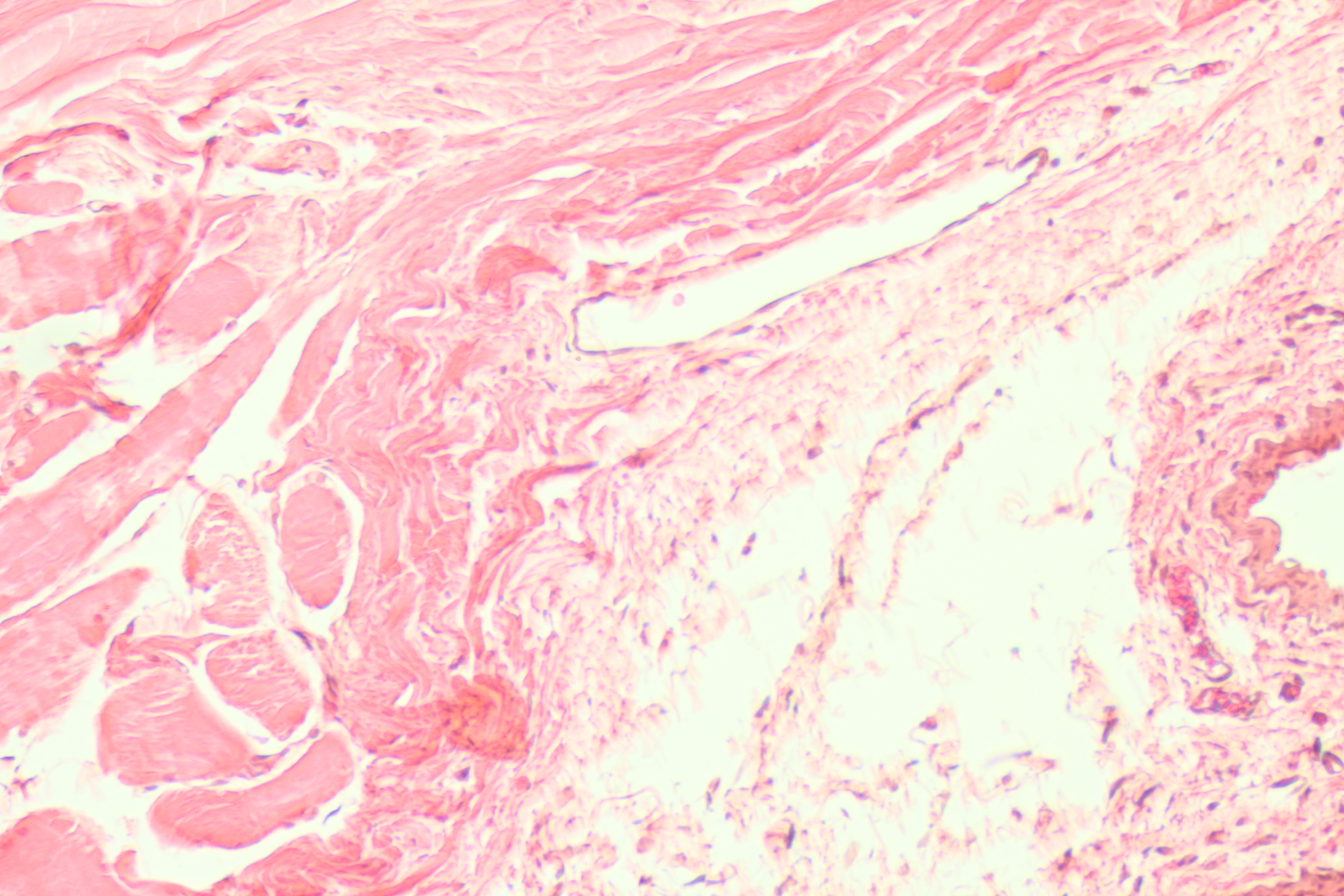

Supplement: S7 Photoset — (ZIP) [file pone.0138054.s008.zip › Multi Tx for Paper - SaratinIlomastatAvastin pics 2/IMG_6303.JPG]

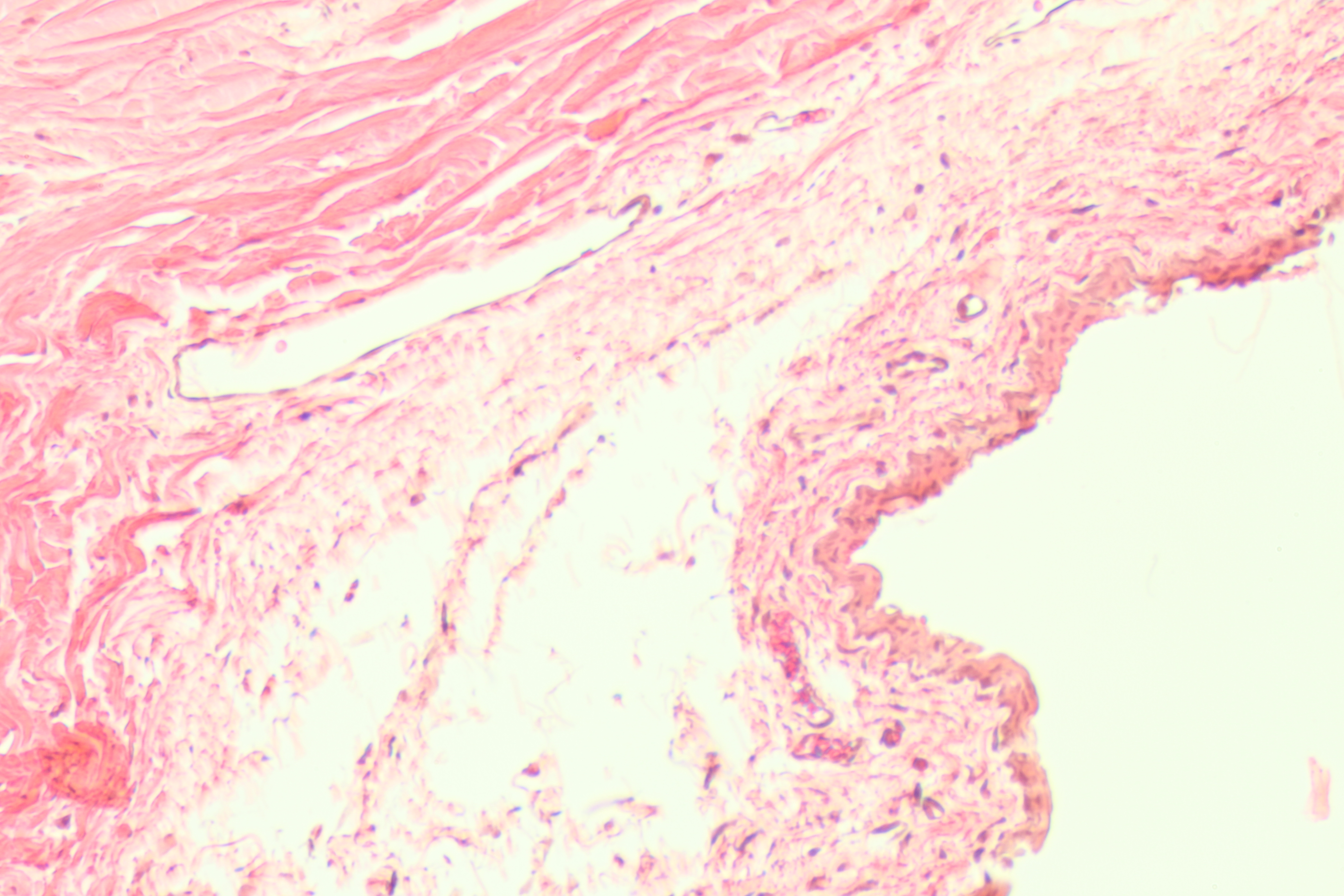

Supplement: S7 Photoset — (ZIP) [file pone.0138054.s008.zip › Multi Tx for Paper - SaratinIlomastatAvastin pics 2/IMG_6304.JPG]

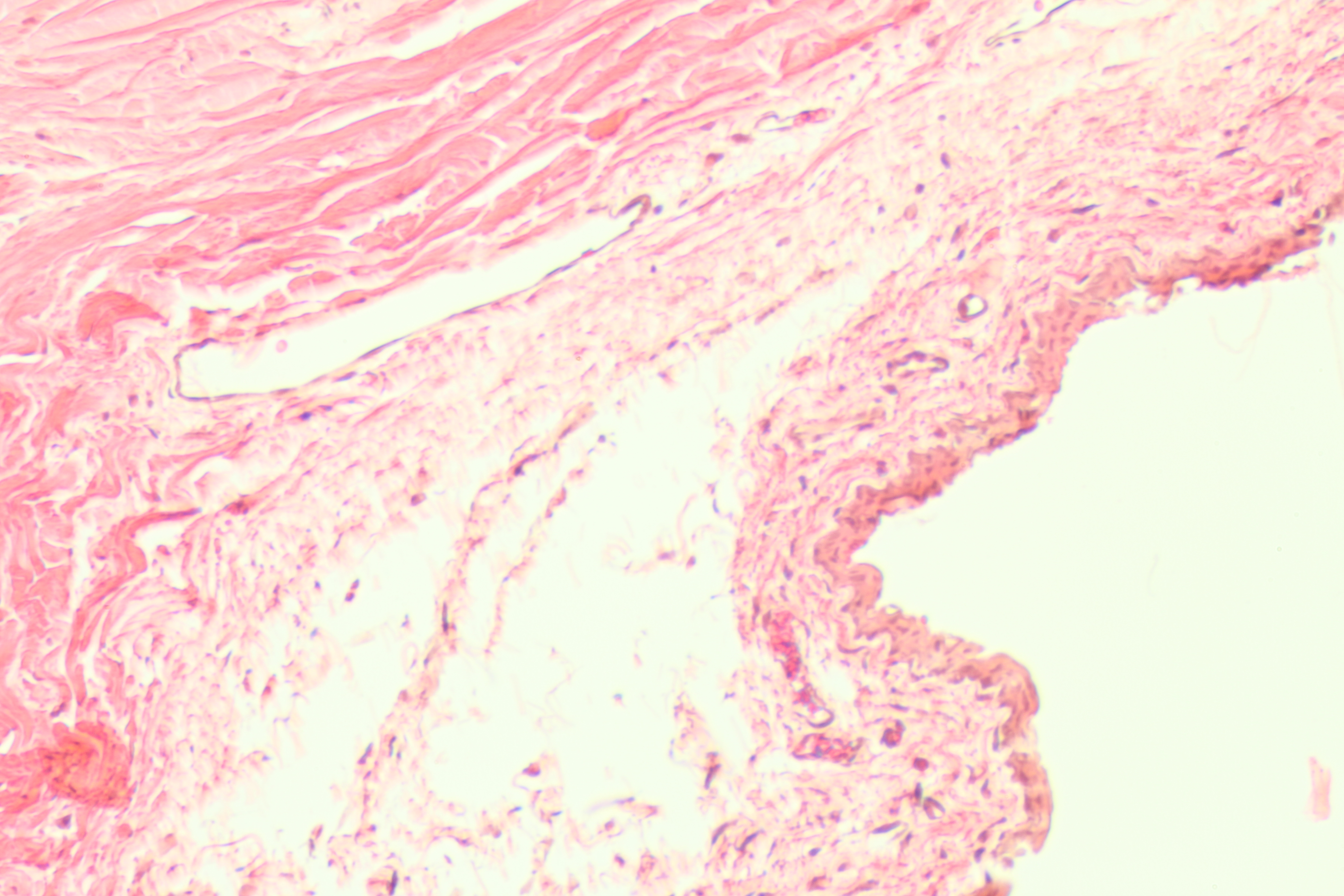

Supplement: S7 Photoset — (ZIP) [file pone.0138054.s008.zip › Multi Tx for Paper - SaratinIlomastatAvastin pics 2/IMG_6305.JPG]

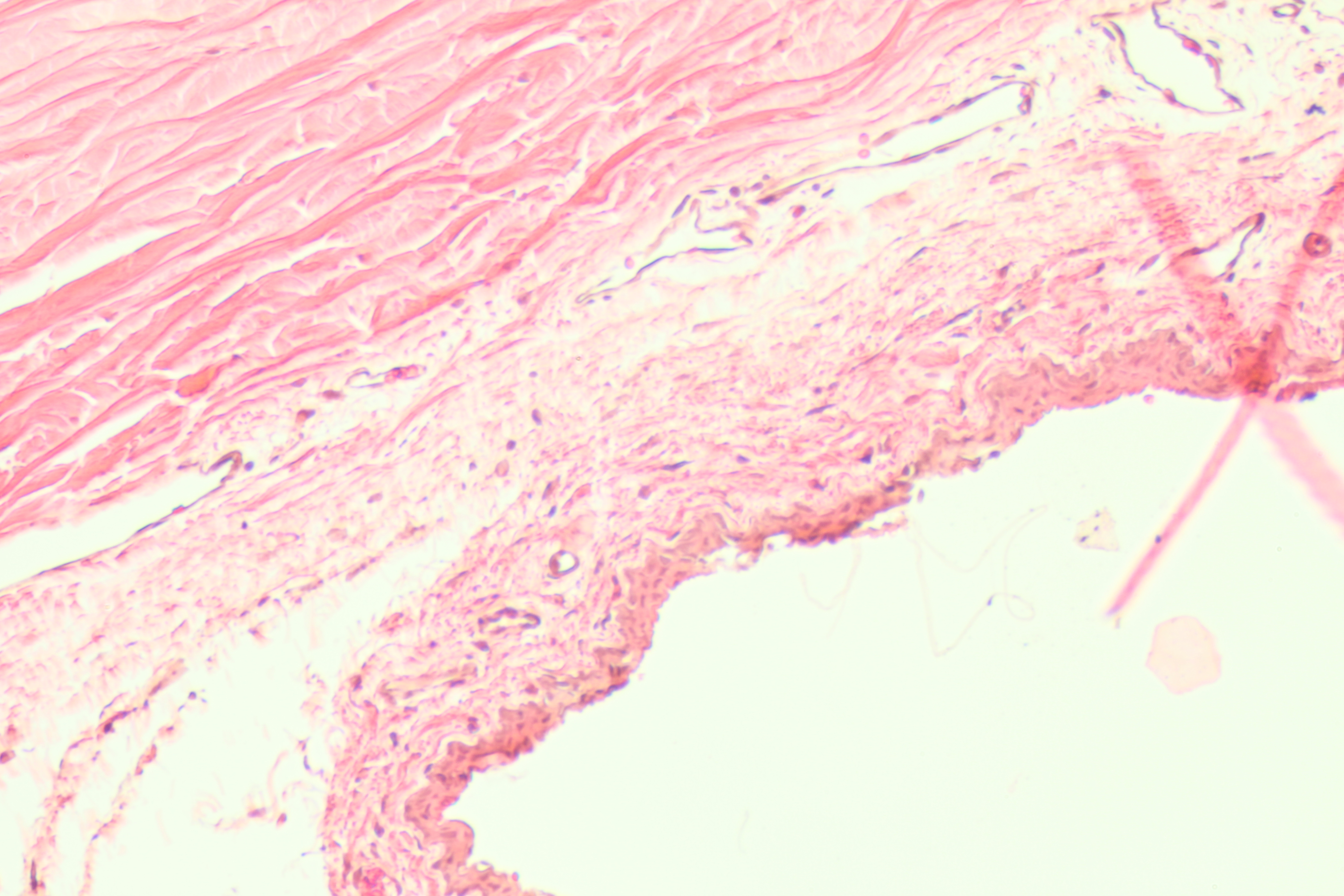

Supplement: S7 Photoset — (ZIP) [file pone.0138054.s008.zip › Multi Tx for Paper - SaratinIlomastatAvastin pics 2/IMG_6306.JPG]

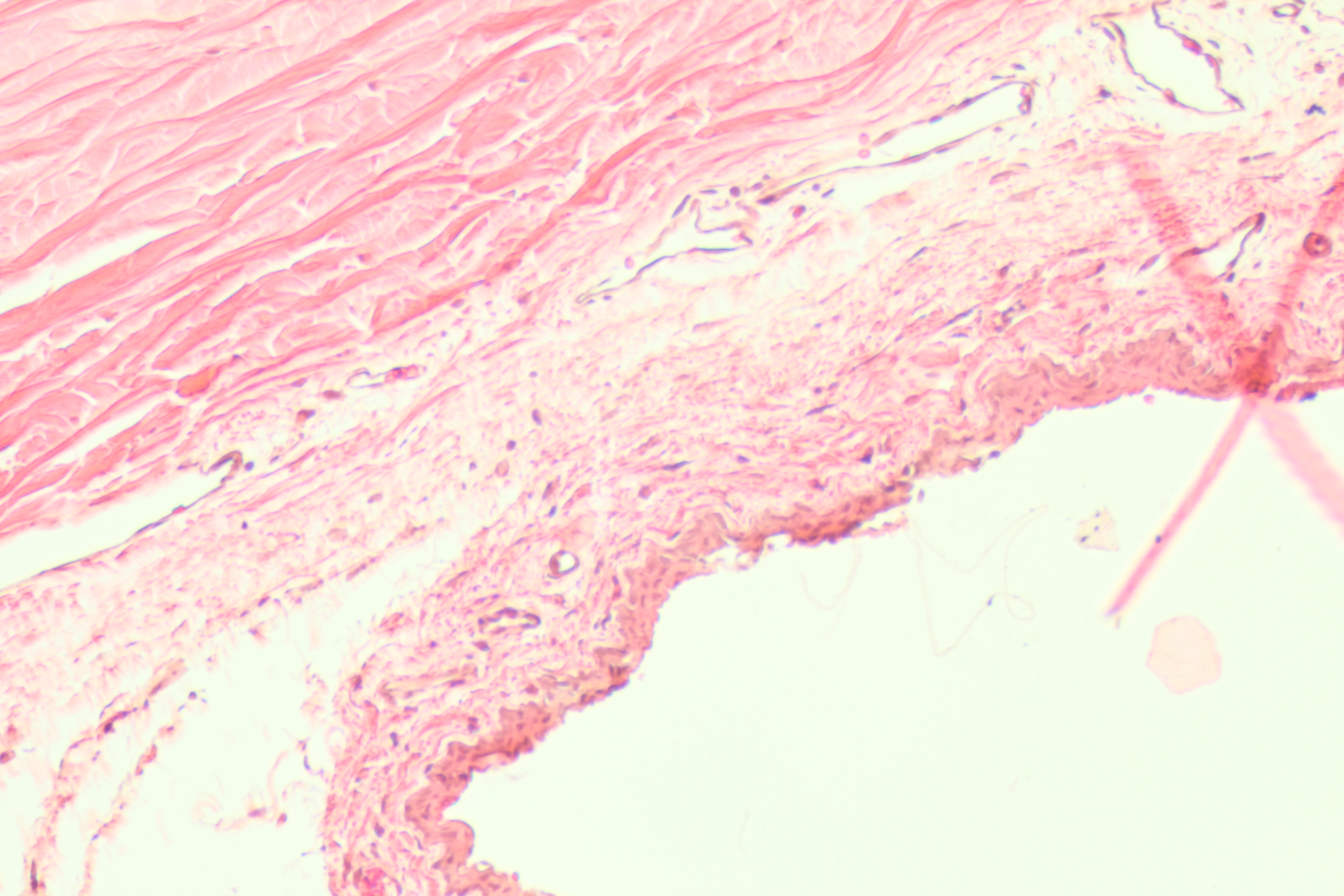

Supplement: S7 Photoset — (ZIP) [file pone.0138054.s008.zip › Multi Tx for Paper - SaratinIlomastatAvastin pics 2/IMG_6307.JPG]

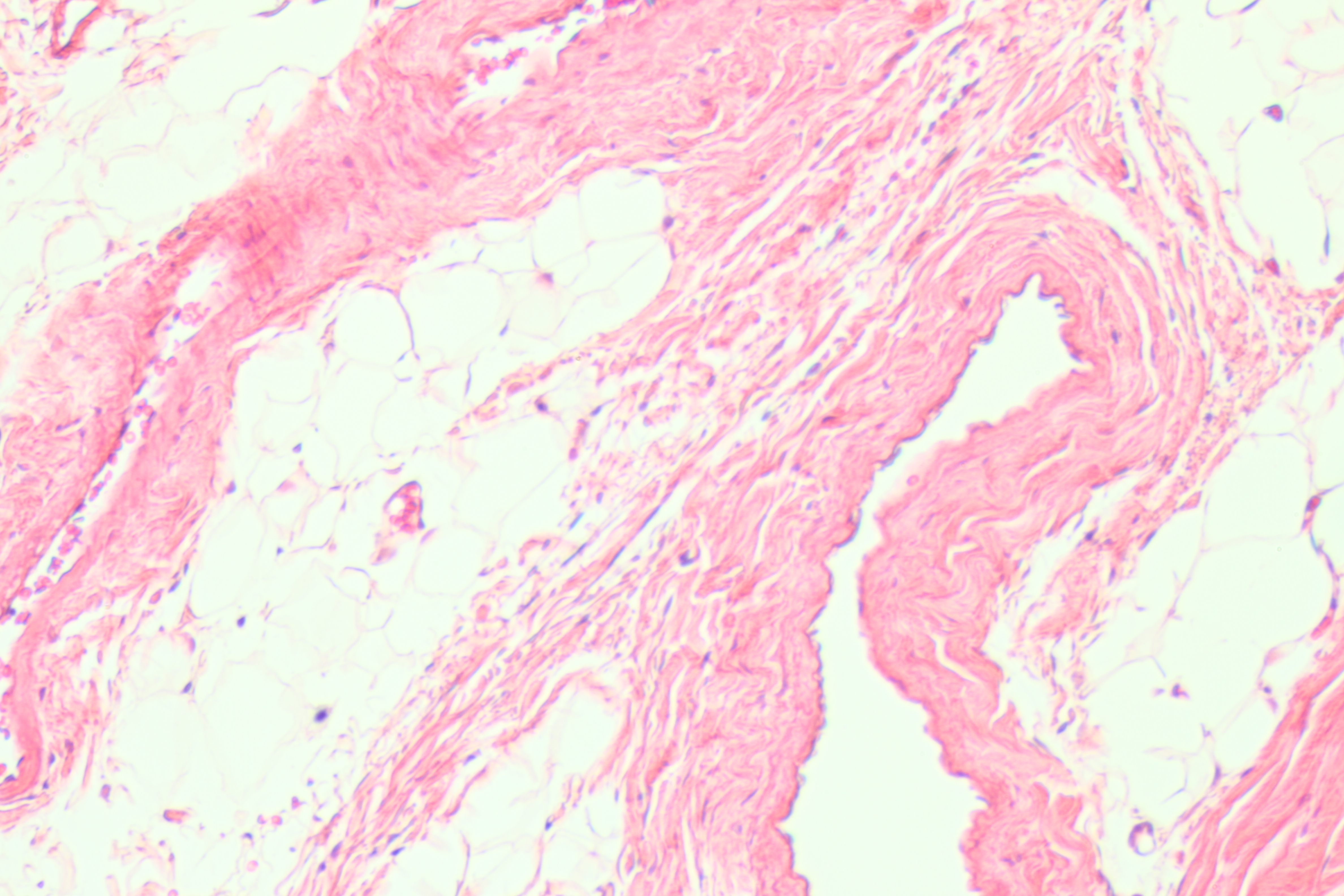

Supplement: S7 Photoset — (ZIP) [file pone.0138054.s008.zip › Multi Tx for Paper - SaratinIlomastatAvastin pics 2/IMG_6320.JPG]

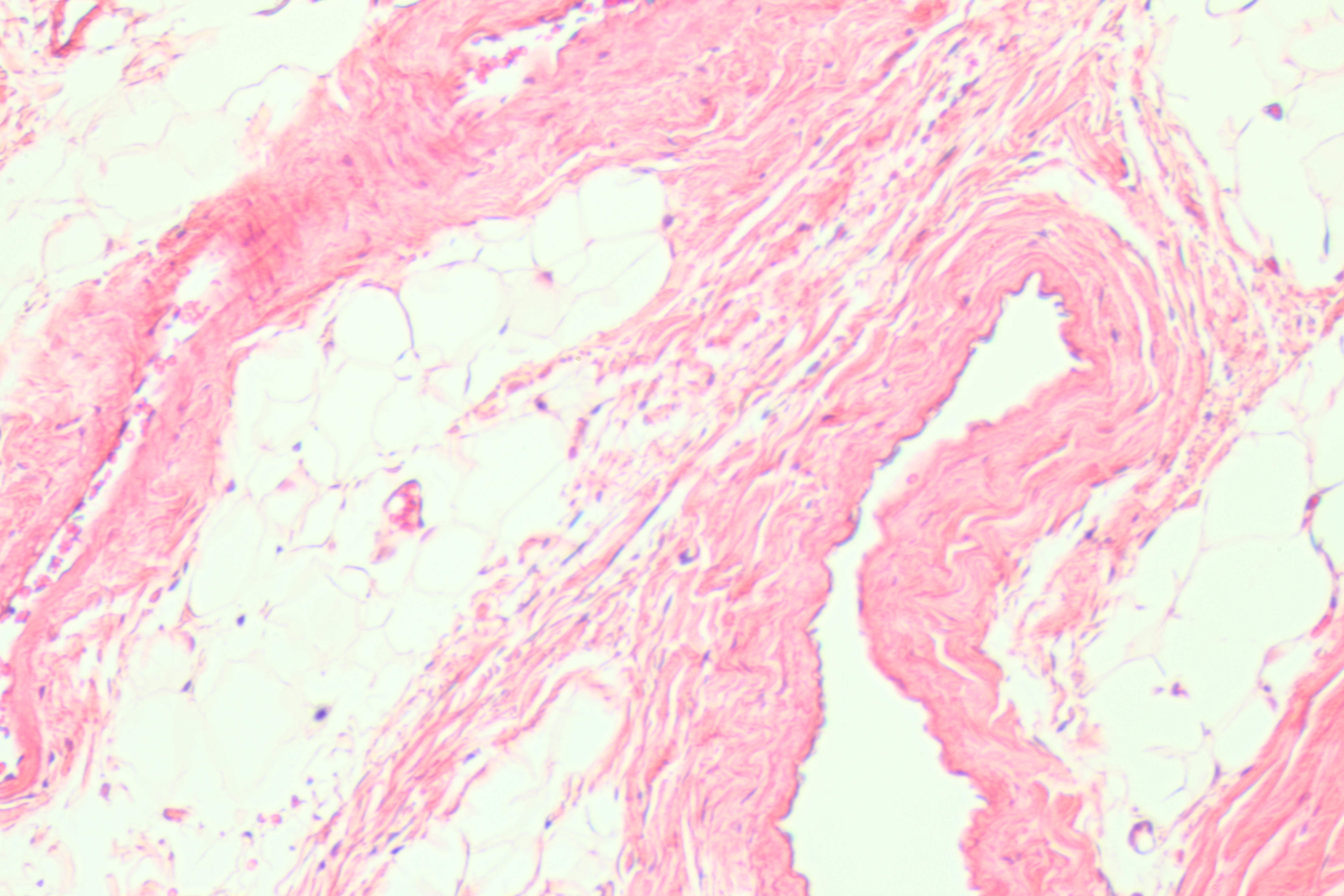

Supplement: S7 Photoset — (ZIP) [file pone.0138054.s008.zip › Multi Tx for Paper - SaratinIlomastatAvastin pics 2/IMG_6321.JPG]

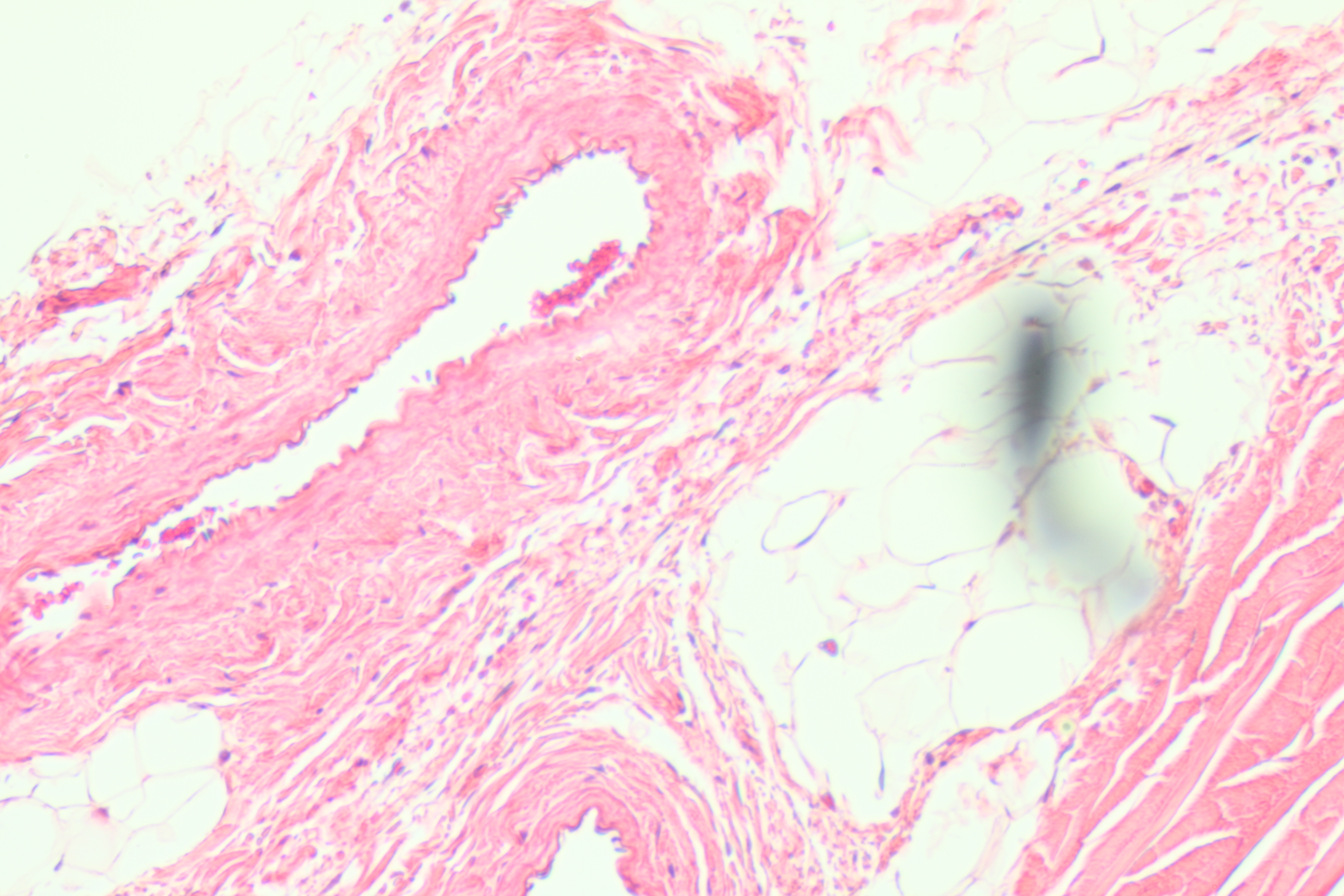

Supplement: S7 Photoset — (ZIP) [file pone.0138054.s008.zip › Multi Tx for Paper - SaratinIlomastatAvastin pics 2/IMG_6322.JPG]

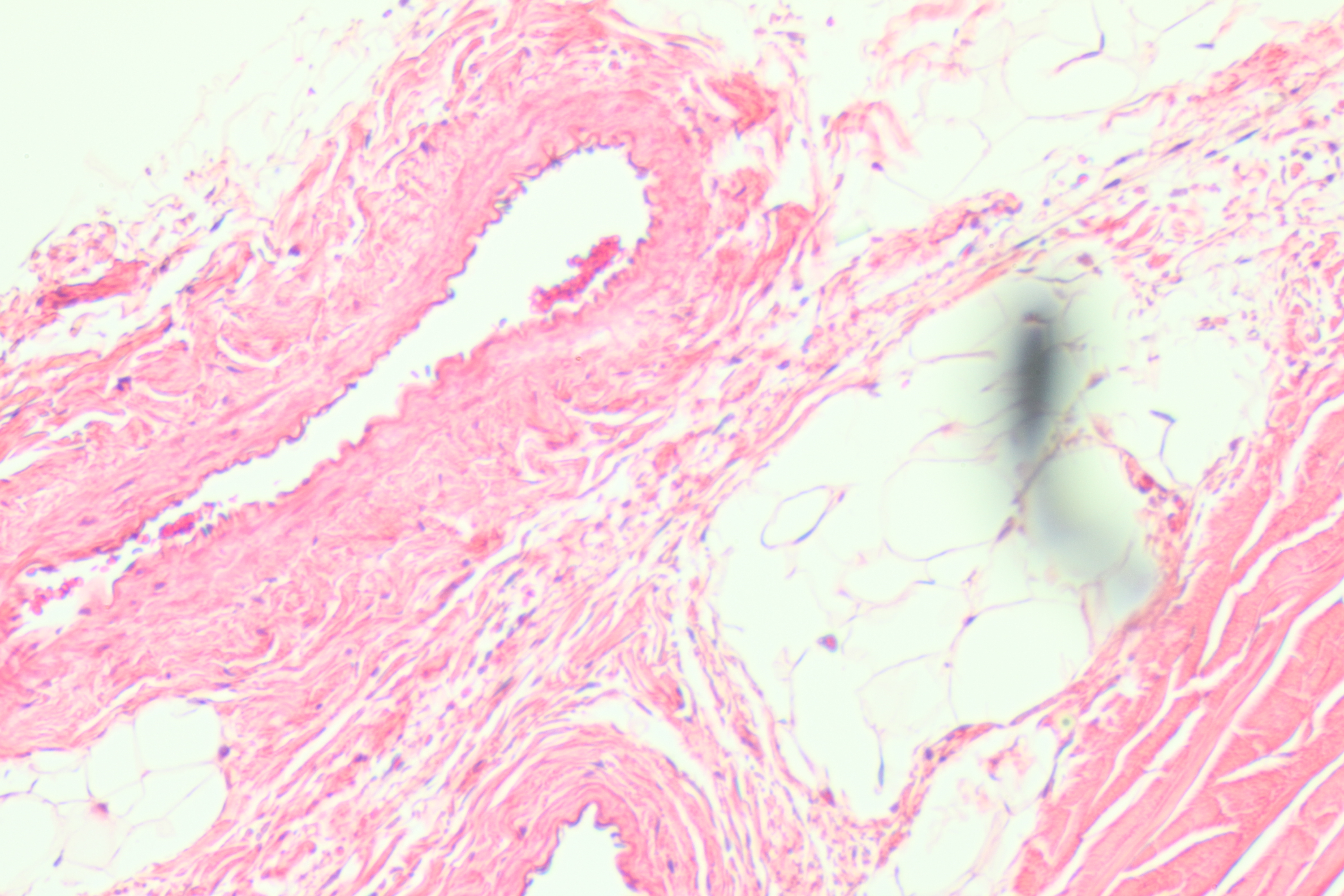

Supplement: S7 Photoset — (ZIP) [file pone.0138054.s008.zip › Multi Tx for Paper - SaratinIlomastatAvastin pics 2/IMG_6323.JPG]

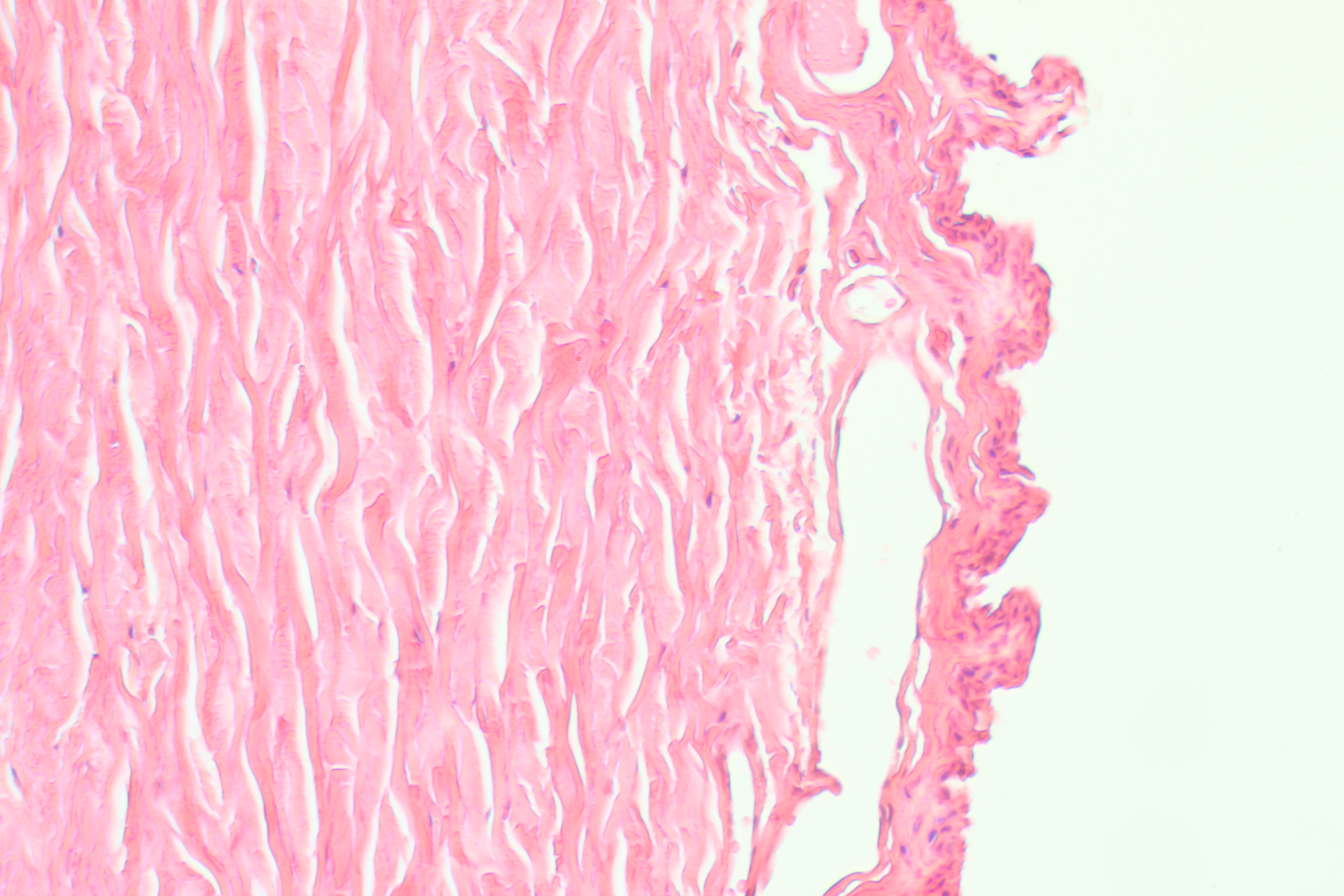

Supplement: S7 Photoset — (ZIP) [file pone.0138054.s008.zip › Multi Tx for Paper - SaratinIlomastatAvastin pics 2/IMG_6324.JPG]

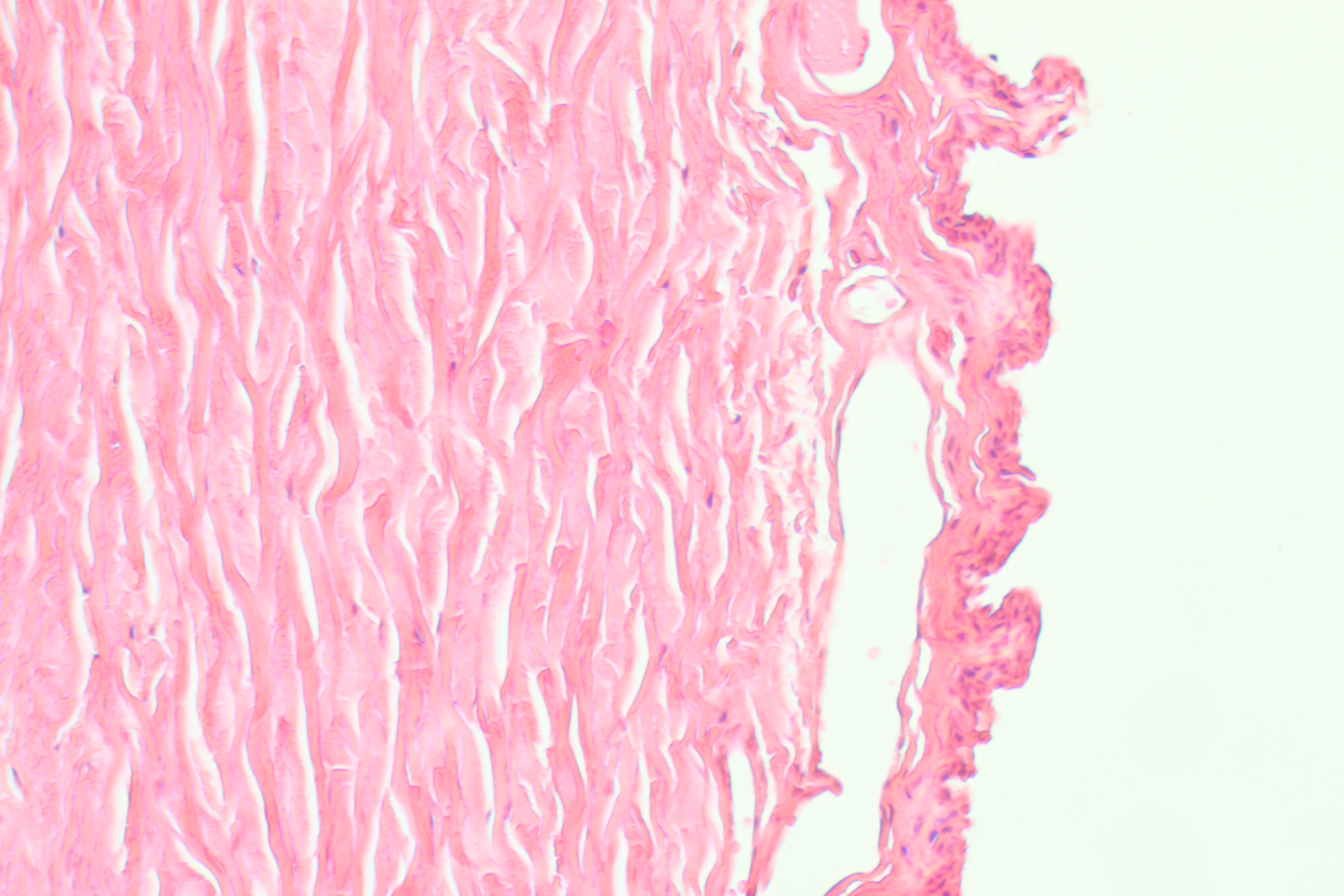

Supplement: S7 Photoset — (ZIP) [file pone.0138054.s008.zip › Multi Tx for Paper - SaratinIlomastatAvastin pics 2/IMG_6325.JPG]

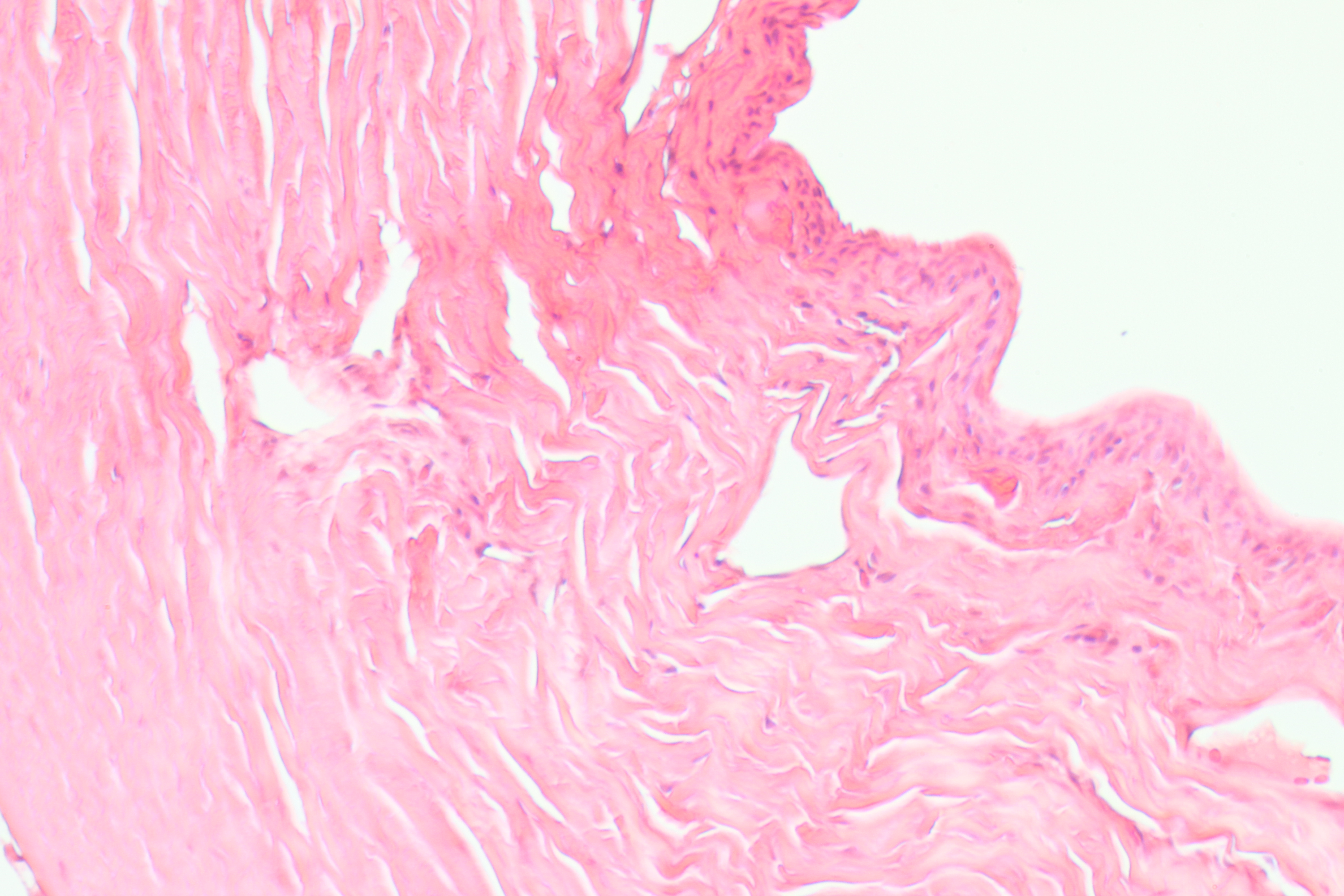

Supplement: S7 Photoset — (ZIP) [file pone.0138054.s008.zip › Multi Tx for Paper - SaratinIlomastatAvastin pics 2/IMG_6326.JPG]

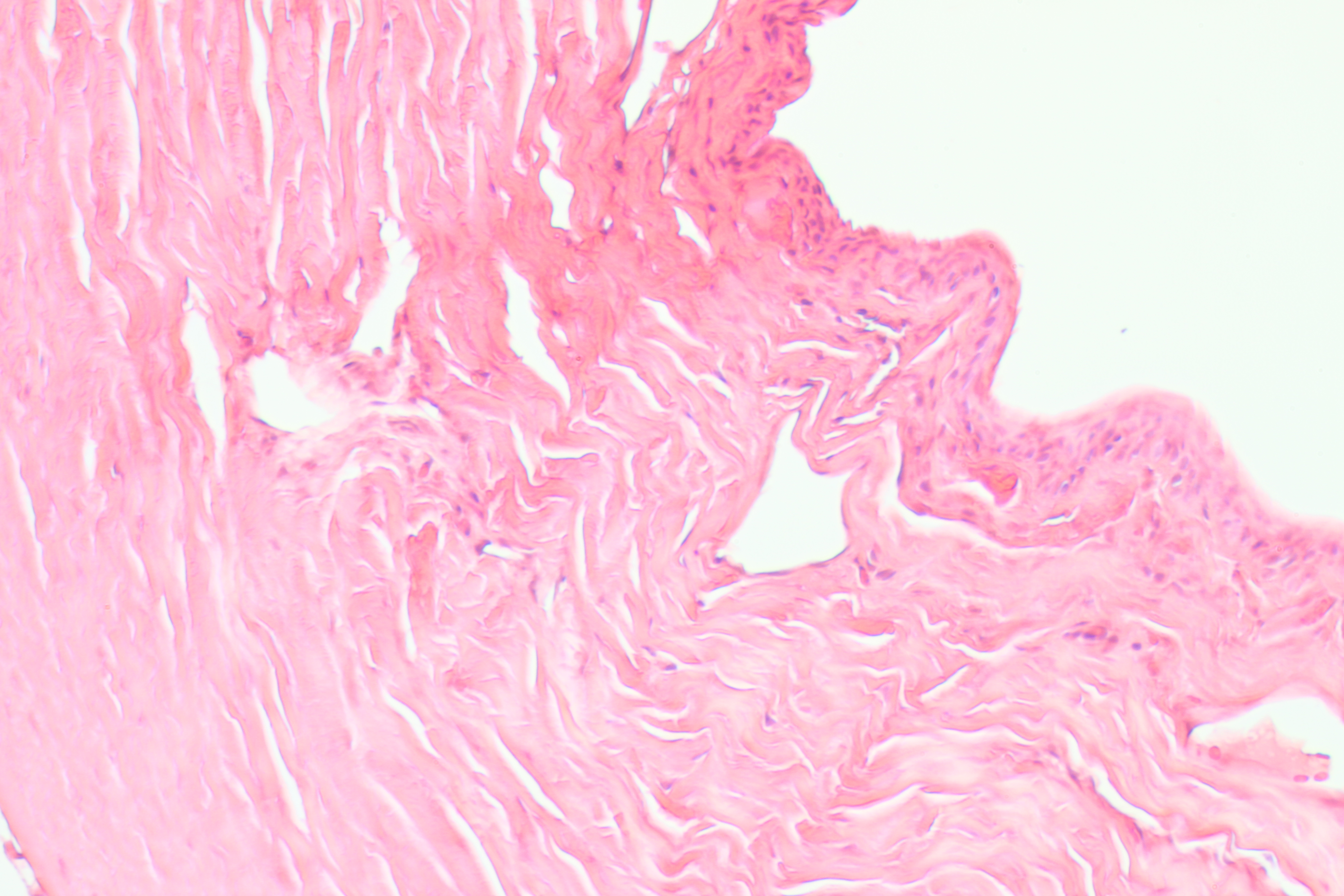

Supplement: S7 Photoset — (ZIP) [file pone.0138054.s008.zip › Multi Tx for Paper - SaratinIlomastatAvastin pics 2/IMG_6327.JPG]

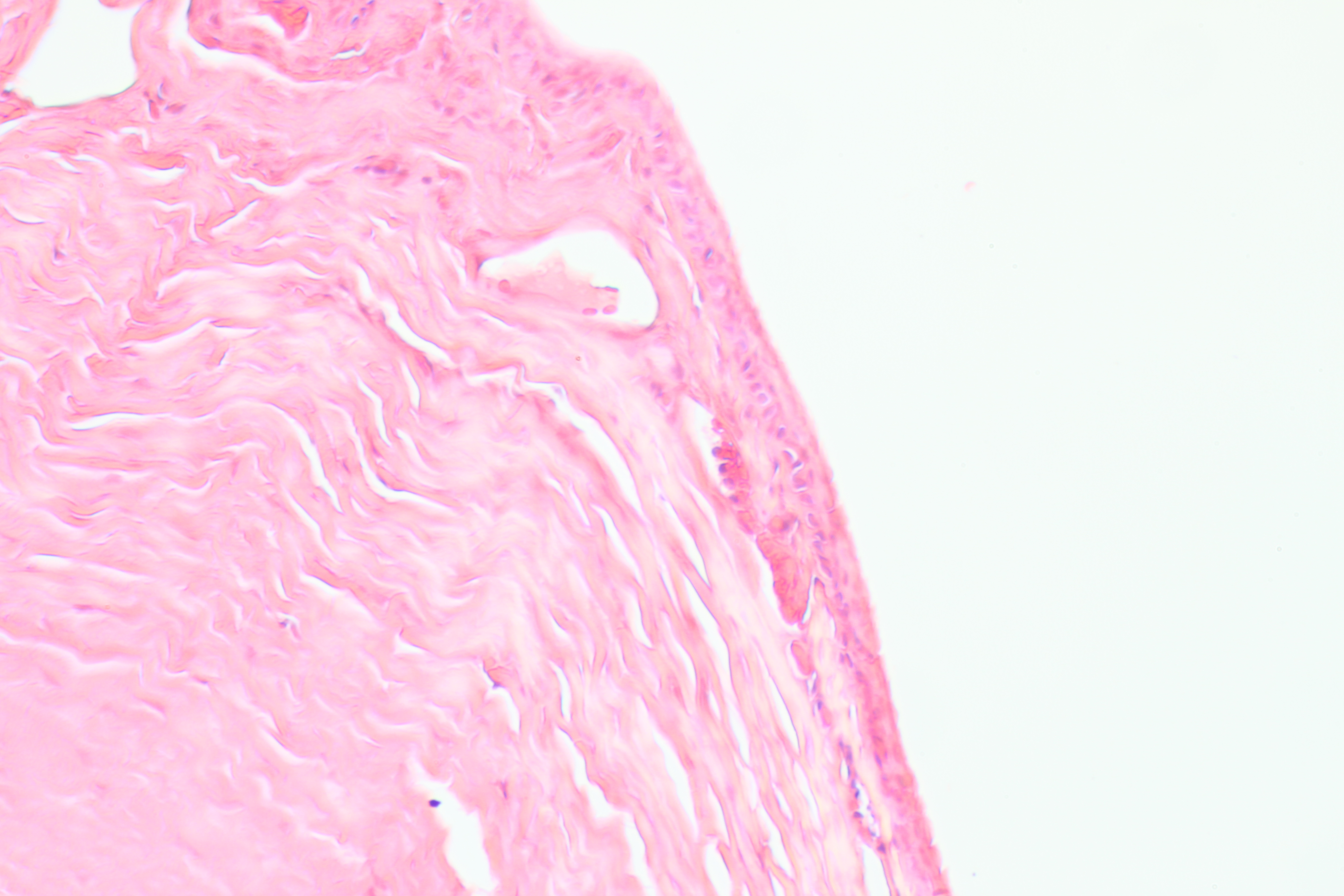

Supplement: S7 Photoset — (ZIP) [file pone.0138054.s008.zip › Multi Tx for Paper - SaratinIlomastatAvastin pics 2/IMG_6328.JPG]

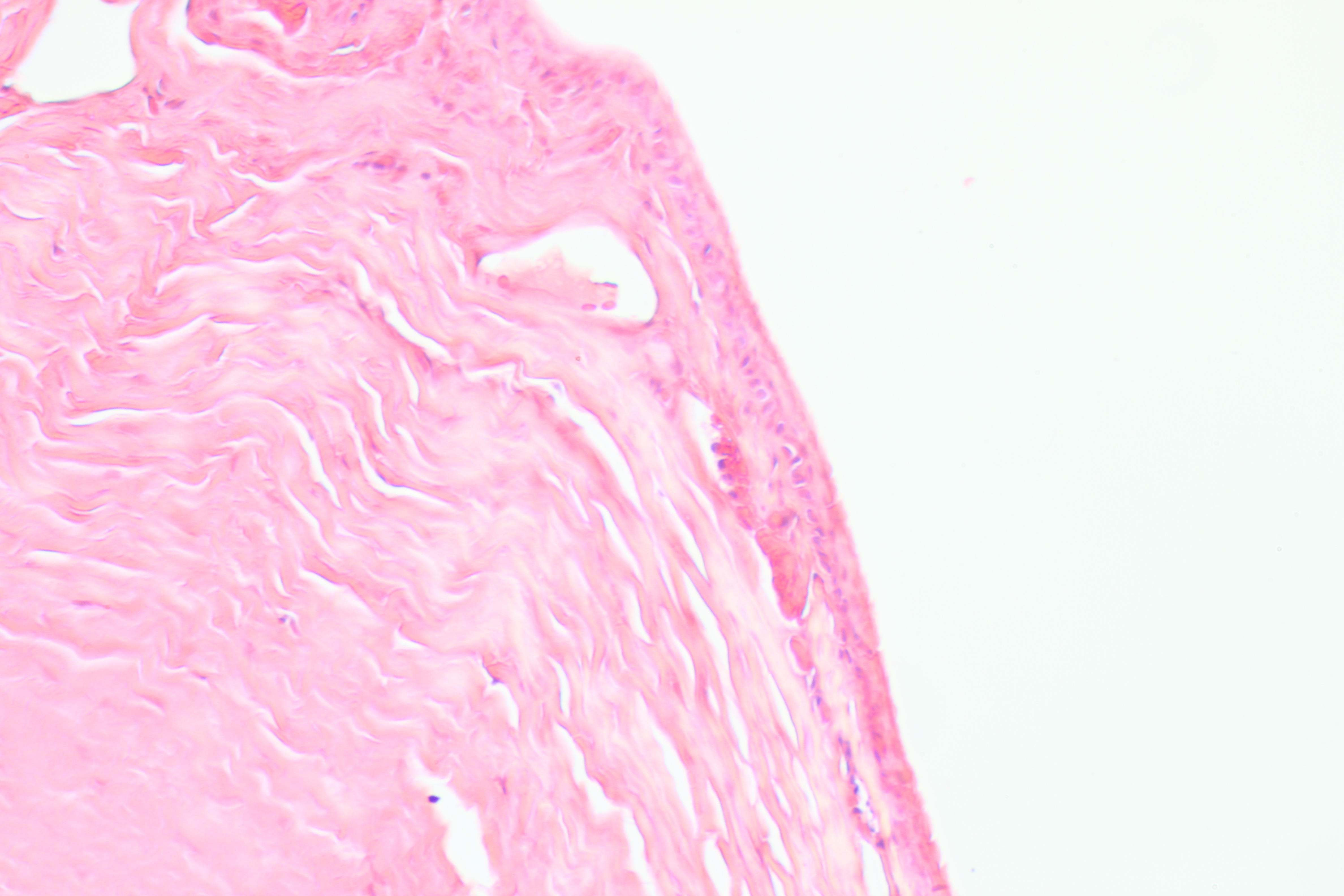

Supplement: S7 Photoset — (ZIP) [file pone.0138054.s008.zip › Multi Tx for Paper - SaratinIlomastatAvastin pics 2/IMG_6329.JPG]
